# Supplementary material for: Iridium-catalyzed direct asymmetric reductive amination utilizing primary alkyl amines as the N-sources
Source: Nat Commun. 2022 Jun 10;13:3344. doi: 10.1038/s41467-022-31045-5 (PMC9187633; doi:10.1038/s41467-022-31045-5)
Supplement: Supplementary file 1 — Supplementary [file 41467_2022_31045_MOESM1_ESM.pdf]

Supplementary Information for

Iridium-Catalyzed Direct Asymmetric Reductive Amination Utilizing Primary Alkyl Amines as the N-sources

Zitong Wu,[1,2†] Wenji Wang,[1†] Haodong Guo,[1] Guorui Gao,[3] Haizhou Huang[1] and Mingxin Chang*[1,2]

[1]College of Chemistry & Pharmacy, Northwest A&F University, Yangling, Shaanxi 712100, China.
[2]College of Plant Protection, Shaanxi Research Center of Biopesticide Engineering & Technology, Northwest A&F University, Yangling, Shaanxi 712100, China.
[3]College of Chemistry, Chemical Engineering and Materials Science, Collaborative Innovation Center of Functionalized Probes for Chemical Imaging in Universities of Shandong, Shandong Normal University, 88 Wenhuadong Road, Jinan 250014, China.
[†]These authors contributed equally to this work.
*e-mails: mxchang@nwsuaf.edu.cn

CONTENTS

1. Supplementary Notes 3

2. Supplementary Methods 3

2.1 Procedures for **L4** synthesis 3

2.2 General procedure for asymmetric reductive amination 4

3. Supplementary Discussion 69

4. Supplementary Figures 71

5. Supplementary References 137

1. Supplementary Notes

All reactions were performed in the nitrogen-filled glovebox or under nitrogen using standard Schlenk techniques unless otherwise noted. Column chromatography was performed using silica gel 60 (200 – 300 mesh). ^{1}H NMR, ^{13}C NMR and ^{31}P NMR spectral data were obtained from Bruker 400/500 MHz spectrometers. Chemical shifts are reported in ppm. Enantiomeric excess values were determined by chiral HPLC on an Agilent 1220 Series instrument or by an Agilent 7890 Series GC to determine the enantiomeric excesses. Key new products were further characterized by HRMS. A positive ion mass spectrum of sample was acquired on a Thermo Scientific LTQ Orbitrap XL mass spectrometer with an electrospray ionization source.

2. Supplementary Methods

2.1 Procedures for L4 synthesis

Chiral ligands **L4**–**L6** were prepared with modified procedures from literatures.[1]
Procedures for the synthesis of (*R*)-**L4** starting from commercially available **S1**:

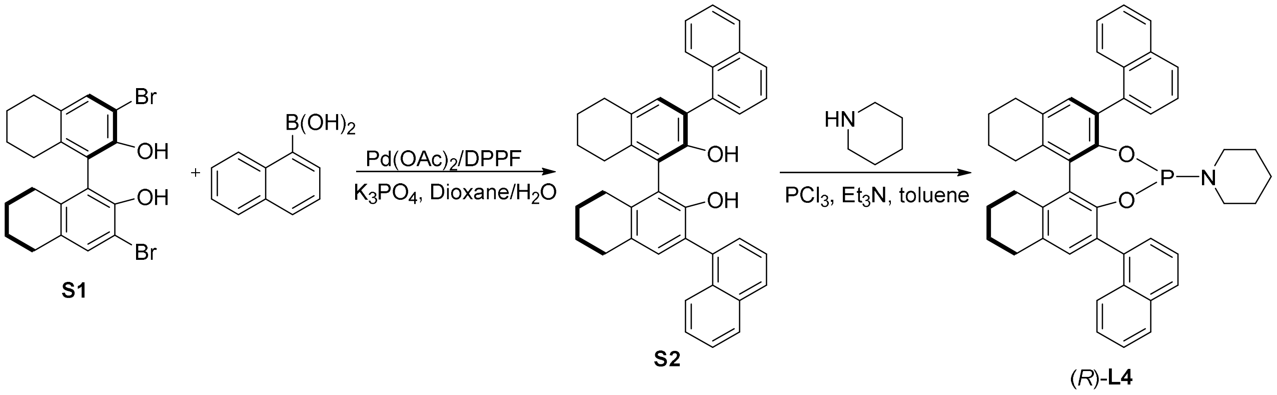

Supplementary Figure 1. Synthetic routes for **L4**.

Step 1:[1a] A 100 mL two-necked flask was charged with (***R***)-**S1** (1.36 g, 3 mmol), 1-naphthyboronic acid (1.23 g, 7.2 mmol, 2.4 equiv.), potassium phosphate trihydrate (3.20 g, 12 mmol, 4.0 equiv.), palladium(II) acetate (20.2 mg, 0.09 mmol, 3 mol%), 1,1'-bis(diphenylphosphino)ferrocene (DPPF) (50.0 mg, 0.09 mmol, 3 mol%) and equipped with a magnetic stirring bar. The apparatus was evacuated and backfilled with argon. Then 1,4-dioxane (40 mL) and distilled water (10 mL) were added via syringe. The apparatus was heated to 110°C with oil bath for 12 h, and the reaction was monitored by TLC. After **S1** was consumed completely, saturated aqueous NH_4Cl (50 mL) was added to the flask and the organic solvent was removed *in vacuo*. The residue was extracted with CH_2Cl_2 (50 mL × 3). The organic layers were combined and washed with brine (50 mL), dried over anhydrous Na_2SO_4 and concentrated to give the crude product, which was purified by column chromatography (petroleum ether / EtOAc = 20 / 1) to give **S2** as white solid (1.34 g, 82% yield).

Step 2:[1b] To a 50 mL Schlenk flask equipped with vacuum/argon stopcock and a magnetic stirring bar, PCl_3 (210 μL, 2.4 mmol, 1.0 equiv.) and toluene (5 mL) were added. The flask was cooled to 0 °C and a solution of piperidine (2.4 mmol, 1.0 equiv.) and Et_3N (2.0 mL, 14.4 mmol, 6.0 equiv.) in toluene (5 mL) was injected to the flask slowly over 5 min. After 5 min the solution was heated with oil bath at 60 °C for 8 h. Then the flask was cooled to 0 °C, and a solution of **S2** (1.31 g, 2.4 mmol, 1.0 equiv.) in 10 mL toluene was injected to the flask over 10 min. After all **S2** was consumed, the reaction mixture was filtered. The filtrate was concentrated *in vacuo* to give the crude product, which was purified by column chromatography (petroleum ether / EtOAc = 40 / 1 with 1% Et_3N) to give **L4** as white solid (0.97 g, 61% yield).

((11bR)-2,6-di(naphthalen-1-yl)-8,9,10,11,12,13,14,15-octahydrodinaphtho[2,1-d:1',2'-f][1,3,2]dioxaphosphepin-4-yl)piperidine(L4): ^{1}H NMR (400 MHz, Chloroform-*d*) δ 7.77 (dq, *J* = 17.2, 7.8 Hz, 6H), 7.43 (dq, *J* = 29.0, 8.2 Hz, 8H), 7.11 (d, *J* = 5.9 Hz, 2H), 2.89 (q, *J* = 5.8, 5.2 Hz, 6H), 2.56 (s, 2H), 2.27 (d, *J* = 6.3 Hz, 3H), 2.03 – 1.63 (m, 8H), 1.17 (d, *J* = 5.9 Hz, 2H), 1.07 – 0.39 (m, 5H). ^{13}C NMR (101 MHz, $CDCl_3$) δ 146.54, 137.79, 137.38, 137.11, 136.68, 133.51, 133.42, 133.26, 132.97, 132.13, 131.60, 131.56, 130.03, 129.97, 127.86, 127.76, 127.49, 127.20, 127.08, 126.99, 125.86, 125.58, 125.37, 125.28, 124.80, 44.06, 43.86, 29.45, 29.40, 29.35, 28.12, 27.98, 26.63, 26.59, 24.83, 23.13, 23.07, 22.97. ^{31}P NMR (162 MHz, Chloroform-*d*) δ 137.20.

2-((11bR)-2,6-di(naphthalen-1-yl)-8,9,10,11,12,13,14,15-octahydrodinaphtho[2,1-d:1',2'-f][1,3,2]dioxaphosphepin-4-yl)-1,2,3,4-tetrahydroisoquinoline(L5): ^{1}H NMR (400 MHz, Chloroform-*d*) δ 7.87 – 7.70 (m, 4H), 7.35 (dddd, *J* = 8.1, 6.7, 3.4, 1.2 Hz, 2H), 7.29 (d, *J* = 8.6 Hz, 1H), 7.23 (d, *J* = 8.4 Hz, 1H), 7.20 – 7.12 (m, 2H), 7.11 – 6.97 (m, 3H), 4.26 (d, *J* = 6.3 Hz, 2H), 3.11 – 2.80 (m, 3H), 2.69 (ddd, *J* = 15.0, 9.4, 6.5 Hz, 1H), 2.42 (s, 6H), 2.12 (d, *J* = 5.7 Hz, 3H), 1.84 (d, *J* = 7.6 Hz, 4H), 1.56 (s, 1H), 1.07 (t, *J* = 7.3 Hz, 6H). ^{13}C NMR (101 MHz, $CDCl_3$) δ 149.24, 149.19, 138.10, 134.72, 134.09, 133.19, 133.09, 131.74, 131.51, 131.22, 130.64, 129.05, 128.53, 128.45, 127.68, 127.64, 127.52, 126.95, 126.93, 125.12, 125.09, 124.67, 124.46, 33.38, 33.26, 30.57, 23.48, 23.45, 22.69, 20.26, 20.22, 14.31, 14.28, 14.23. ^{31}P NMR (162 MHz, Chloroform-*d*) δ 146.68.

(2,6-dimethylbenzyl)-N-methyl-2,6-dipropyldinaphtho[2,1-d:1',2'-f][1,3,2]dioxaphosphepin-4-amine(L6): ^{1}H NMR (400 MHz, Chloroform-*d*) δ 7.95 – 7.49 (m, 6H), 7.48 – 7.07 (m, 7H), 7.07 – 6.77 (m, 5H), 6.77 – 6.30 (m, 2H), 3.48 – 3.06 (m, 2H), 2.81 (dp, *J* = 17.4, 11.0, 9.9 Hz, 7H), 2.62 – 2.21 (m, 3H), 1.83 (dt, *J* = 13.6, 7.0 Hz, 10H). ^{13}C NMR (101 MHz, $CDCl_3$) δ 146.37, 145.27, 137.44, 137.40, 137.29, 136.42, 135.24, 134.61, 134.54, 133.59, 133.45, 133.40, 133.23, 132.14, 132.10, 131.95, 131.72, 130.14, 129.95, 129.10, 127.98, 127.93, 127.76, 127.64, 127.16, 126.63, 125.93, 125.75, 125.64, 125.49, 125.40, 125.31, 125.23, 124.98, 44.97, 44.74, 40.82, 40.64, 29.51, 29.41, 29.35, 28.11, 27.97, 23.17, 23.10, 23.05, 22.98. ^{31}P NMR (162 MHz, $CDCl_3$) δ 138.69.

2.2 General procedure for asymmetric reductive amination

In a nitrogen–filled glovebox, $[Ir(cod)Cl]_2$ (2.0 mg, 3 μmol) and **L4** (3.3 mg, 12.6 μmol) were dissolved in anhydrous trifluoroethanol (2 mL) in a 10 mL vial equipped with a stir bar. The above solution was stirred at room temperature for 20 minutes to *in situ* generate the Ir-**L4** complex. To a 5 mL vial equipped with a stir bar were added ketone (0.3 mmol) and amine (0.29 mmol, 0.95 equiv.) substrates, followed by the addition of anhydrous trifluoroethanol (0.5 mL), Ti(O*i*Pr)$_4$ (0.36 mmol, 1.2 equiv.), $FeCl_3$ (0.09 mmol, 30 mol%), and the solution of the Ir-**L4** complex (50 μL, 0.05 mol%). The total amount of solvent was made to 1.2 mL (CF_3CH_2OH/MeOAc = 1:1). The resulting vial was transferred to an autoclave, which was purged with H_2 3 times and then charged with H_2 (40 atm), and stirred at 40 °C for 24 h. The hydrogen gas was released slowly and the solution was concentrated to give the crude products, which were purified by column chromatography (silica gel, petroleum ether/EtOAc from 10/1 to 5/1 with 0.5% Et_3N) to afford the final product.

(1-**phenyl)ethyl-3-phenylpropan-1-amine (3)**:[3] 98% yield, 98% ee, colorless oil. ^{1}H NMR (500 MHz, $CDCl_3$): δ 7.40 – 7.20 (m, 10H), 3.81 (q, J = 6.6 Hz, 1H, CH), 2.75–2.50 (m, 4H), 1.86 (m, 2H), 1.70 (bs, NH), 1.41 (d, J = 6.7 Hz, 3H, CH_3). $[\alpha]_D^{20}$ =-38.4 (c=1.0, EtOH). Enantiomeric excess was determined by chiral HPLC after the product was converted to the corresponding acetamide: IB-3, Hex/IPA=90:10, 1 mL/min, 220 nm, 8.6 min, 9.3 min.

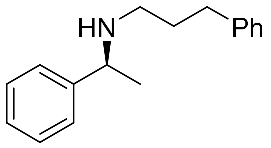

3

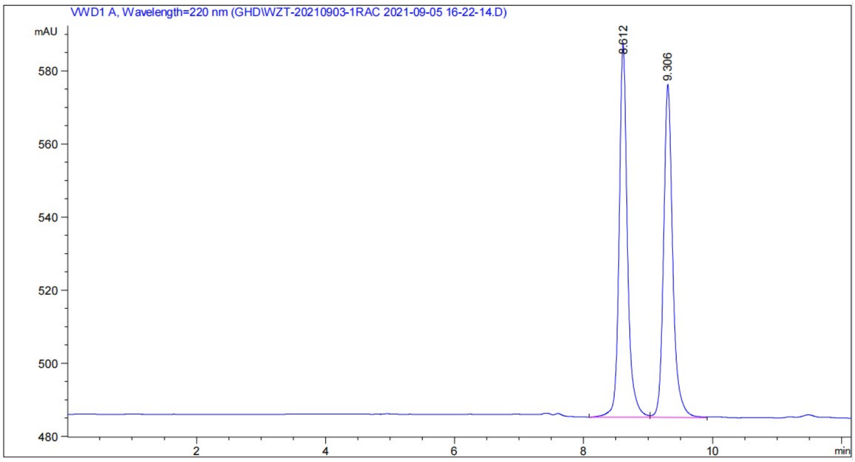

峰 #	保留时间 [min]	类型	峰宽 [min]	峰面积 [mAU*s]	峰高 [mAU]	峰面积 %
1	8.612	BV	0.1335	915.06244	102.16724	50.4486
2	9.306	VV	0.1491	898.78973	91.12868	49.5514

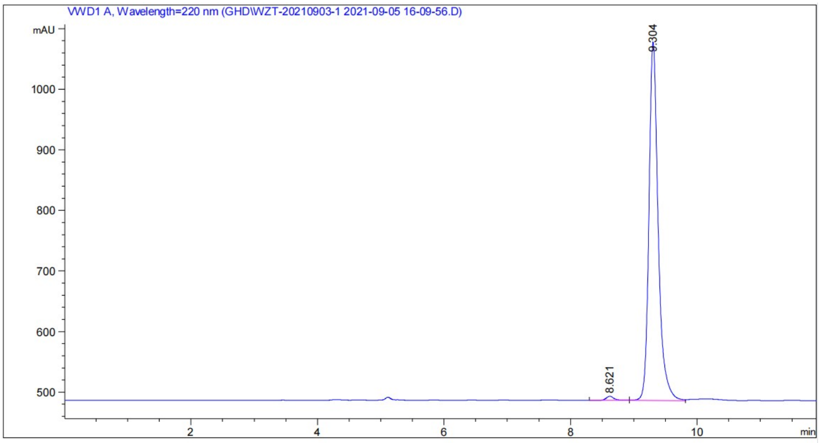

峰 #	保留时间 [min]	类型	峰宽 [min]	峰面积 [mAU*s]	峰高 [mAU]	峰面积 %
1	8.621	BV	0.1450	69.14479	7.01226	1.1374
2	9.304	VV	0.1525	6010.28076	591.56018	98.8626

Supplementary Figure 2. HPLC spectra for racemic and chiral **3**.

N-(1-(4-fluorophenyl)ethyl)-3-phenylpropan-1-amine (4): 95% yield, 96% ee, brown oil. ^{1}H NMR (500 MHz, Chloroform-*d*) δ 7.31 (ddd, *J* = 7.8, 5.0, 2.0 Hz, 4H), 7.27 – 7.16 (m, 3H), 7.05 (t, *J* = 8.7 Hz, 2H), 3.79 (q, *J* = 6.6 Hz, 1H), 2.75 – 2.44 (m, 4H), 1.90 – 1.78 (m, 2H), 1.56 – 1.41 (m, 1H), 1.37 (d, *J* = 6.6 Hz, 3H). ^{13}C NMR (126 MHz, Chloroform-*d*) δ 142.1, 128.37, 128.3, 128.0, 128.0, 125.7, 115.2, 115.0, 57.7, 47.3, 33.6, 31.8, 24.4. $[\alpha]_D^{20}$ =-42.1 (c=1.0, EtOH). Enantiomeric excess was determined by chiral HPLC after the product was converted to the corresponding acetamide: Chiralpak AD-H column, Hex/IPA = 90:10, 1 mL/min, 220 nm, 7.4 min, 7.8 min.

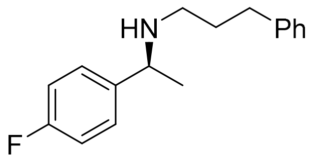

4

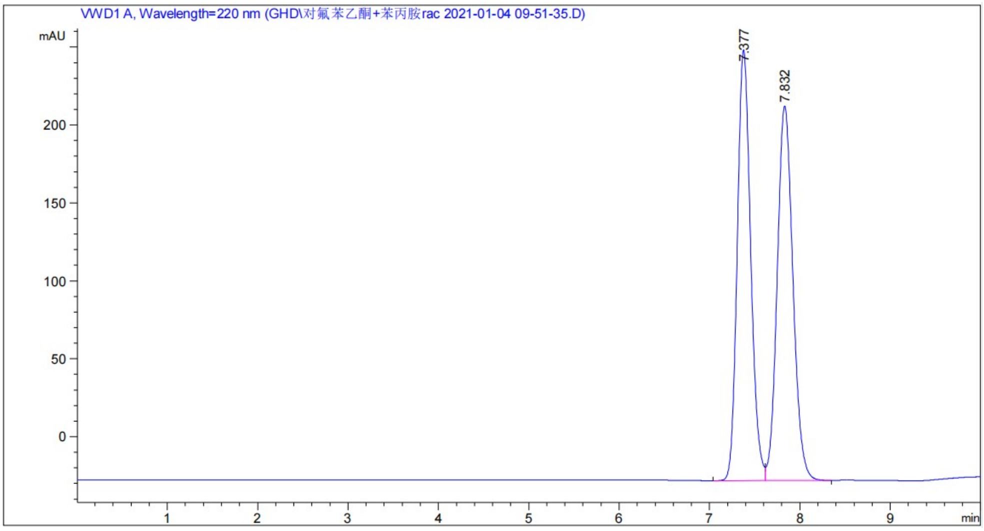

峰 #	保留时间 [min]	类型	峰宽 [min]	峰面积 [mAU*s]	峰高 [mAU]	峰面积 %
1	7.377	BV	0.1592	2848.80444	276.40857	49.5193
2	7.832	VV	0.1870	2904.11206	240.29732	50.4807

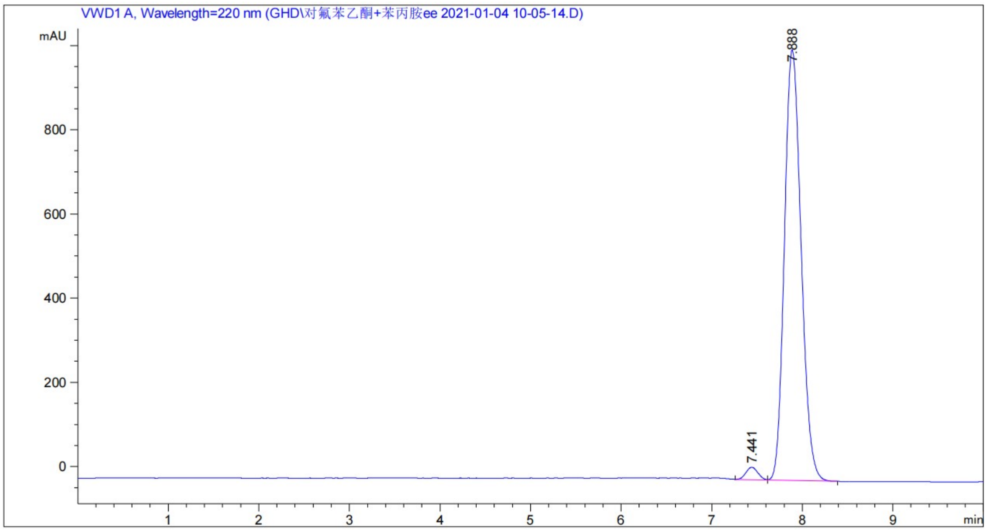

峰 #	保留时间 [min]	类型	峰宽 [min]	峰面积 [mAU*s]	峰高 [mAU]	峰面积 %
1	7.441	BV	0.1450	270.65869	29.52475	2.0307
2	7.888	VB	0.2010	1.30578e4	1022.46716	97.9693

Supplementary Figure 3. HPLC spectra for racemic and chiral **4**.

N-(1-(4-chlorophenyl)ethyl)-3-phenylpropan-1-amine (5):[4] 95% yield, 97% ee, brown oil. ^{1}H NMR (400 MHz, Chloroform-*d*) δ 7.33 – 7.19 (m, 6H), 7.19 – 7.08 (m, 3H), 3.71 (q, *J* = 6.6 Hz, 1H), 2.57 (ddt, *J* = 29.9, 18.3, 6.8 Hz, 3H), 2.48 – 2.38 (m, 1H), 1.77 (p, *J* = 7.3 Hz, 3H), 1.30 (d, *J* = 6.6 Hz, 3H). ^{13}C NMR (126 MHz, Chloroform-*d*) δ 144.4, 142.1, 128.5, 128.3, 128.3, 128.0, 125.7, 57.7, 47.3, 33.6, 31.8, 24.4. $[\alpha]_D^{20}$ =-44.2 (c=1.0, EtOH). Enantiomeric excess was determined by chiral HPLC after the product was converted to the corresponding acetamide: Chiralpak IB-3 column, Hex/IPA=95:5, 1 mL/min, 220 nm, 35.1 min, 36.9 min.

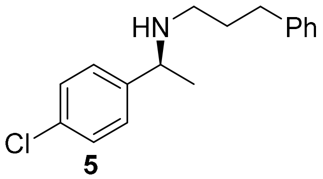

5

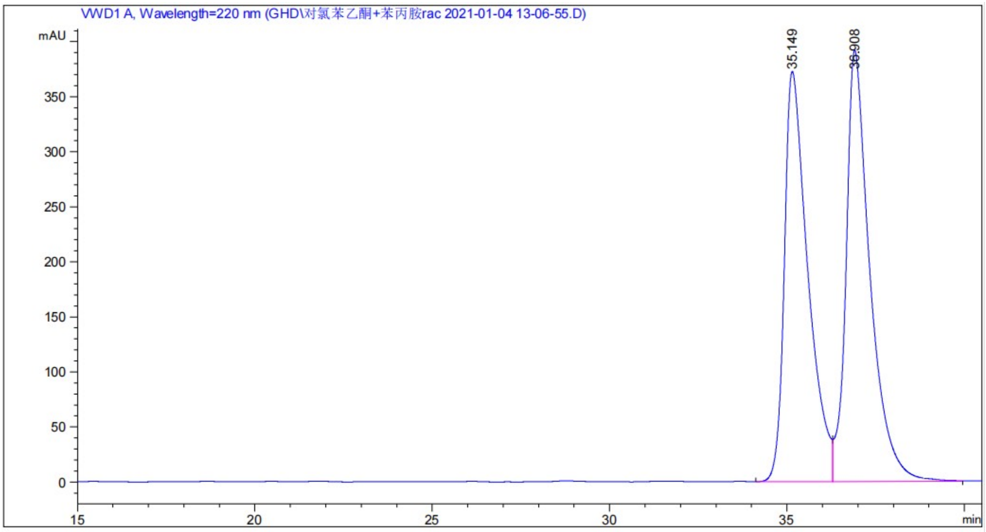

峰 #	保留时间 [min]	类型	峰宽 [min]	峰面积 [mAU*s]	峰高 [mAU]	峰面积 %
1	35.149	BV	0.6746	1.71996e4	372.56650	48.2747
2	36.908	VV	0.6807	1.84290e4	391.84341	51.7253

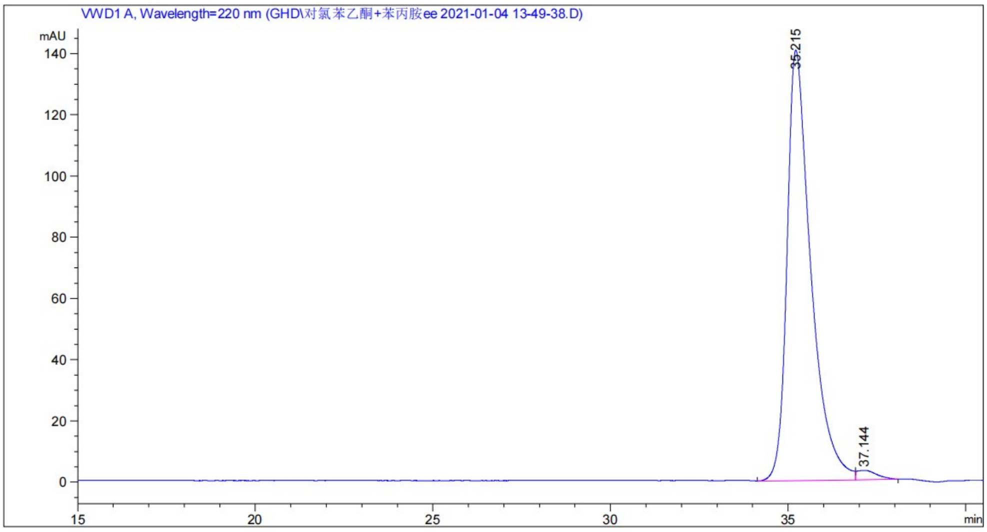

峰 #	保留时间 [min]	类型	峰宽 [min]	峰面积 [mAU*s]	峰高 [mAU]	峰面积 %
1	35.215	BV	0.6810	6567.97852	140.60835	98.2416
2	37.144	VB	0.5622	117.56129	3.03318	1.7584

Supplementary Figure 4. HPLC spectra for racemic and chiral **5**.

N-(1-(4-bromophenyl)ethyl)-3-phenylpropan-1-amine (6): 98% yield, 97% ee, brown oil. ^{1}H NMR (500 MHz, Chloroform-*d*) δ 7.56 – 7.39 (m, 2H), 7.42 – 7.13 (m, 7H), 3.76 (q, *J* = 6.5 Hz, 1H), 2.77 – 2.44 (m, 4H), 1.91 – 1.77 (m, 2H), 1.45 (s, 1H), 1.36 (d, *J* = 6.6 Hz, 3H). ^{13}C NMR (126 MHz, Chloroform-*d*) δ 144.9, 142.1, 131.5, 128.4, 128.3, 128.3, 125.8, 120.4, 57.9, 47.3, 33.6, 31.8, 24.4. $[\alpha]_D^{20}$ =+43.9 (c=1.0, EtOH). Enantiomeric excess was determined by chiral HPLC after the product was converted to the corresponding acetamide: Chiralpak AD-H column, Hex/IPA = 95:5, 1 mL/min, 220 nm, 15.0 min, 16.0 min.

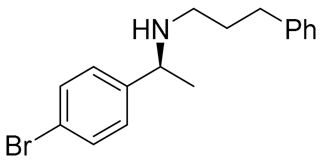

6

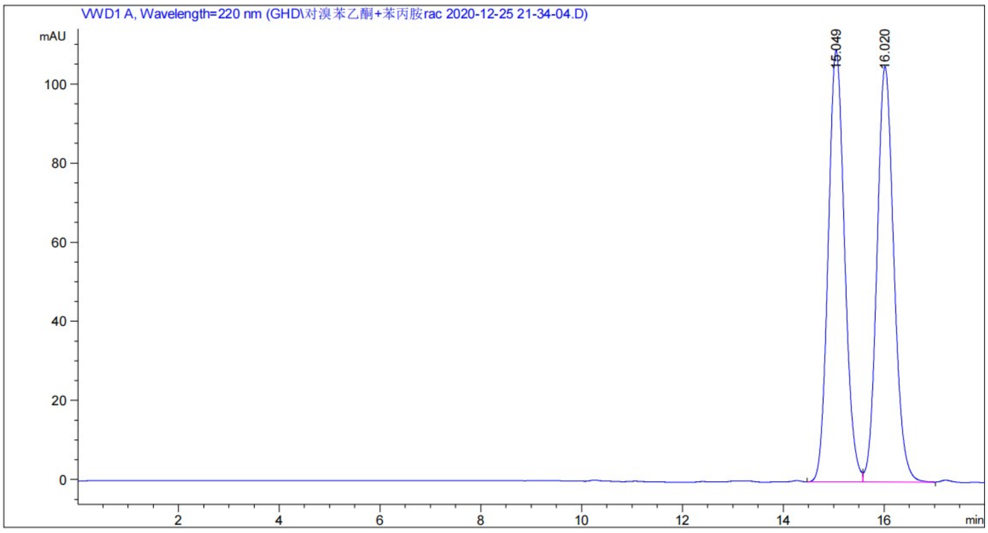

峰 #	保留时间 [min]	类型	峰宽 [min]	峰面积 [mAU*s]	峰高 [mAU]	峰面积 %
1	15.049	BV	0.3498	2467.79175	109.14702	50.2078
2	16.020	VB	0.3597	2447.36401	105.07175	49.7922

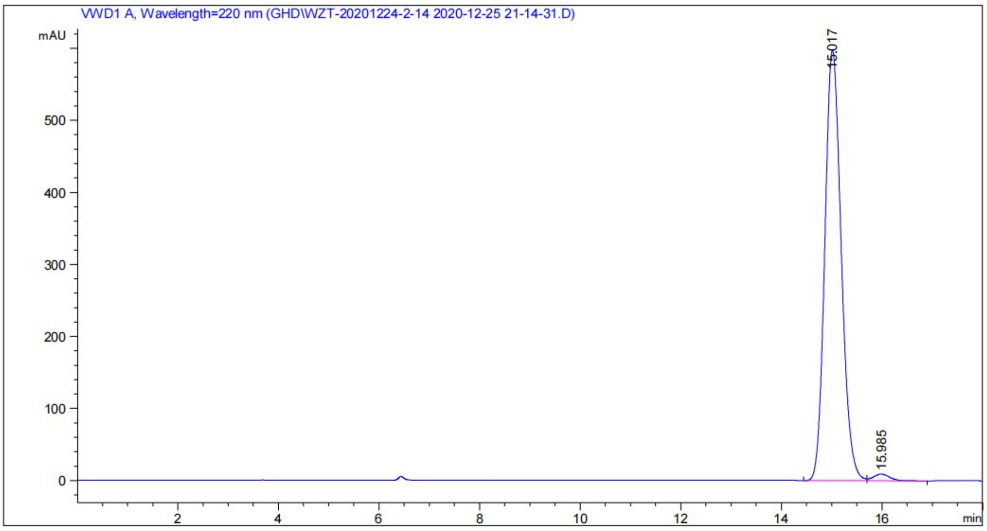

峰 #	保留时间 [min]	类型	峰宽 [min]	峰面积 [mAU*s]	峰高 [mAU]	峰面积 %
1	15.017	BV	0.3514	1.34992e4	597.82922	98.4340
2	15.985	VB	0.3496	214.76472	9.36328	1.5660

Supplementary Figure 5. HPLC spectra for racemic and chiral **6**.

3-phenyl-N-(1-(4-(trifluoromethyl)phenyl)ethyl)propan-1-amine (7): 95% yield, 98% ee, brown oil. ^{1}H NMR (400 MHz, Chloroform-*d*) δ 7.57 (d, *J* = 7.9 Hz, 2H), 7.42 (d, *J* = 7.9 Hz, 2H), 7.25 (t, *J* = 7.4 Hz, 2H), 7.20 – 7.05 (m, 3H), 3.80 (q, *J* = 6.6 Hz, 1H), 2.59 (ddq, *J* = 28.3, 13.6, 6.7 Hz, 3H), 2.48 – 2.34 (m, 1H), 1.78 (p, *J* = 7.4 Hz, 2H), 1.42 (s, 1H), 1.33 (d, *J* = 6.6 Hz, 3H). $[\alpha]_D^{20}$ =+38.2 (c=1.0, EtOH). Enantiomeric excess was determined by chiral HPLC after the product was converted to the corresponding acetamide: Chiralpak IB-3 column, Hex/IPA = 95:5, 1 mL/min, 220 nm, 19.2 min, 20.7 min.

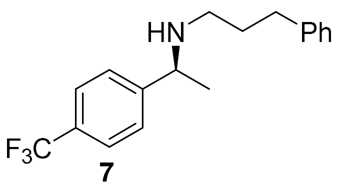

7

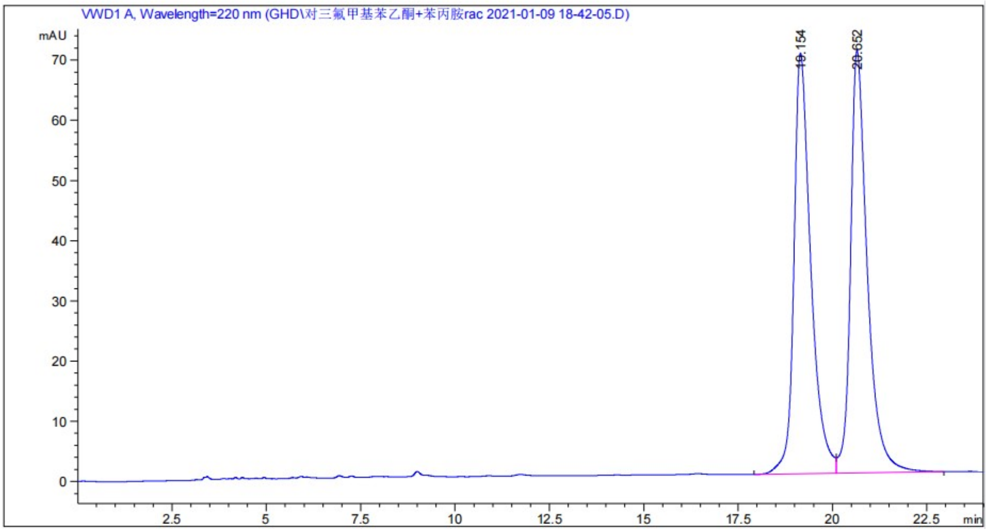

峰 #	保留时间 [min]	类型	峰宽 [min]	峰面积 [mAU*s]	峰高 [mAU]	峰面积 %
1	19.154	BV	0.4622	2182.84717	69.92355	50.1555
2	20.652	VB	0.4544	2169.31519	70.21412	49.8445

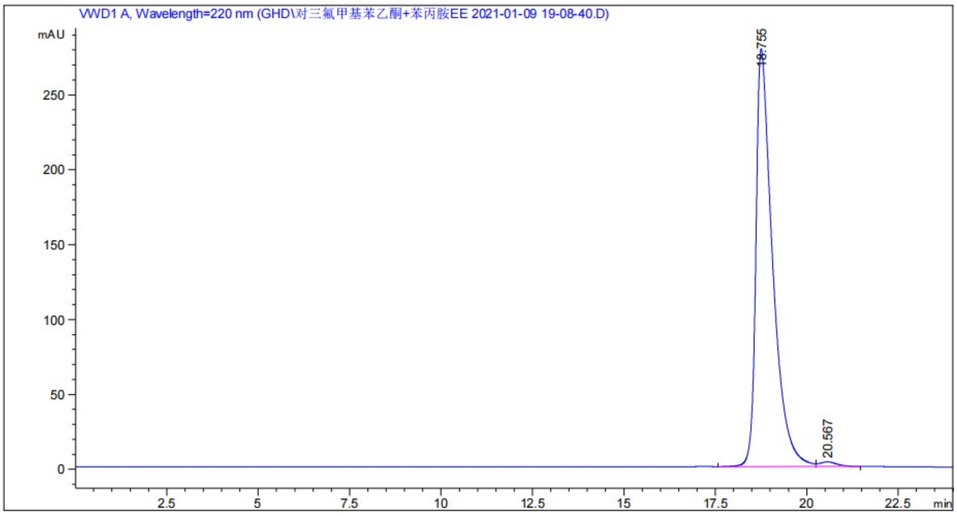

峰 #	保留时间 [min]	类型	峰宽 [min]	峰面积 [mAU*s]	峰高 [mAU]	峰面积 %
1	18.755	BV	0.4617	8842.16797	279.02475	98.8405
2	20.567	VB	0.4918	103.73003	3.13717	1.1595

Supplementary Figure 6. HPLC spectra for racemic and chiral **7**.

3-phenyl-N-(1-(p-tolyl)ethyl)propan-1-amine (8): 95% yield, 97% ee, brown oil. ^{1}H NMR (500 MHz, Chloroform-*d*) δ 7.37 – 7.28 (m, 2H), 7.28 – 7.14 (m, 7H), 3.78 (q, *J* = 6.6 Hz, 1H), 2.75 – 2.46 (m, 4H), 2.41 (s, 3H), 1.92 – 1.77 (m, 2H), 1.59 (s, 1H), 1.40 (d, *J* = 6.6 Hz, 3H). ^{13}C NMR (126 MHz, Chloroform-*d*) δ 142.8, 142.2, 136.4, 129.1, 128.4, 128.3, 126.5, 125.7, 58.0, 47.4, 33.7, 31.9, 24.3, 21.1. $[\alpha]_D^{20}$ =-46.9 (c=1.0, EtOH). Enantiomeric excess was determined by chiral HPLC after the product was converted to the corresponding acetamide: Chiralpak IB-3 column, Hex/IPA=90:10, 1 mL/min, 220 nm, 11.1 min, 11.9 min.

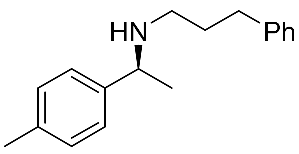

8

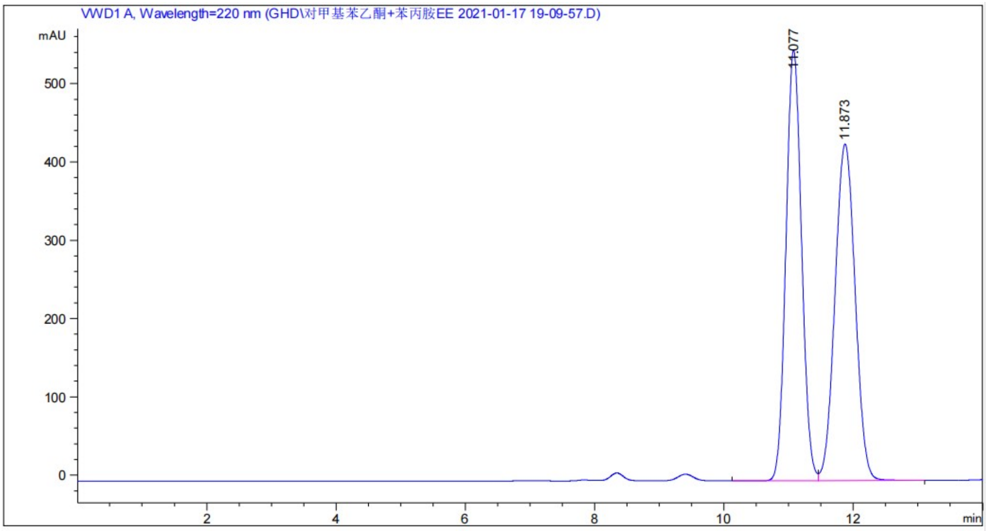

峰 #	保留时间 [min]	类型	峰宽 [min]	峰面积 [mAU*s]	峰高 [mAU]	峰面积 %
1	11.077	BV	0.2617	9220.04492	549.76593	49.7748
2	11.873	VB	0.3426	9303.48340	429.61957	50.2252

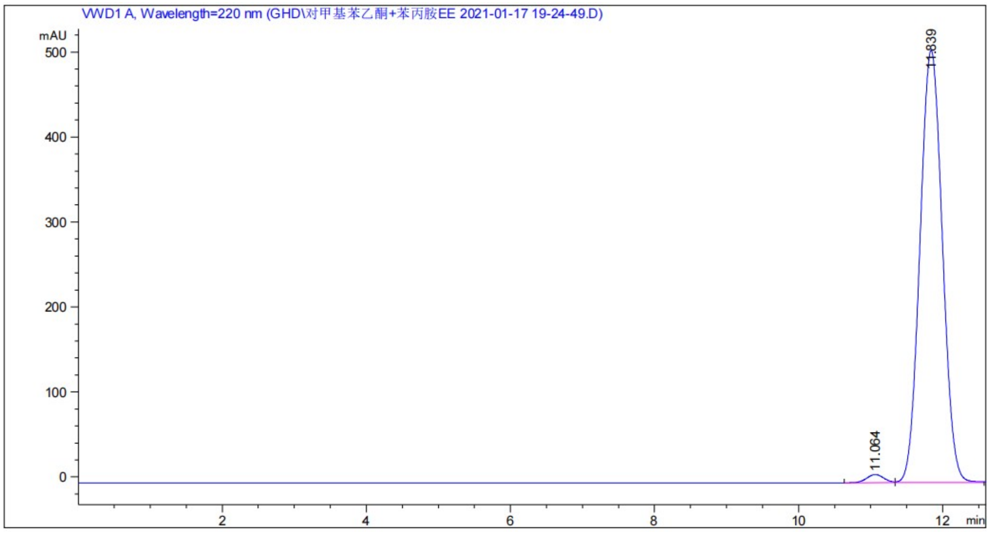

峰 #	保留时间 [min]	类型	峰宽 [min]	峰面积 [mAU*s]	峰高 [mAU]	峰面积 %
1	11.064	BV	0.2588	159.06155	9.62730	1.4304
2	11.839	VV	0.3414	1.09612e4	508.73810	98.5696

Supplementary Figure 7. HPLC spectra for racemic and chiral **8**.

N-(1-(4-methoxyphenyl)ethyl)-3-phenylpropan-1-amine(9): 96% yield, 98% ee, brown oil. ^{1}H NMR (400 MHz, Chloroform-*d*) δ 7.24 (q, *J* = 8.7, 8.1 Hz, 4H), 7.19 – 7.10 (m, 3H), 6.86 (d, *J* = 8.4 Hz, 2H), 3.79 (s, 3H), 3.70 (d, *J* = 6.6 Hz, 1H), 2.68 – 2.40 (m, 4H), 1.98 (s, 1H), 1.78 (td, *J* = 7.7, 2.9 Hz, 2H), 1.32 (d, *J* = 6.6 Hz, 3H). ^{13}C NMR (126 MHz, Chloroform-*d*) δ 137.8, 128.3, 128.3, 127.5, 125.7, 113.7, 57.6, 55.2, 47.3, 33.7, 31.8, 24.2. $[\alpha]_D^{20}$ =-38.4 (c=1.0, EtOH). Enantiomeric excess was determined by chiral HPLC after the product was converted to the corresponding acetamide: Chiralpak AD-H column, Hex/IPA=97:3, 1 mL/min, 220 nm, 7.2 min, 7.8 min.

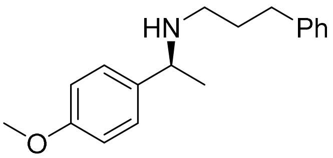

9

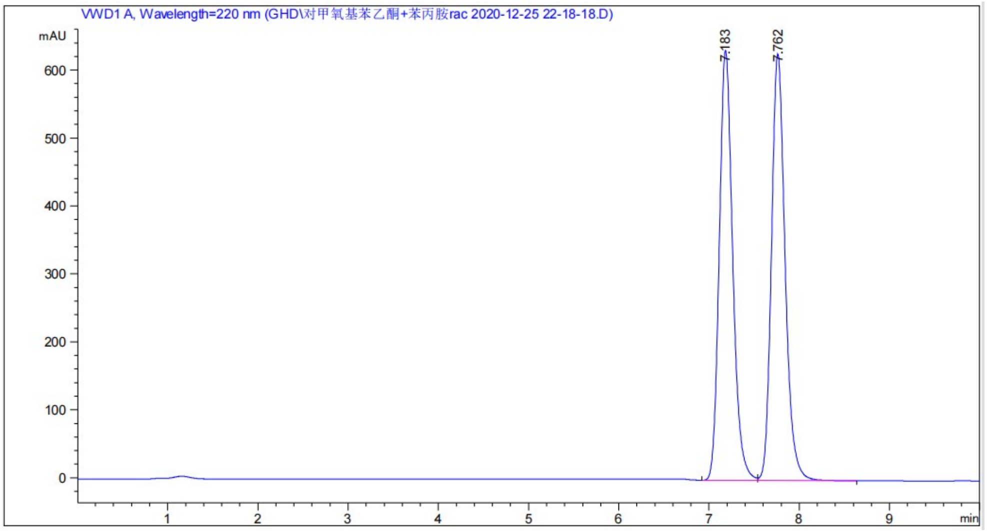

峰 #	保留时间 [min]	类型	峰宽 [min]	峰面积 [mAU*s]	峰高 [mAU]	峰面积 %
1	7.183	BV	0.1586	6535.38770	632.46198	49.7717
2	7.762	VB	0.1606	6595.34375	627.54730	50.2283

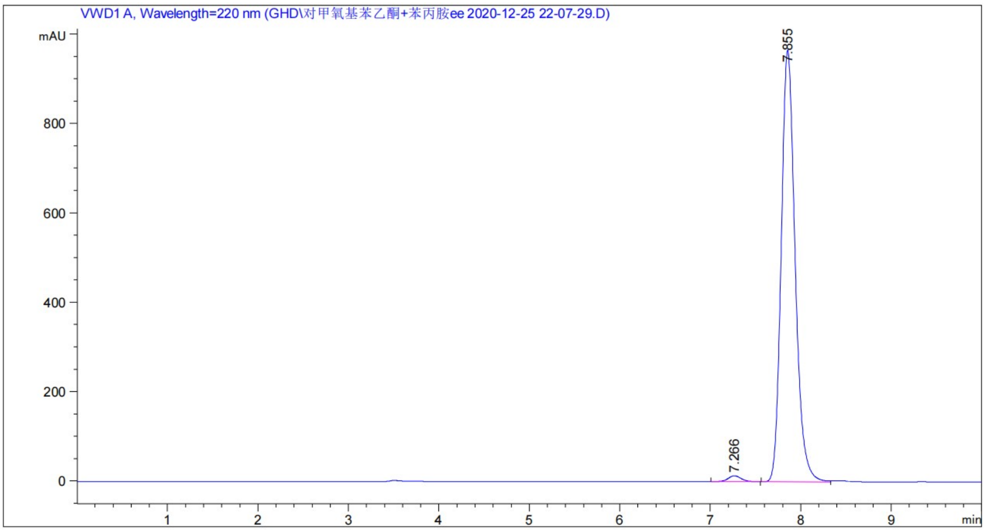

峰 #	保留时间 [min]	类型	峰宽 [min]	峰面积 [mAU*s]	峰高 [mAU]	峰面积 %
1	7.266	BB	0.1540	130.42784	13.11518	1.2499
2	7.855	BV	0.1645	1.03045e4	965.66522	98.7501

Supplementary Figure 8. HPLC spectra for racemic and chiral **9**.

N-(1-(4-cyclohexylphenyl)ethyl)-3-phenylpropan-1-amine(10): 94% yield, 96% ee, brown oil. ^{1}H NMR (500 MHz, Chloroform-*d*) δ 7.39 – 7.16 (m, 9H), 3.80 (q, J = 6.6 Hz, 1H), 2.77 – 2.50 (m, 5H), 2.05 – 1.77 (m, 8H), 1.57 – 1.26 (m, 10H). ^{13}C NMR (126 MHz, Chloroform-*d*) δ 146.6, 143.1, 142.3, 128.4, 128.3, 126.8, 126.4, 125.7, 58.0, 47.4, 44.2, 34.5, 33.7, 31.9, 27.0, 26.2, 24.2. $[\alpha]_D^{20}$ =-41.9 (c=1.0, EtOH). Enantiomeric excess was determined by chiral HPLC after the product was converted to the corresponding acetamide: Chiralpak IB-3 column, Hex/IPA=95:5, 1 mL/min, 220 nm, 12.9 min, 14.1 min.

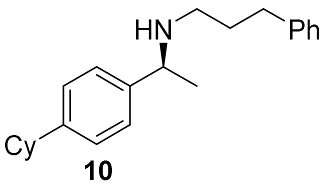

10

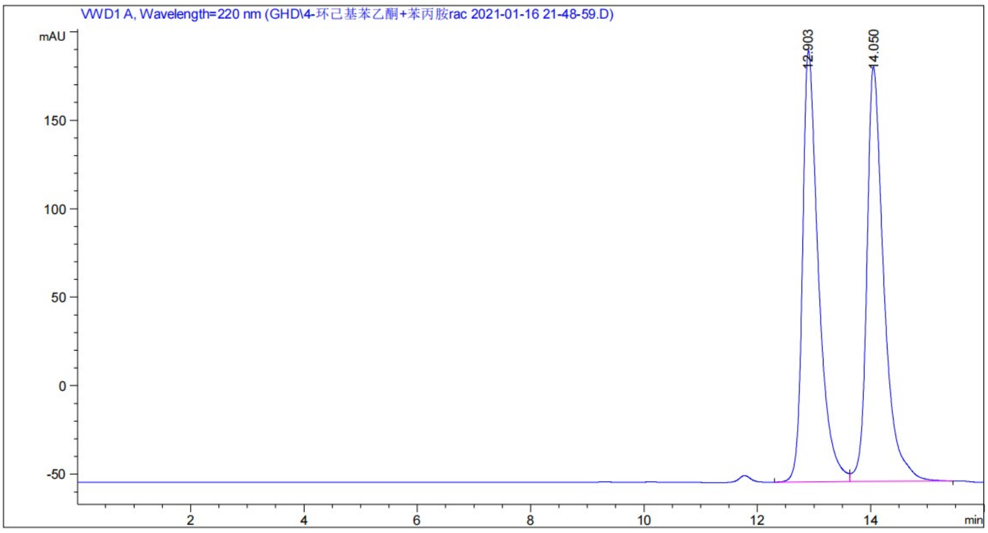

峰 #	保留时间 [min]	类型	峰宽 [min]	峰面积 [mAU*s]	峰高 [mAU]	峰面积 %
1	12.903	BV	0.2881	4764.91748	243.74258	49.3381
2	14.050	VB	0.3097	4892.76563	234.24722	50.6619

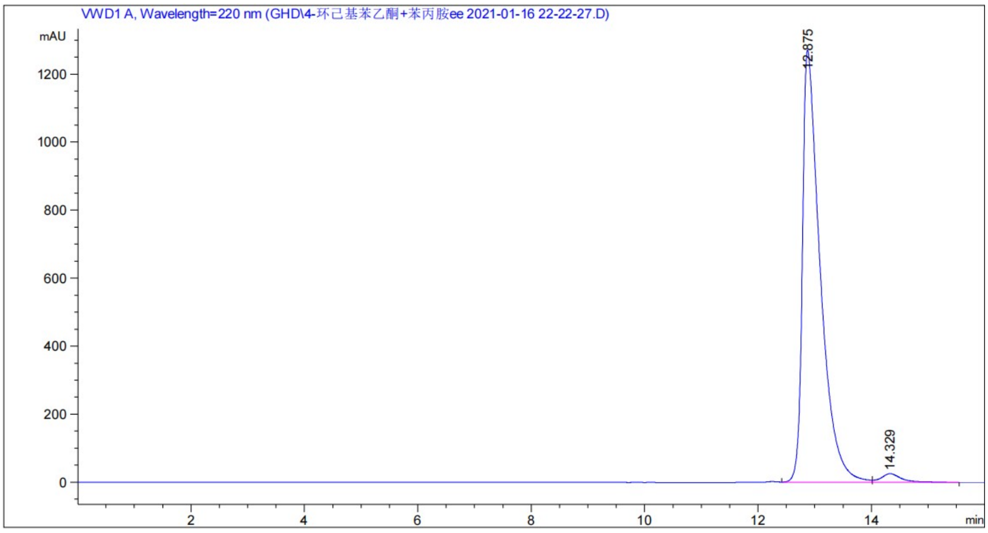

峰 #	保留时间 [min]	类型	峰宽 [min]	峰面积 [mAU*s]	峰高 [mAU]	峰面积 %
1	12.875	VV	0.3202	2.76884e4	1270.83813	97.7114
2	14.329	VV	0.3673	648.51410	25.62067	2.2886

Supplementary Figure 9. HPLC spectra for racemic and chiral **10**.

(1-([1,1'-biphenyl]-4-yl)ethyl)-N-methyl-3-phenylpropan-1-amine(11): 96% yield, 98% ee, brown oil. ^{1}H NMR (500 MHz, Chloroform-*d*) δ 7.70 – 7.58 (m, 4H), 7.53 – 7.36 (m, 5H), 7.36 – 7.27 (m, 2H), 7.27 – 7.18 (m, 3H), 3.86 (q, J = 6.6 Hz, 1H), 2.78 – 2.53 (m, 4H), 1.87 (pd, J = 7.1, 2.6 Hz, 2H), 1.51 (s, 1H), 1.45 (d, J = 6.6 Hz, 3H). ^{13}C NMR (126 MHz, Chloroform-*d*) δ 145.0, 142.2, 128.8, 128.4, 128.3, 127.2, 127.1, 127.1, 127.0, 125.8, 58.1, 47.4, 33.7, 31.9, 24.3. $[\alpha]_D^{20}$ =-49.1 (c=1.0, EtOH). Enantiomeric excess was determined by chiral HPLC after the product was converted to the corresponding acetamide: Chiralpak AD-H column, Hex/IPA = 90:10, 1 mL/min, 220 nm, 20.3 min, 21.6 min.

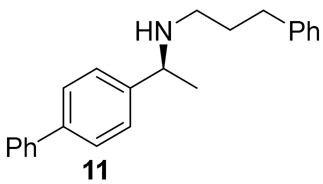

11

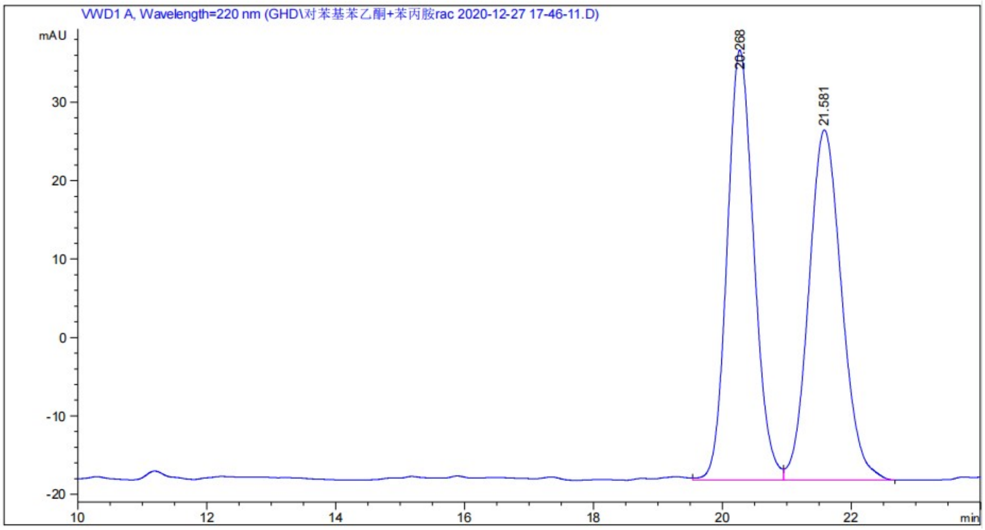

峰 #	保留时间 [min]	类型	峰宽 [min]	峰面积 [mAU*s]	峰高 [mAU]	峰面积 %
1	20.268	VV	0.4459	1579.03357	54.84510	50.0242
2	21.581	VB	0.5500	1577.50330	44.66727	49.9758

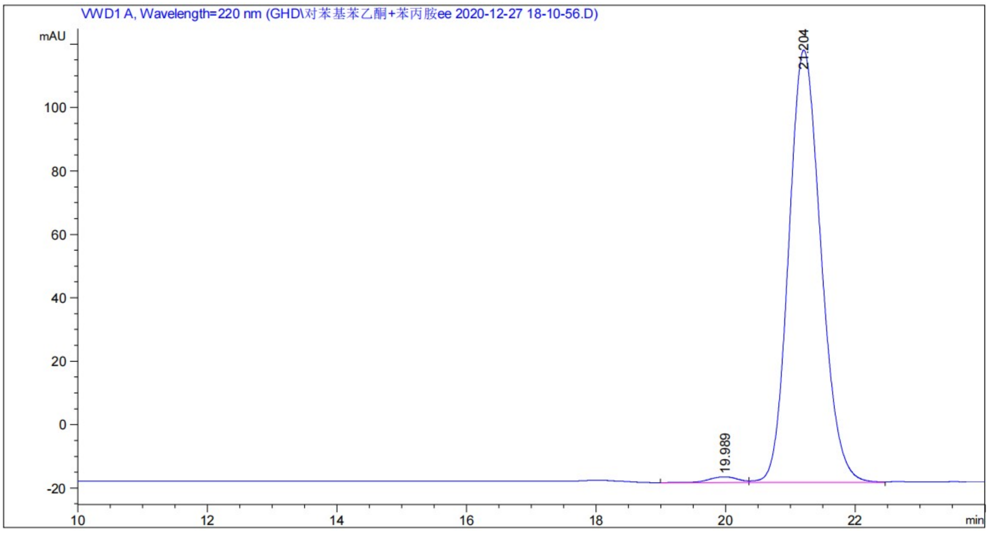

峰 #	保留时间 [min]	类型	峰宽 [min]	峰面积 [mAU*s]	峰高 [mAU]	峰面积 %
1	19.989	BV	0.4918	56.70331	1.77907	1.1919
2	21.204	VV	0.5352	4700.83447	136.36661	98.8081

Supplementary Figure 10. HPLC spectra for racemic and chiral **11**.

N-(1-(3-fluorophenyl)ethyl)-3-phenylpropan-1-amine(12): 96% yield, 98% ee, brown oil. ^{1}H NMR (500 MHz, Chloroform-*d*) δ 7.33 (tt, *J* = 8.1, 3.3 Hz, 3H), 7.27 – 7.18 (m, 3H), 7.16 – 7.08 (m, 2H), 6.98 (td, *J* = 8.5, 2.6 Hz, 1H), 3.80 (q, *J* = 6.6 Hz, 1H), 2.78 – 2.48 (m, 4H), 1.92 – 1.79 (m, 2H), 1.55 – 1.46 (m, 1H), 1.39 (d, *J* = 6.6 Hz, 3H). ^{13}C NMR (126 MHz, Chloroform-*d*) δ 164.14, 162.1, 148.8, 142.1, 129.8, 128.3, 125.7, 122.2, 58.0, 47.3, 33.6, 31.8, 24.3. $[\alpha]_D^{20}$ =+45.2 (c=0.5, EtOH). Enantiomeric excess was determined by chiral HPLC after the product was converted to the corresponding acetamide: Chiralpak IB-3 column, Hex/IPA = 95:5, 1 mL/min, 220 nm, 15.5 min, 18.3 min.

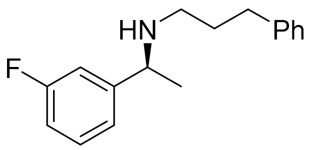

12

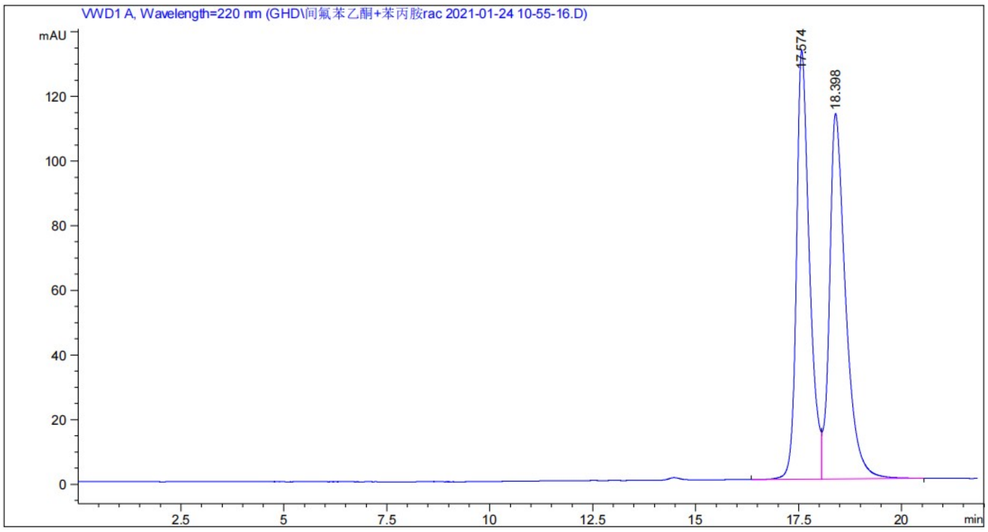

峰 #	保留时间 [min]	类型	峰宽 [min]	峰面积 [mAU*s]	峰高 [mAU]	峰面积 %
1	17.574	BV	0.3282	2925.85498	132.72664	48.6974
2	18.398	VB	0.4031	3082.38062	113.06351	51.3026

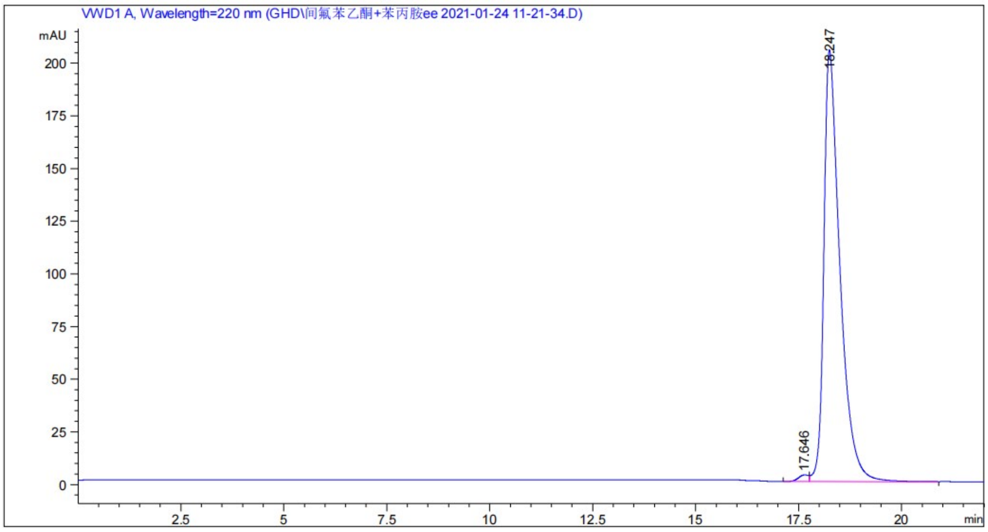

峰 #	保留时间 [min]	类型	峰宽 [min]	峰面积 [mAU*s]	峰高 [mAU]	峰面积 %
1	17.646	BV	0.2567	53.12796	3.13444	0.9449
2	18.247	VB	0.3992	5569.48730	204.82466	99.0551

Supplementary Figure 11. HPLC spectra for racemic and chiral **12**.

N-(1-(3-chlorophenyl)ethyl)-3-phenylpropan-1-amine(13):[5] 97% yield, 98% ee, brown oil. ^{1}H NMR (500 MHz, Chloroform-*d*) δ 7.41 – 7.14 (m, 9H), 3.77 (q, J = 6.6 Hz, 1H), 2.76 – 2.46 (m, 4H), 1.84 (p, J = 7.3 Hz, 2H), 1.46 (s, 1H), 1.37 (d, J = 6.6 Hz, 3H). ^{13}C NMR (126 MHz, Chloroform-*d*) δ 148.1, 142.1, 134.3, 129.7, 128.3, 128.3, 127.0, 126.7, 125.7, 124.8, 58.0, 47.3, 33.6, 31.8, 24.3. $[\alpha]_D^{20}$ =-40.4 (c=0.5, EtOH). Enantiomeric excess was determined by chiral HPLC after the product was converted to the corresponding acetamide: Chiralpak IB-3 column, Hex/IPA=90:10, 1 mL/min, 220nm, 11.4 min, 12.7 min.

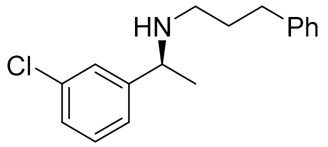

13

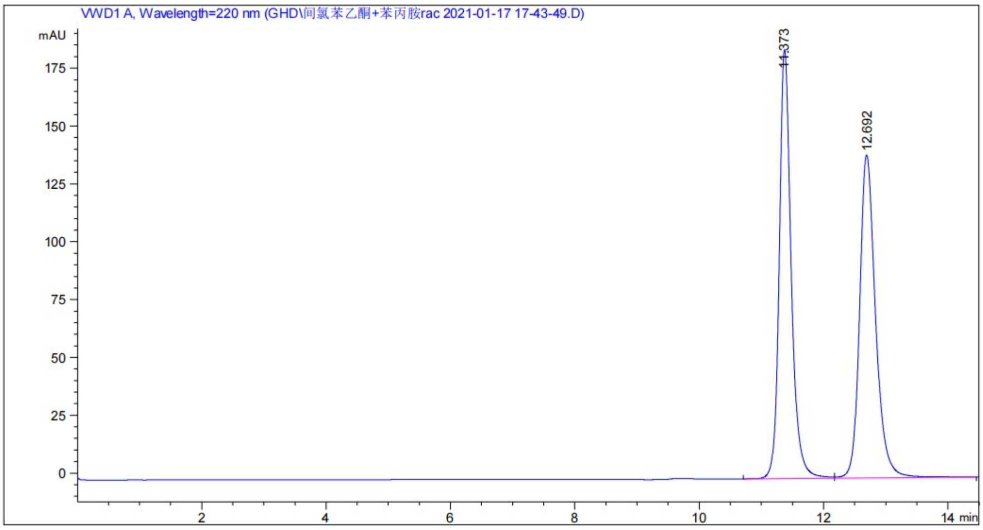

峰 #	保留时间 [min]	类型	峰宽 [min]	峰面积 [mAU*s]	峰高 [mAU]	峰面积 %
1	11.373	BV	0.2000	2462.65527	185.30247	49.7900
2	12.692	VB	0.2697	2483.43140	139.52892	50.2100

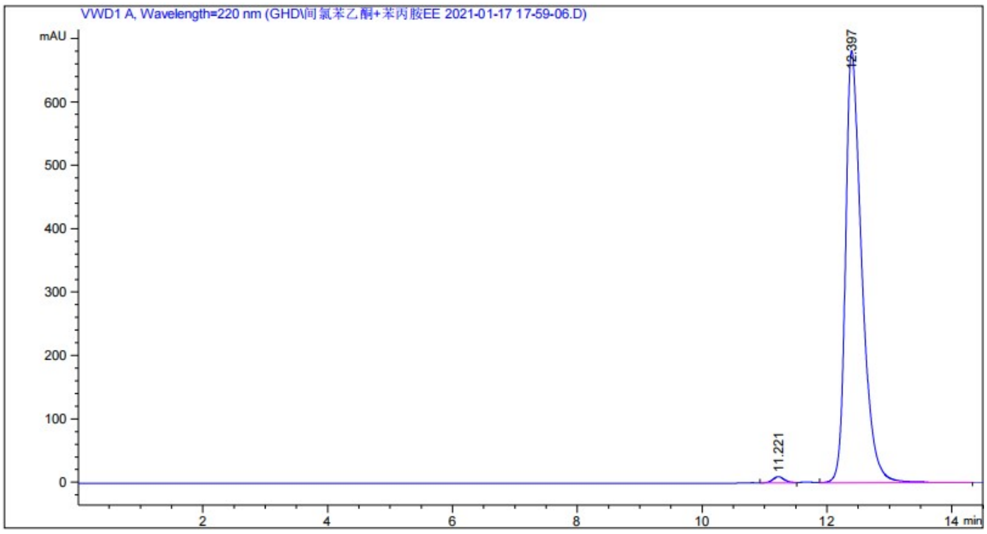

峰 #	保留时间 [min]	类型	峰宽 [min]	峰面积 [mAU*s]	峰高 [mAU]	峰面积 %
1	11.221	VV	0.2015	136.82048	10.19909	1.0937
2	12.397	VV	0.2719	1.23726e4	681.11426	98.9063

Supplementary Figure 12. HPLC spectra for racemic and chiral **13**.

N-(1-(3-bromophenyl)ethyl)-3-phenylpropan-1-amine(14): 96% yield, 98% ee, colorless oil. ^{1}H NMR (500 MHz, Chloroform-*d*) δ 7.52 (t, *J* = 1.9 Hz, 1H), 7.41 (dt, *J* = 7.7, 1.7 Hz, 1H), 7.35 – 7.16 (m, 7H), 3.76 (q, *J* = 6.6 Hz, 1H), 2.75 – 2.46 (m, 4H), 1.83 (p, *J* = 7.4 Hz, 2H), 1.44 (s, 1H), 1.37 (d, *J* = 6.6 Hz, 3H). ^{13}C NMR (126 MHz, Chloroform-*d*) δ 148.4, 142.1, 130.0, 129.9, 129.7, 128.3, 128.3, 125.7, 125.3, 122.6, 58.0, 47.3, 33.6, 31.8, 24.4. $[\alpha]_D^{20}$ =+46.2 (c=1.0, EtOH). Enantiomeric excess was determined by chiral HPLC after the product was converted to the corresponding acetamide: IB-3, Hex/IPA = 90:10, 1 mL/min, 220 nm, 12.3 min, 13.9 min.

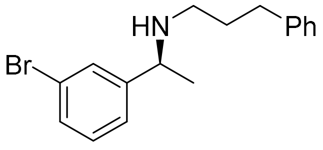

14

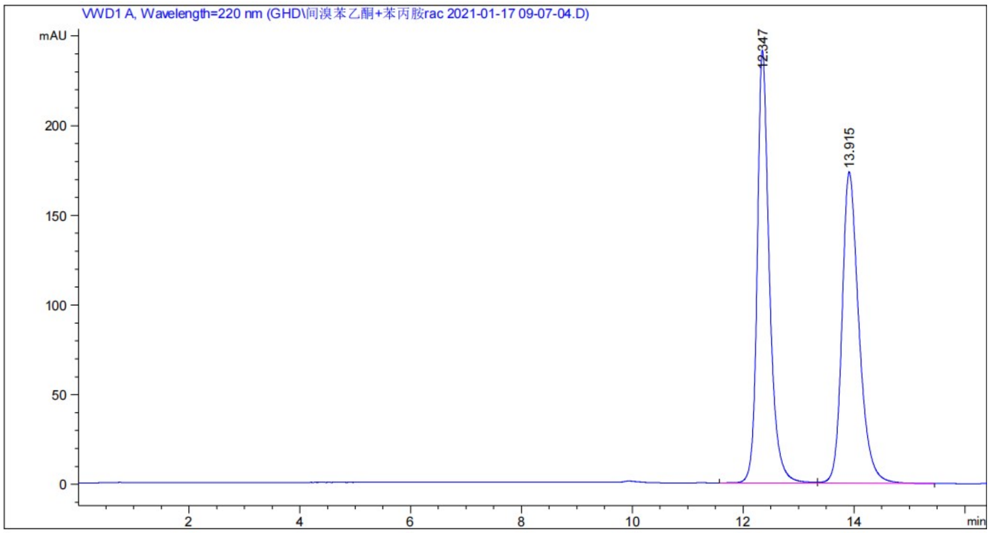

峰 #	保留时间 [min]	类型	峰宽 [min]	峰面积 [mAU*s]	峰高 [mAU]	峰面积 %
1	12.347	VV	0.2284	3690.45361	241.31906	50.0800
2	13.915	VB	0.3194	3678.66821	173.50508	49.9200

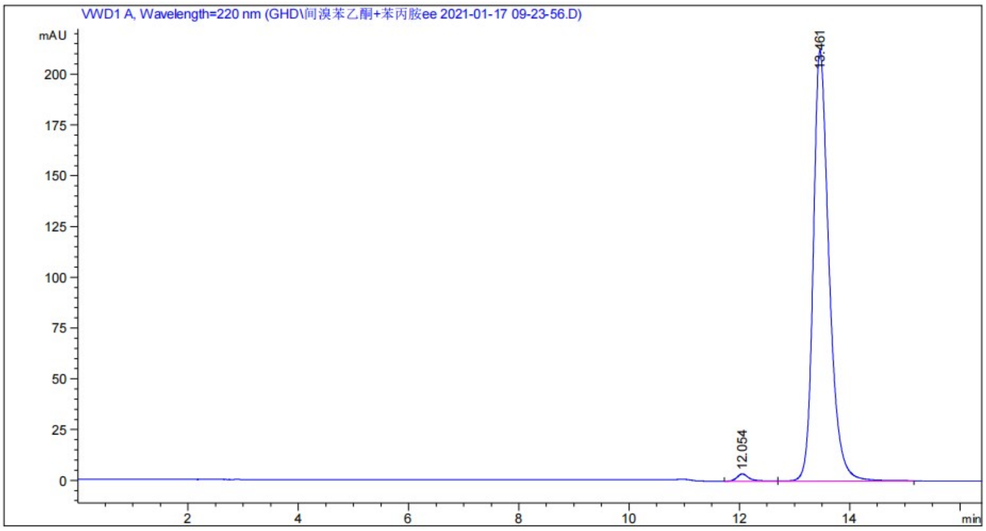

峰 #	保留时间 [min]	类型	峰宽 [min]	峰面积 [mAU*s]	峰高 [mAU]	峰面积 %
1	12.054	BV	0.2260	54.46158	3.58950	1.2717
2	13.461	VV	0.3005	4228.13135	212.16360	98.7283

Supplementary Figure 13. HPLC spectra for racemic and chiral **14**.

3-phenyl-N-(1-(3-(trifluoromethyl)phenyl)ethyl)propan-1-amine(15): 95% yield, 98% ee, colorless oil. ^{1}H NMR (500 MHz, Chloroform-*d*) δ 7.63 (s, 1H), 7.59 – 7.52 (m, 2H), 7.47 (t, *J* = 7.7 Hz, 1H), 7.31 (d, *J* = 6.9 Hz, 2H), 7.26 – 7.16 (m, 3H), 3.86 (q, *J* = 6.6 Hz, 1H), 2.76 – 2.56 (m, 3H), 2.49 (dt, *J* = 11.7, 7.2 Hz, 1H), 1.84 (p, *J* = 7.4 Hz, 2H), 1.53 – 1.43 (m, 1H), 1.39 (d, *J* = 6.6 Hz, 3H). ^{13}C NMR (126 MHz, Chloroform-*d*) δ 146.9, 142.0, 130.8, 130.6, 130.0, 128.8, 128.3(d, *J* = 2.3 Hz), 125.7, 123.7(d, *J* = 4.0 Hz), 123.4(d, *J* = 3.7 Hz), 58.1, 53.4, 47.3, 33.6, 31.8, 24.4. $[\alpha]_D^{20}$ =-33.8 (c=1.0, EtOH). Enantiomeric excess was determined by chiral HPLC after the product was converted to the corresponding acetamide: AD-H, Hex/IPA = 95:5, 1 mL/min, 220 nm, 8.5 min, 9.1 min.

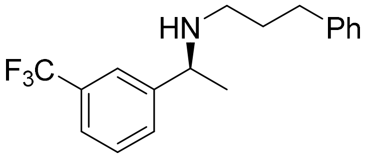

15

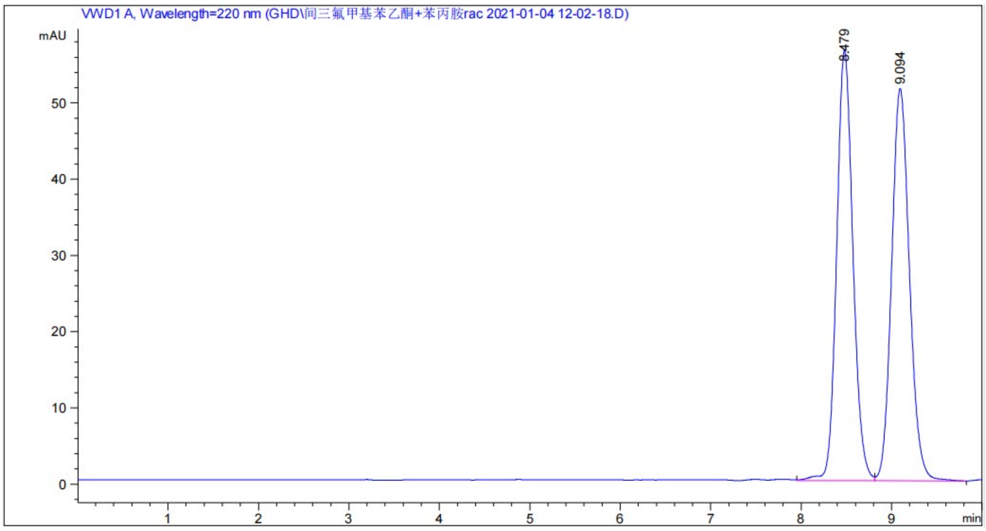

峰 #	保留时间 [min]	类型	峰宽 [min]	峰面积 [mAU*s]	峰高 [mAU]	峰面积 %
1	8.479	VV	0.1932	707.78253	56.49751	50.1234
2	9.094	VB	0.2116	704.29694	51.49147	49.8766

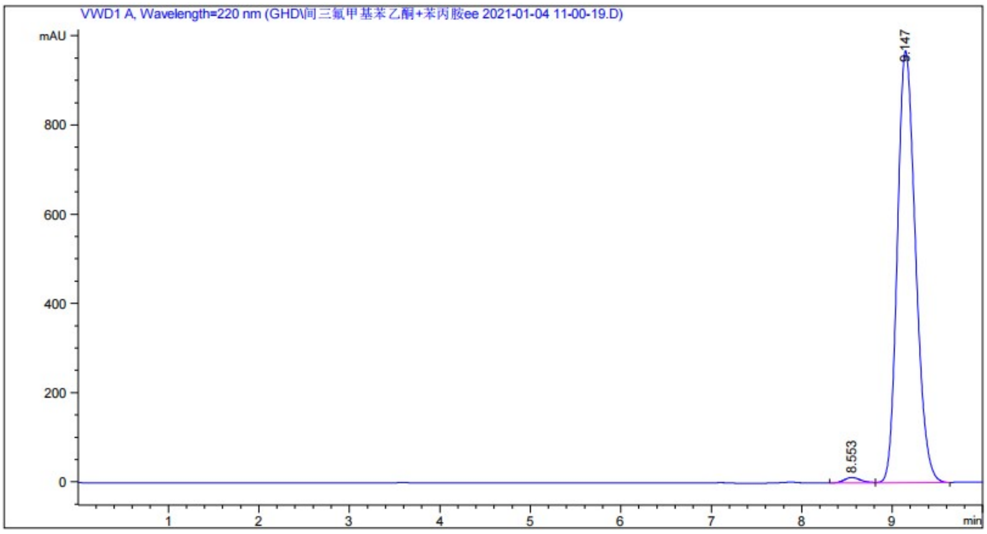

峰 #	保留时间 [min]	类型	峰宽 [min]	峰面积 [mAU*s]	峰高 [mAU]	峰面积 %
1	8.553	BV	0.1931	152.68581	12.19358	1.1173
2	9.147	VB	0.2169	1.35134e4	967.77924	98.8827

Supplementary Figure 14. HPLC spectra for racemic and chiral **15**.

3-phenyl-N-(1-(m-tolyl)ethyl)propan-1-amine(16): 96% yield, 98% ee, brown oil. ^{1}H NMR (500 MHz, Chloroform-*d*) δ 7.35 – 7.08 (m, 9H), 3.77 (q, J = 6.6 Hz, 1H), 2.75 – 2.50 (m, 4H), 2.41 (s, 3H), 1.85 (m, 2H), 1.60 (s, 1H), 1.40 (d, J = 6.6 Hz, 3H). ^{13}C NMR (126 MHz, Chloroform-*d*) δ 145.7, 142.2, 138.0, 128.3, 128.3, 127.65, 127.2, 125.7, 123.6, 58.3, 47.4, 33.7, 31.9, 24.2, 21.5. $[\alpha]_D^{20}$ =+43.8 (c=1.0, EtOH). Enantiomeric excess was determined by chiral HPLC after the product was converted to the corresponding acetamide: Chiralpak IB-3 column, Hex/IPA = 90:10, 1 mL/min, 220 nm, 9.1 min, 9.7 min.

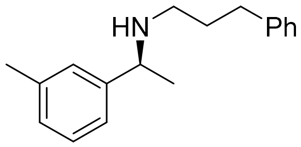

16

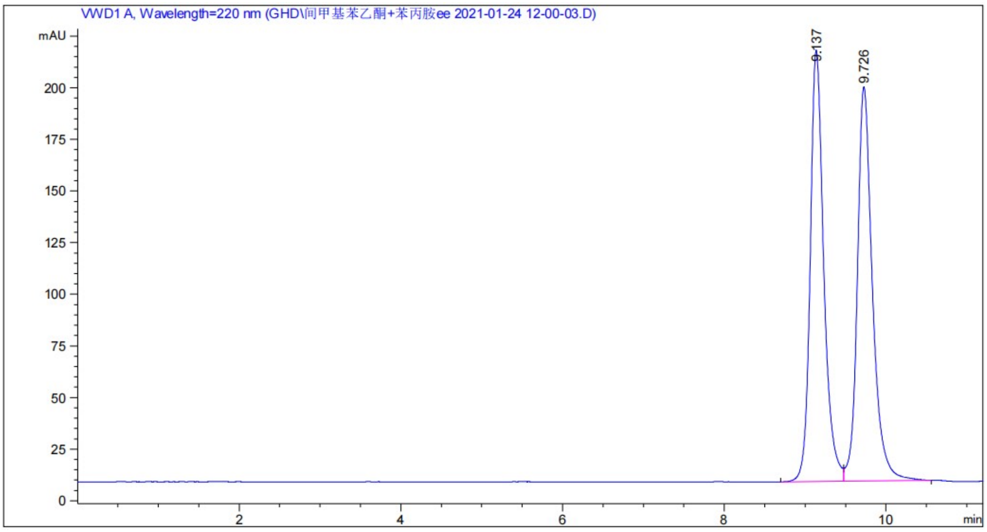

峰 #	保留时间 [min]	类型	峰宽 [min]	峰面积 [mAU*s]	峰高 [mAU]	峰面积 %
1	9.137	BV	0.1748	2431.52051	208.88414	49.5807
2	9.726	VB	0.1932	2472.65137	190.75246	50.4193

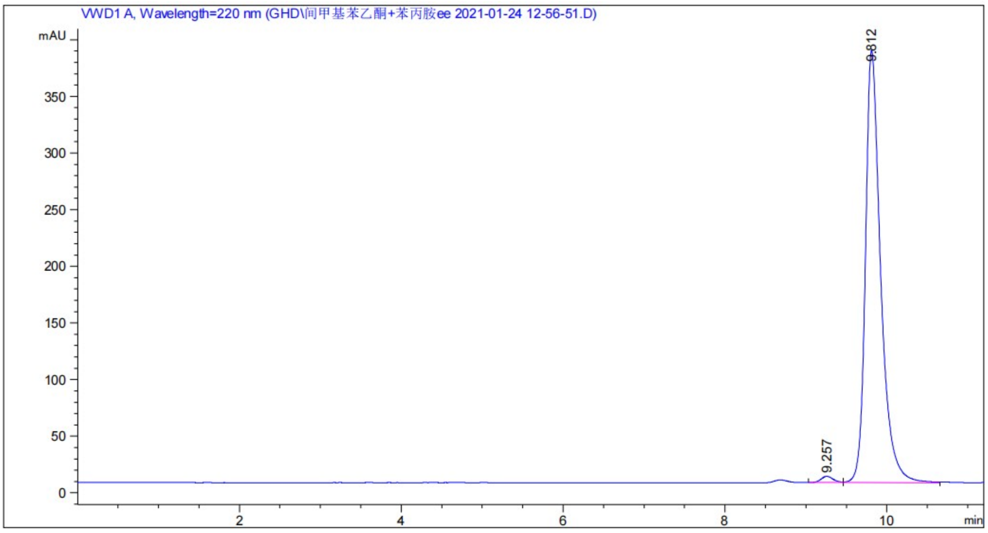

峰 #	保留时间 [min]	类型	峰宽 [min]	峰面积 [mAU*s]	峰高 [mAU]	峰面积 %
1	9.257	BV	0.1617	56.99795	5.46339	1.1290
2	9.812	VV	0.1946	4991.51660	381.60043	98.8710

Supplementary Figure 15. HPLC spectra for racemic and chiral **16**.

***N*-(1-(3-methoxyphenyl)ethyl)-3-phenylpropan-1-amine (17)**:[5] 95% yield, 96% ee, brown oil. ^{1}H NMR (500 MHz, $CDCl_3$): δ 7.32 (m, 3H), 7.23 (m, 3H), 6.97 (m, 2H), 6.85 (m, 1H), 3.88 (s, 3H), 3.80 (q, J = 6.6 Hz, 1H, CH), 2.70–2.54 (m, 4H), 1.86 (m, 2H), 1.64 (bs, 1H, NH), 1.40 (d, J = 6.6 Hz, 3H, CH_3). $[\alpha]_D^{20}$ =-44.6 (c=0.5, EtOH). Enantiomeric excess was determined by chiral HPLC after the product was converted to the corresponding acetamide: IB-3, Hex/IPA = 90:10, 1 mL/min, 220 nm, 14.0 min, 16.3 min.

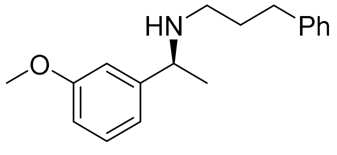

17

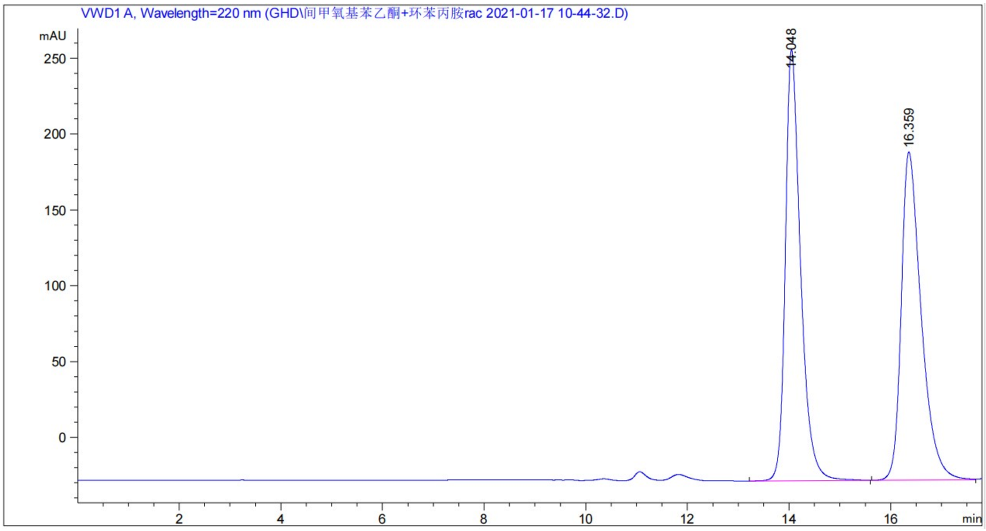

峰 #	保留时间 [min]	类型	峰宽 [min]	峰面积 [mAU*s]	峰高 [mAU]	峰面积 %
1	14.048	BB	0.3120	5919.79102	284.23993	50.0156
2	16.359	BV	0.4090	5916.10938	216.38806	49.9844

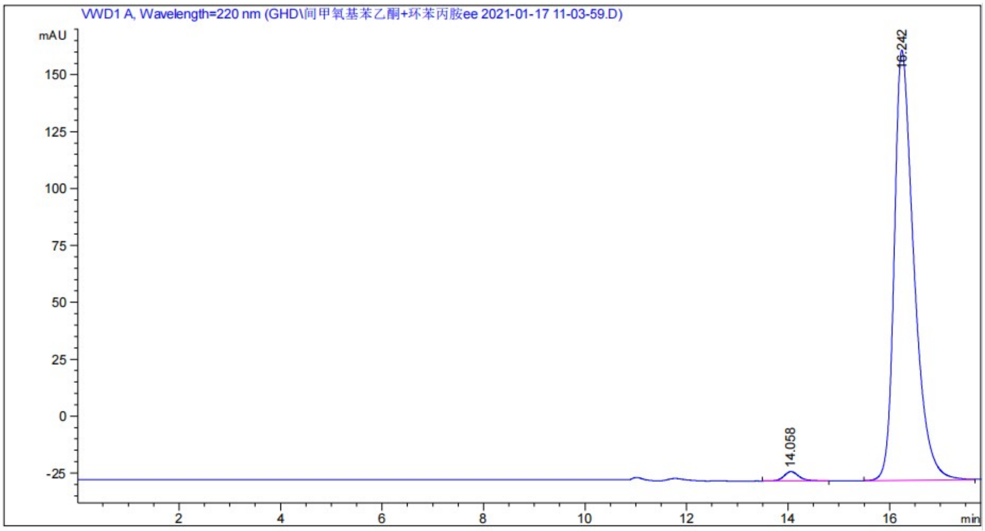

峰 #	保留时间 [min]	类型	峰宽 [min]	峰面积 [mAU*s]	峰高 [mAU]	峰面积 %
1	14.058	BB	0.3048	82.60979	4.10400	1.5994
2	16.242	BV	0.4027	5082.51660	189.01675	98.4006

Supplementary Figure 16. HPLC spectra for racemic and chiral **17**.

(1-(2-chlorophenyl)ethyl)-3-phenylpropan-1-amine (18): 96% yield, 95% ee, brown oil. ^{1}H NMR (500 MHz, $CDCl_3$): δ 7.53 (dd, J = 7.8, 1.8 Hz, 1H), 7.37 (dd, J = 7.9, 1.4 Hz, 1H), 7.34 – 7.28 (m, 3H), 7.25 – 7.16 (m, 4H), 4.31 (q, J = 6.6 Hz, 1H), 2.81 – 2.45 (m, 4H), 1.84 (dddd, J = 14.1, 8.8, 7.2, 2.3 Hz, 2H), 1.52 (s, 2H), 1.37 (d, J = 6.6 Hz, 3H); ^{13}C NMR (125 MHz, $CDCl_3$): δ 142.8, 142.2, 133.3, 129.6, 128.5, 128.4, 128.3, 127.8, 127.4, 127.1, 125.8, 54.2, 47.2, 33.7, 31.9, 22.7. HRMS (ESI) m/z calcd for $C_{17}H_{21}ClN^+$ $(M+H)^+$ 274.13570, found 274.13620. $[\alpha]_D^{20}$ =+27.9 (c=1.0, EtOH). Enantiomeric excess was determined by chiral HPLC after the product was converted to the corresponding acetamide: Chiralpak AD-H column, Hex/IPA = 90:10, 1 mL/min, 220n m, 7.7 min, 10.4 min.

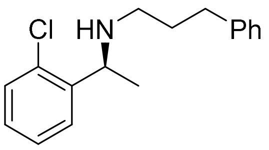

18

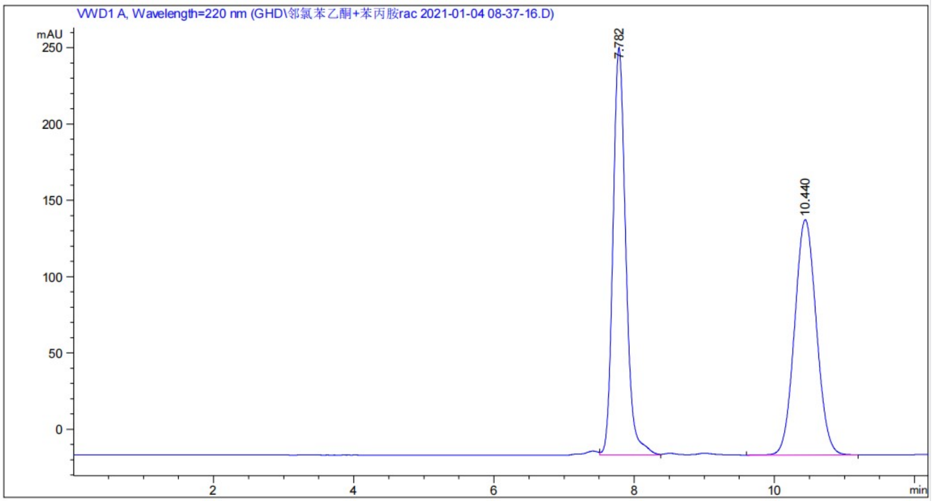

峰 #	保留时间 [min]	类型	峰宽 [min]	峰面积 [mAU*s]	峰高 [mAU]	峰面积 %
1	7.782	VV	0.1949	3381.49048	266.81531	50.4003
2	10.440	BB	0.3379	3327.77271	154.11115	49.5997

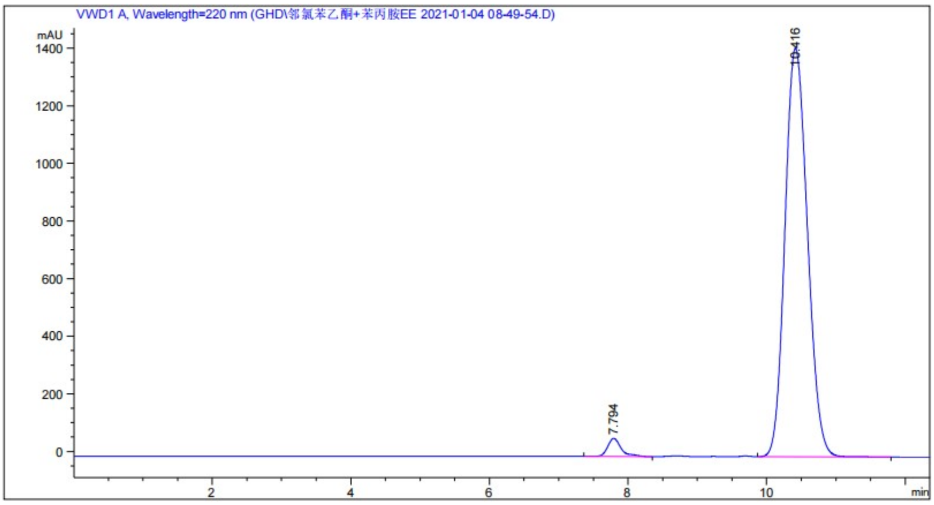

峰 #	保留时间 [min]	类型	峰宽 [min]	峰面积 [mAU*s]	峰高 [mAU]	峰面积 %
1	7.794	VB	0.2054	857.52393	63.14200	2.6172
2	10.416	VB	0.3565	3.19077e4	1418.36072	97.3828

Supplementary Figure 17. HPLC spectra for racemic and chiral **18**.

(1-(2-bromophenyl) ethyl)-3-phenylpropan-1-amine (19): 97% yield, 96% ee, yellow oily liquid. ^{1}H NMR (500 MHz, Chloroform-d) δ 7.60 (ddd, J = 10.4, 7.9, 1.5 Hz, 2H), 7.44 – 7.32 (m, 3H), 7.26 (dd, J = 7.7, 5.6 Hz, 3H), 7.17 (td, J = 7.6, 1.7 Hz, 1H), 4.33 (q, J = 6.6 Hz, 1H), 2.83 – 2.49 (m, 4H), 1.89 (tt, J = 9.1, 6.3 Hz, 2H), 1.41 (d, J = 6.7 Hz, 3H); ^{13}C NMR (125 MHz, $CDCl_3$): δ 144.3, 142.2, 132.9, 128.4, 128.3, 128.2, 127.8, 127.6, 125.8, 123.7, 56.7, 47.2, 33.7, 32.0, 22.8. HRMS (ESI) m/z calcd for $C_{17}H_{21}BrN^{+}$ (M+H)$^{+}$ 318.08519, found 318.08578. $[\alpha]_D^{20}$ =-18.9 (c=1.0, EtOH). Enantiomeric excess was determined by chiral HPLC after the product was converted to the corresponding acetamide: Chiralpak AD-H column, Hex/IPA = 90:10, 1 mL/min, 220 nm, 8.4 min, 11.4 min.

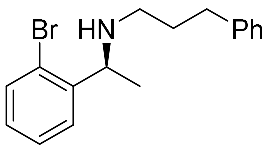

19

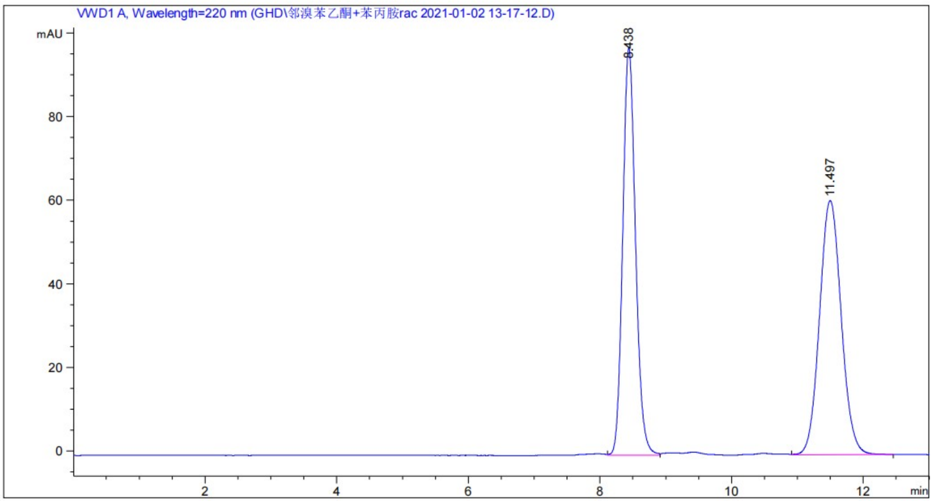

峰 #	保留时间 [min]	类型	峰宽 [min]	峰面积 [mAU*s]	峰高 [mAU]	峰面积 %
1	8.438	VV	0.2083	1314.39392	97.50633	48.5303
2	11.497	VB	0.3574	1394.00635	60.82961	51.4697

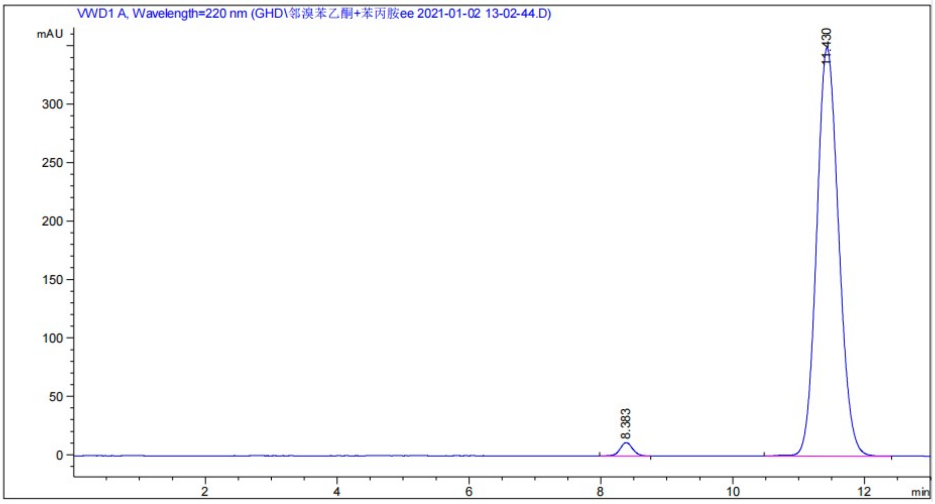

峰 #	保留时间 [min]	类型	峰宽 [min]	峰面积 [mAU*s]	峰高 [mAU]	峰面积 %
1	8.383	VB	0.2050	152.21764	11.45937	1.8485
2	11.430	BV	0.3608	8082.22754	349.47549	98.1515

Supplementary Figure 18. HPLC spectra for racemic and chiral **19**.

(1-(o-tolyl)ethyl)-3-phenylpropan-1-amine (20): 96% yield, 98% ee, brown oil. ^{1}H NMR (500 MHz, $CDCl_3$): δ 7.50 (d, *J* = 7.7 Hz, 1H), 7.39–7.15 (m, 8H), 4.10 (q, *J* = 6.6 Hz, 1H), 2.77–2.53 (m, 4H), 2.41 (s, 3H), 1.93 – 1.81 (m, 2H), 1.54 (s, 1H), 1.37 (d, *J* = 6.6 Hz, 3H); ^{13}C NMR (125 MHz, $CDCl_3$): δ 143.8, 142.3, 135.2, 130.3, 126.4, 126.3, 125.7, 125.1, 53.6, 47.4, 33.7, 32.0, 23.1, 19.2. HRMS (ESI) m/z calcd for $C_{18}H_{24}N^+$ $(M+H)^+$ 254.19033, found 254.19067. $[\alpha]_D^{20}$ =-39.1 (c=1.0, EtOH). Enantiomeric excess was determined by chiral HPLC after the product was converted to the corresponding acetamide: Chiralpak IB-3 column, Hex/IPA = 90:10, 1 mL/min, 220 nm, 15.7 min, 17.0 min.

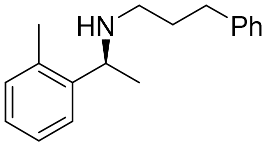

20

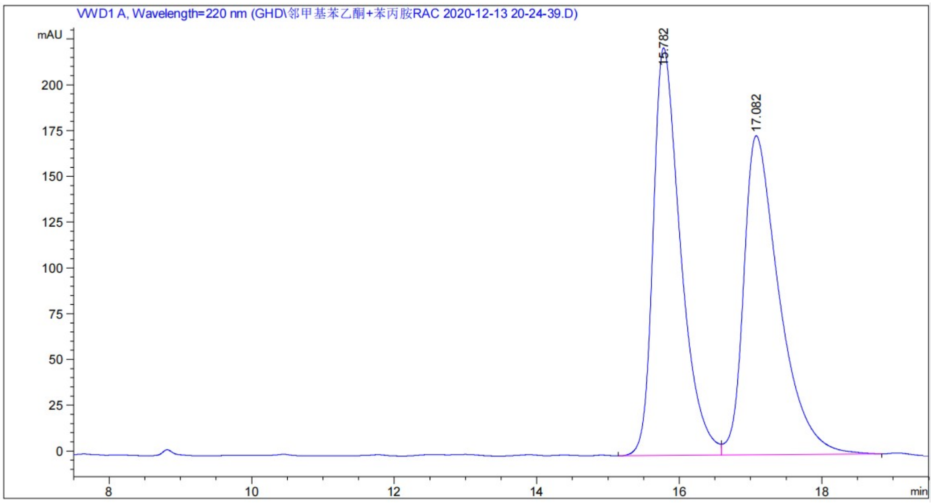

峰 #	保留时间 [min]	类型	峰宽 [min]	峰面积 [mAU*s]	峰高 [mAU]	峰面积 %
1	15.782	BV	0.4006	5966.82617	222.66689	49.6324
2	17.082	VB	0.5174	6055.21826	174.26689	50.3676

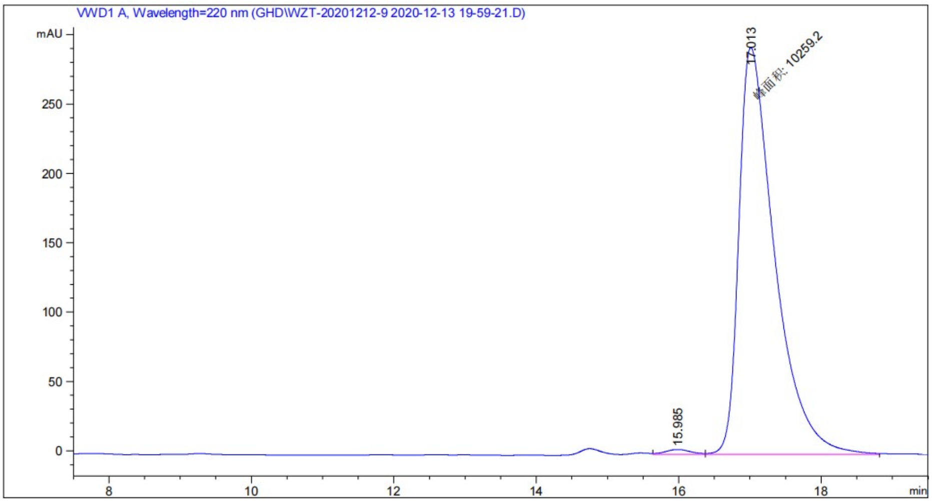

峰 #	保留时间 [min]	类型	峰宽 [min]	峰面积 [mAU*s]	峰高 [mAU]	峰面积 %
1	15.985	VV	0.3835	91.58893	3.61593	0.8848
2	17.013	MF	0.5826	1.02592e4	293.49719	99.1152

Supplementary Figure 19. HPLC spectra for racemic and chiral **20**.

***N*-(1-(2-methoxyphenyl)ethyl)-3-phenylpropan-1-amine (21)**: 97% yield, 95% ee, brown oil. ^{1}H NMR (500 MHz, $CDCl_3$): δ7.40 – 7.19 (m, 7H), 7.07 – 6.99 (m, 1H), 6.95 (d, J = 8.2 Hz, 1H), 4.18 (q, J = 6.7 Hz, 1H), 3.89 (s, 3H), 2.76 – 2.64 (m, 2H), 2.59 (m, 2H), 2.32 – 2.21 (m, 2H), 1.97 – 1.79 (s, 1H), 1.45 (d, J = 6.7 Hz, 3H); ^{13}C NMR (125 MHz, $CDCl_3$): δ 157.2, 142.3, 128.4, 128.3,127.6, 127.2, 125.7, 120.7, 55.3, 52.8, 47.2, 33.7, 31.9, 22.1. $[\alpha]_D^{20}$ =-32.7 (c=1.0, EtOH). Enantiomeric excess was determined by chiral HPLC after the product was converted to the corresponding acetamide: Chiralpak AD-H column, Hex/IPA = 90:10, 1 mL/min, 220 nm, 9.6 min, 10.5 min.

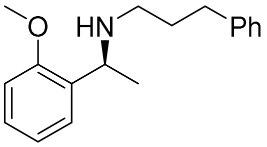

21

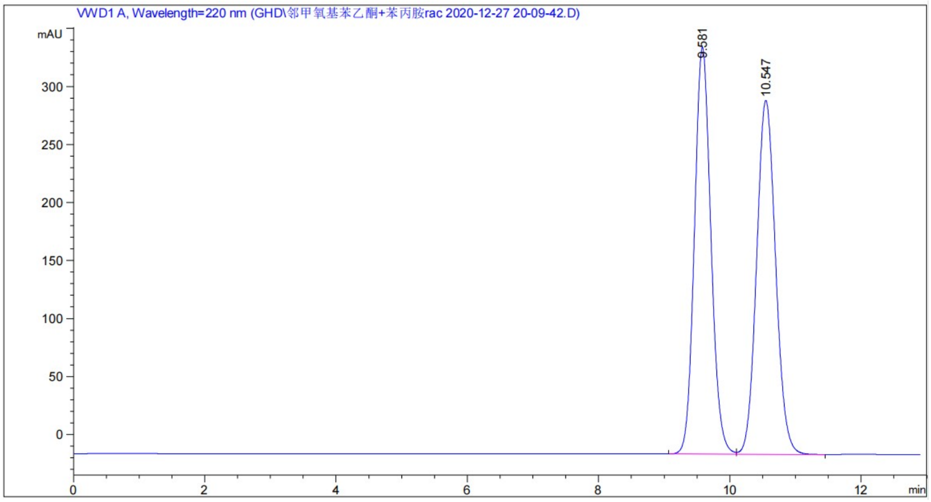

峰 #	保留时间 [min]	类型	峰宽 [min]	峰面积 [mAU*s]	峰高 [mAU]	峰面积 %
1	9.581	BV	0.2704	6113.12207	350.56598	49.9079
2	10.547	VB	0.3137	6135.69043	305.05417	50.0921

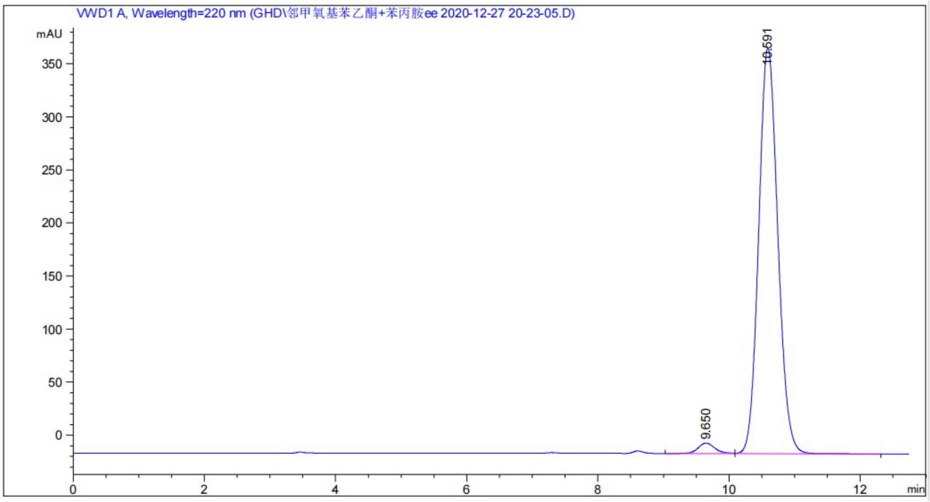

峰 #	保留时间 [min]	类型	峰宽 [min]	峰面积 [mAU*s]	峰高 [mAU]	峰面积 %
1	9.650	BV	0.2804	185.06808	9.97800	2.3333
2	10.591	VB	0.3141	7746.66553	382.77136	97.6667

Supplementary Figure 20. HPLC spectra for racemic and chiral **21**.

***N*-(1-([1,1'-biphenyl]-2-yl)ethyl)-3-phenylpropan-1-amine (22)**: 53% yield, 94% ee, brown oil, unknown compound. ^{1}H NMR (500 MHz, $CDCl_3$): δ7.68 (dd, *J* = 7.9, 2.0 Hz, 1H), 7.47 (dt, *J* = 15.9, 7.5 Hz, 4H), 7.40 – 7.23 (m, 7H), 7.19 (d, *J* = 7.4 Hz, 2H), 4.01 (qd, *J* = 6.6, 3.1 Hz, 1H), 2.64 (qt, *J* = 14.2, 7.6 Hz, 2H), 2.52 – 2.43 (m, 2H), 1.82 – 1.67 (m, 2H), 1.40–1.26 (m, 4H); ^{13}C NMR (125 MHz, $CDCl_3$): δ 143.4, 142.3, 141.7, 141.6, 129.8, 129.4, 128.4, 128.3, 128.1, 128.0, 127.9, 126.9, 126.2, 125.7, 53.1, 47.1, 33.6, 31.9, 24.1. HRMS (ESI) m/z calcd for $C_{23}H_{26}N^{+}$ $(M+H)^{+}$ 316.20598, found 316.20630. $[\alpha]_D^{20}$ =-21.2 (c=0.5, EtOH). Enantiomeric excess was determined by chiral HPLC after the product was converted to the corresponding acetamide: OD-H, Hex/IPA = 85:15, 1 mL/min, 220 nm, 7.1 min, 9.0 min.

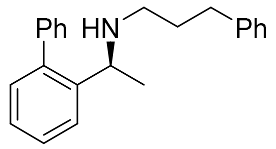

22

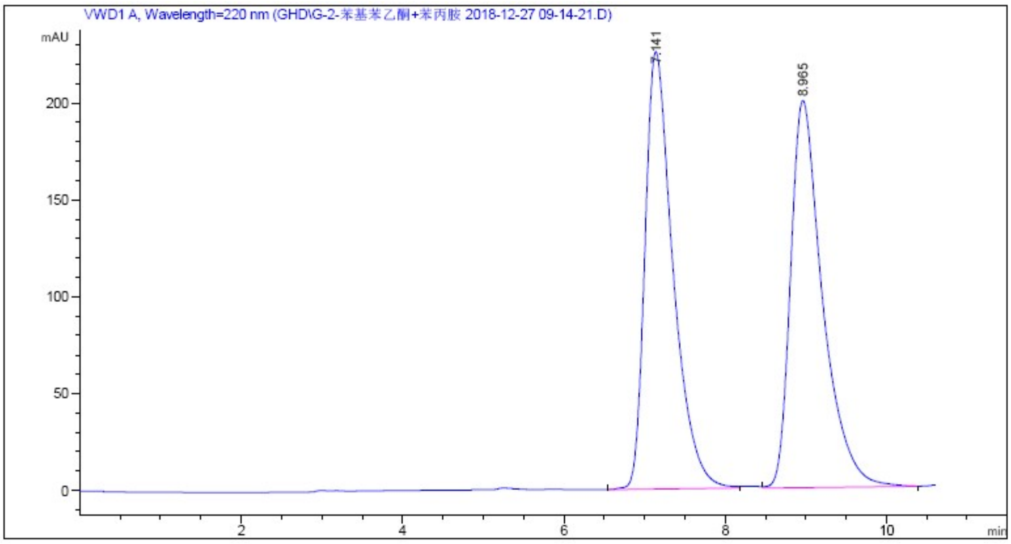

#	[min]		[min]	[mAU*s]	[mAU]	%
1	7.141	BV	0.3615	5465.61084	225.73409	49.8326
2	8.965	VB	0.4104	5502.32422	199.72755	50.1674

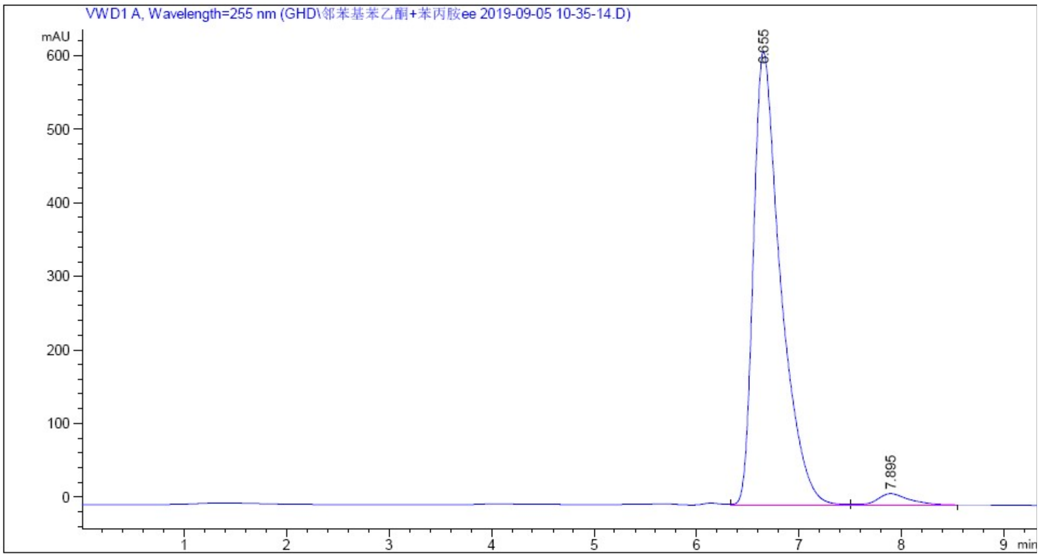

#	[min]		[min]	[mAU*s]	[mAU]	%
1	6.655	VV	0.2732	1.13652e4	616.05420	96.9914
2	7.895	VV	0.3241	352.53864	15.81240	3.0086

Supplementary Figure 21. HPLC spectra for racemic and chiral **22**.

***N*-(1-(2,4-dimethylphenyl)ethyl)-3-phenylpropan-1-amine (23)**: 95% yield, 97% ee, brown oil. ^{1}H NMR (500 MHz, $CDCl_3$): δ 7.38 (d, *J* = 7.9 Hz, 1H), 7.35 – 7.30 (m, 2H), 7.26 – 7.20 (m, 3H), 7.10 (dd, *J* = 7.9, 1.8 Hz, 1H), 7.02 (d, *J* = 1.8 Hz, 1H), 4.07 (q, *J* = 6.6 Hz, 1H), 2.77 – 2.52 (m, 4H), 2.37 (d, *J* = 2.1 Hz, 6H), 1.93 – 1.78 (m, 2H), 1.60 (s, 1H), 1.35 (d, *J* = 6.6 Hz, 3H); ^{13}C NMR (125 MHz, $CDCl_3$): δ 142.3, 140.7, 135.8, 135.0, 131.1, 128.4, 128.3, 127.1, 125.7, 125.1, 53.3, 47.4, 33.7, 32.0, 23.2, 21.0, 19.1. HRMS (ESI) m/z calcd for $C_{19}H_{26}N^+$ $(M+H)^+$ 268.20598, found 268.20630. $[\alpha]_D^{20}$ =-50.67 (c=0.2, EtOH). Enantiomeric excess was determined by chiral HPLC after the product was converted to the corresponding acetamide: Chiralpak AD-H column, Hex/IPA = 90:10, 1 mL/min, 220 nm, 4.7 min, 5.3 min.

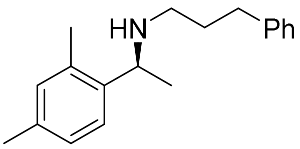

23

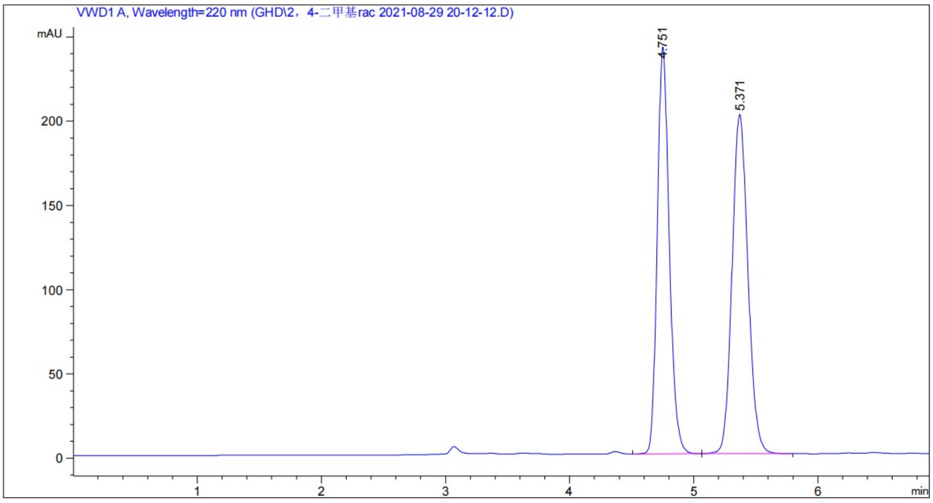

峰 #	保留时间 [min]	类型	峰宽 [min]	峰面积 [mAU*s]	峰高 [mAU]	峰面积 %
1	4.751	BB	0.1034	1623.08215	241.40559	47.5304
2	5.371	BV	0.1389	1791.75098	201.28769	52.4696

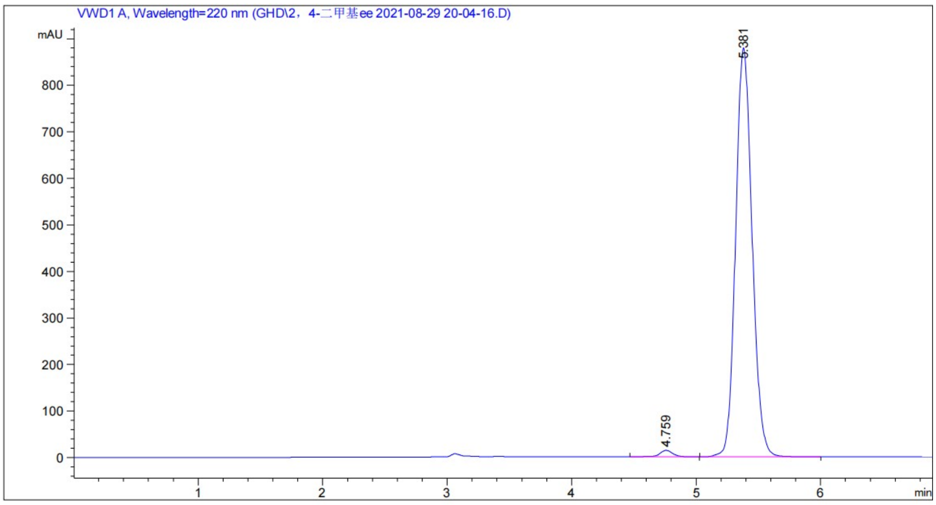

峰 #	保留时间 [min]	类型	峰宽 [min]	峰面积 [mAU*s]	峰高 [mAU]	峰面积 %
1	4.759	BB	0.1142	108.93918	14.24308	1.3268
2	5.381	BV	0.1435	8101.86572	878.89679	98.6732

Supplementary Figure 22. HPLC spectra for racemic and chiral **23**.

3-phenyl-N-(1-phenylpropyl)propan-1-amine (24): 89% yield, 90% ee, brown oil. ^{1}H NMR (500 MHz, Chloroform-*d*) δ 7.41 (t, *J* = 7.5 Hz, 2H), 7.38 – 7.29 (m, 5H), 7.28 – 7.19 (m, 3H), 3.64 (dd, *J* = 7.8, 6.1 Hz, 1H), 2.77 – 2.50 (m, 4H), 1.92 – 1.63 (m, 4H), 1.56 – 1.42 (m, 1H), 1.36 (dddd, *J* = 13.3, 10.1, 7.6, 6.0 Hz, 1H), 1.31 – 1.20 (m, 1H), 0.97 (t, *J* = 7.4 Hz, 3H). $[\alpha]_D^{20}$ =-25.7 (c=1.0, EtOH). Enantiomeric excess was determined by chiral HPLC after the product was converted to the corresponding acetamide: IB-3, Hex/IPA = 90:10, 1 mL/min, 220 nm, 9.2 min, 10.4 min.

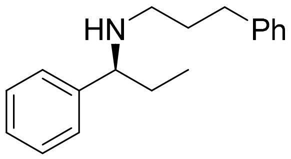

24

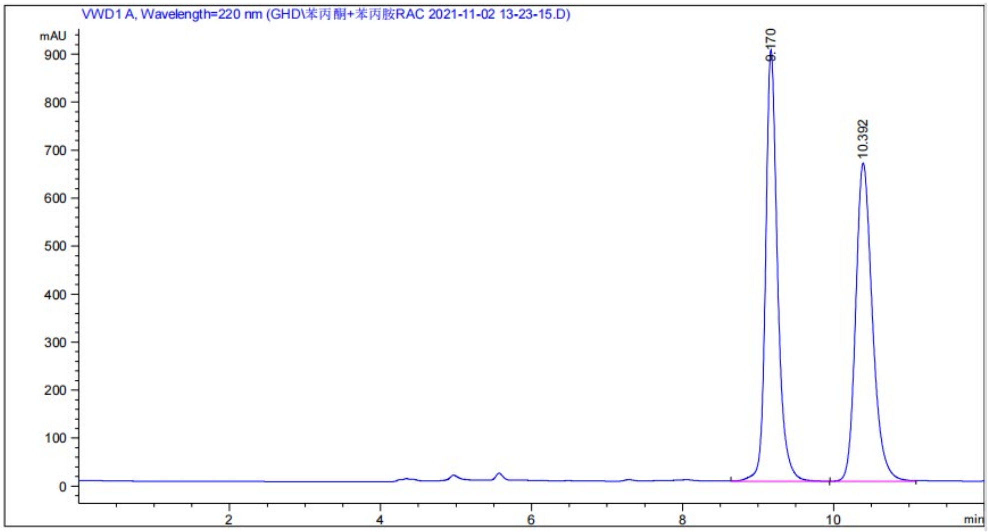

峰 #	保留时间 [min]	类型	峰宽 [min]	峰面积 [mAU*s]	峰高 [mAU]	峰面积 %
1	9.170	VB	0.1663	9872.37988	898.04712	49.1254
2	10.392	BV	0.2359	1.02239e4	663.18542	50.8746

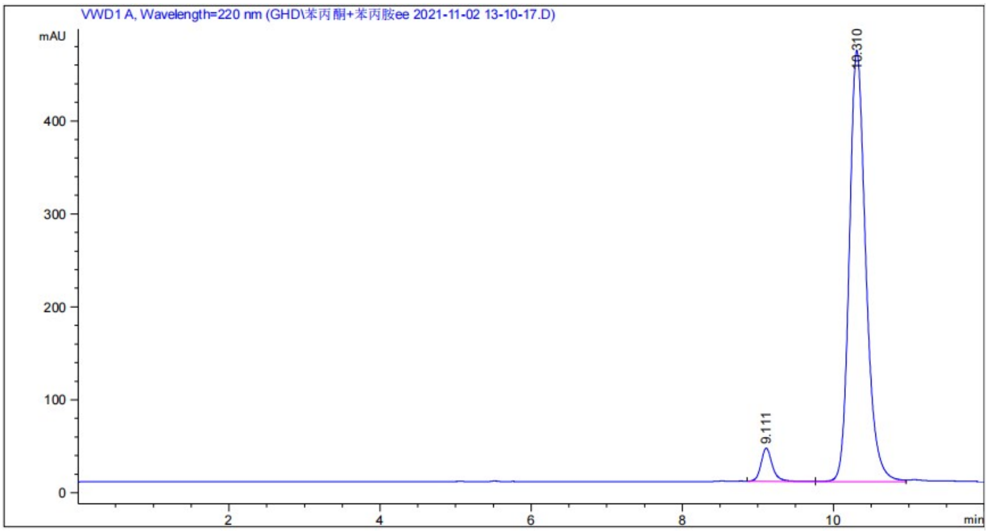

峰 #	保留时间 [min]	类型	峰宽 [min]	峰面积 [mAU*s]	峰高 [mAU]	峰面积 %
1	9.111	VV	0.1601	381.24554	35.84549	5.1245
2	10.310	VV	0.2335	7058.48340	463.96967	94.8755

Supplementary Figure 23. HPLC spectra for racemic and chiral **24**.

N-(1-(naphthalen-2-yl)ethyl)-3-phenylpropan-1-amine (25): 96% yield, 98% ee, brown oil. 1H NMR (400 MHz, Chloroform-d) δ 7.86 – 7.75 (m, 3H), 7.74 – 7.68 (m, 1H), 7.44 (dtd, J = 12.3, 6.9, 6.3, 3.5 Hz, 3H), 7.22 (t, J = 7.4 Hz, 2H), 7.18 – 7.08 (m, 3H), 3.90 (q, J = 6.6 Hz, 1H), 2.55 (tdd, J = 26.4, 12.8, 6.9 Hz, 4H), 1.86 – 1.71 (m, 2H), 1.67 – 1.50 (m, 1H), 1.41 (d, J = 6.6 Hz, 3H). ^{13}C NMR (101 MHz, Chloroform-d) δ 143.2, 142.2, 128.4, 128.3, 128.2, 127.8, 127.7, 126.0, 125.7, 125.5, 125.2, 124.9, 58.5, 47.4, 33.7, 31.9, 24.3. $[\alpha]_D^{20}$ =-20.4 (c=1.0, EtOH). Enantiomeric excess was determined by chiral HPLC after the product was converted to the corresponding acetamide: AD-H, Hex/IPA = 90:10, 1 mL/min, 220 nm, 7.5 min, 8.4 min.

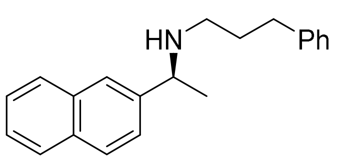

25

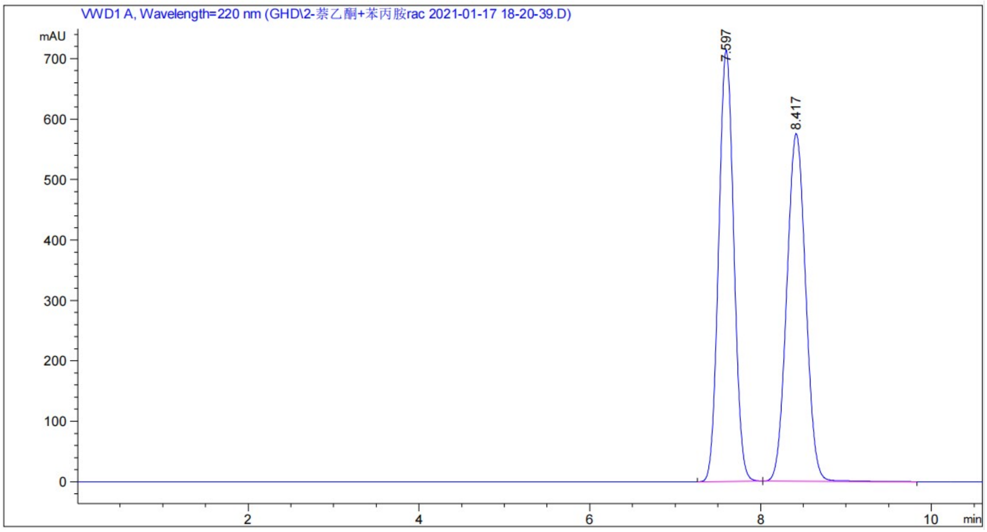

峰 #	保留时间 [min]	类型	峰宽 [min]	峰面积 [mAU*s]	峰高 [mAU]	峰面积 %
1	7.597	BB	0.1926	8781.92871	713.64838	49.8084
2	8.417	BB	0.2416	8849.49512	574.87360	50.1916

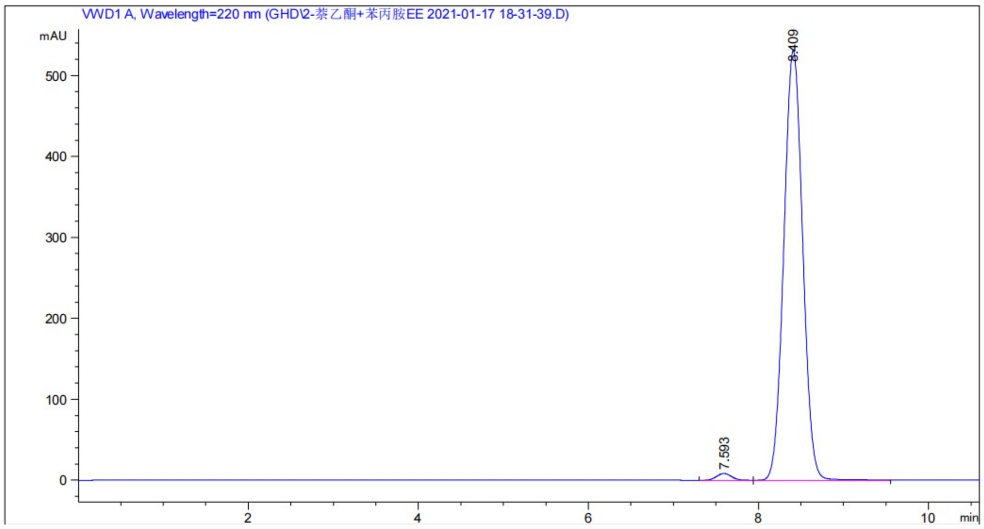

峰 #	保留时间 [min]	类型	峰宽 [min]	峰面积 [mAU*s]	峰高 [mAU]	峰面积 %
1	7.593	BV	0.1932	104.49990	8.45298	1.2609
2	8.409	VV	0.2418	8182.92822	531.06030	98.7391

Supplementary Figure 24. HPLC spectra for racemic and chiral **25**.

(1-(naphthalen-1-yl)ethyl)-3-phenylpropan-1-amine (26):[4] 96% yield, 98% ee, colorless oil, ^{1}H NMR (500 MHz, Chloroform-d) δ 8.30 (d, J = 8.3 Hz, 1H), 7.98 (dd, J = 7.8, 1.7 Hz, 1H), 7.86 (d, J = 8.1 Hz, 1H), 7.77 (d, J = 7.1 Hz, 1H), 7.65 – 7.54 (m, 3H), 7.37 (t, J = 7.5 Hz, 3H), 7.32 – 7.24 (m, 4H), 4.73 (d, J = 6.6 Hz, 1H), 2.91 – 2.62 (m, 4H), 2.09 – 1.84 (m, 2H), 1.60 (d, J = 6.7 Hz, 3H). $[\alpha]_D^{20}$ =+22.0 (c=1.0, EtOH). Enantiomeric excess was determined by chiral HPLC after the product was converted to the corresponding acetamide: Chiralpak AS-H column, Hex/IPA = 90:10, 1 mL/min, 220 nm, 10.2 min, 14.5 min.

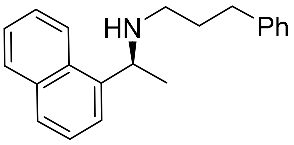

26

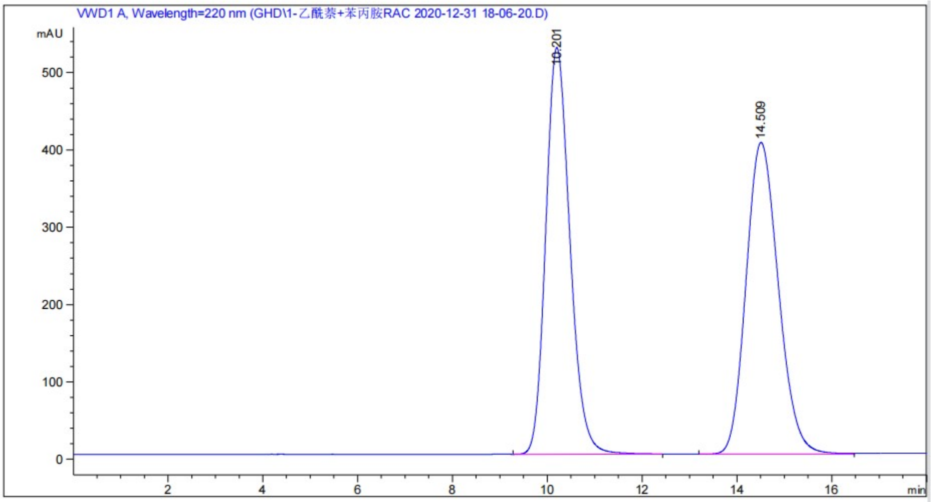

峰 #	保留时间 [min]	类型	峰宽 [min]	峰面积 [mAU*s]	峰高 [mAU]	峰面积 %
1	10.201	VV	0.5472	1.85870e4	526.04523	49.2974
2	14.509	BV	0.7373	1.91169e4	403.11841	50.7026

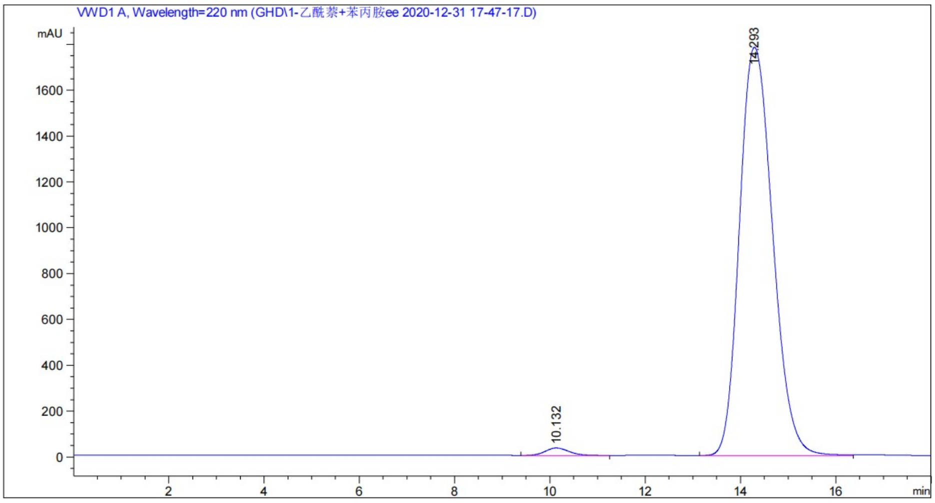

峰 #	保留时间 [min]	类型	峰宽 [min]	峰面积 [mAU*s]	峰高 [mAU]	峰面积 %
1	10.132	BB	0.5361	1111.56519	32.40990	1.2743
2	14.293	BV	0.7552	8.61167e4	1780.68140	98.7257

Supplementary Figure 25. HPLC spectra for racemic and chiral **26**.

***N*-(1,2-diphenylethyl)-3-phenylpropan-1-amine (27)**: 92% yield, 85% ee, colorless oil. ^{1}H NMR (500 MHz, $CDCl_3$): δ 7.46–7.22 (m, 13H), 7.13 (d, *J* = 7.4 Hz, 2H), 3.94 (m, 1H), 3.10–2.85 (m, 2H), 2.62–2.45 (m, 4H), 1.80 (m, 2H), 1.55 (b, 1H). $[\alpha]_D^{20}$ =+22.3 (c=1.0, EtOH). Enantiomeric excess was determined by chiral HPLC after the product was converted to the corresponding acetamide: OD-H, Hex/IPA = 90:10, 1 mL/min, 220 nm, 23.8 min, 27.7 min.

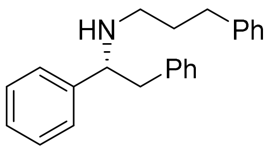

27

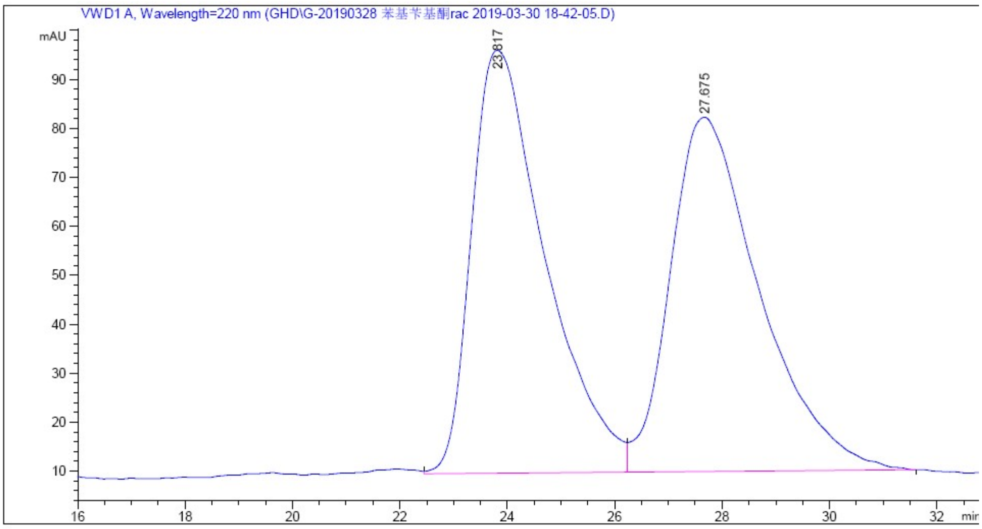

#	[min]		[min]	[mAU*s]	[mAU]	%
1	23.817	VV	1.4059	8248.19922	86.39004	49.6089
2	27.675	VB	1.7542	8378.25977	72.39207	50.3911

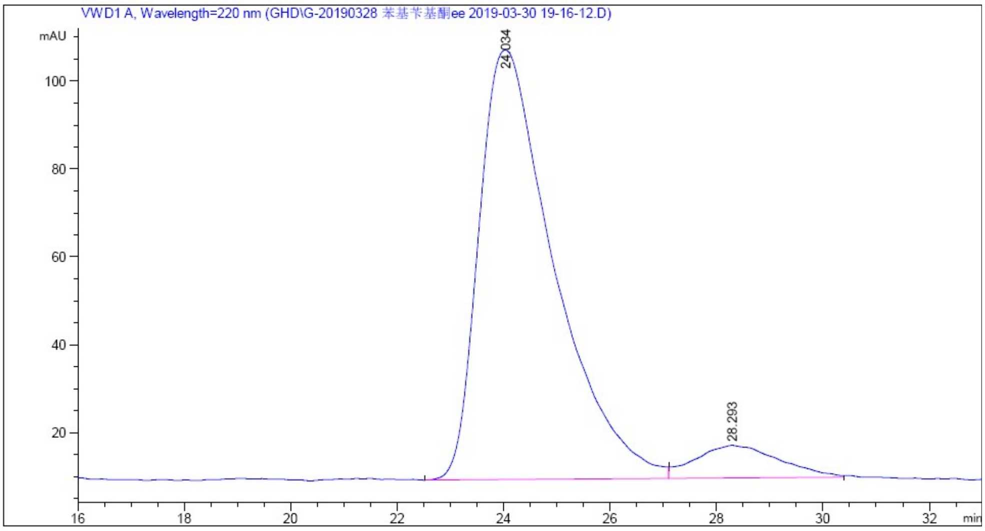

#	[min]		[min]	[mAU*s]	[mAU]	%
1	24.034	BV	1.4410	9575.70898	97.74210	92.1288
2	28.293	VV	1.3554	818.11957	7.43730	7.8712

Supplementary Figure 26. HPLC spectra for racemic and chiral **27**.

2-methyl-1-phenyl-N-(3-phenylpropyl)propan-1-amine (28): 95% yield, 94% ee, colorless oil. ^{1}H NMR (500 MHz, $CDCl_3$):

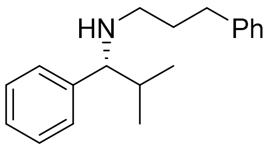

28

δ 7.40 (t, J = 7.4 Hz, 2H), 7.37 – 7.30 (m, 5H), 7.28 – 7.21 (m, 3H), 3.38 (d, J = 7.0 Hz, 1H), 2.79 – 2.61 (m, 2H), 2.53 (t, J = 6.6 Hz, 2H), 1.94 (h, J = 6.7 Hz, 1H), 1.89 – 1.80 (m, 2H), 1.43 (s, 1H), 1.06 (d, J = 6.7 Hz, 3H), 0.84 (d, J = 6.8 Hz, 3H); ^{13}C NMR (125 MHz, $CDCl_3$): δ 143.2, 142.4, 128.4, 128.3, 128.0, 128.0, 126.7, 126.9, 125.7, 69.7, 47.4, 34.4, 33.6, 31.9, 19.8, 19.5. HRMS (ESI) m/z calcd for $C_{19}H_{26}N^+$ $(M+H)^+$ 268.20598, found 268.20624. $[\alpha]_D^{20}$ =+23.8 (c=0.5, EtOH). Enantiomeric excess was determined by chiral HPLC after the product was converted to the corresponding acetamide: OD-H, Hex/IPA = 85:15, 1 mL/min, 220 nm, 8.0 min, 9.4 min.

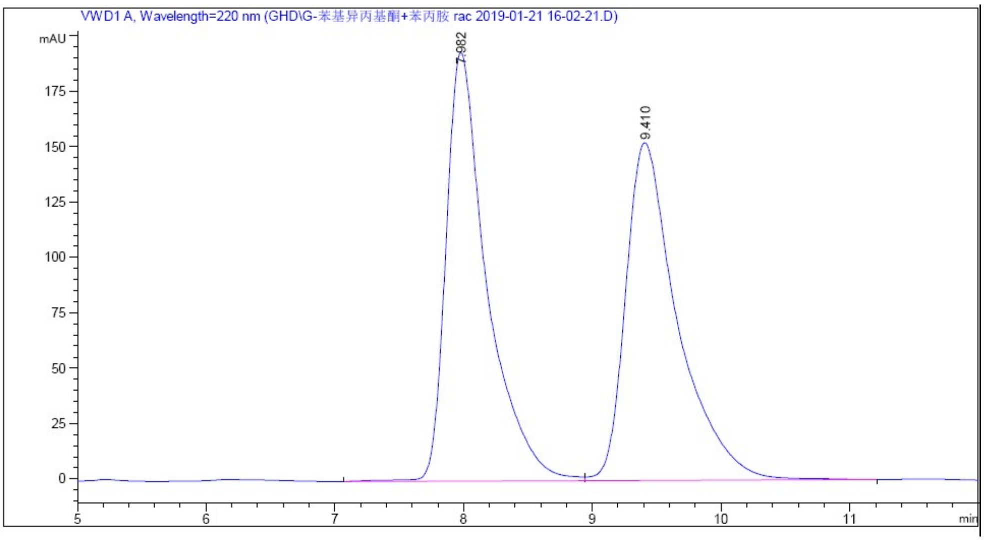

#	[min]		[min]	[mAU*s]	[mAU]	%
1	7.982	BV	0.3237	4269.64355	193.32703	49.9726
2	9.410	VB	0.4113	4274.32959	152.37090	50.0274

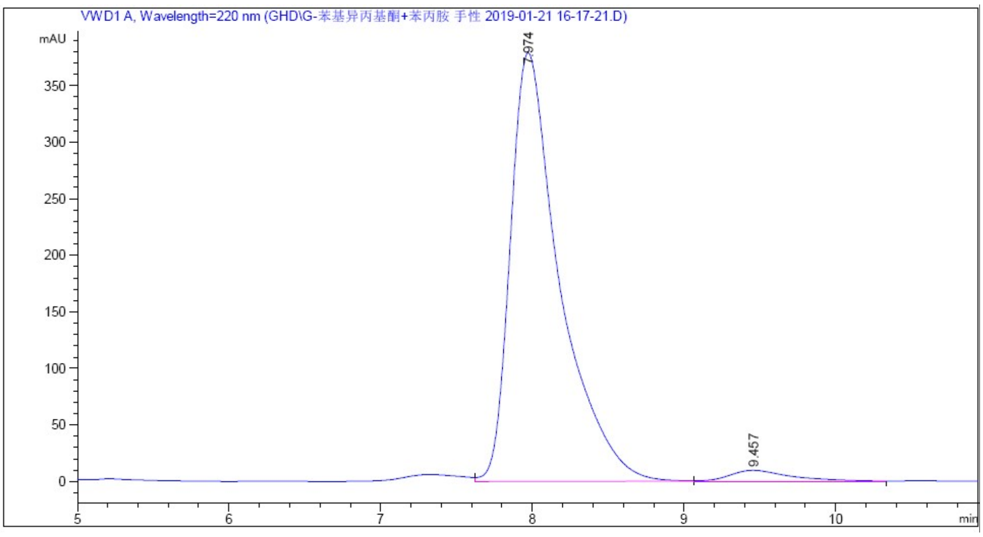

#	[min]		[min]	[mAU*s]	[mAU]	%
1	7.974	VV	0.3317	8567.13379	379.10004	97.0371
2	9.457	VB	0.4054	261.58417	9.55563	2.9629

Supplementary Figure 27. HPLC spectra for racemic and chiral **28**.

***N*-(cyclohexyl(phenyl)methyl)-3-phenylpropan-1-amine (29)**: 94% yield, 94% ee, colorless oil. ^{1}H NMR (500 MHz, $CDCl_3$): δ 7.41–7.20 (m, 5H), 3.40 (d, *J* = 7.2 Hz, 1H), 2.80–2.60 (m, 2H), 2.50 (m, 2H), 2.00 (d, *J* = 12.7 Hz, 1H), 1.85 (m, 3H), 1.71 (m, 2H), 1.60 (m, 1H), 1.55–1.40 (m, 2H), 1.40–1.10 (m, 3H),1.06 (m, 1H), 0.92 (m,1H); ^{13}C NMR (125 MHz, $CDCl_3$): δ 143.4, 142.5, 128.4, 128.3, 128.1, 128.0, 126.7, 125.7, 69.0, 47.3, 44.4, 33.7, 32.0, 30.4, 30.0, 26.7, 26.5, 26.0. HRMS (ESI) m/z calcd for $C_{22}H_{30}N^+$ $(M+H)^+$ 308.23728, found 308.23795. $[\alpha]_D^{20}$ =+21.3 (c=1.0, EtOH). Enantiomeric excess was determined by chiral HPLC after the product was converted to the corresponding acetamide: OD-H, Hex/IPA = 80:10, 1 mL/min, 220 nm, 7.3 min, 9.1 min.

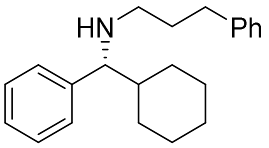

29

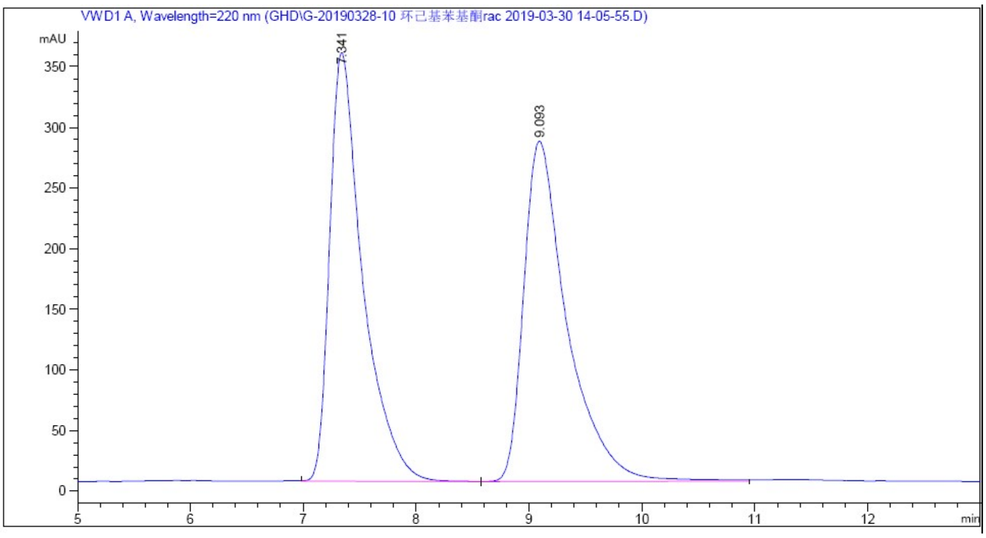

#	[min]		[min]	[mAU*s]	[mAU]	%
1	7.341	VB	0.2997	7200.06592	353.44870	48.9872
2	9.093	BV	0.3918	7497.78369	280.50223	51.0128

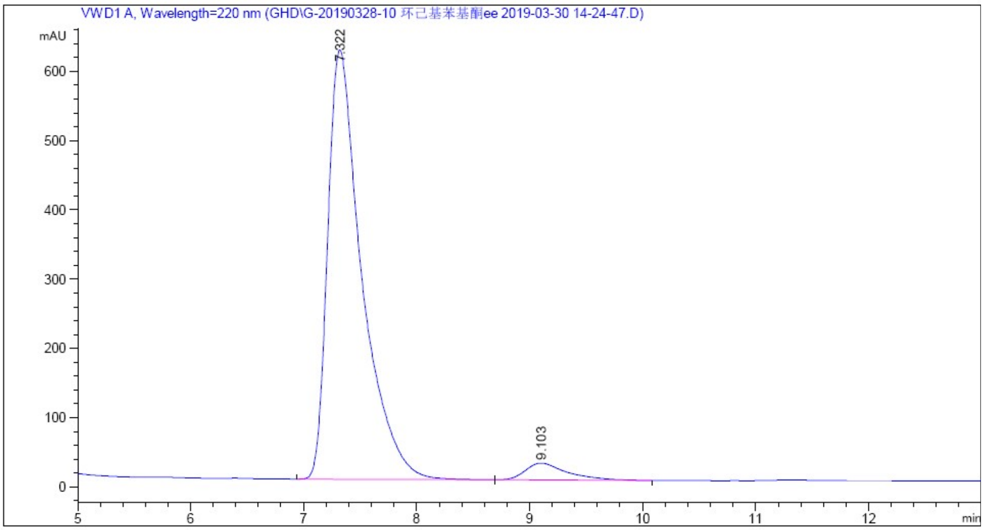

#	[min]		[min]	[mAU*s]	[mAU]	%
1	7.322	BV	0.3083	1.29791e4	619.87518	95.3940
2	9.103	VB	0.3775	626.68811	24.41291	4.6060

Supplementary Figure 28. HPLC spectra for racemic and chiral **29**.

N-(1-(4-nitrophenyl)ethyl)-3-phenylpropan-1-amine (30): 94% yield, 98% ee, brown oil. ^{1}H NMR (400 MHz, Chloroform-*d*) δ 8.17 (d, *J* = 8.3 Hz, 2H), 7.48 (d, *J* = 8.3 Hz, 2H), 7.26 (t, *J* = 7.4 Hz, 2H), 7.16 (dd, *J* = 16.2, 7.4 Hz, 3H), 3.87 (q, *J* = 6.6 Hz, 1H), 2.71 – 2.49 (m, 3H), 2.13 – 1.88 (m, 1H), 1.80 (p, *J* = 7.4 Hz, 2H), 1.35 (d, *J* = 6.6 Hz, 3H).^{13}C NMR (126 MHz, Chloroform-*d*) δ 153.7, 141.9, 128.3, 128.3, 127.4, 125.8, 123.7, 58.0, 47.3, 33.5, 31.7, 24.4. $[\alpha]_D^{20}$ =-34.7 (c=1.0, EtOH). Enantiomeric excess was determined by chiral HPLC after the product was converted to the corresponding trifluoroacetamide: IB-3, Hex/IPA = 90:10, 1 mL/min, 220 nm, 11.7 min, 11.4 min.

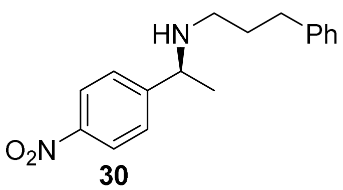

30

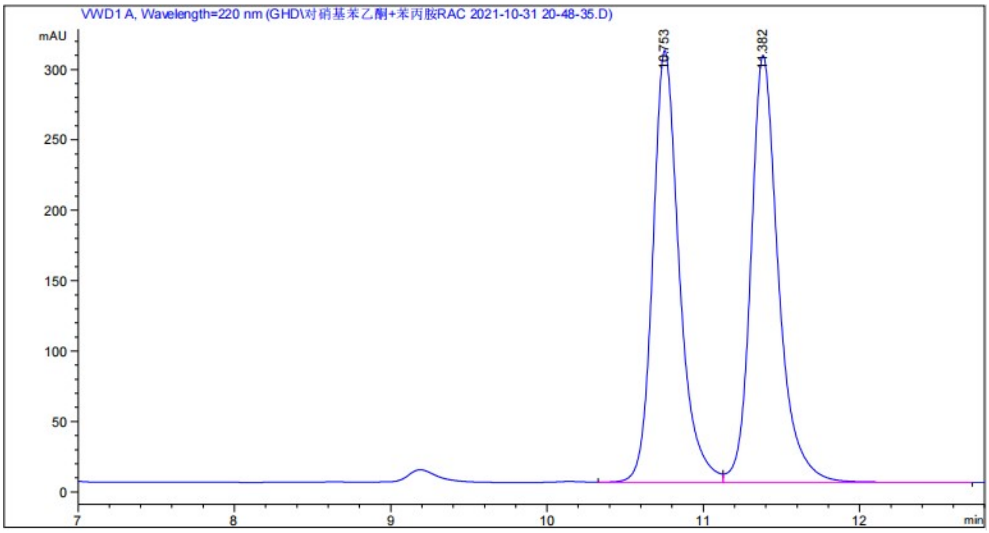

峰<br>\#	保留时间<br>[min]	类型	峰宽<br>[min]	峰面积<br>[mAU*s]	峰高<br>[mAU]	峰面积<br>%
1	10.753	VV	0.1774	3611.03125	306.72250	49.2489
2	11.382	VB	0.1851	3721.17188	303.37482	50.7511

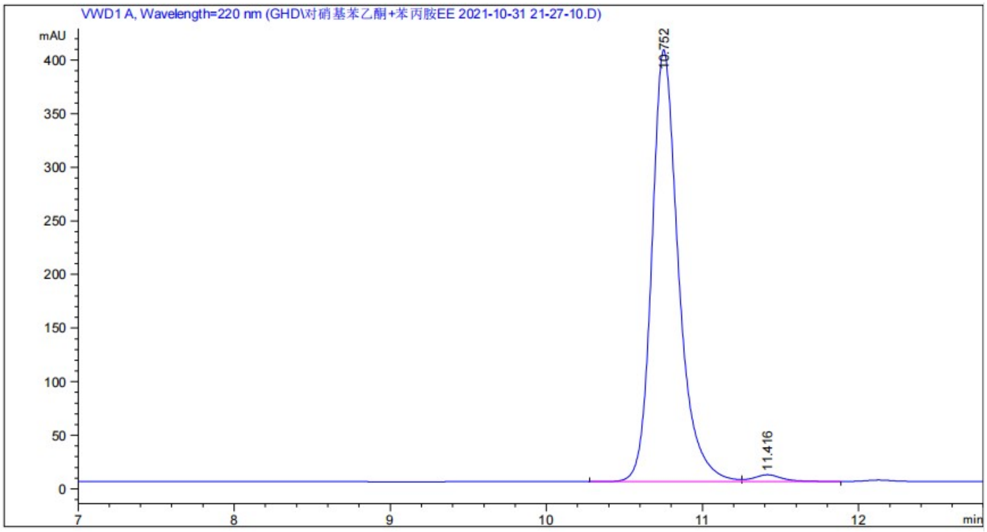

峰<br>\#	保留时间<br>[min]	类型	峰宽<br>[min]	峰面积<br>[mAU*s]	峰高<br>[mAU]	峰面积<br>%
1	10.752	BV	0.1791	4801.77783	402.71210	98.2666
2	11.416	VV	0.1939	84.70185	6.42277	1.7334

Supplementary Figure 29. HPLC spectra for racemic and chiral **30**.

N-(4-(1-((3-phenylpropyl)amino)ethyl)phenyl)pivalamide (31): 94% yield,95% ee, brown oil. ^{1}H NMR (400 MHz, Chloroform-*d*) δ 7.28 – 7.01 (m, 9H), 6.51 (s, 1H), 3.62 (q, *J* = 6.6 Hz, 1H), 2.61 – 2.32 (m, 4H), 1.69 (td, *J* = 7.7, 3.4 Hz, 2H), 1.52 (s, 1H), 1.44 (d, *J* = 1.1 Hz, 9H), 1.23 (d, *J* = 6.5 Hz, 3H). ^{13}C NMR (101 MHz, $CDCl_3$) δ 152.93, 142.22, 140.41, 137.06, 128.37, 128.32, 127.15, 125.73, 118.77, 57.77, 47.31, 33.70, 31.86, 28.38, 24.26. $[\alpha]_D^{20}$ =-41.3 (c=1.0, EtOH). Enantiomeric excess was determined by chiral HPLC after the product was converted to the corresponding trifluoroacetamide: IB-3, Hex/IPA = 90:10, 1 mL/min, 220 nm, 8.7 min, 10.7 min.

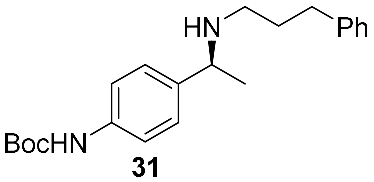

31

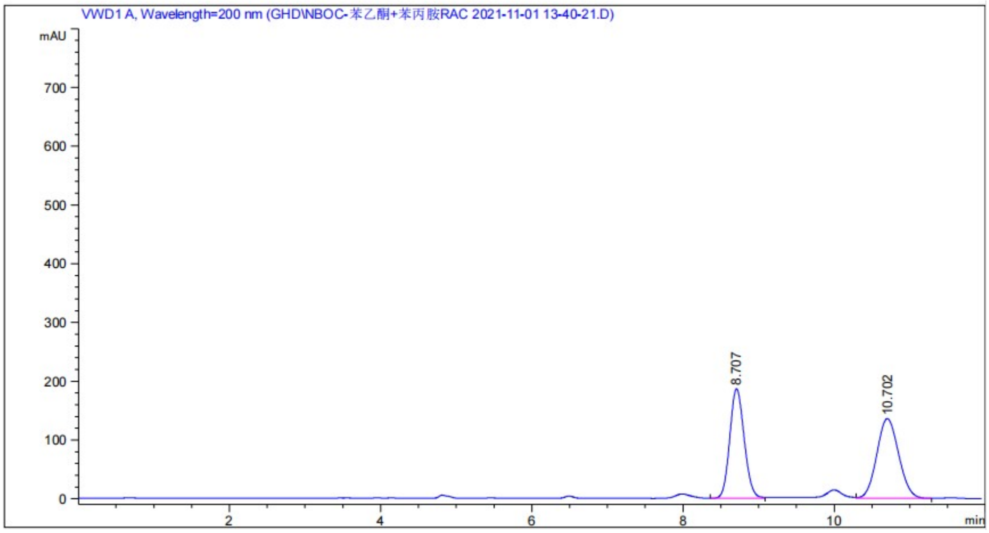

峰 #	保留时间 [min]	类型	峰宽 [min]	峰面积 [mAU*s]	峰高 [mAU]	峰面积 %
1	8.707	BV	0.2071	2477.50024	186.34790	47.5675
2	10.702	VB	0.3128	2730.88550	135.71867	52.4325

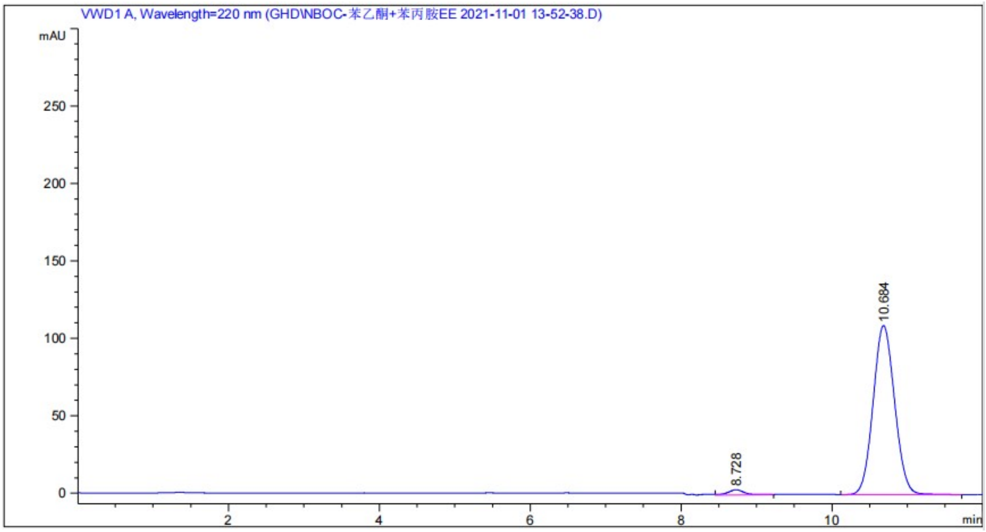

峰 #	保留时间 [min]	类型	峰宽 [min]	峰面积 [mAU*s]	峰高 [mAU]	峰面积 %
1	8.728	VV	0.2331	52.59563	3.35083	2.3736
2	10.684	BB	0.3093	2163.29468	109.13593	97.6264

Supplementary Figure 30. HPLC spectra for racemic and chiral **31**.

N-benzyl-4-(1-(benzylamino)ethyl)aniline (32): 94% yield, 95% ee, brown oil. ^{1}H NMR (400 MHz, Chloroform-*d*) δ 7.46 – 7.34 (m, 4H), 7.34 – 7.19 (m, *J* = 6.8 Hz, 8H), 6.95 (d, *J* = 8.2 Hz, 2H), 5.04 (s, 2H), 3.76 (q, *J* = 6.6 Hz, 1H), 3.69 – 3.53 (m, 2H), 1.61 (s, 1H), 1.34 (d, *J* = 6.6 Hz, 3H). ^{13}C NMR (101 MHz, $CDCl_3$) δ 157.87, 140.72, 137.95, 137.21, 128.61, 128.40, 128.17, 127.96, 127.79, 127.55, 126.86, 114.79, 70.10, 56.84, 51.64, 24.52. $[\alpha]_D^{20}$ =-38.9 (c=1.0, EtOH). Enantiomeric excess was determined by chiral HPLC after the product was converted to the corresponding trifluoroacetamide: AD-H, Hex/IPA = 90:10, 1 mL/min, 220 nm, 7.7 min, 8.1 min.

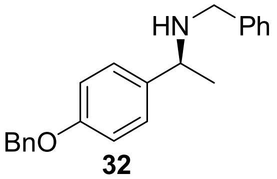

32

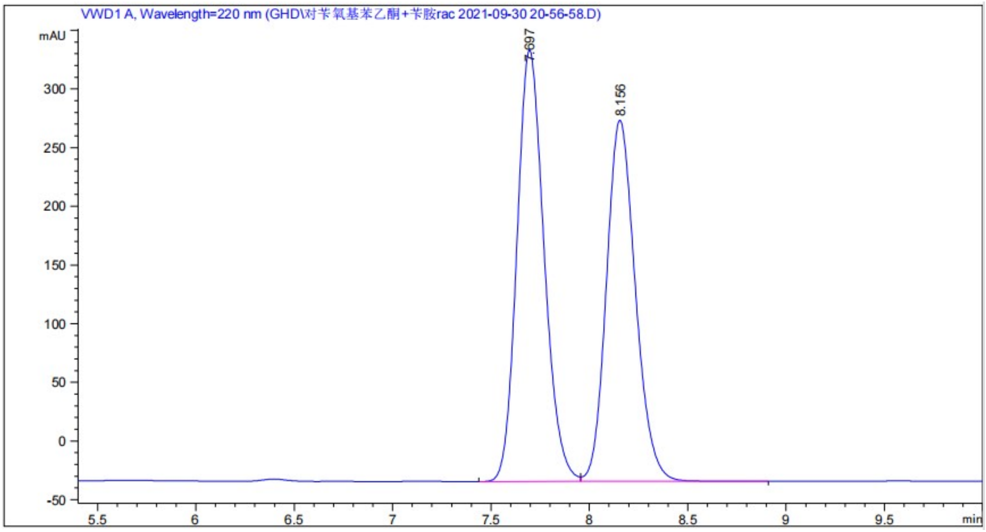

峰 #	保留时间 [min]	类型	峰宽 [min]	峰面积 [mAU*s]	峰高 [mAU]	峰面积 %
1	7.697	BV	0.1481	3532.38159	367.49106	52.8348
2	8.156	VB	0.1595	3153.32422	307.76837	47.1652

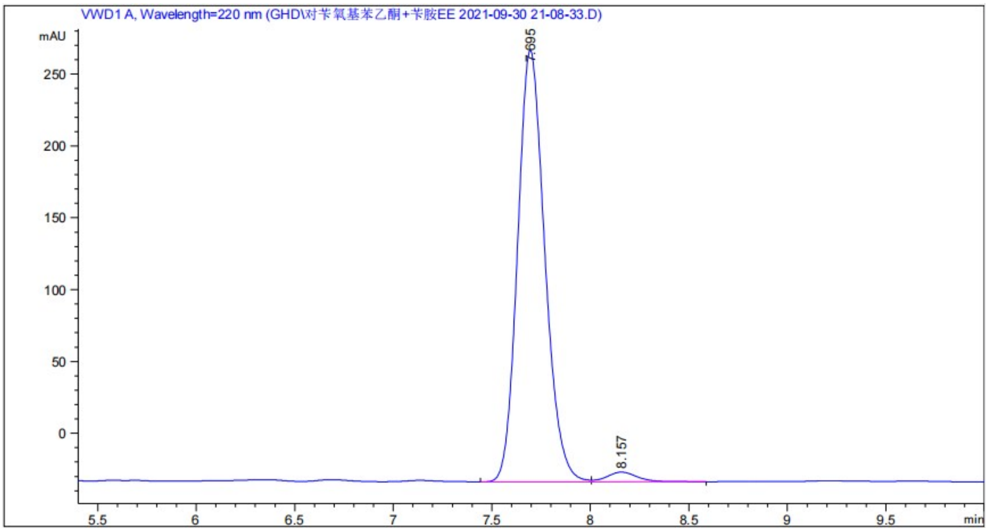

峰 #	保留时间 [min]	类型	峰宽 [min]	峰面积 [mAU*s]	峰高 [mAU]	峰面积 %
1	7.695	BV	0.1477	2874.59814	300.25116	97.6021
2	8.157	VB	0.1608	70.62242	6.70696	2.3979

Supplementary Figure 31. HPLC spectra for racemic and chiral **32**.

N-benzyl-1-(4-(methylthio)phenyl)ethan-1-amine (33): 94% yield, 92% ee, colorless oil. ^{1}H NMR (400 MHz, Chloroform-*d*) δ 7.36 – 7.16 (m, 9H), 3.77 (q, *J* = 6.6 Hz, 1H), 3.68 – 3.53 (m, 2H), 2.47 (s, 3H), 1.77 (s, 1H), 1.34 (d, *J* = 6.6 Hz, 3H). ^{13}C NMR (101 MHz, $CDCl_3$) δ 142.63, 140.53, 136.60, 128.40, 128.15, 127.33, 127.00, 126.91, 57.04, 51.62, 24.47, 16.13. $[\alpha]_D^{20}$ =-63.4 (c=0.5, EtOH). Enantiomeric excess was determined by chiral HPLC after the product was converted to the corresponding trifluoroacetamide: AD-H, Hex/IPA = 90:10, 1 mL/min, 220 nm, 7.2 min, 8.7 min.

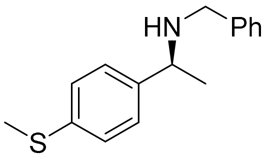

33

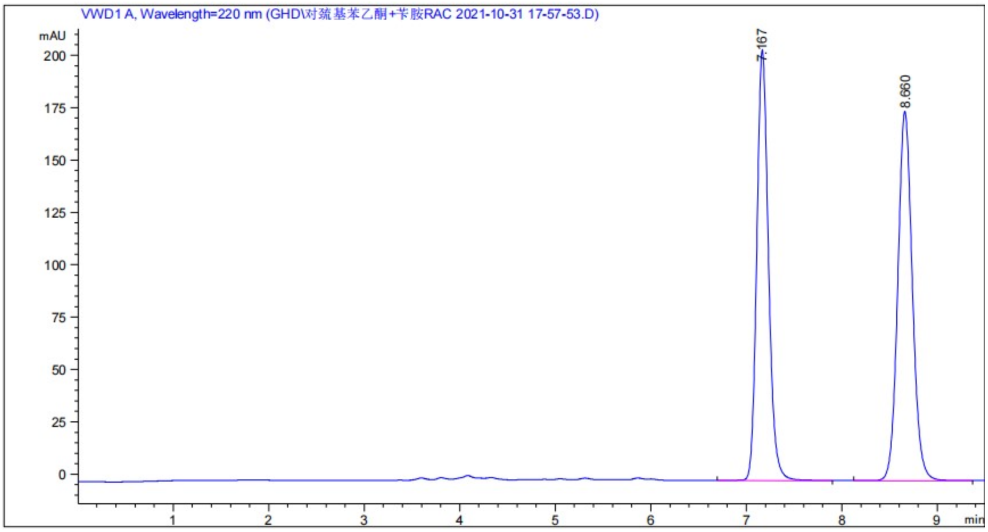

峰 #	保留时间 [min]	类型	峰宽 [min]	峰面积 [mAU*s]	峰高 [mAU]	峰面积 %
1	7.167	BV	0.1299	1742.05420	205.57968	48.1905
2	8.660	VV	0.1640	1872.87976	176.22234	51.8095

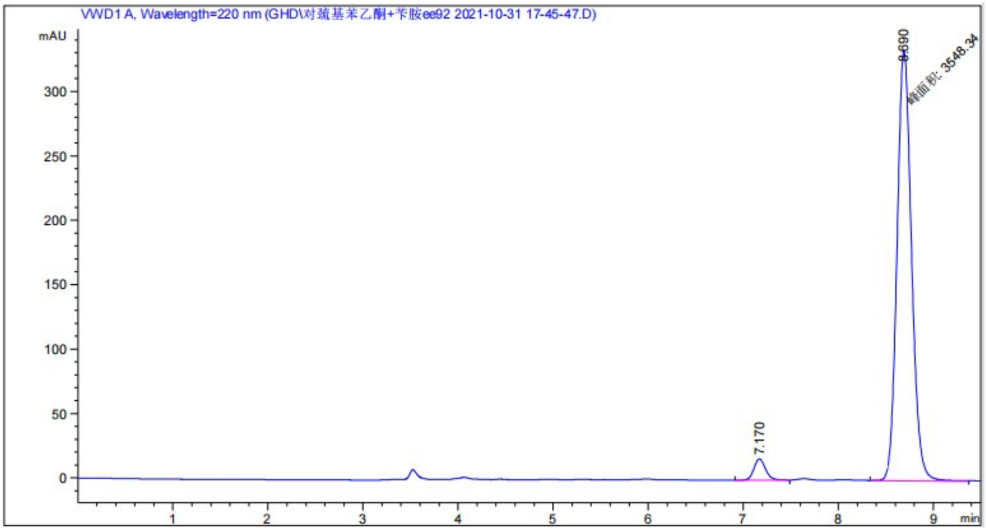

峰 #	保留时间 [min]	类型	峰宽 [min]	峰面积 [mAU*s]	峰高 [mAU]	峰面积 %
1	7.170	BV	0.1330	143.32539	16.55205	3.8824
2	8.690	MF	0.1769	3548.34326	334.34564	96.1176

Supplementary Figure 32. HPLC spectra for racemic and chiral **33**.

3-phenyl-N-(1-(4-(4,4,5,5-tetramethyl-1,3,2-dioxaborolan-2-yl)phenyl)ethyl)propan-1-amine (34): 96% yield, 97% ee,

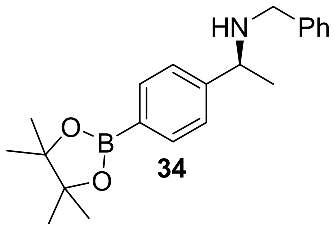

34

brown oil. ^{1}H NMR (400 MHz, Chloroform-*d*) δ 7.81 (d, *J* = 7.6 Hz, 2H), 7.37 (d, *J* = 7.7 Hz, 2H), 7.34 – 7.19 (m, 5H), 3.82 (q, *J* = 6.7 Hz, 1H), 3.68 – 3.52 (m, 2H), 1.72 (s, 1H), 1.35 (d, *J* = 7.8 Hz, 15H). ^{13}C NMR (101 MHz, $CDCl_3$) δ 148.89, 140.51, 135.11, 128.41, 128.20, 126.91, 126.19, 83.74, 57.60, 51.65, 24.91, 24.47. $[\alpha]_D^{20}$ =-48.3 (c=1.0, EtOH). Enantiomeric excess was determined by chiral HPLC after the product was converted to the corresponding trifluoroacetamide: Chiralpak IB-3 column, Hex/IPA = 90:10, 1 mL/min, 220 nm, 7.5 min, 7.9 min.

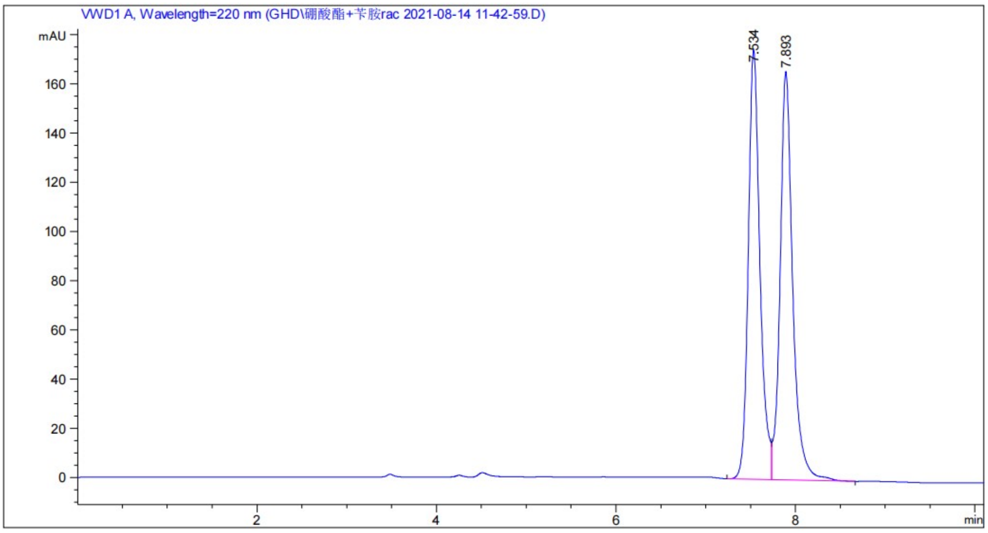

峰 #	保留时间 [min]	类型	峰宽 [min]	峰面积 [mAU*s]	峰高 [mAU]	峰面积 %
1	7.534	BV	0.1351	1570.29639	174.30858	49.2399
2	7.893	VB	0.1458	1618.77747	165.92351	50.7601

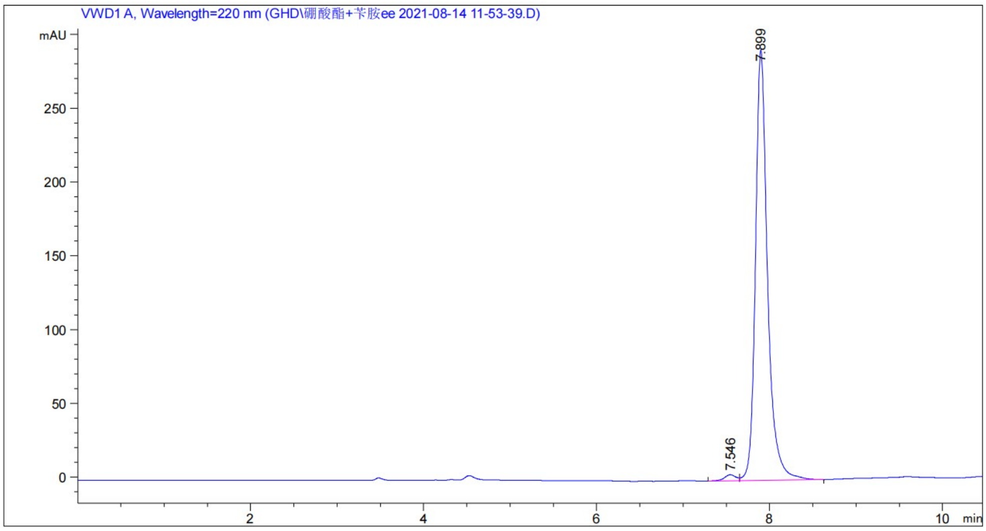

峰 #	保留时间 [min]	类型	峰宽 [min]	峰面积 [mAU*s]	峰高 [mAU]	峰面积 %
1	7.546	BV	0.1379	38.12642	4.12158	1.3312
2	7.899	VB	0.1450	2825.96680	291.63834	98.6688

Supplementary Figure 33. HPLC spectra for racemic and chiral **34**.

methyl 4-(1-(benzylamino)ethyl)benzoate (35): 94% yield, 92% ee, colorless oil. ^{1}H NMR (400 MHz, Chloroform-*d*) δ 8.06 – 7.99 (m, 1H), 7.44 (d, *J* = 8.2 Hz, 1H), 7.34 – 7.21 (m, 2H), 3.94 – 3.82 (m, 2H), 3.60 (q, *J* = 13.1 Hz, 1H), 1.36 (d, *J* = 6.6 Hz, 2H). ^{13}C NMR (101 MHz, $CDCl_3$) δ 167.10, 151.08, 140.33, 129.91, 128.44, 128.12, 127.00, 126.76, 57.36, 52.04, 51.70, 29.72, 24.44. $[\alpha]_D^{20}$ =-39.2 (c=1.0, EtOH). Enantiomeric excess was determined by chiral HPLC after the product was converted to the corresponding benzamide: IB-3, Hex/IPA = 90:10, 1mL/min, 220 nm, 8.3 min, 8.6 min.

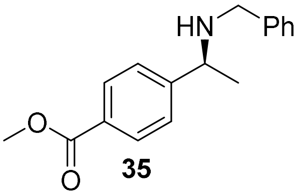

35

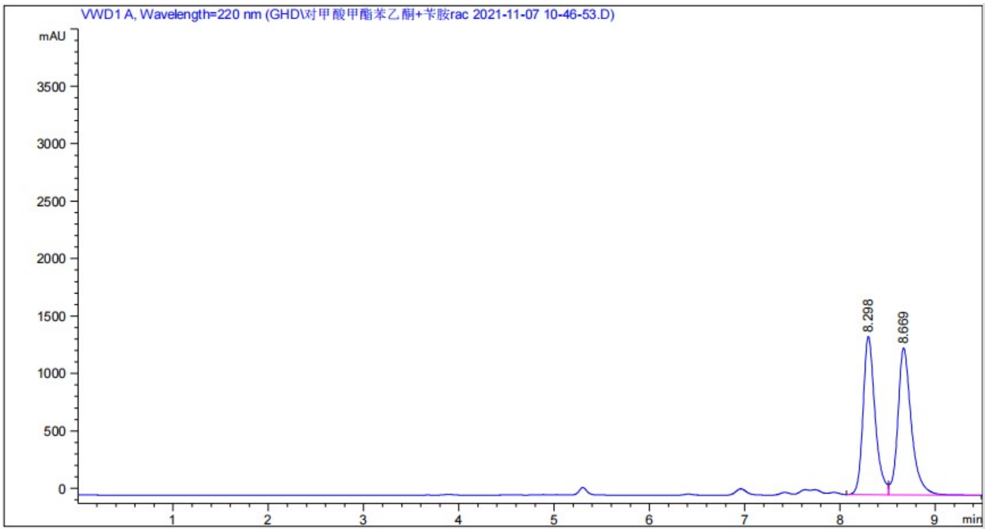

峰 #	保留时间 [min]	类型	峰宽 [min]	峰面积 [mAU*s]	峰高 [mAU]	峰面积 %
1	8.298	BV	0.1324	1.21022e4	1378.66492	49.0756
2	8.669	VB	0.1467	1.25581e4	1277.23035	50.9244

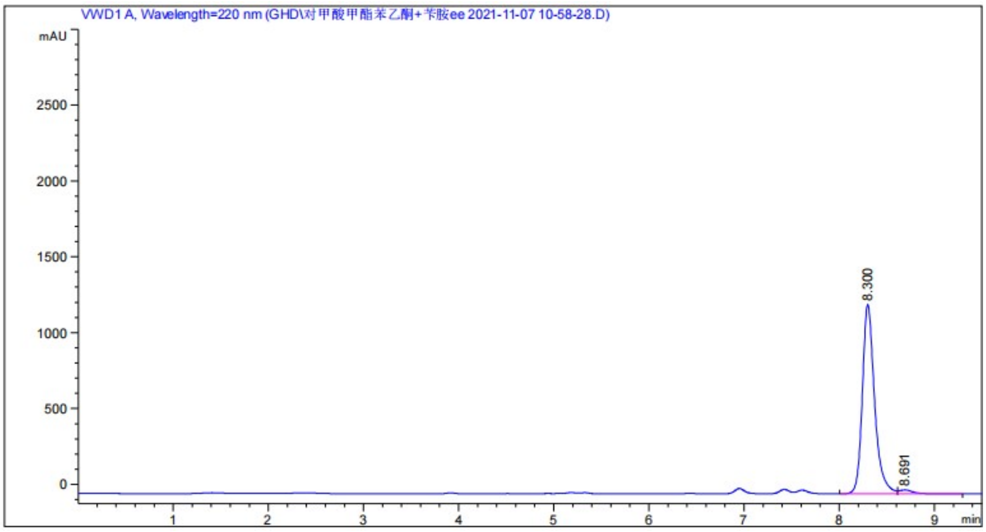

峰 #	保留时间 [min]	类型	峰宽 [min]	峰面积 [mAU*s]	峰高 [mAU]	峰面积 %
1	8.300	BV	0.1344	1.11605e4	1247.19373	97.6486
2	8.691	VB	0.1491	268.74731	26.31872	2.3514

Supplementary Figure 34. HPLC spectra for racemic and chiral **35**.

3-phenyl-N-(1-(thiophen-2-yl)ethyl)propan-1-amine (36):[4] 94% yield, 93% ee, colorless oil. ^{1}H NMR (500 MHz, Chloroform-*d*) δ 7.36 – 7.29 (m, 2H), 7.27 – 7.18 (m, 4H), 6.99 (dd, *J* = 5.1, 3.4 Hz, 1H), 6.94 (d, *J* = 3.1 Hz, 1H), 4.12 (q, *J* = 6.5 Hz, 1H), 2.78 – 2.57 (m, 4H), 1.94 – 1.77 (m, 2H), 1.58 – 1.52 (m, 1H), 1.50 (d, *J* = 6.6 Hz, 3H). ^{13}C NMR (126 MHz, Chloroform-*d*) δ 150.9, 142.2, 128.3, 126.3, 125.7, 123.5, 123.2, 53.7, 47.1, 33.6, 31.8, 24.8. $[\alpha]_D^{20}$ =-41.0 (c=0.5, EtOH). Enantiomeric excess was determined by chiral HPLC after the product was converted to the corresponding acetamide: AD-H, Hex/IPA = 90:10, 1 mL/min, 220 nm, 8.2 min, 8.9 min.

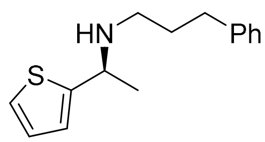

36

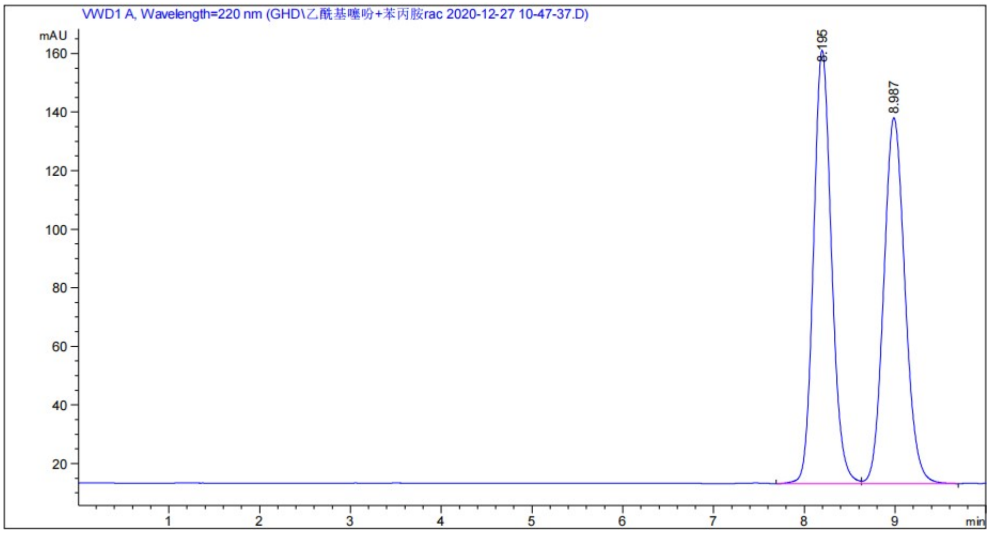

峰 #	保留时间 [min]	类型	峰宽 [min]	峰面积 [mAU*s]	峰高 [mAU]	峰面积 %
1	8.195	BV	0.2127	2037.20142	147.93654	50.0786
2	8.987	VB	0.2517	2030.81030	124.87027	49.9214

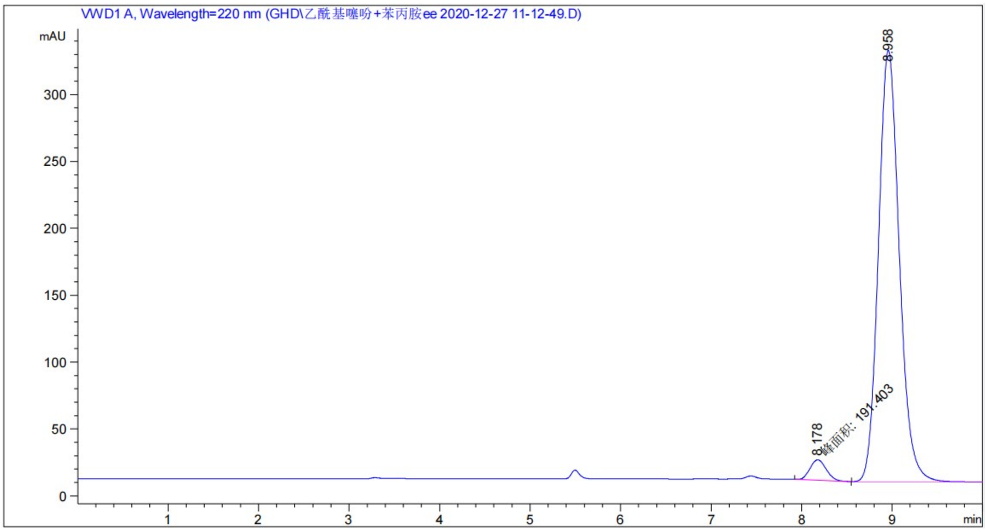

峰 #	保留时间 [min]	类型	峰宽 [min]	峰面积 [mAU*s]	峰高 [mAU]	峰面积 %
1	8.178	MM	0.2085	191.40323	15.30070	3.5705
2	8.958	BB	0.2470	5169.25000	322.47556	96.4295

Supplementary Figure 35. HPLC spectra for racemic and chiral **36**.

N-phenethyl-1-phenylethan-1-amine (37):[6] 95% yield, 97% ee, brown oil. ^{1}H NMR (500 MHz, Chloroform-*d*) δ 7.41 – 7.16 (m, 10H), 3.83 (q, *J* = 6.6 Hz, 1H), 2.90 – 2.71 (m, 4H), 1.71 (s, 1H), 1.39 (d, *J* = 6.6 Hz, 3H). ^{13}C NMR (126 MHz, Chloroform-*d*) δ 128.7, 128.4, 128.4, 126.9, 126.5, 126.1, 58.2, 48.92 , 36.4, 24.2. $[\alpha]_D^{20}$ =-29.0 (c=1.0, EtOH). Enantiomeric excess was determined by chiral HPLC after the product was converted to the corresponding acetamide: Chiralpak AD-H column, Hex/IPA = 90:10, 1 mL/min, 220 nm, 8.8 min, 9.7 min.

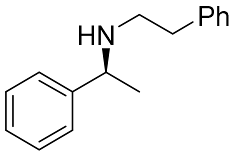

37

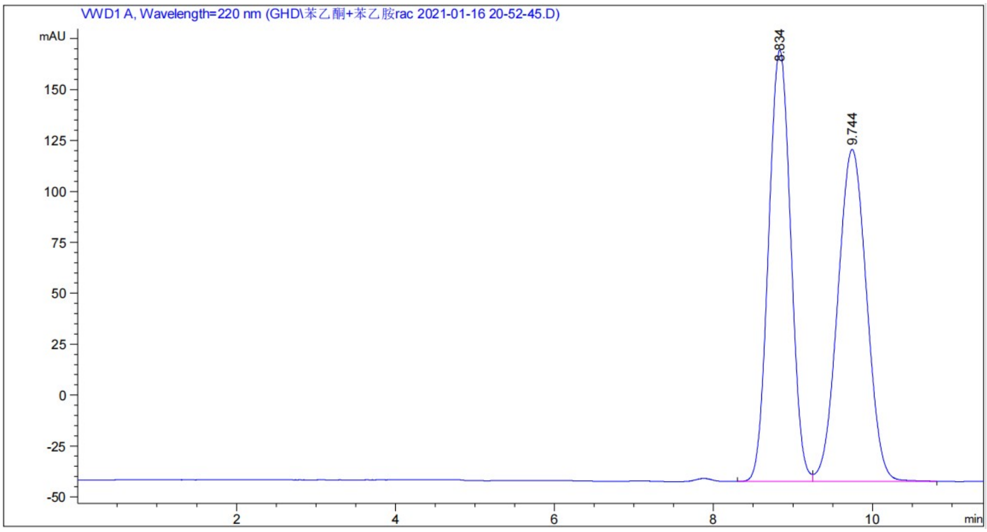

峰 #	保留时间 [min]	类型	峰宽 [min]	峰面积 [mAU*s]	峰高 [mAU]	峰面积 %
1	8.834	BV	0.3075	4093.11694	211.78670	49.8946
2	9.744	VB	0.3982	4110.40234	163.02216	50.1054

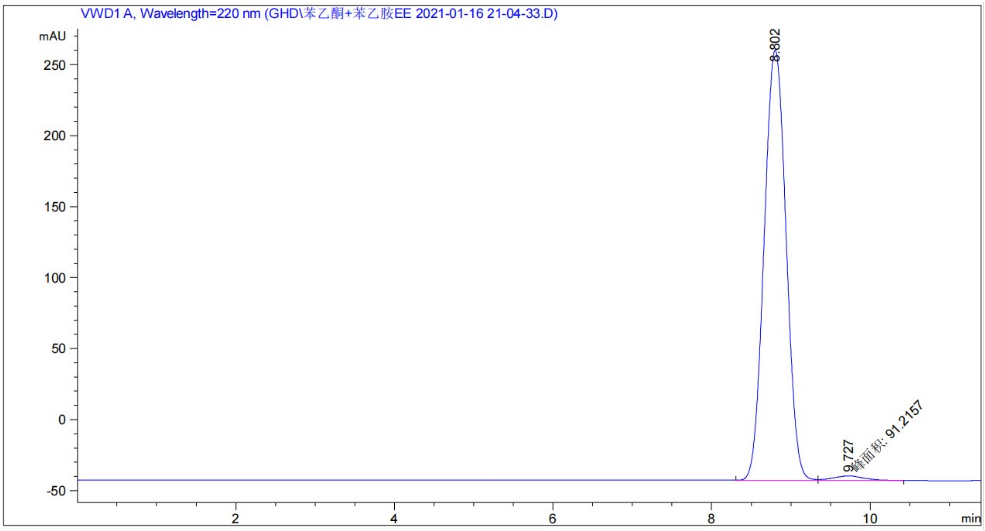

峰 #	保留时间 [min]	类型	峰宽 [min]	峰面积 [mAU*s]	峰高 [mAU]	峰面积 %
1	8.802	VV	0.3046	5808.85986	303.05508	98.4540
2	9.727	MF	0.4565	91.21573	3.33051	1.5460

Supplementary Figure 36. HPLC spectra for racemic and chiral **37**.

N-benzyl-1-phenylethan-1-amine(38):[6] 95% yield, 97% ee, brown oil. ^{1}H NMR (400 MHz, Chloroform-*d*) δ 7.40 – 7.17 (m, 10H), 3.79 (t, J = 6.4 Hz, 1H), 3.70 – 3.53 (m, 2H), 1.65 (s, 1H), 1.44 – 1.31 (m, 3H). $[\alpha]_D^{20}$ =-23.9 (c=1.0, EtOH).Enantiomeric excess was determined by chiral HPLC after the product was converted to the corresponding acetamide: Chiralpak AD-H column, Hex/IPA = 90:10, 1 mL/min, 220 nm, 10.2 min, 11.9 min.

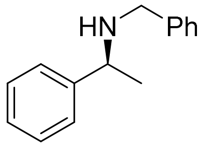

38

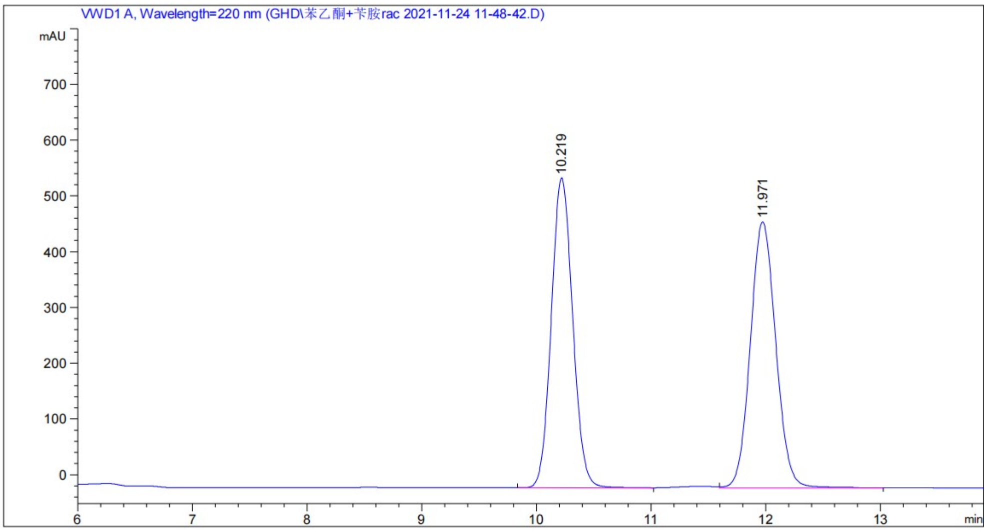

峰 #	保留时间 [min]	类型	峰宽 [min]	峰面积 [mAU*s]	峰高 [mAU]	峰面积 %
1	10.219	VB	0.2009	7183.79736	555.42834	49.4674
2	11.971	VV	0.2397	7338.49072	476.33173	50.5326

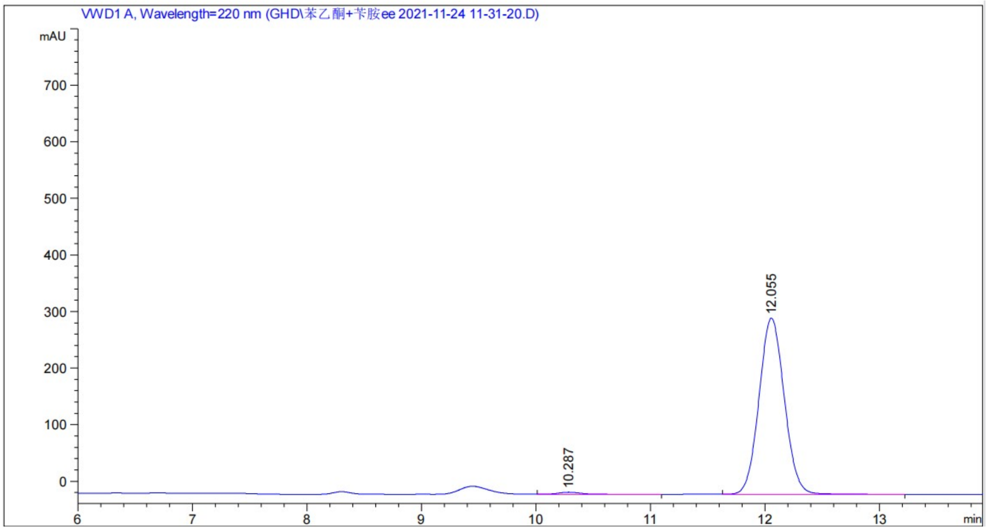

峰 #	保留时间 [min]	类型	峰宽 [min]	峰面积 [mAU*s]	峰高 [mAU]	峰面积 %
1	10.287	VB	0.2321	59.83847	3.83270	1.2331
2	12.055	VV	0.2395	4792.76904	311.58923	98.7669

Supplementary Figure 37. HPLC spectra for racemic and chiral **38**.

N-(1-phenylethyl)aniline (39):[3] 94% yield, 98% ee, brown oil. ^{1}H NMR (400 MHz, Chloroform-*d*) δ 7.40 – 7.23 (m, 4H), 7.23 – 7.13 (m, 1H), 7.12 – 7.02 (m, 2H), 6.68 – 6.57 (m, 1H), 6.48 (d, J = 7.9 Hz, 2H), 4.45 (q, J = 6.7 Hz, 1H), 1.47 (d, J = 6.8 Hz, 3H). $[\alpha]_D^{20}$ =-21.3 (c=1.0, EtOH). Enantiomeric excess was determined by chiral HPLC after the product was converted to the corresponding acetamide: Chiralpak IB-3 column, Hex/IPA = 90:10, 1 mL/min, 220 nm, 8.0 min, 10.2 min.

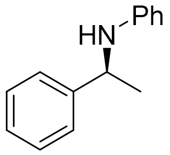

39

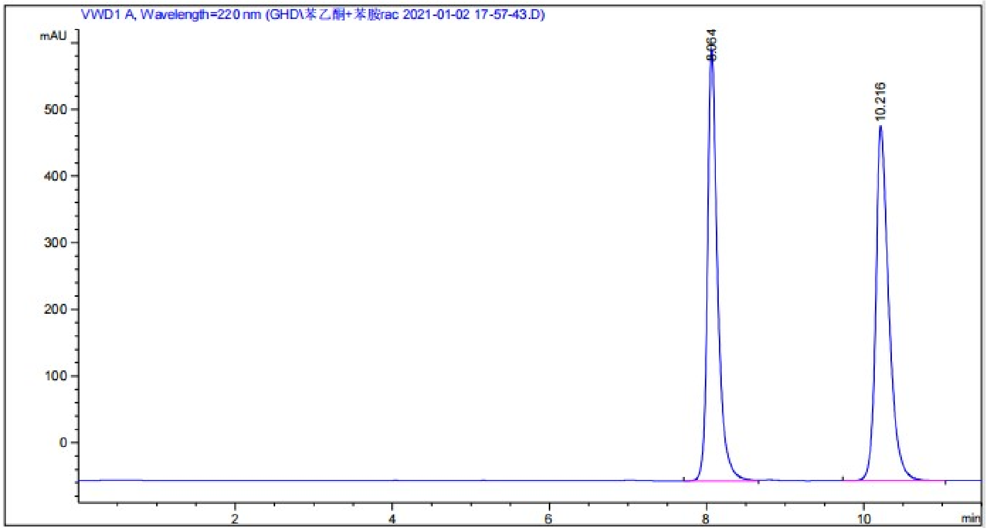

峰 #	保留时间 [min]	类型	峰宽 [min]	峰面积 [mAU*s]	峰高 [mAU]	峰面积 %
1	8.064	BV	0.1322	5767.57617	645.90509	48.4146
2	10.216	VV	0.1728	6145.31592	531.91138	51.5854

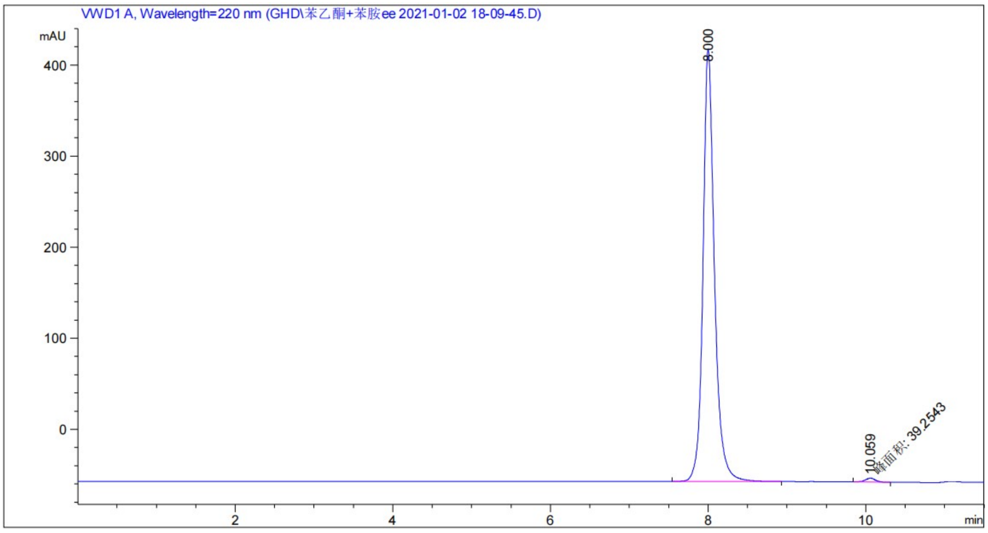

峰 #	保留时间 [min]	类型	峰宽 [min]	峰面积 [mAU*s]	峰高 [mAU]	峰面积 %
1	8.000	BB	0.1508	4785.58838	473.82346	99.1864
2	10.059	FM	0.1521	39.25426	4.30139	0.8136

Supplementary Figure 38. HPLC spectra for racemic and chiral **39**.

N-methyl-1-phenylethan-1-amine (40):[4] 95% yield, 95% ee, colorless oil. ^{1}H NMR (400 MHz, Chloroform-*d*) δ 7.36 – 7.26 (m, 5H), 3.64 (q, *J* = 6.6 Hz, 1H), 2.30 (s, 3H), 1.81 – 1.71 (m, 1H), 1.36 (d, *J* = 6.7 Hz, 3H). $[\alpha]_D^{20}$ =-32.7 (c=1.0, EtOH). Enantiomeric excess was determined by chiral HPLC after the product was converted to the corresponding acetamide: Chiralpak AS-H column, Hex/IPA = 90:10, 1 mL/min, 220 nm, 7.0 min, 7.7 min.

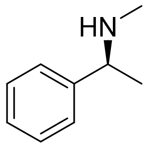

40

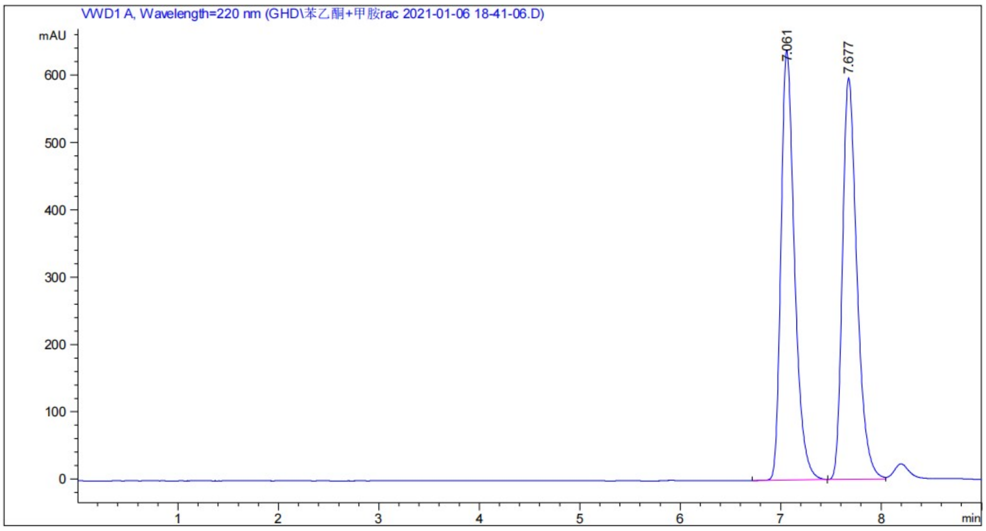

峰 #	保留时间 [min]	类型	峰宽 [min]	峰面积 [mAU*s]	峰高 [mAU]	峰面积 %
1	7.061	BB	0.1433	5978.30225	638.18549	49.9541
2	7.677	BV	0.1532	5989.28320	595.97443	50.0459

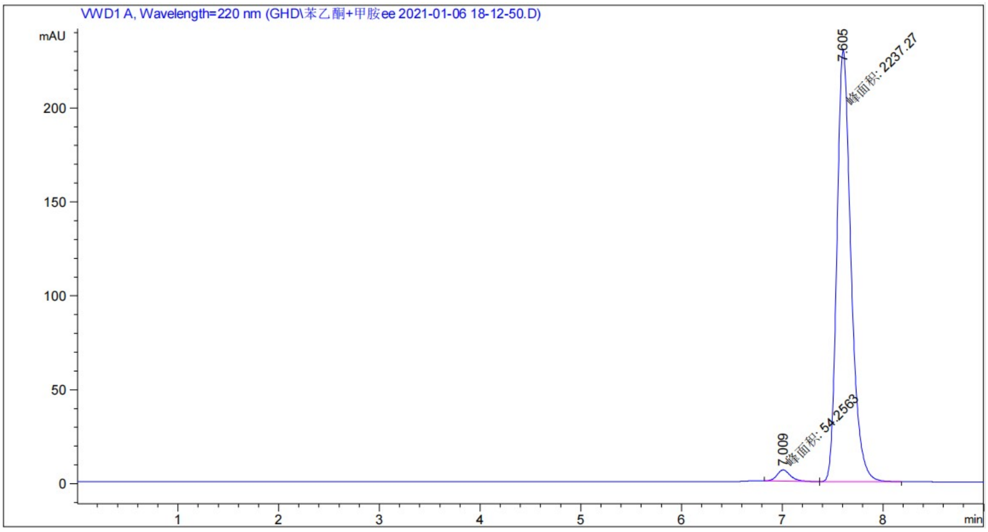

峰 #	保留时间 [min]	类型	峰宽 [min]	峰面积 [mAU*s]	峰高 [mAU]	峰面积 %
1	7.009	MM	0.1523	54.25631	5.93693	2.3677
2	7.605	MM	0.1622	2237.26782	229.81935	97.6323

Supplementary Figure 39. HPLC spectra for racemic and chiral **40**.

N-(1-phenylethyl)propan-1-amine (41):[7] 97% yield, 98% ee, brown oil. ^{1}H NMR (400 MHz, Chloroform-*d*) δ 7.37 – 7.18 (m, 5H), 3.76 (q, J = 6.6 Hz, 1H), 2.42 (dddd, J = 34.0, 11.3, 8.2, 6.3 Hz, 2H), 1.60 – 1.39 (m, 4H), 1.35 (d, J = 6.6 Hz, 3H), 0.87 (t, J = 7.4 Hz, 3H). $[\alpha]_D^{20}$ =+31.8 (c=1.0, EtOH). Enantiomeric excess was determined by chiral HPLC after the product was converted to the corresponding acetamide: Chiralpak IB-3 column, Hex/IPA = 90:10, 1 mL/min, 220 nm, 7.1 min, 7.9 min.

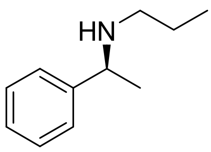

41

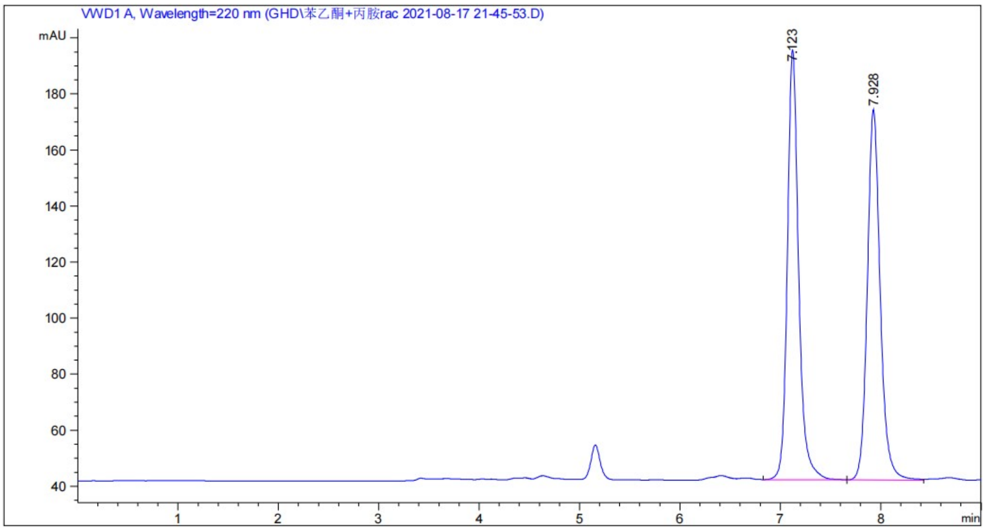

峰 #	保留时间 [min]	类型	峰宽 [min]	峰面积 [mAU*s]	峰高 [mAU]	峰面积 %
1	7.123	BB	0.1179	1194.29541	153.31541	50.3598
2	7.928	BV	0.1349	1177.22864	132.13121	49.6402

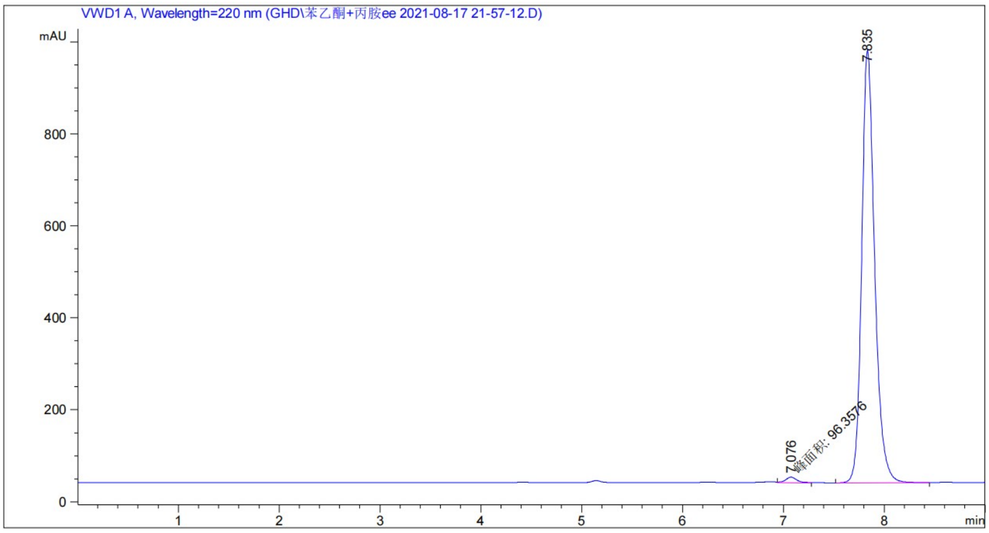

峰 #	保留时间 [min]	类型	峰宽 [min]	峰面积 [mAU*s]	峰高 [mAU]	峰面积 %
1	7.076	MF	0.1295	96.35764	12.39794	1.1349
2	7.835	BV	0.1351	8394.14844	940.90240	98.8651

Supplementary Figure 40. HPLC spectra for racemic and chiral **41**.

N-(1-phenylethyl)butan-1-amine (42):[3] 96% yield, 97% ee, brown oil. ^{1}H NMR (400 MHz, Chloroform-*d*) δ 7.36 – 7.17 (m, 5H), 3.75 (q, J = 6.6 Hz, 1H), 2.54 – 2.36 (m, 2H), 1.53 – 1.38 (m, 3H), 1.38 – 1.23 (m, 5H), 0.87 (t, J = 7.3 Hz, 3H). $[\alpha]_D^{20}$ =+32.8 (c=1.0, EtOH). Enantiomeric excess was determined by chiral HPLC after the product was converted to the corresponding acetamide: Chiralpak AD-H column, Hex/IPA = 90:10, 1 mL/min, 220 nm, 6.0 min, 6.4 min.

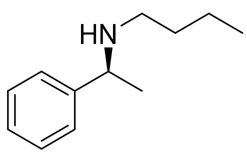

42

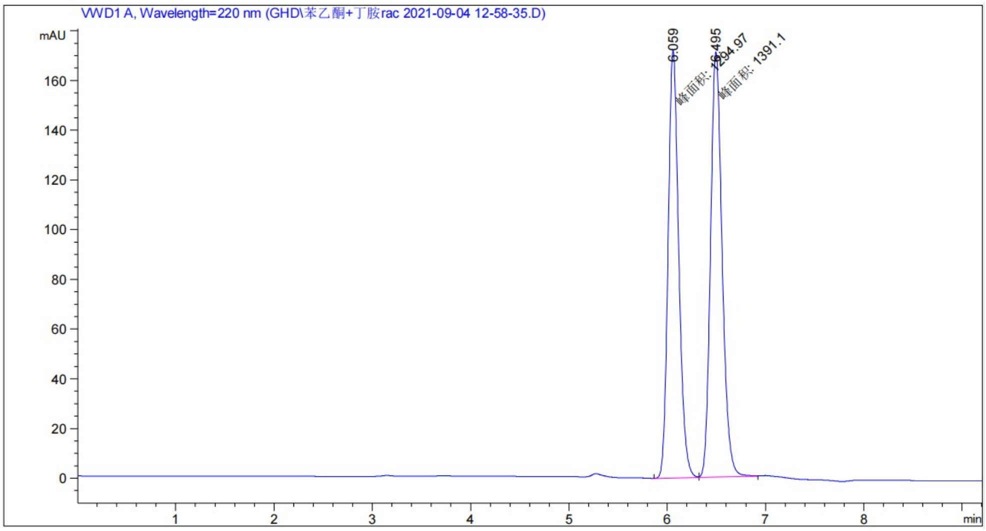

峰 #	保留时间 [min]	类型	峰宽 [min]	峰面积 [mAU*s]	峰高 [mAU]	峰面积 %
1	6.059	MF	0.1253	1294.96753	172.28510	48.2106
2	6.495	FM	0.1355	1391.09851	171.13409	51.7894

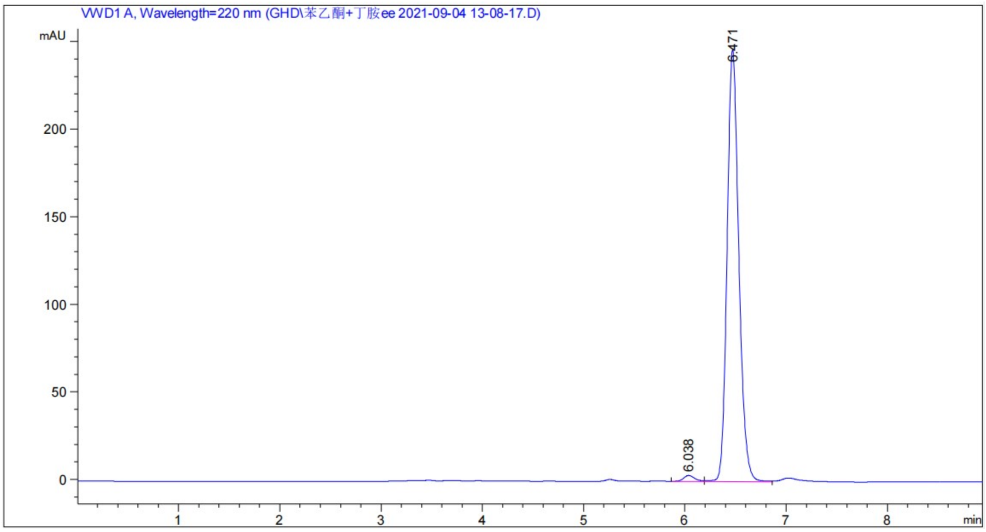

峰 #	保留时间 [min]	类型	峰宽 [min]	峰面积 [mAU*s]	峰高 [mAU]	峰面积 %
1	6.038	BV	0.1169	26.23280	3.40278	1.3081
2	6.471	VV	0.1237	1979.22375	246.32550	98.6919

Supplementary Figure 41. HPLC spectra for racemic and chiral **42**.

(1-phenylethyl)hexan-1-amine (43):[3] 94% yield, 97% ee, brown oil. ^{1}H NMR (400 MHz, Chloroform-*d*) δ 7.31 (d, *J* = 5.8 Hz, 4H), 7.25 (s, 1H), 3.75 (q, *J* = 6.6 Hz, 1H), 2.54 – 2.35 (m, 2H), 1.65 – 1.53 (m, 1H), 1.45 (d, *J* = 6.6 Hz, 2H), 1.40 – 1.16 (m, 10H), 0.86 (t, *J* = 6.8 Hz, 3H). $[\alpha]_D^{20}$ =+29.3 (c=1.0, EtOH). Enantiomeric excess was determined by chiral HPLC after the product was converted to the corresponding acetamide: Chiralpak IB-3 column, Hex/IPA = 90:10, 1 mL/min, 220 nm, 5.6 min, 6.1 min.

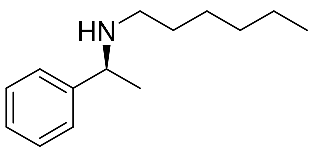

43

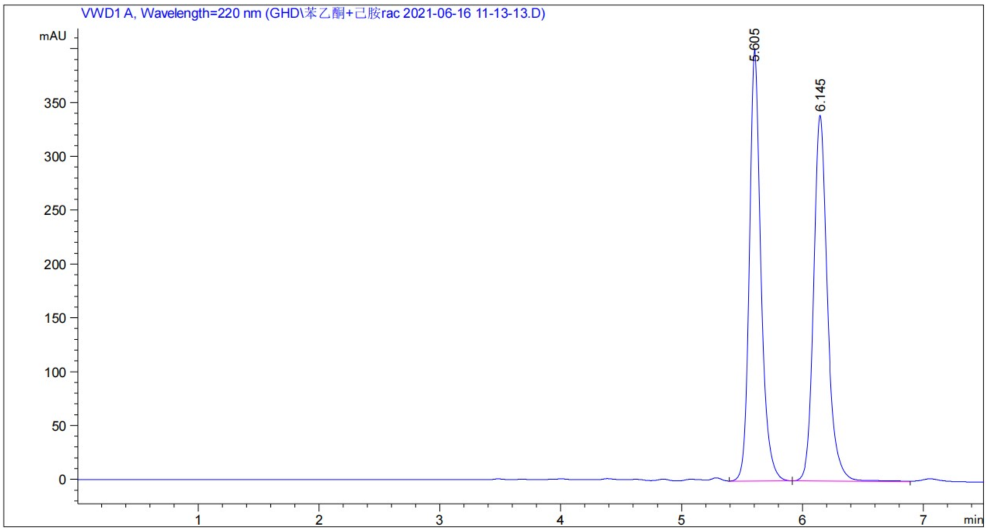

峰 #	保留时间 [min]	类型	峰宽 [min]	峰面积 [mAU*s]	峰高 [mAU]	峰面积 %
1	5.605	BB	0.1014	2692.12866	400.81000	49.9979
2	6.145	BV	0.1204	2692.35547	339.78415	50.0021

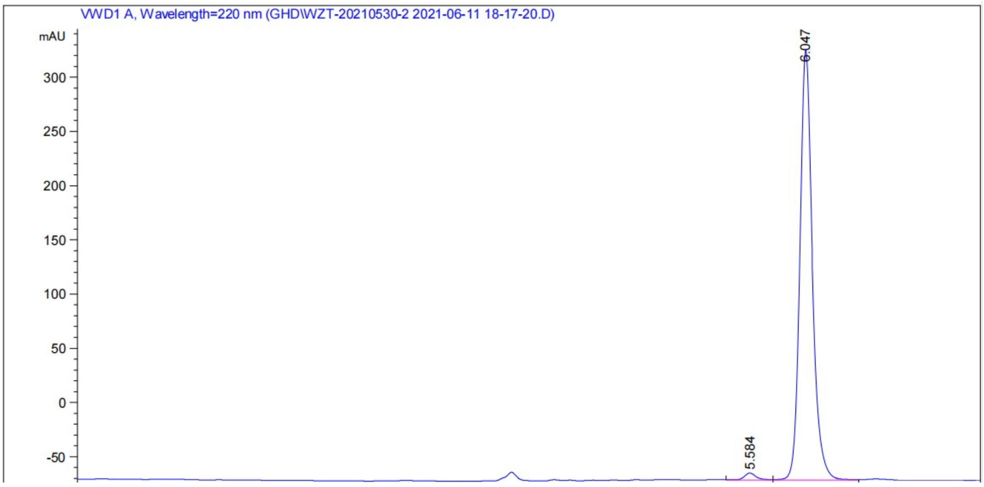

峰 #	保留时间 [min]	类型	峰宽 [min]	峰面积 [mAU*s]	峰高 [mAU]	峰面积 %
1	5.584	VV	0.1008	43.43737	6.43157	1.5041
2	6.047	VV	0.1098	2844.44800	396.12198	98.4959

Supplementary Figure 42. HPLC spectra for racemic and chiral **43**.

N-(cyclohexylmethyl)-1-phenylethan-1-amine (44):[6] 96% yield, 95% ee, brown oil. ^{1}H NMR (400 MHz, Chloroform-*d*) δ 7.53 – 7.15 (m, 5H), 3.96 (q, J = 6.6 Hz, 1H), 2.26 (tt, J = 10.1, 3.7 Hz, 1H), 2.04 – 1.90 (m, 1H), 1.77 – 1.61 (m, 3H), 1.55 (t, J = 4.8 Hz, 1H), 1.33 (d, J = 6.6 Hz, 3H), 1.22 – 0.93 (m, 5H). ^{13}C NMR (101 MHz, Chloroform-*d*) δ 146.3, 128.4, 126.6, 126.4, 54.4, 53.6, 34.5, 33.2, 26.19, 25.2, 25.0, 24.9. $[\alpha]_D^{20}$ =+20.8 (c=1.0, EtOH). Enantiomeric excess was determined by chiral HPLC after the product was converted to the corresponding acetamide: Chiralpak IB-3 column, Hex/IPA = 90:10, 1 mL/min, 220 nm, 6.4 min, 7.0 min.

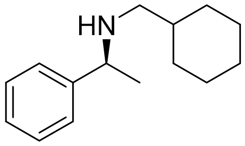

44

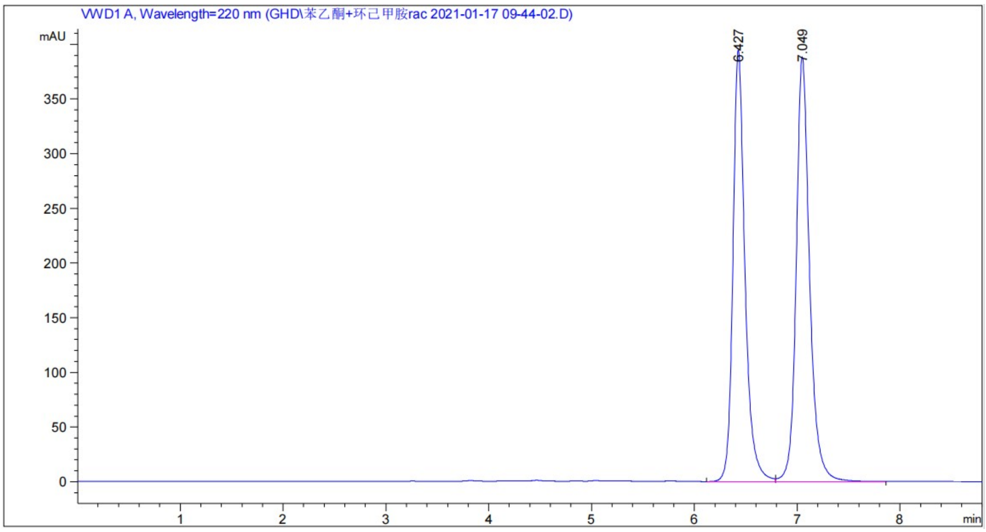

峰 #	保留时间 [min]	类型	峰宽 [min]	峰面积 [mAU*s]	峰高 [mAU]	峰面积 %
1	6.427	BV	0.1219	3208.65210	394.61450	47.7040
2	7.049	VV	0.1357	3517.52344	388.10599	52.2960

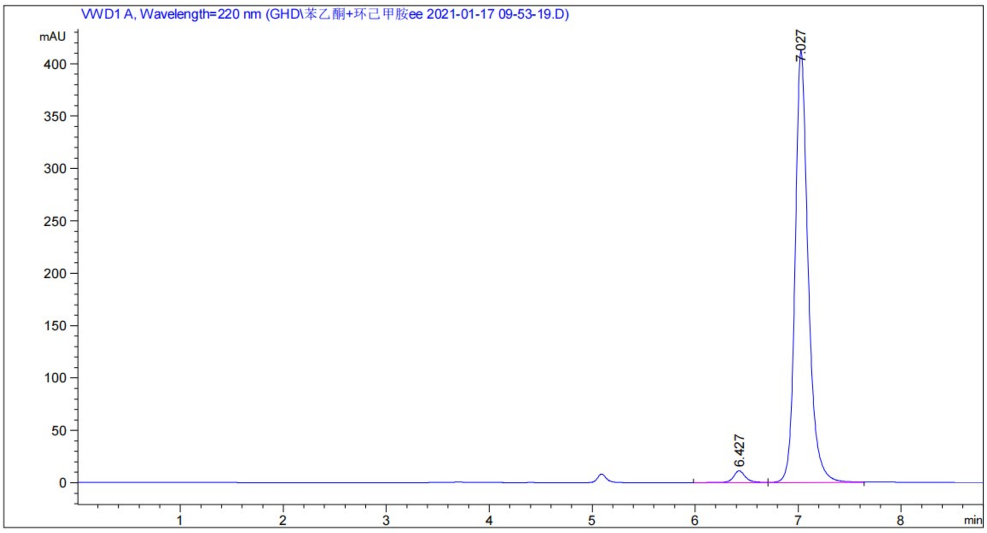

峰 #	保留时间 [min]	类型	峰宽 [min]	峰面积 [mAU*s]	峰高 [mAU]	峰面积 %
1	6.427	BV	0.1246	95.07542	11.36426	2.5465
2	7.027	VV	0.1329	3638.47070	412.59406	97.4535

Supplementary Figure 43. HPLC spectra for racemic and chiral **44**.

N-(cyclohexylmethyl)-1-(naphthalen-1-yl)ethan-1-amine (45): 95% yield, 97% ee, colorless oil. ^{1}H NMR (500 MHz, $CDCl_3$): δ 8.22 (d, *J* = 8.4 Hz, 1H), 7.91 (d, *J* = 7.8 Hz, 1H), 7.78 (d, *J* = 8.2 Hz, 1H), 7.72 (d, J = 7.1 Hz, 1H), 7.53 (m, 3H), 4.65 (q, *J* = 6.5 Hz, 1H), 2.55–2.36 (m, 2H), 1.90–1.10 (m, 13H), 0.93 (m, 2H); ^{13}C NMR (125 MHz, $CDCl_3$): δ 134.0, 131.4, 129.0, 127.1, 125.8, 125.3, 123.0, 122.8, 54.8, 53.9, 38.3, 31.6, 31.5, 26.7, 26.1, 26.0, 23.6. HRMS (ESI) m/z calcd for $C_{19}H_{26}N^{+}$ $(M+H)^{+}$ 268.20598, found 268.20633. $[\alpha]_D^{20}$ =+18.9 (c=1.0, EtOH). Enantiomeric excess was determined by chiral HPLC after the product was converted to the corresponding acetamide: OJ-3, Hex/IPA = 90:10, 1 mL/min, 220 nm, 6.8 min, 7.9 min.

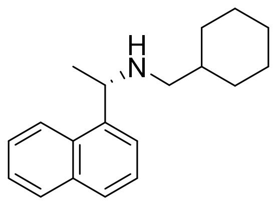

45

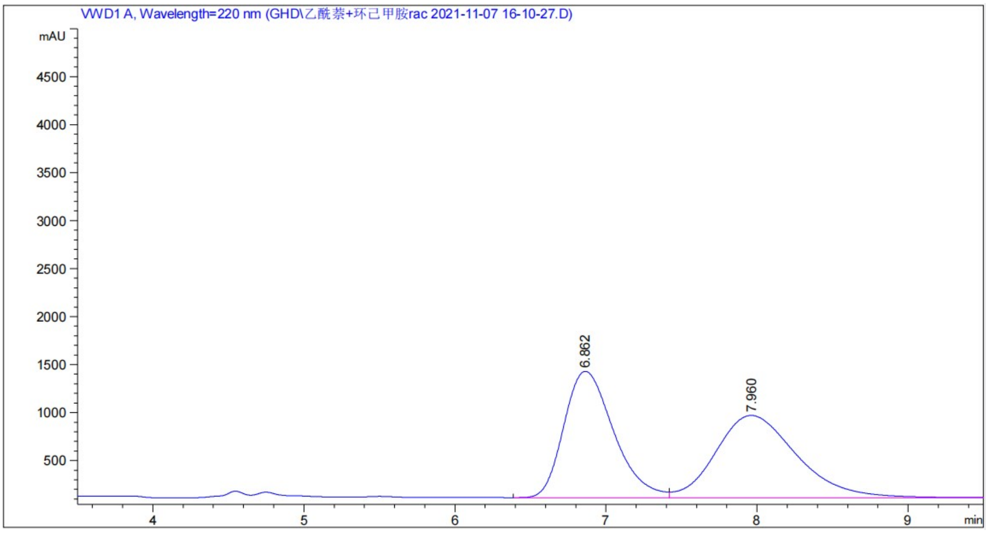

峰 #	保留时间 [min]	类型	峰宽 [min]	峰面积 [mAU*s]	峰高 [mAU]	峰面积 %
1	6.862	BV	0.3550	3.01029e4	1315.56726	48.5566
2	7.960	VBA	0.5723	3.18926e4	856.83154	51.4434

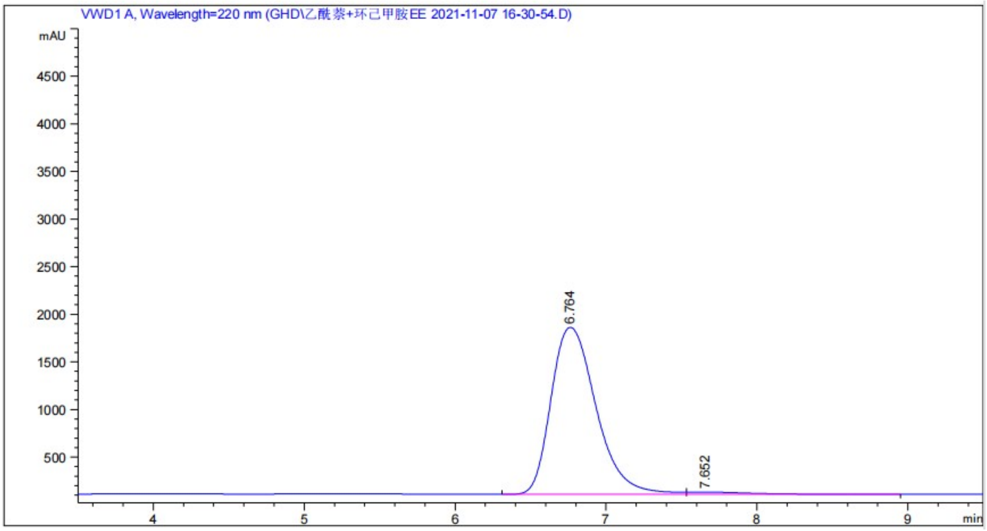

峰 #	保留时间 [min]	类型	峰宽 [min]	峰面积 [mAU*s]	峰高 [mAU]	峰面积 %
1	6.764	BV	0.3233	3.63990e4	1752.65771	98.2746
2	7.652	VV	0.4329	639.03973	21.87001	1.7254

Supplementary Figure 44. HPLC spectra for racemic and chiral **45**.

***N*-(1-(naphthalen-1-yl)ethyl)cyclopentanamine (46)**: 95% yield, 96% ee, colorless oil. ^{1}H NMR (500 MHz, $CDCl_3$): δ 8.27 (d, J = 8.3 Hz, 1H), 8.01 – 7.88 (m, 1H), 7.80 (d, J = 8.1 Hz, 1H), 7.71 (d, J = 7.1 Hz, 1H), 7.63 – 7.46 (m, 3H), 4.78 (q, J = 6.6 Hz, 1H), 3.08 (p, J = 7.1 Hz, 1H), 1.95 – 1.80 (m, 2H), 1.80 – 1.64 (m, 2H), 1.65 – 1.12 (m, 7H); ^{13}C NMR (125 MHz, $CDCl_3$): δ 141.8, 134.0, 131.4, 129.0, 127.1, 125.8, 125.3, 122.9, 122.9, 57.6, 51.7, 33.7, 33.3, 26.7, 24.2, 24.0, 23.8. HRMS (ESI) m/z calcd for $C_{17}H_{22}N^+$ $(M+H)^+$ 240.17468, found 240.17493. $[\alpha]_D^{20}$ =+8.4 (c=1.0, EtOH). Enantiomeric excess was determined by chiral HPLC after the product was converted to the corresponding acetamide: OJ-H, Hex/IPA = 90:10, 1 mL/min, 220 nm, 6.5 min, 10.1 min.

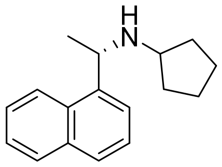

46

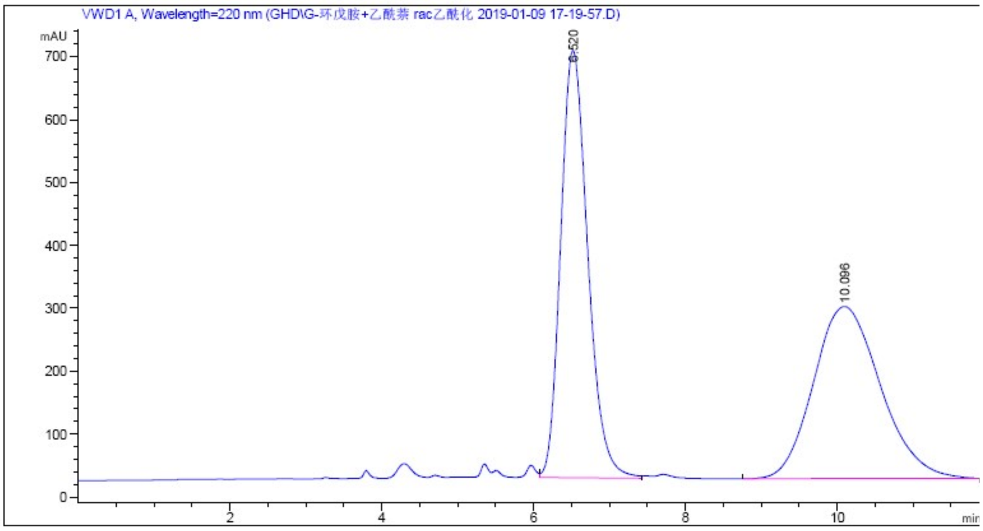

#	[min]		[min]	[mAU*s]	[mAU]	%
1	6.520	VV	0.3847	1.68042e4	679.06470	50.0409
2	10.096	BB	0.9433	1.67767e4	273.62872	49.9591

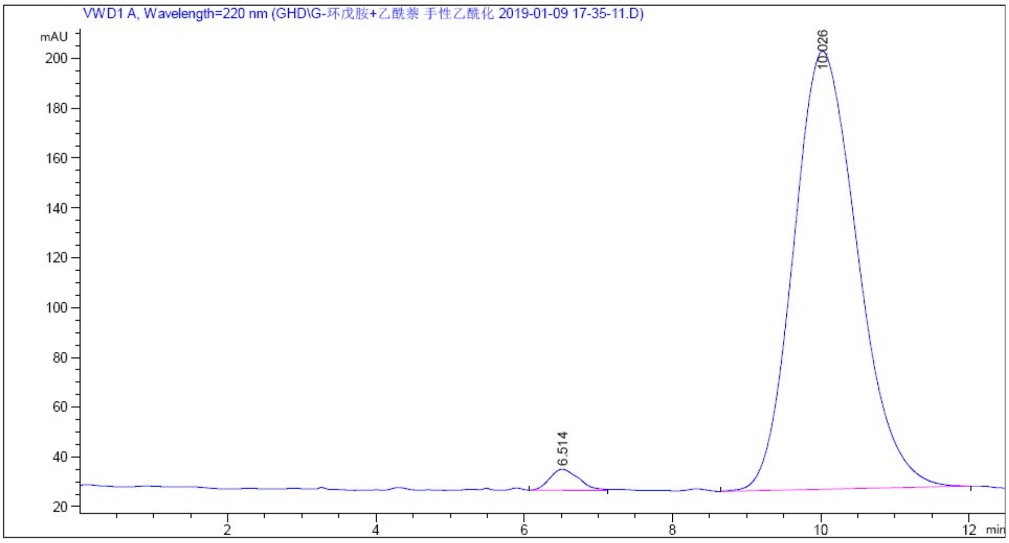

#	[min]		[min]	[mAU*s]	[mAU]	%
1	6.514	BV	0.4060	230.79298	8.41368	2.1448
2	10.026	BB	0.9261	1.05298e4	175.97020	97.8552

Supplementary Figure 45. HPLC spectra for racemic and chiral **46**.

N-(furan-2-ylmethyl)-1-phenylethan-1-amine (47):[8] 95% yield, 97% ee, brown oil. ^{1}H NMR (400 MHz, Chloroform-*d*) δ 7.33 (d, J = 4.5 Hz, 5H), 7.25 (q, J = 4.3 Hz, 1H), 6.29 (dd, J = 3.2, 1.8 Hz, 1H), 3.78 (q, J = 6.6 Hz, 1H), 3.71 – 3.52 (m, 2H), 1.84 (s, 1H), 1.36 (d, J = 6.6 Hz, 3H). $[\alpha]_D^{20}$ =-89.5 (c=0.5, EtOH). Enantiomeric excess was determined by chiral HPLC after the product was converted to the corresponding acetamide: Chiralpak AD-H column, Hex/IPA = 90:10, 1 mL/min, 220 nm, 7.0 min, 7.5 min.

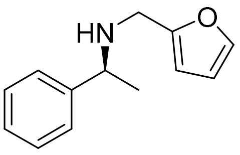

47

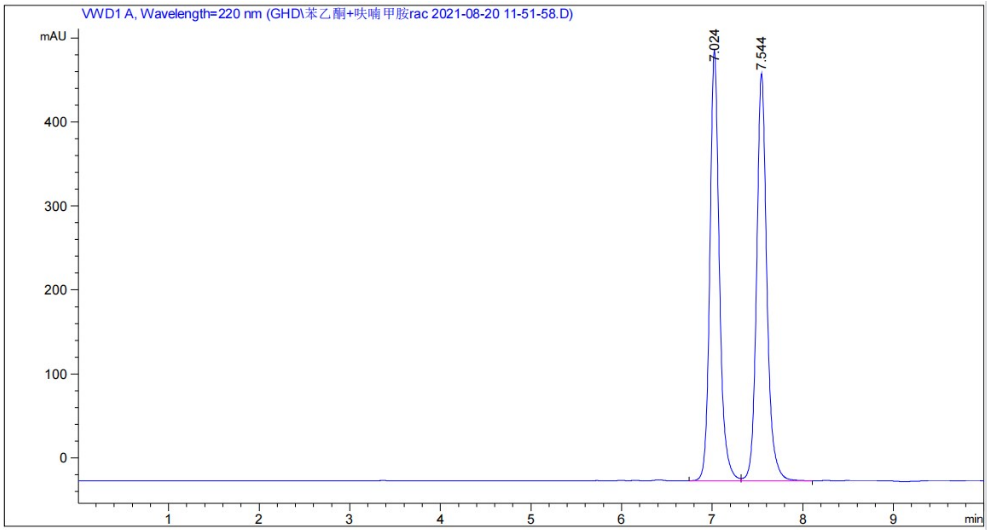

峰 #	保留时间 [min]	类型	峰宽 [min]	峰面积 [mAU*s]	峰高 [mAU]	峰面积 %
1	7.024	BV	0.1101	3742.85303	513.14471	49.5973
2	7.544	VB	0.1185	3803.63599	485.12766	50.4027

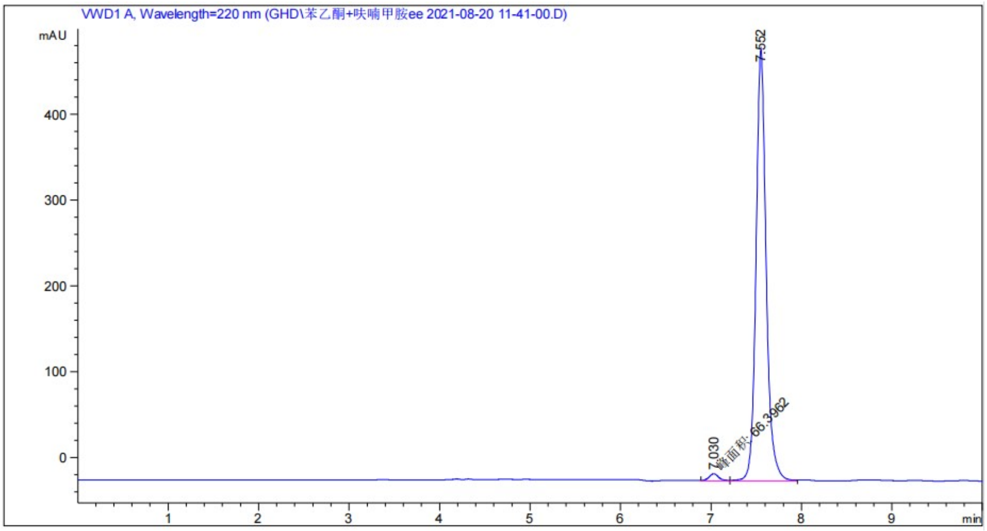

峰 #	保留时间 [min]	类型	峰宽 [min]	峰面积 [mAU*s]	峰高 [mAU]	峰面积 %
1	7.030	FM	0.1301	66.39621	8.50509	1.6620
2	7.552	VV	0.1183	3928.64844	502.07617	98.3380

Supplementary Figure 46. HPLC spectra for racemic and chiral **47**.

1-phenyl-N-(2-(thiophen-2-yl)ethyl)-1l3-ethan-1-amine (48): 95% yield, 97% ee, brown oil. ^{1}H NMR (400 MHz, Chloroform-*d*) δ 7.33 – 7.21 (m, 2H), 7.20 – 7.07 (m, 3H), 6.89 (td, *J* = 5.2, 4.8, 2.1 Hz, 2H), 6.78 (dd, *J* = 8.2, 2.5 Hz, 1H), 3.81 (d, *J* = 1.3 Hz, 3H), 3.73 (dq, *J* = 9.3, 6.6 Hz, 1H), 2.83 – 2.41 (m, 4H), 1.79 (dddd, *J* = 14.5, 12.5, 9.1, 6.4 Hz, 2H), 1.61 (s, 1H), 1.34 (dd, *J* = 6.6, 4.2 Hz, 3H). ^{13}C NMR (101 MHz, Chloroform-*d*) δ 145.5, 142.7, 128.4, 126.9, 126.8, 126.6, 124.9, 123.5, 58.1, 48.9, 30.5, 24.3. $[\alpha]_D^{20}$ =-54.5 (c=0.5, EtOH). Enantiomeric excess was determined by chiral HPLC after the product was converted to the corresponding acetamide: Chiralpak AD-H column, Hex/IPA = 90:10, 1 mL/min, 220 nm, 11.2 min, 12.4 min.

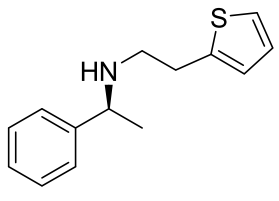

48

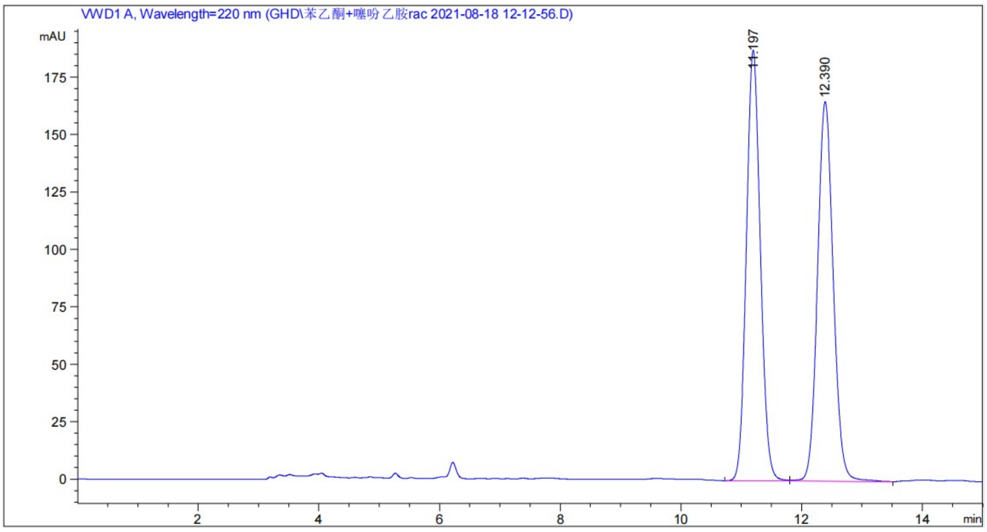

峰 #	保留时间 [min]	类型	峰宽 [min]	峰面积 [mAU*s]	峰高 [mAU]	峰面积 %
1	11.197	BV	0.2499	3020.23315	187.48233	50.1881
2	12.390	VB	0.2797	2997.59399	165.17590	49.8119

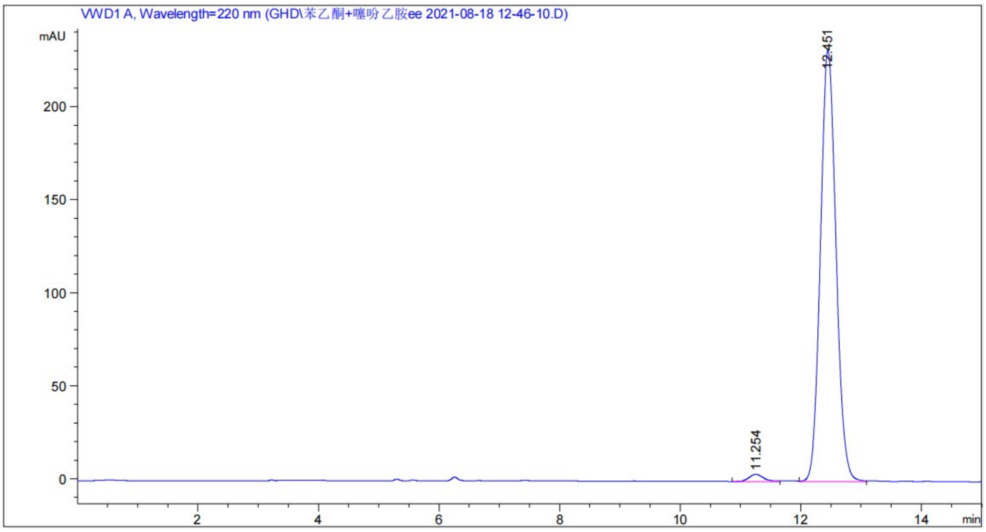

峰 #	保留时间 [min]	类型	峰宽 [min]	峰面积 [mAU*s]	峰高 [mAU]	峰面积 %
1	11.254	BV	0.2629	65.36448	3.83358	1.5459
2	12.451	VV	0.2795	4162.82520	231.81723	98.4541

Supplementary Figure 47. HPLC spectra for racemic and chiral **48**.

N-(2-(1H-indol-3-yl)ethyl)-1-phenylethan-1-amine (49):[4] 95% yield, 93% ee, colorless oil. ^{1}H NMR (400 MHz, Chloroform-*d*) δ 8.14 (s, 1H), 7.54 (d, J = 7.9 Hz, 1H), 7.37 – 7.13 (m, 7H), 7.08 (t, J = 7.4 Hz, 1H), 6.95 (d, J = 2.2 Hz, 1H), 3.79 (q, J = 6.6 Hz, 1H), 2.95 (t, J = 6.3 Hz, 2H), 2.90 – 2.75 (m, 2H), 1.93 (s, 1H), 1.33 (d, J = 6.6 Hz, 3H). $[\alpha]_D^{20}$ =-35.2 (c=1.0, EtOH). Enantiomeric excess was determined by chiral HPLC after the product was converted to the corresponding trifluoroacetamide: AD-H, Hex/IPA = 90:10, 1 mL/min, 220 nm, 11.4 min, 12.6 min.

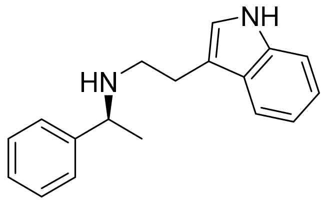

49

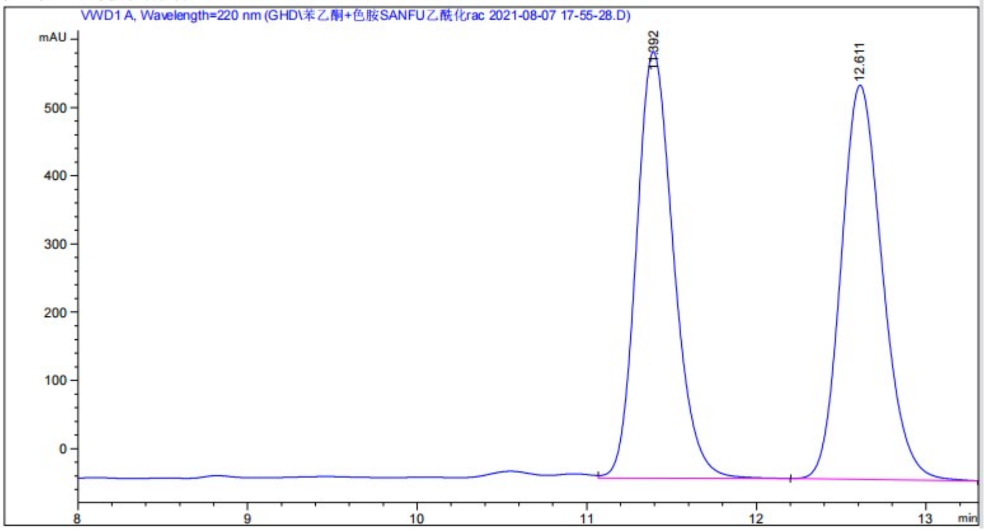

峰 #	保留时间 [min]	类型	峰宽 [min]	峰面积 [mAU*s]	峰高 [mAU]	峰面积 %
1	11.392	VB	0.2353	9501.11328	625.23859	49.3651
2	12.611	BB	0.2608	9745.52148	577.72723	50.6349

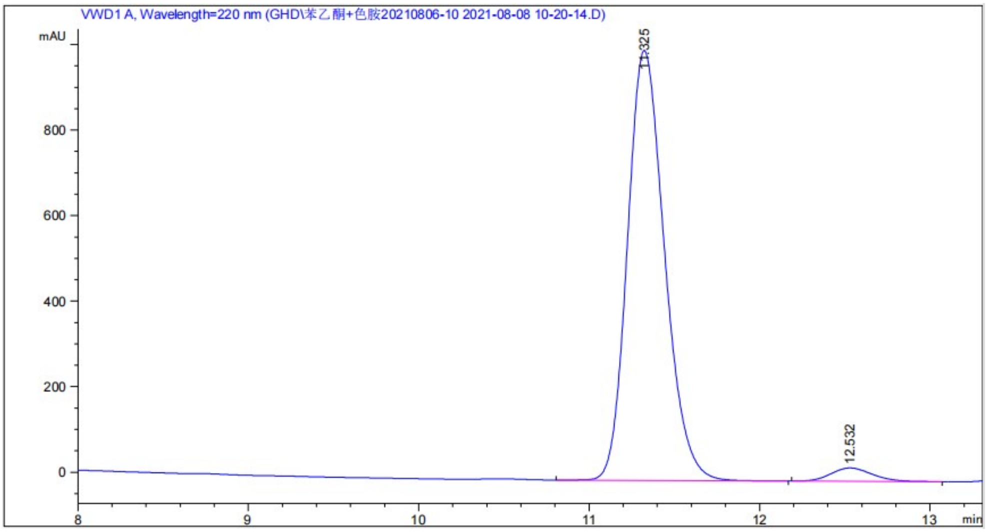

峰 #	保留时间 [min]	类型	峰宽 [min]	峰面积 [mAU*s]	峰高 [mAU]	峰面积 %
1	11.325	BB	0.2343	1.51916e4	1005.54749	96.6562
2	12.532	BB	0.2605	525.54309	31.19539	3.3438

Supplementary Figure 48. HPLC spectra for racemic and chiral **49**.

Phenyl-N-(pyridin-3-ylmethyl)ethan-1-amine (50):[9] 94% yield, 93% ee, brown oil. ^{1}H NMR (400 MHz, Chloroform-*d*) δ 8.55 – 8.41 (m, 2H), 7.64 (dt, *J* = 7.8, 1.9 Hz, 1H), 7.35 (d, *J* = 5.1 Hz, 4H), 7.25 (ddt, *J* = 12.6, 7.9, 4.1 Hz, 2H), 3.80 (q, *J* = 6.6 Hz, 1H), 3.69 – 3.56 (m, 2H), 1.85 (s, 1H), 1.38 (d, *J* = 6.6 Hz, 3H). ^{13}C NMR (101 MHz, Chloroform-*d*) δ 149.7, 148.3, 145.1, 135.8, 128.6, 127.1, 126.6, 123.3, 57.6, 48.9, 24.4. $[\alpha]_D^{20}$ =+8.5 (c=1.0, EtOH). Enantiomeric excess was determined by chiral HPLC after the product was converted to the corresponding acetamide: AD-H, Hex/IPA = 90:10, 1 mL/min, 220 nm, 22.3 min, 27.1 min.

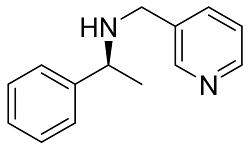

50

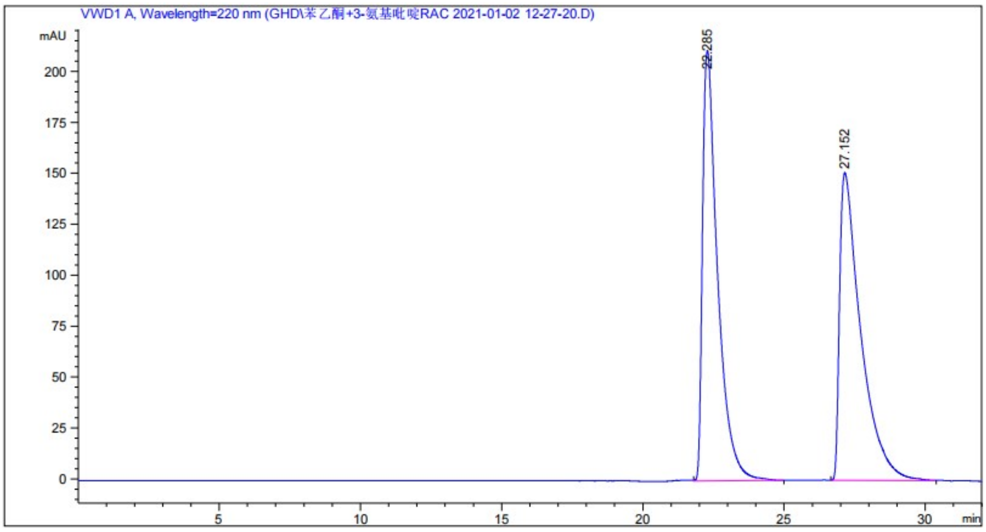

峰 #	保留时间 [min]	类型	峰宽 [min]	峰面积 [mAU*s]	峰高 [mAU]	峰面积 %
1	22.285	VB	0.5396	7847.21338	211.13625	49.8633
2	27.152	VV	0.7500	7890.24707	150.90987	50.1367

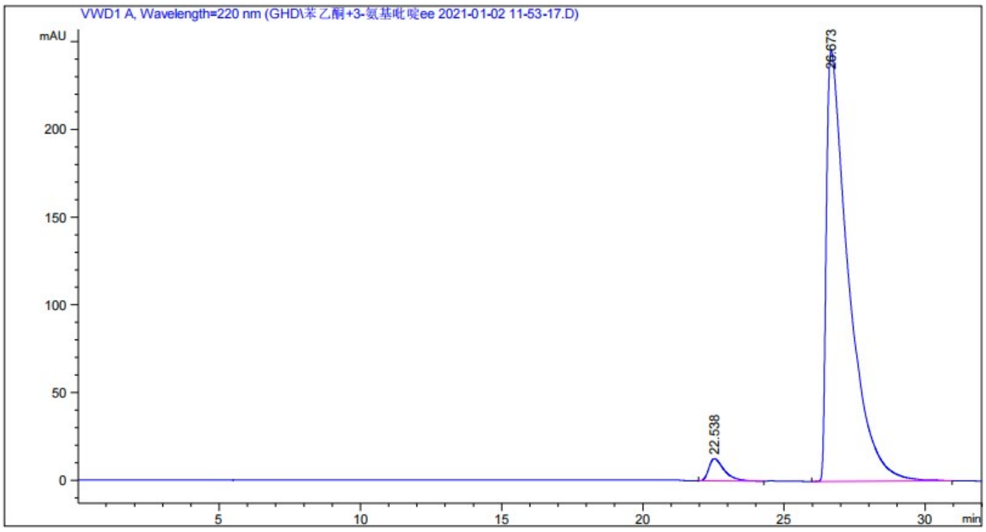

峰 #	保留时间 [min]	类型	峰宽 [min]	峰面积 [mAU*s]	峰高 [mAU]	峰面积 %
1	22.538	BV	0.5743	492.84662	12.79878	3.5616
2	26.673	BV	0.7869	1.33448e4	245.59335	96.4384

Supplementary Figure 49. HPLC spectra for racemic and chiral **50**.

***tert*-butyl-(3-((1-phenylethyl)amino)propyl)carbamate (51)**: 96% yield, 95% ee, brown oil. ^{1}H NMR (400 MHz, Chloroform-*d*) δ 7.31 – 7.10 (m, 5H), 4.90 (s, 1H), 3.68 (q, J = 6.6 Hz, 1H), 3.08 (p, J = 6.8 Hz, 2H), 2.54 (dt, J = 11.6, 5.6 Hz, 1H), 2.45 (ddd, J = 12.2, 7.1, 5.2 Hz, 1H), 1.60 – 1.46 (m, 1H), 1.36 (s, 9H), 1.26 (d, J = 6.6 Hz, 3H). ^{13}C NMR (101 MHz, Chloroform-*d*) δ 155.1, 144.3, 127.4, 125.9, 125.4, 56.9, 45.9, 39.5, 27.4, 23.2. $[\alpha]_D^{20}$ =+24.1 (c=1.0, EtOH). Enantiomeric excess was determined by chiral HPLC after the product was converted to the corresponding acetamide: Chiralpak AS-H column, Hex/IPA = 90:10, 1 mL/min, 220 nm, 8.1 min, 12.5 min.

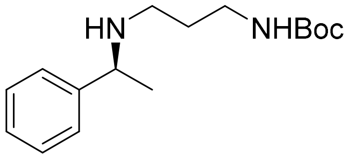

51

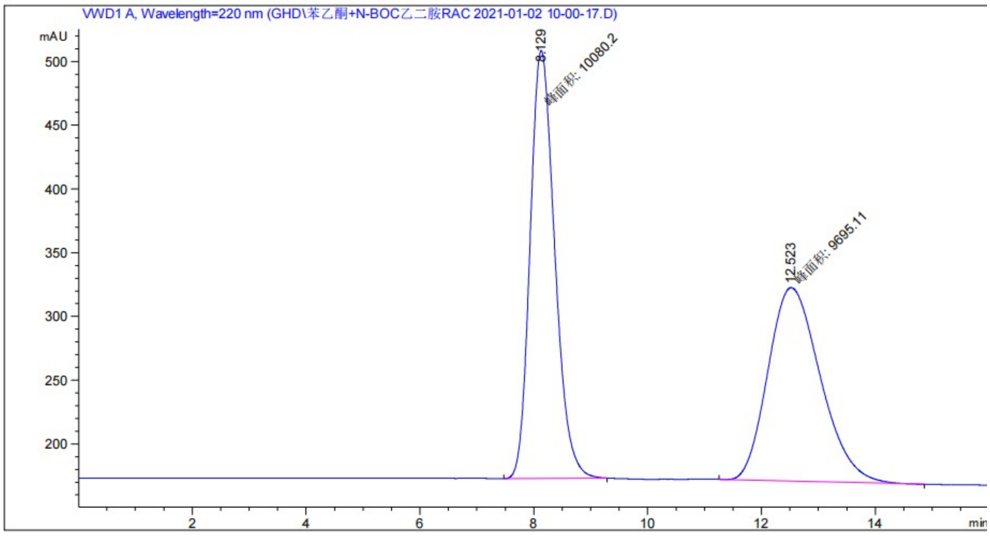

峰 #	保留时间 [min]	类型	峰宽 [min]	峰面积 [mAU*s]	峰高 [mAU]	峰面积 %
1	8.129	MM	0.5005	1.00802e4	335.69489	50.9736
2	12.523	MM	1.0649	9695.10645	151.74081	49.0264

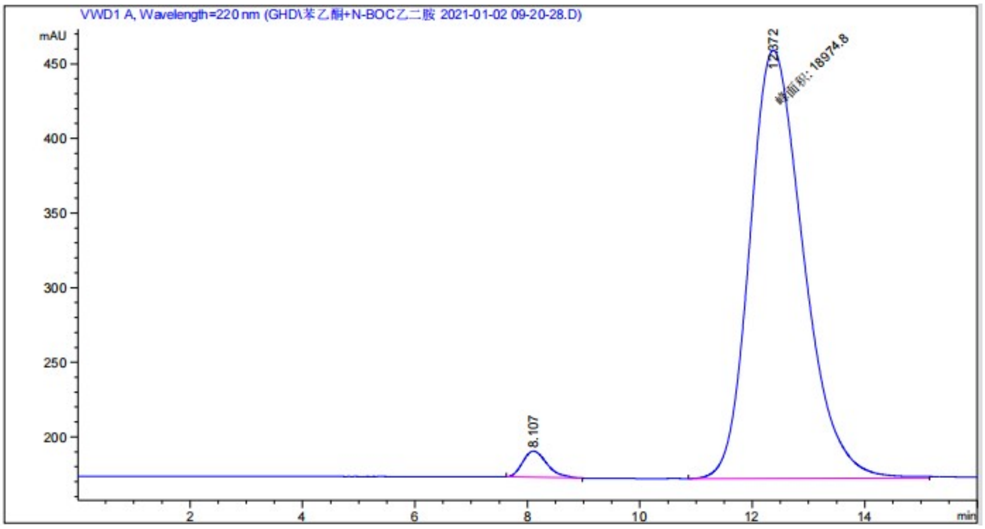

峰 #	保留时间 [min]	类型	峰宽 [min]	峰面积 [mAU*s]	峰高 [mAU]	峰面积 %
1	8.107	VB	0.4693	523.53912	17.48448	2.6850
2	12.372	MF	1.1026	1.89748e4	286.81433	97.3150

Supplementary Figure 50. HPLC spectra for racemic and chiral **51**.

3-methoxy-N-(1-phenylethyl)propan-1-amine (52):[3] 95% yield, 95% ee, brown oil. ^{1}H NMR (400 MHz, Chloroform-*d*) δ 7.31 (d, *J* = 5.5 Hz, 4H), 7.24 (dt, *J* = 8.7, 5.6 Hz, 1H), 3.76 (q, *J* = 6.6 Hz, 1H), 3.40 (td, *J* = 6.3, 1.5 Hz, 2H), 3.29 (d, *J* = 0.9 Hz, 3H), 2.59 (dt, *J* = 11.4, 6.9 Hz, 1H), 2.50 (dt, *J* = 11.5, 7.1 Hz, 1H), 2.13 – 1.91 (m, 1H), 1.73 (p, *J* = 6.7 Hz, 2H), 1.35 (d, *J* = 6.6 Hz, 3H). $[\alpha]_D^{20}$ =-40.0 (c=1.0, EtOH). Enantiomeric excess was determined by chiral HPLC after the product was converted to the corresponding acetamide: Chiralpak AD-H column, Hex/IPA = 90:10, 1 mL/min, 220 nm, 7.2 min, 7.6 min.

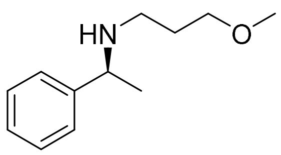

52

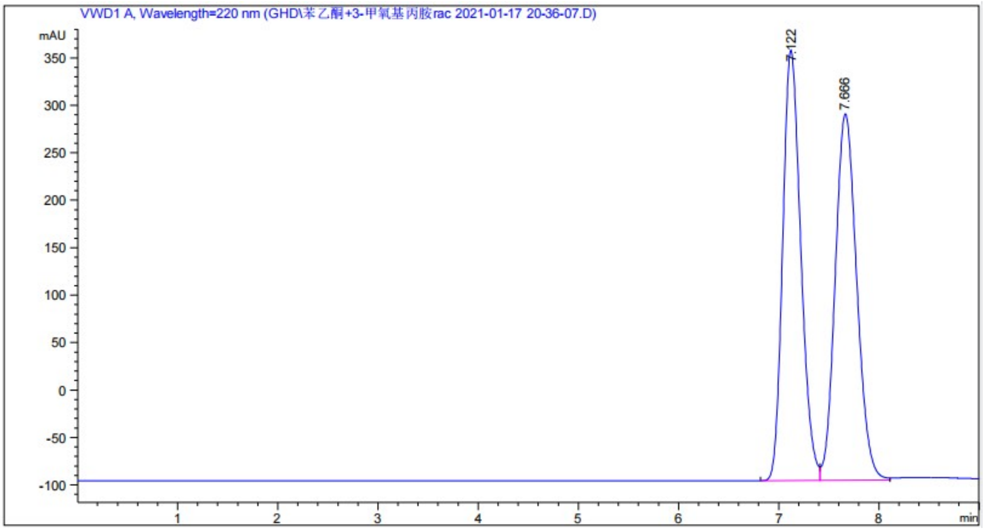

峰 #	保留时间 [min]	类型	峰宽 [min]	峰面积 [mAU*s]	峰高 [mAU]	峰面积 %
1	7.122	BV	0.1941	5676.91064	453.18054	49.6223
2	7.666	VV	0.2322	5763.33936	385.96384	50.3777

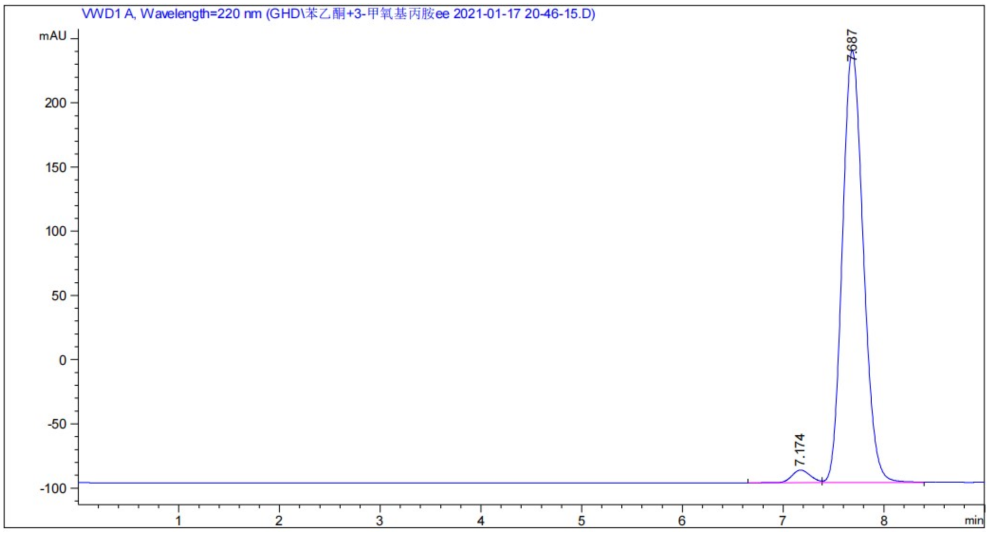

#	[min]		[min]	[mAU*s]	[mAU]	%
1	7.174	BV	0.1981	125.83643	9.84636	2.5314
2	7.687	VV	0.2249	4845.16748	336.58975	97.4686

Supplementary Figure 51. HPLC spectra for racemic and chiral **52**.

2-(cyclohex-1-en-1-yl)-N-(1-phenylethyl)ethan-1-amine(53):[6] 94% yield, 95% ee, brown oil. ^{1}H NMR (400 MHz, Chloroform-*d*) δ 7.40 – 7.17 (m, 5H), 5.43 (tt, J = 3.8, 1.7 Hz, 1H), 3.75 (q, J = 6.6 Hz, 1H), 2.67 – 2.40 (m, 2H), 2.10 (t, J = 7.0 Hz, 2H), 1.97 (dtd, J = 7.0, 5.1, 4.6, 2.5 Hz, 2H), 1.84 – 1.78 (m, 2H), 1.55 (dtdd, J = 17.7, 10.2, 5.0, 2.3 Hz, 4H), 1.35 (d, J = 6.6 Hz, 3H). $[\alpha]_D^{20}$ =+11.0 (c=1.0, EtOH). Enantiomeric excess was determined by chiral HPLC after the product was converted to the corresponding acetamide: Chiralpak AD-H column, Hex/IPA = 90:10, 1 mL/min, 220 nm, 6.9 min, 7.7 min.

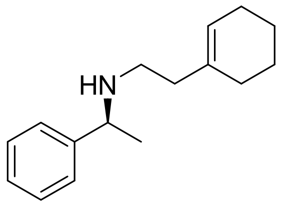

53

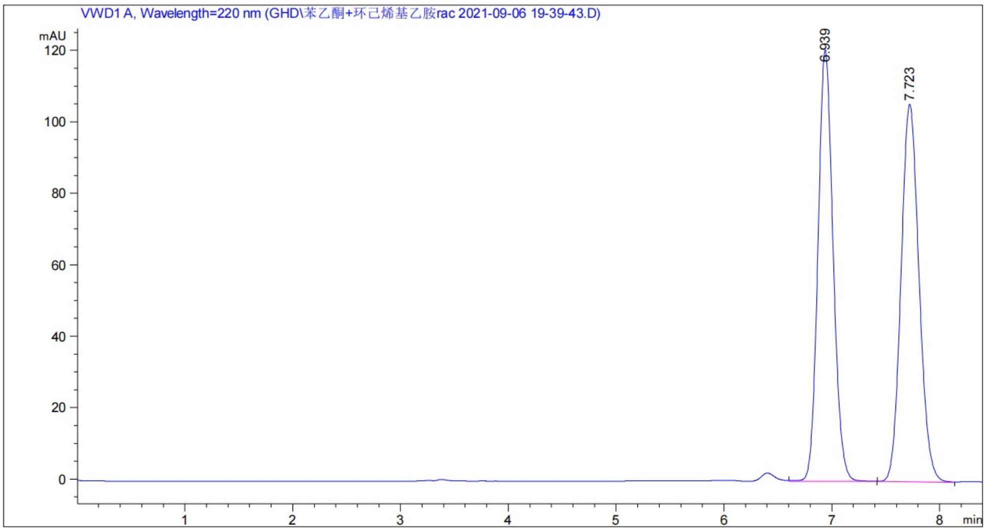

峰 #	保留时间 [min]	类型	峰宽 [min]	峰面积 [mAU*s]	峰高 [mAU]	峰面积 %
1	6.939	VB	0.1507	1177.08301	120.76971	49.0266
2	7.723	BV	0.1792	1223.82471	105.64319	50.9734

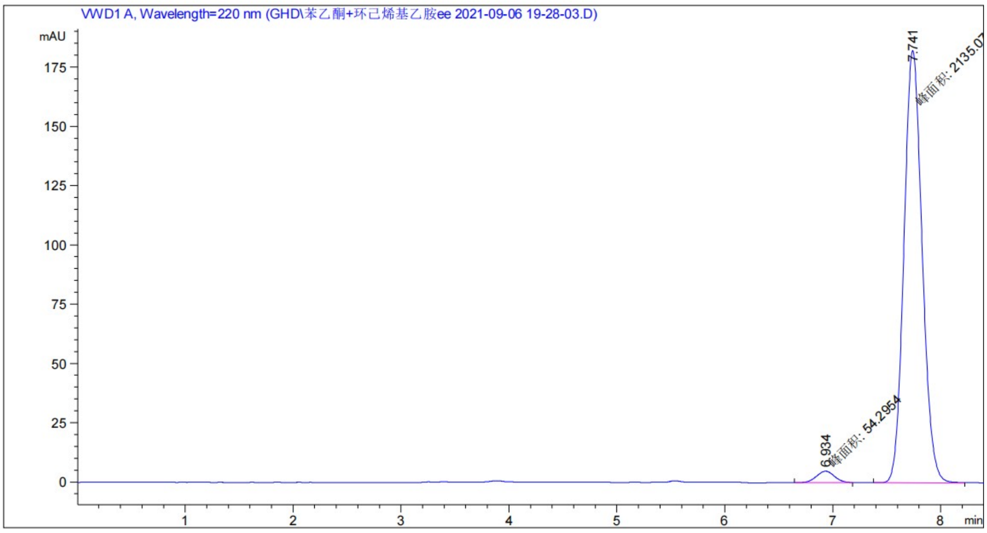

峰 #	保留时间 [min]	类型	峰宽 [min]	峰面积 [mAU*s]	峰高 [mAU]	峰面积 %
1	6.934	MM	0.1895	54.29544	4.77630	2.4800
2	7.741	MF	0.1950	2135.06616	182.47360	97.5200

Supplementary Figure 52. HPLC spectra for racemic and chiral **53**.

Phenyl-N-(3-phenylpropyl)butan-2-amine (54):[10] 95% yield, 94% ee, brown oil. ^{1}H NMR (400 MHz, Chloroform-*d*) δ 7.26 (t, *J* = 7.4 Hz, 4H), 7.20 – 7.12 (m, 6H), 2.71 – 2.50 (m, 7H), 1.77 (dq, *J* = 15.9, 9.1, 8.3 Hz, 3H), 1.60 (ddt, *J* = 13.4, 10.3, 6.6 Hz, 1H), 1.38 (s, 1H), 1.07 (d, *J* = 6.3 Hz, 3H). ^{13}C NMR (101 MHz, Chloroform-*d*) δ 142.4, 142.2, 128.4, 128.4, 125.8, 125.7, 52.7, 46.7, 38.7, 33.8, 32.4, 32.0, 20.43. $[\alpha]_D^{20}$ =-5.7 (c=1.0, EtOH). Enantiomeric excess was determined by chiral HPLC after the product was converted to the corresponding acetamide: IB-3, Hex/IPA = 90:10, 1 mL/min, 220 nm, 10.3 min, 11.0 min.

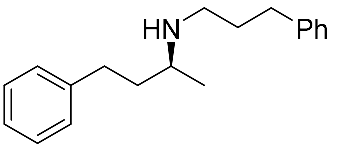

54

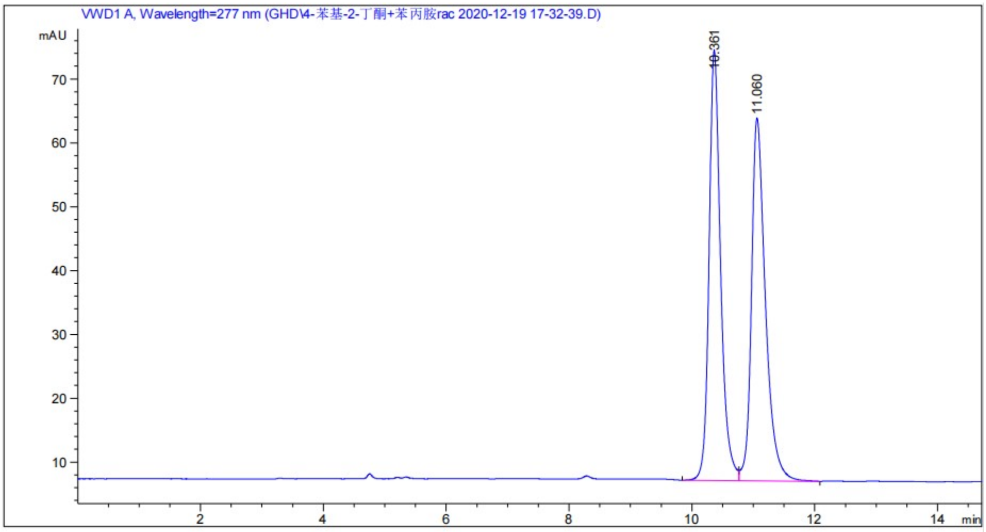

峰 #	保留时间 [min]	类型	峰宽 [min]	峰面积 [mAU*s]	峰高 [mAU]	峰面积 %
1	10.361	BV	0.1952	873.35541	67.38835	49.2682
2	11.060	VB	0.2366	899.29932	56.82100	50.7318

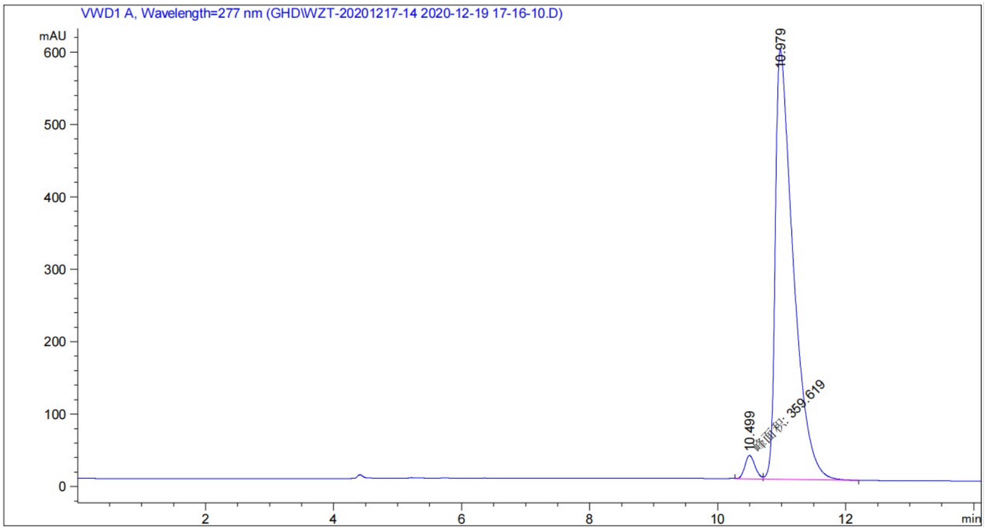

峰 #	保留时间 [min]	类型	峰宽 [min]	峰面积 [mAU*s]	峰高 [mAU]	峰面积 %
1	10.499	FM	0.1847	359.61926	32.44995	3.0060
2	10.979	VB	0.2883	1.16039e4	593.06885	96.9940

Supplementary Figure 53. HPLC spectra for racemic and chiral **54**.

N-(3-phenylpropyl)hexan-2-amine (55):[10] 95% yield, 88% ee, colorless oil. ^{1}H NMR (400 MHz, Chloroform-*d*) δ 7.32 – 7.23 (m, 2H), 7.19 (d, *J* = 7.3 Hz, 3H), 2.72 – 2.53 (m, 5H), 1.81 (p, *J* = 7.4 Hz, 2H), 1.59 (s, 1H), 1.43 (t, *J* = 5.9 Hz, 1H), 1.36 – 1.18 (m, 5H), 1.02 (d, *J* = 6.1 Hz, 3H), 0.89 (t, *J* = 6.7 Hz, 3H). $[\alpha]_D^{20}$ =-8.6 (c=1.0, EtOH). Enantiomeric excess was determined by chiral HPLC after the product was converted to the corresponding acetamide: AD-H, Hex/IPA = 90:10, 1 mL/min, 220 nm, 7.5 min, 8.9 min.

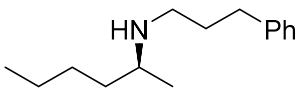

55

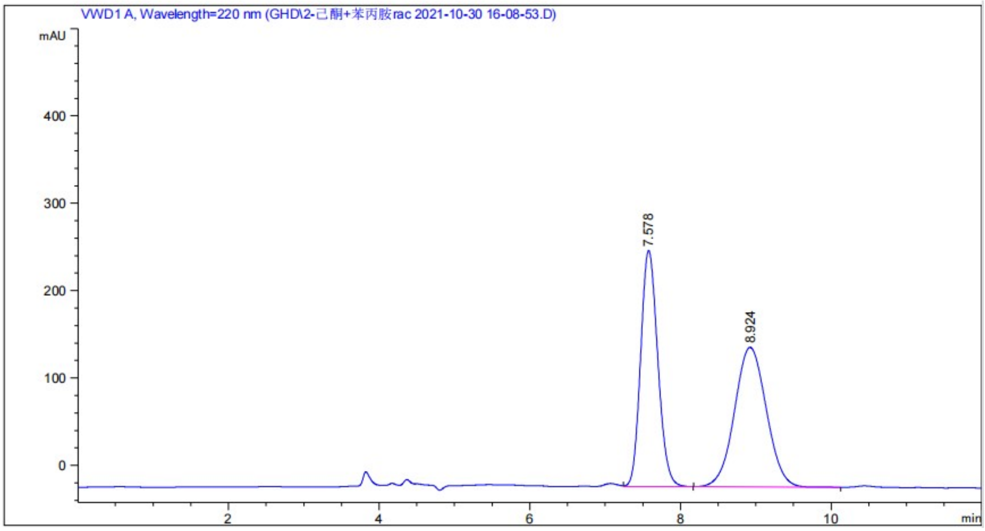

峰 #	保留时间 [min]	类型	峰宽 [min]	峰面积 [mAU*s]	峰高 [mAU]	峰面积 %
1	7.578	VB	0.2459	4261.37988	270.27032	47.5349
2	8.924	BV	0.4593	4703.36572	159.83472	52.4651

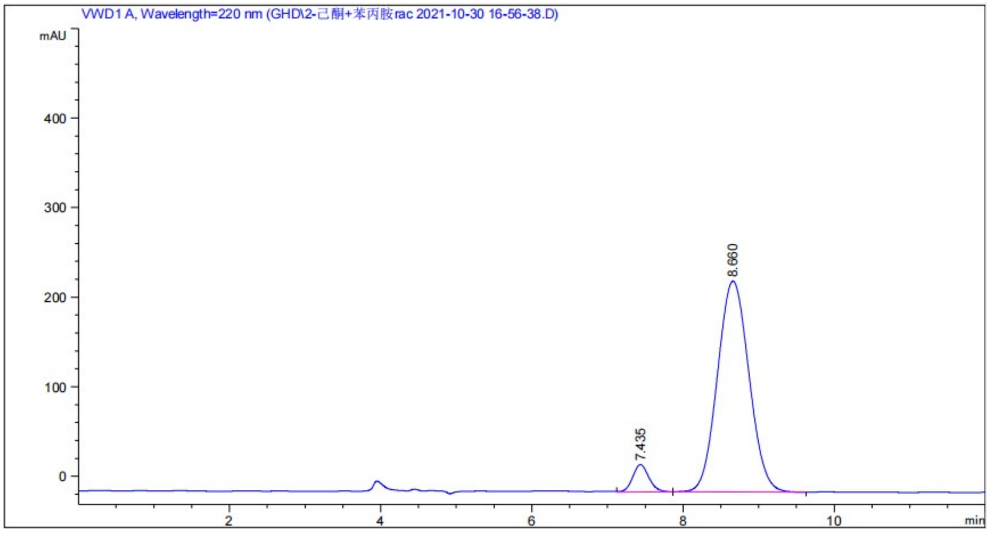

峰 #	保留时间 [min]	类型	峰宽 [min]	峰面积 [mAU*s]	峰高 [mAU]	峰面积 %
1	7.435	VB	0.2304	456.85141	30.55924	6.2815
2	8.660	BB	0.4578	6816.13428	235.42532	93.7185

Supplementary Figure 54. HPLC spectra for racemic and chiral **55**.

5-methyl-N-(3-phenylpropyl)hexan-2-amine (56): 95% yield, 90% ee, brown oil. ^{1}H NMR (400 MHz, Chloroform-*d*) δ 7.32 – 7.24 (m, 2H), 7.19 (d, *J* = 7.5 Hz, 3H), 2.73 – 2.48 (m, 5H), 1.81 (p, *J* = 7.4 Hz, 2H), 1.56 – 1.33 (m, 3H), 1.32 – 1.21 (m, 1H), 1.16 (dt, *J* = 9.3, 6.8 Hz, 2H), 1.02 (d, *J* = 6.3 Hz, 3H), 0.88 (d, *J* = 6.7 Hz, 6H). ^{13}C NMR (101 MHz, Chloroform-*d*) δ 142.2, 128.3, 128.3, 125.7, 53.4, 46.8, 35.2, 34.9, 33.8, 32.0, 28.3, 22.7, 22.5, 20.3. $[\alpha]_D^{20}$ =+7.8 (c=0.5, EtOH). Enantiomeric excess was determined by chiral HPLC after the product was converted to the corresponding acetamide: AS-H, Hex/IPA = 90:10, 1 mL/min, 220 nm, 7.6 min, 8.9 min.

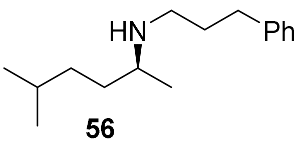

56

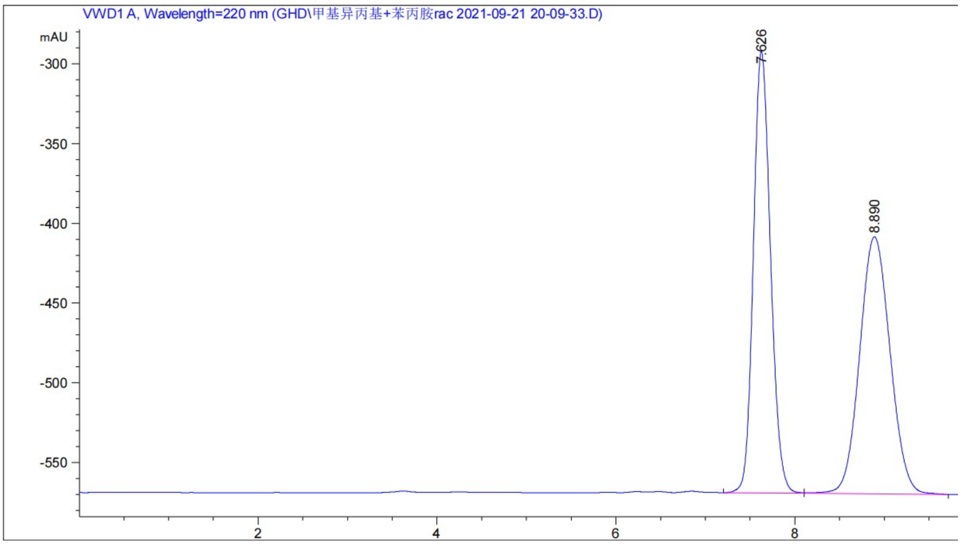

峰 #	保留时间 [min]	类型	峰宽 [min]	峰面积 [mAU*s]	峰高 [mAU]	峰面积 %
1	7.626	BV	0.2074	3687.23999	276.89767	49.2160
2	8.890	VB	0.3694	3804.71899	161.15222	50.7840

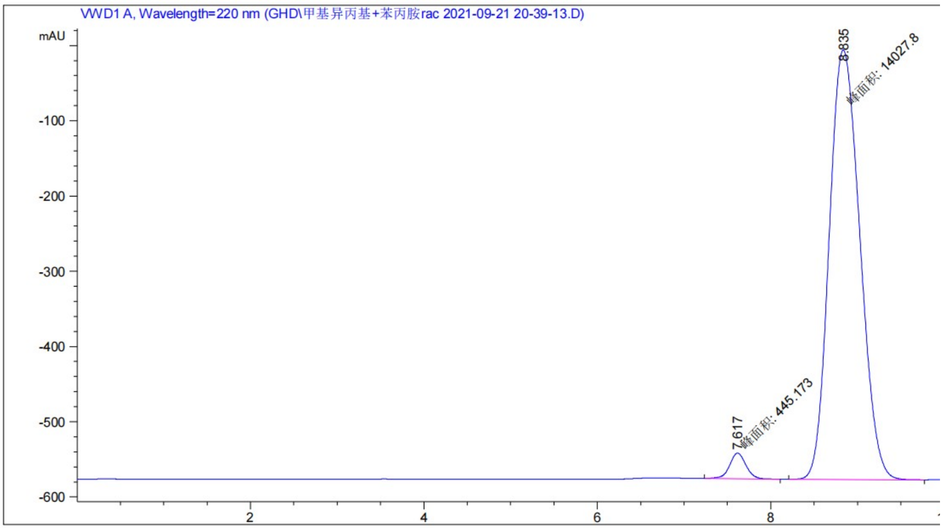

峰 #	保留时间 [min]	类型	峰宽 [min]	峰面积 [mAU*s]	峰高 [mAU]	峰面积 %
1	7.617	MM	0.2174	445.17303	34.12295	3.0759
2	8.835	MM	0.4094	1.40278e4	571.09967	96.9241

Supplementary Figure 55. HPLC spectra for racemic and chiral **56**.

3-methyl-N-(3-phenylpropyl)butan-2-amine (57): 95% yield, 90% ee, colorless oil. ^{1}H NMR (400 MHz, Chloroform-*d*) δ 7.27 (t, *J* = 7.5 Hz, 2H), 7.18 (d, *J* = 7.7 Hz, 3H), 2.67 (dt, *J* = 15.3, 7.6 Hz, 3H), 2.56 (dt, *J* = 11.3, 7.3 Hz, 1H), 2.47 – 2.39 (m, 1H), 1.80 (p, *J* = 7.3 Hz, 2H), 1.67 (pd, *J* = 7.0, 5.0 Hz, 1H), 1.13 (s, 1H), 0.94 (d, *J* = 6.5 Hz, 3H), 0.86 (dd, *J* = 15.6, 6.9 Hz, 6H). ^{13}C NMR (101 MHz, Chloroform-*d*) δ 142.29, 128.41, 128.33, 125.74, 58.31, 47.13, 33.82, 32.15 (d, *J* = 12.2 Hz), 19.47, 17.18, 15.99. $[\alpha]_D^{20}$ =+11.6 (c=0.5, EtOH).. Enantiomeric excess was determined by chiral HPLC after the product was converted to the corresponding acetamide: AS-H, Hex/IPA = 90:10, 1 mL/min, 220 nm, 9.1 min, 11.1 min.

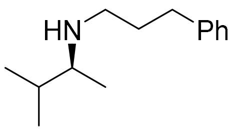

57

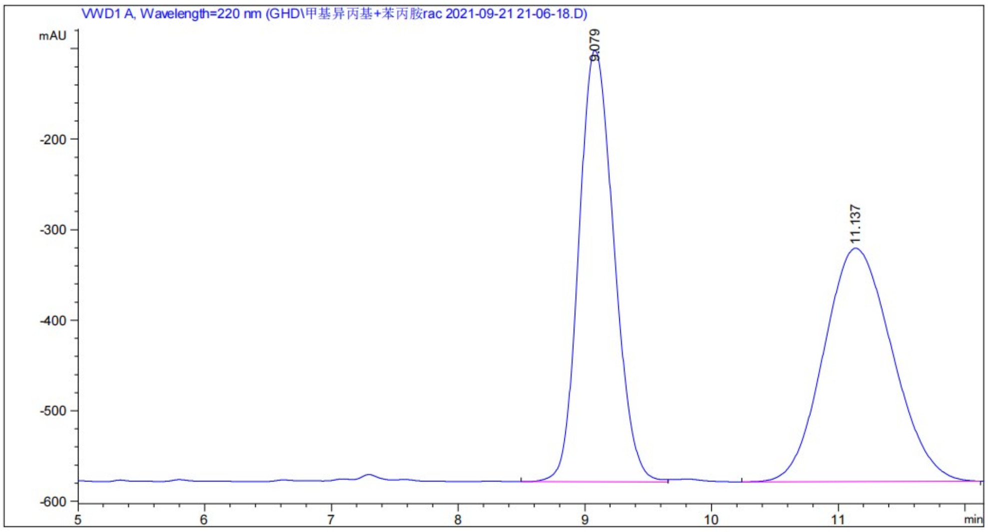

峰 #	保留时间 [min]	类型	峰宽 [min]	峰面积 [mAU*s]	峰高 [mAU]	峰面积 %
1	9.079	BV	0.3022	9160.12988	476.58087	48.6811
2	11.137	BB	0.5932	9656.48633	257.69214	51.3189

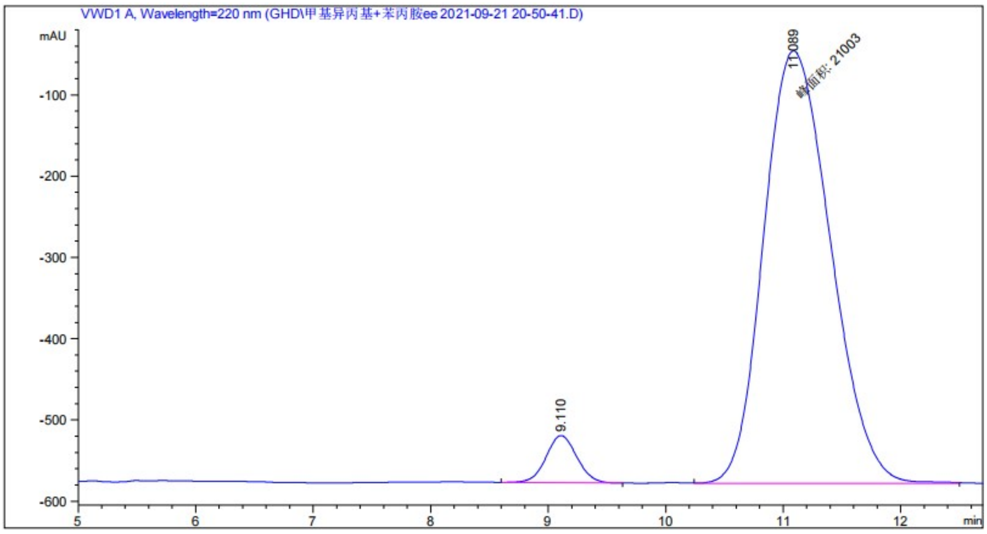

峰 #	保留时间 [min]	类型	峰宽 [min]	峰面积 [mAU*s]	峰高 [mAU]	峰面积 %
1	9.110	BB	0.2829	1059.34460	58.03852	4.8016
2	11.089	MF	0.6578	2.10030e4	532.14368	95.1984

Supplementary Figure 56. HPLC spectra for racemic and chiral **57**.

N-(1-cyclohexylethyl)-3-phenylpropan-1-amine (58):[11] 94% yield, 96% ee, colorless oil. ^{1}H NMR (400 MHz, Chloroform-*d*) δ 7.30 – 7.23 (m, 2H), 7.18 (d, *J* = 7.5 Hz, 3H), 2.67 (ddd, *J* = 13.6, 9.8, 6.9 Hz, 3H), 2.55 (dt, *J* = 11.4, 7.2 Hz, 1H), 2.47 – 2.38 (m, 1H), 1.88 – 1.60 (m, 8H), 1.39 – 1.06 (m, 5H), 1.01 – 0.94 (m, 4H). $[\alpha]_D^{20}$ =-6.5 (c=1.0, EtOH). Enantiomeric excess was determined by chiral HPLC after the product was converted to the corresponding acetamide: IB-3, Hex/IPA = 90:10, 1 mL/min, 220 nm, 9.2 min, 10.1 min.

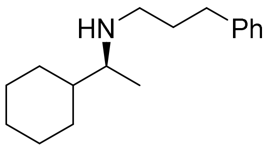

58

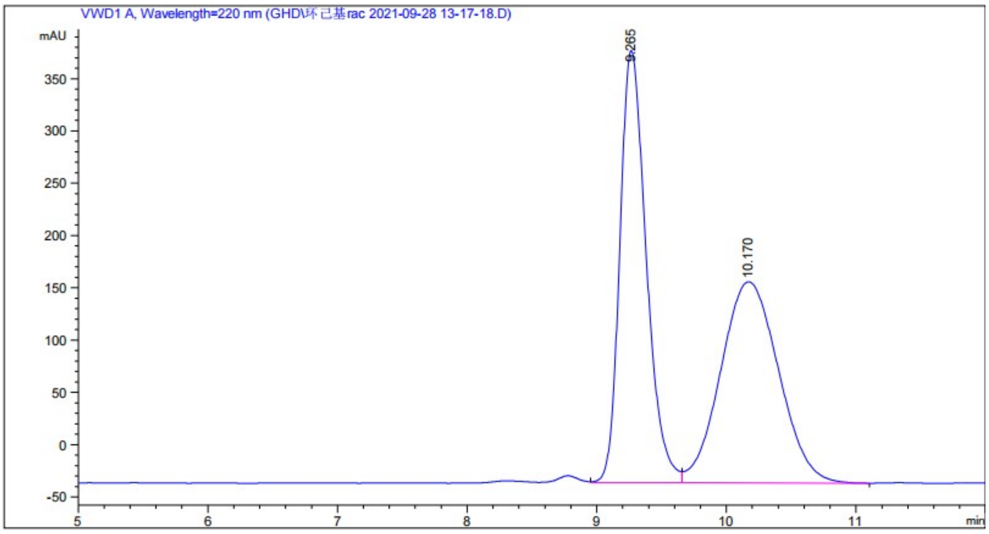

峰 #	保留时间 [min]	类型	峰宽 [min]	峰面积 [mAU*s]	峰高 [mAU]	峰面积 %
1	9.265	VV	0.2190	5838.22803	412.88666	49.4687
2	10.170	VB	0.4878	5963.62598	192.31772	50.5313

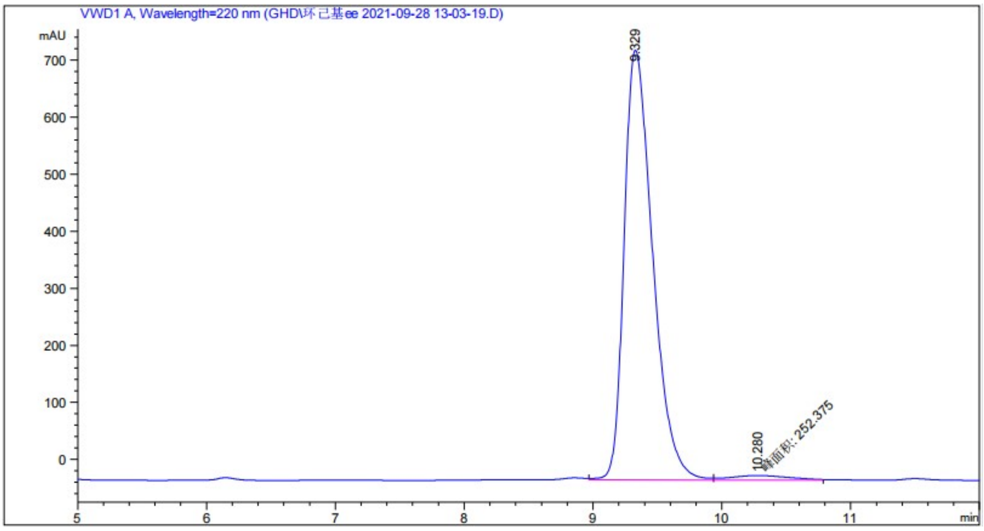

峰 #	保留时间 [min]	类型	峰宽 [min]	峰面积 [mAU*s]	峰高 [mAU]	峰面积 %
1	9.329	VV	0.2394	1.15695e4	752.43176	97.8652
2	10.280	MF	0.5215	252.37466	8.06554	2.1348

Supplementary Figure 57. HPLC spectra for racemic and chiral **58**.

N-(1-cyclohexylethyl)hexan-1-amine (59): 95% yield, 96% ee, brown oil. ^{1}H NMR (400 MHz, Chloroform-*d*) δ 2.63 (dt, *J* = 11.2, 7.3 Hz, 1H), 2.46 (ddt, *J* = 26.7, 11.6, 6.7 Hz, 2H), 1.82 – 1.60 (m, 5H), 1.57 – 1.07 (m, 13H), 1.07 – 0.94 (m, 5H), 0.89 (t, *J* = 6.7 Hz, 3H). ^{13}C NMR (101 MHz, $CDCl_3$) δ 57.89, 47.72, 42.87, 31.83, 30.43, 30.04, 27.92, 27.18, 26.80, 26.69, 26.53, 22.65, 16.78, 14.07. $[\alpha]_D^{20}$ =10.6 (c=1.0, EtOH). Enantiomeric excess was determined by chiral HPLC after the product was converted to the corresponding benzamide: IB-3, Hex/IPA = 90:10, 1 mL/min, 220 nm, 8.0 min, 9.8 min.

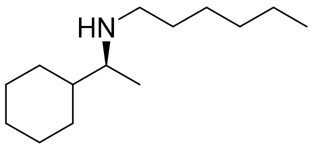

59

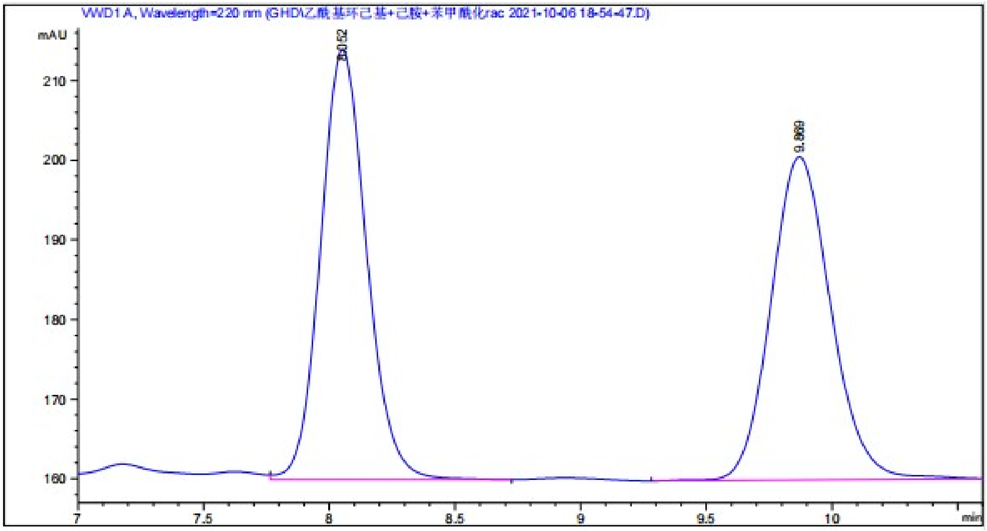

峰 #	保留时间 [min]	类型	峰宽 [min]	峰面积 [mAU*s]	峰高 [mAU]	峰面积 %
1	8.052	VB	0.1968	688.41479	53.96659	50.5310
2	9.869	BBA	0.2578	673.94666	40.56728	49.4690

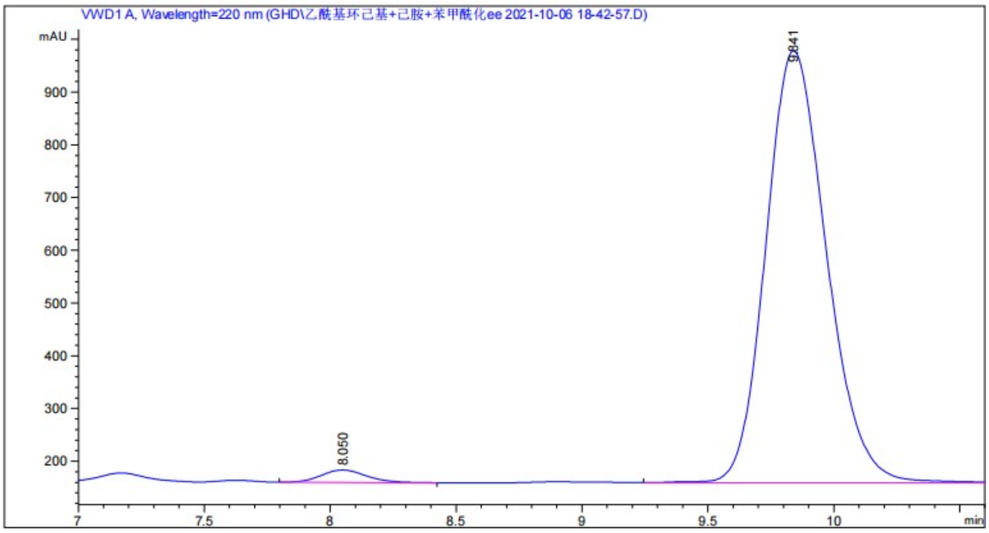

峰 #	保留时间 [min]	类型	峰宽 [min]	峰面积 [mAU*s]	峰高 [mAU]	峰面积 %
1	8.050	VB	0.1941	295.81909	23.61604	2.1126
2	9.841	VBA	0.2612	1.37070e4	819.06360	97.8874

Supplementary Figure 58. HPLC spectra for racemic and chiral **59**.

N-(4-methylpentan-2-yl)hexan-1-amine (60):[12] 95% yield, 93% ee, colorless oil.^{1}H NMR (400 MHz, Chloroform-*d*) δ 2.63 (dt, J = 11.2, 7.3 Hz, 1H), 2.46 (ddt, J = 26.7, 11.6, 6.7 Hz, 2H), 1.82 – 1.60 (m, 5H), 1.57 – 1.07 (m, 13H), 1.07 – 0.94 (m, 5H), 0.89 (t, J = 6.7 Hz, 3H). $[\alpha]_D^{20}$ =-13.8 (c=1.0, EtOH). Enantiomeric excess was determined by chiral GC after the product was converted to the corresponding acetamide: Astec Chiraldex G-DP, from 80 °C to 140 °C at 1 °C/min, 1 mL/min, 66.4 min, 66.9 min.

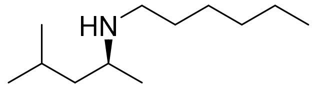

60

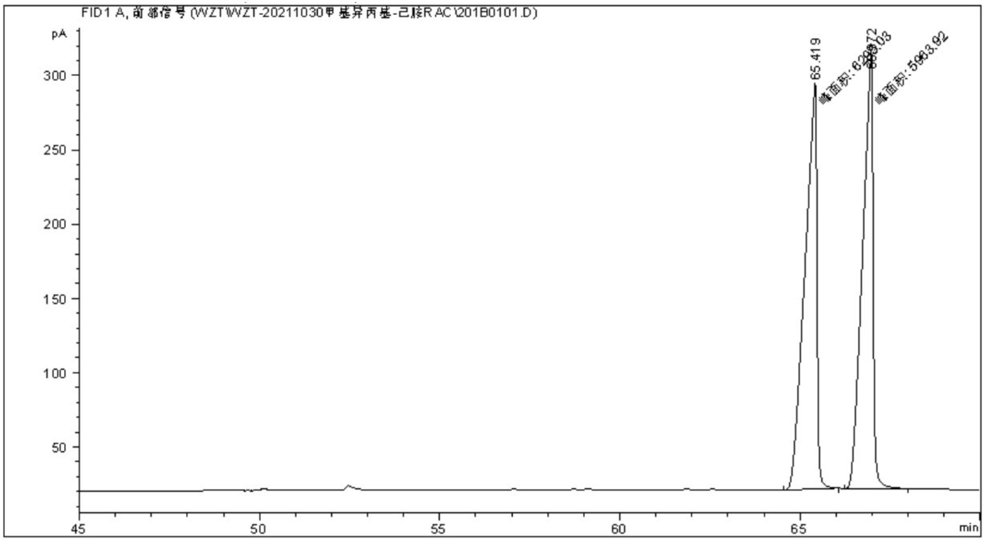

峰 #	保留时间 [min]	类型	峰宽 [min]	峰面积 [pA*s]	峰高 [pA]	峰面积 %
1	65.418	BB	0.2902	6279.96045	272.73575	51.52908
2	66.973	BB	0.2507	5907.25635	294.68112	48.47092

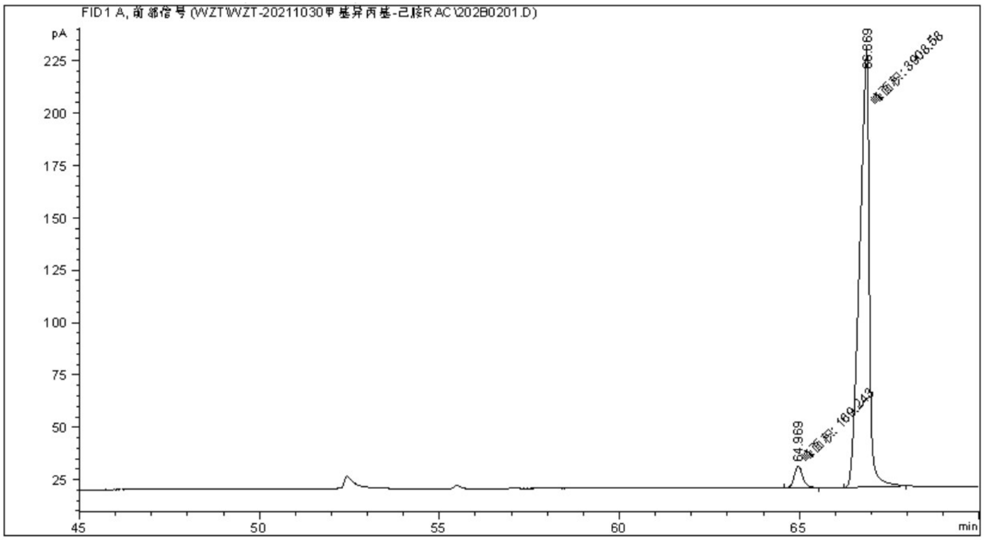

峰 #	保留时间 [min]	类型	峰宽 [min]	峰面积 [pA*s]	峰高 [pA]	峰面积 %
1	64.972	BB	0.1756	142.07343	9.75139	3.61118
2	66.870	BB	0.2334	3792.18921	207.28477	96.38882

Supplementary Figure 59. HPLC spectra for racemic and chiral **60**.

N-(3-methylbutan-2-yl)hexan-1-amine (61): 93 % yield, 92% ee, colorless oil. ^{1}H NMR (400 MHz, Chloroform-*d*) δ 2.67 – 2.57 (m, 1H), 2.56 – 2.38 (m, 2H), 1.69 (pd, *J* = 6.8, 4.9 Hz, 1H), 1.55 – 1.39 (m, 2H), 1.39 – 1.22 (m, 7H), 0.95 (d, *J* = 6.4 Hz, 3H), 0.87 (dd, *J* = 17.4, 6.8 Hz, 9H). ^{13}C NMR (101 MHz, CDCl$_3$) δ 58.36, 47.68, 32.12, 31.80, 30.41, 27.13, 22.60, 19.41, 17.07, 15.93, 14.00. $[\alpha]_D^{20}$ =+11.6 (c=0.5, EtOH). Enantiomeric excess was determined by chiral GC after the product was converted to the corresponding acetamide: Astec Chiraldex G-DP, from 80 °C to 140 °C at 1 °C/min, 1 mL/min, 62.6 min, 65.0 min.

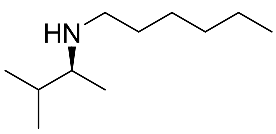

61

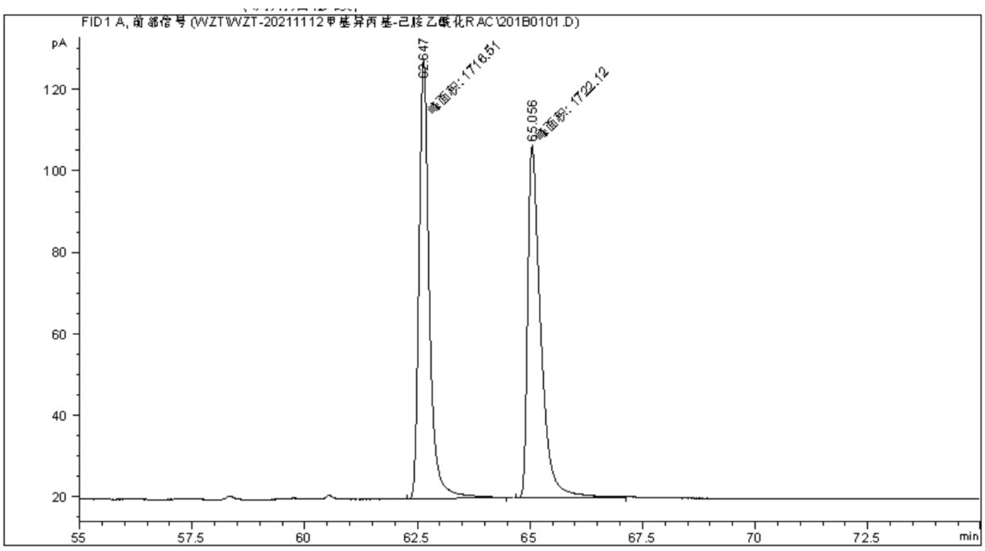

峰 #	保留时间 [min]	类型	峰宽 [min]	峰面积 [pA*s]	峰高 [pA]	峰面积 %
1	62.646	BB	0.1787	1560.76917	106.14487	50.27615
2	65.056	BB	0.2205	1543.62378	84.74599	49.72385

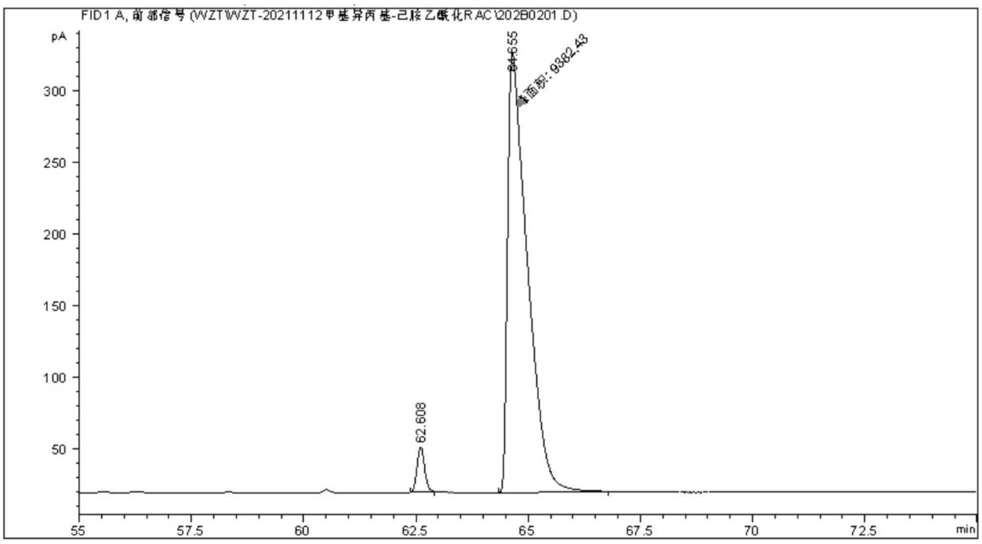

峰 #	保留时间 [min]	类型	峰宽 [min]	峰面积 [pA*s]	峰高 [pA]	峰面积 %
1	62.608	BB	0.1432	367.78674	31.54616	3.94691
2	64.655	BB	0.3445	8950.55371	305.25348	96.05309

Supplementary Figure 60. HPLC spectra for racemic and chiral **61**.

3-(1-(methylamino)ethyl)phenyl ethyl(methyl)carbamate (62):[13] 94% yield, 96% ee, colorless oil. ^{1}H NMR (400 MHz, Chloroform-*d*) δ 7.30 (t, *J* = 7.8 Hz, 1H), 7.12 (d, *J* = 7.6 Hz, 1H), 7.10 – 7.04 (m, 1H), 7.00 (d, *J* = 7.9 Hz, 1H), 3.64 (q, *J* = 6.6 Hz, 1H), 3.44 (dq, *J* = 25.6, 7.1 Hz, 2H), 3.30 (q, *J* = 7.2 Hz, 1H), 3.03 (d, *J* = 30.6 Hz, 3H), 2.84 (s, 1H), 2.79 (d, *J* = 4.7 Hz, 1H), 2.31 (s, 3H), 1.95 (d, *J* = 10.0 Hz, 2H), 1.35 (d, *J* = 6.6 Hz, 3H), 1.22 (dt, *J* = 21.2, 7.1 Hz, 3H), 1.10 (t, *J* = 7.1 Hz, 1H). $[\alpha]_D^{20}$ =-45.8 (c=0.5, EtOH). Enantiomeric excess was determined by chiral HPLC after the product was converted to the corresponding acetamide: AD -H, Hex/IPA = 90:10, 1 mL/min, 220 nm, 10.3 min, 12.4 min.

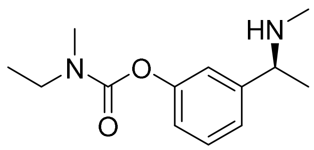

62

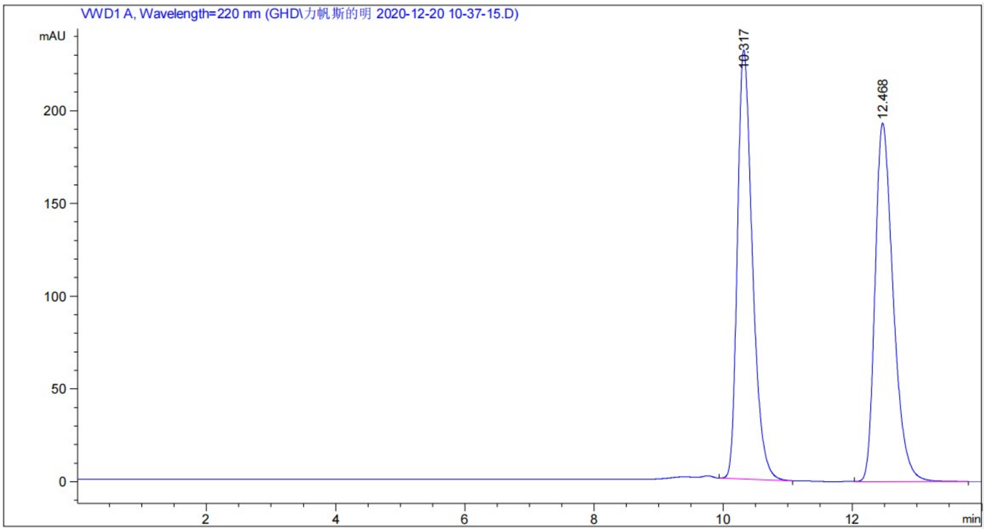

峰 #	保留时间 [min]	类型	峰宽 [min]	峰面积 [mAU*s]	峰高 [mAU]	峰面积 %
1	10.317	BB	0.2469	3725.52344	231.29913	49.6633
2	12.468	VB	0.3001	3776.03149	193.14458	50.3367

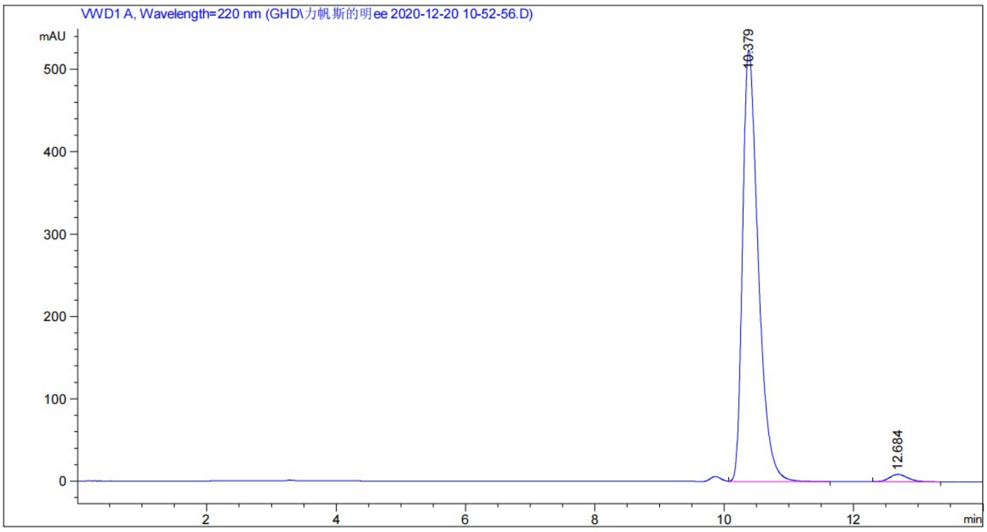

峰 #	保留时间 [min]	类型	峰宽 [min]	峰面积 [mAU*s]	峰高 [mAU]	峰面积 %
1	10.379	VV	0.2585	8822.83105	523.82611	98.0552
2	12.684	VB	0.3028	174.99190	8.92248	1.9448

Supplementary Figure 61. HPLC spectra for racemic and chiral **62**.

(*S*)-3-(2-chlorophenyl)-N-(1-(3-methoxyphenyl)ethyl)-N-methylpropan-1-amine (63):[4] ^{1}H NMR (400 MHz, Chloroform-*d*) δ 7.27 (dd, *J* = 23.2, 7.5 Hz, 3H), 7.20 – 7.10 (m, 3H), 7.03 (dd, *J* = 7.9, 1.7 Hz, 1H), 6.95 (s, 1H), 3.99 (q, *J* = 6.6 Hz, 1H), 2.70 – 2.43 (m, 4H), 2.29 (d, *J* = 1.9 Hz, 6H), 1.78 (p, *J* = 7.2 Hz, 2H), 1.54 – 1.43 (m, 1H), 1.28 (d, *J* = 6.6 Hz, 3H). $[\alpha]_D^{20}$ =-41.3 (c=1.0, EtOH). Enantiomeric excess was determined by chiral HPLC after the product was converted to the corresponding acetamide: IB-3, Hex/IPA = 95:5, 1 mL/min, 220 nm, 14.3 min, 15.5 min.

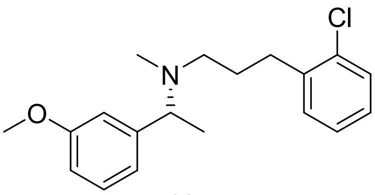

63

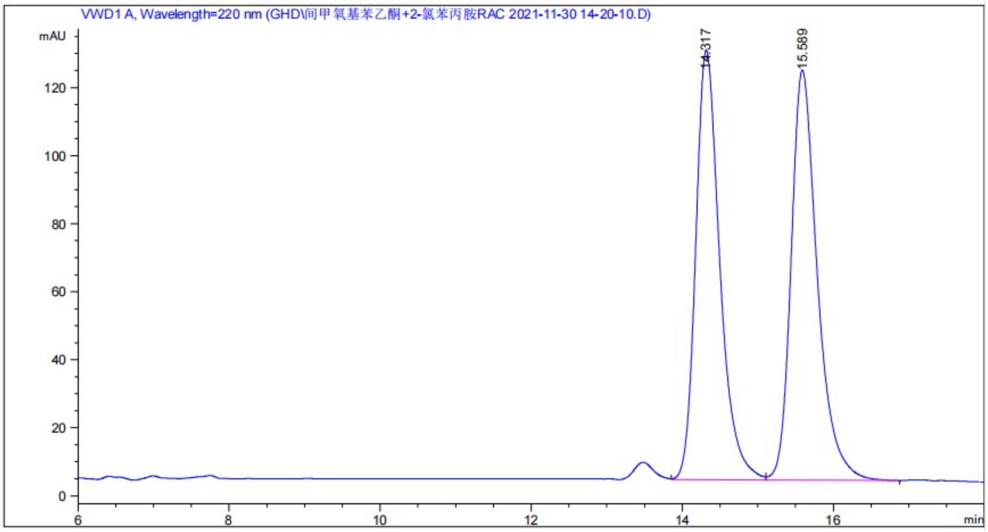

峰 #	保留时间 [min]	类型	峰宽 [min]	峰面积 [mAU*s]	峰高 [mAU]	峰面积 %
1	14.317	VV	0.3390	2803.52368	126.30320	49.0419
2	15.589	VB	0.3659	2913.06543	120.57419	50.9581

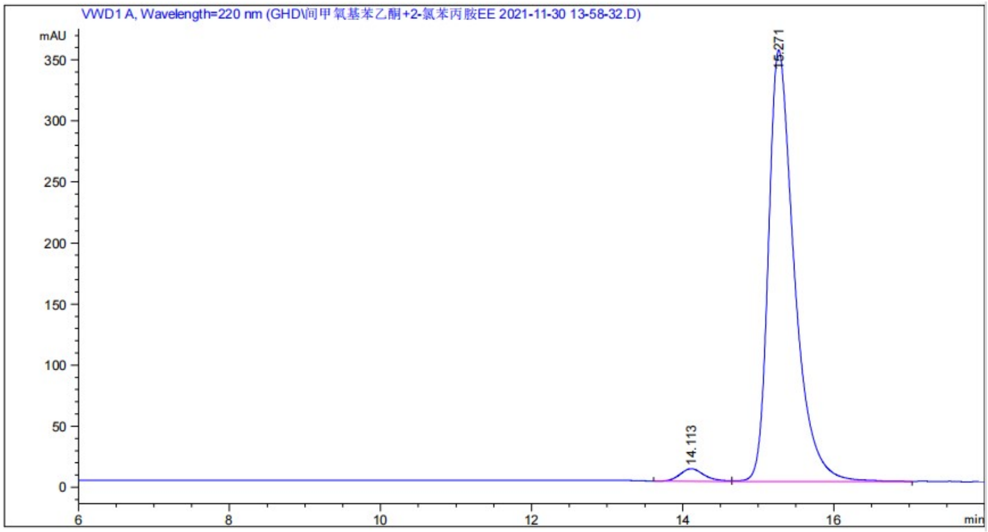

峰 #	保留时间 [min]	类型	峰宽 [min]	峰面积 [mAU*s]	峰高 [mAU]	峰面积 %
1	14.113	BV	0.3385	228.71181	10.28327	2.6498
2	15.271	VV	0.3585	8402.41406	353.27173	97.3502

Supplementary Figure 62. HPLC spectra for racemic and chiral **63**.

***N*-(1-(naphthalen-1-yl)ethyl)-3-(3-(trifluoromethyl)phenyl)propan-1-amine (64)**:[4] 95% yield, 98% ee, colorless oil.

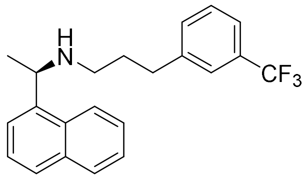

64

^{1}H NMR (500 MHz, Chloroform-*d*) δ 8.30 (d, *J* = 8.3 Hz, 1H), 7.96 (d, *J* = 8.0 Hz, 1H), 7.84 (d, *J* = 8.2 Hz, 1H), 7.75 (d, *J* = 7.2 Hz, 1H), 7.65 – 7.48 (m, 5H), 7.40 (dd, *J* = 14.9, 7.7 Hz, 2H), 4.70 (t, *J* = 6.7 Hz, 1H), 2.75 (tdd, *J* = 29.0, 12.8, 5.7 Hz, 4H), 1.91 (q, *J* = 7.4 Hz, 2H), 1.59 (d, *J* = 6.5 Hz, 3H), 1.48 (s, 1H); ^{13}C NMR (125 MHz, $CDCl_3$): δ 143.1, 141.2, 134.1, 131.8, 131.4, 130.8, 130.5, 129.1, 128.7, 127.3, 125.8, 125.7, 125.1 (q, *J* = 3.7 Hz), 123.0, 122.8, 122.7 (m), 53.8, 47.3, 33.5, 31.9, 23.6. $[\alpha]_D^{20}$ =-15.6 (c=0.5, EtOH). Enantiomeric excess was determined by chiral HPLC after the product was converted to the corresponding acetamide: AS-H, Hex/IPA = 90:10, 1 mL/min, 220 nm, 9.2 min,11.6 min.

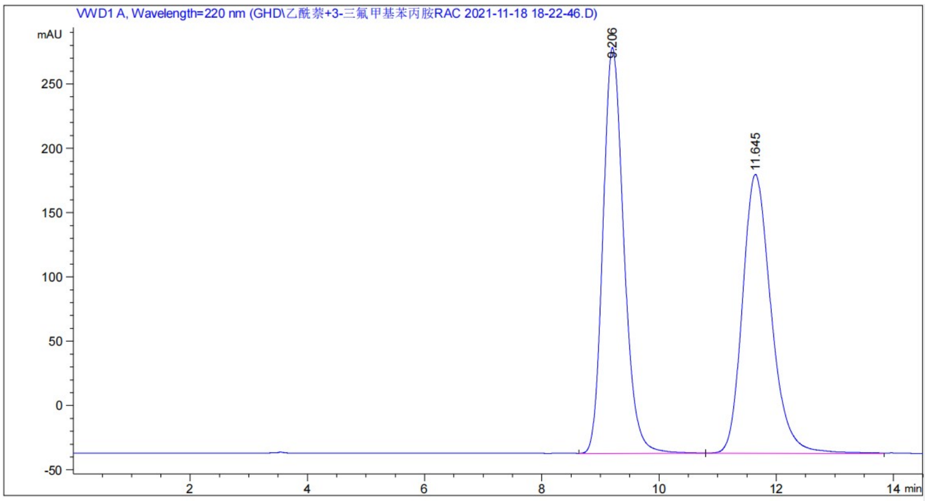

峰 #	保留时间 [min]	类型	峰宽 [min]	峰面积 [mAU*s]	峰高 [mAU]	峰面积 %
1	9.206	BB	0.3809	7786.93604	315.53568	51.4207
2	11.645	BV	0.5209	7356.64844	216.81020	48.5793

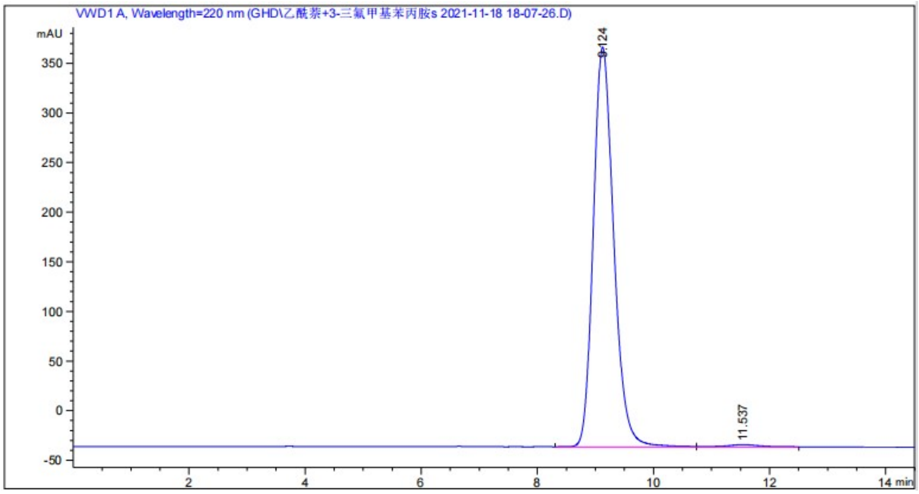

峰 #	保留时间 [min]	类型	峰宽 [min]	峰面积 [mAU*s]	峰高 [mAU]	峰面积 %
1	9.124	BV	0.3769	9836.42773	402.82025	98.9512
2	11.537	VV	0.5625	104.25516	2.49009	1.0488

Supplementary Figure 63. HPLC spectra for racemic and chiral **64**.

3,3-diphenyl-N-(1-phenylethyl)propan-1-amine (65):[5] 95% yield, 96% ee, colorless oil. ^{1}H NMR (500 MHz, Chloroform-*d*) δ 7.37 – 7.17 (m, 15H), 4.03 (t, *J* = 7.8 Hz, 1H), 3.74 (q, *J* = 6.6 Hz, 1H), 2.59 – 2.43 (m, 2H), 2.35 – 2.17 (m, 2H), 1.54 (s, 1H), 1.35 (d, *J* = 6.7 Hz, 3H). $[\alpha]_D^{20}$ =-39.2 (c=1.0, EtOH). Enantiomeric excess was determined by chiral HPLC after the product was converted to the corresponding acetamide: AD-H, Hex/IPA = 90:10, 1 mL/min, 220 nm, 13.0 min, 13.8 min.

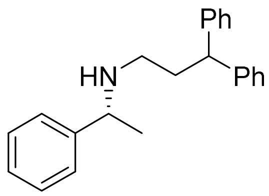

65

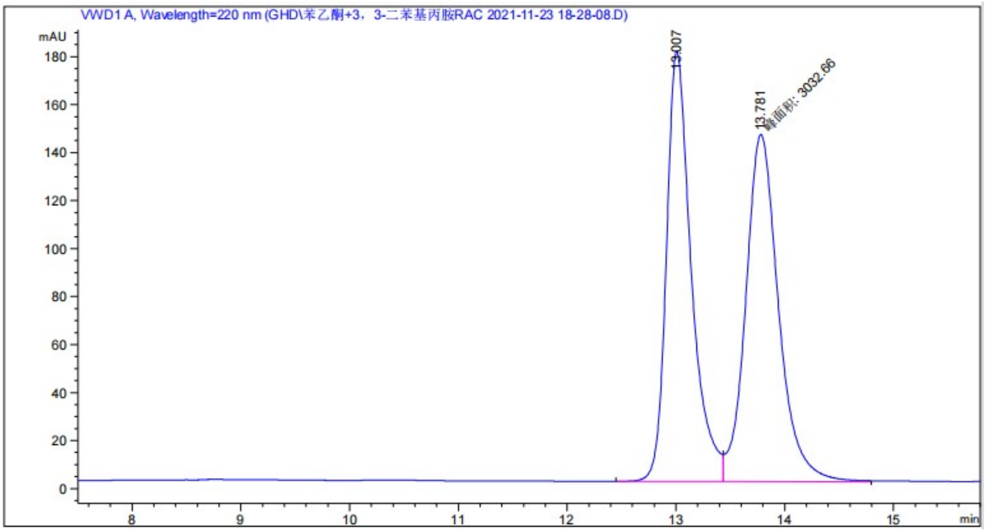

峰 #	保留时间 [min]	类型	峰宽 [min]	峰面积 [mAU*s]	峰高 [mAU]	峰面积 %
1	13.007	VV	0.2381	2860.43872	179.35573	48.5388
2	13.781	MF	0.3495	3032.66284	144.63979	51.4612

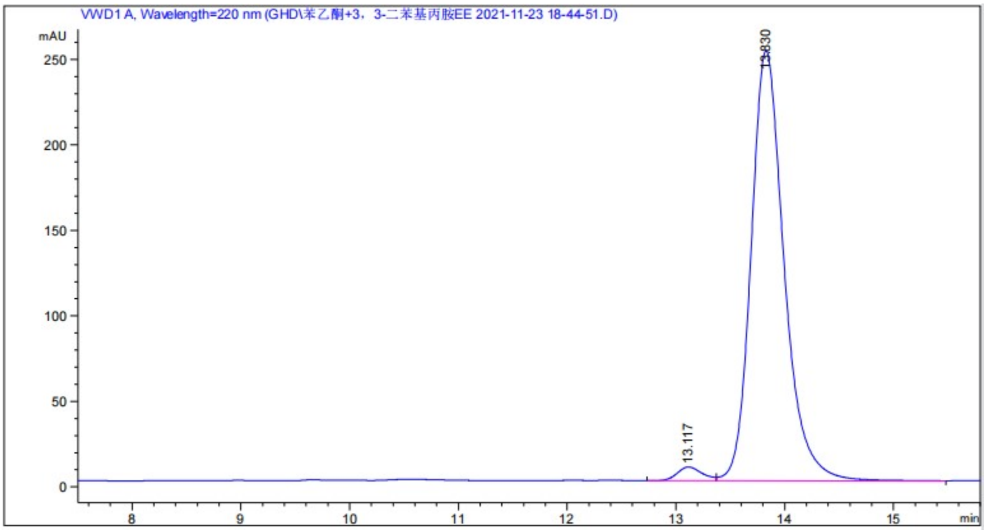

峰 #	保留时间 [min]	类型	峰宽 [min]	峰面积 [mAU*s]	峰高 [mAU]	峰面积 %
1	13.117	BV	0.2426	129.22226	8.08057	2.3763
2	13.830	VB	0.3222	5308.71094	251.60071	97.6237

Supplementary Figure 64. HPLC spectra for racemic and chiral **65**.

1-(3,5-bis(trifluoromethyl)phenyl)-N-methylethan-1-amine (66):[14] 95% yield, 96% ee, brown oil. ^{1}H NMR (400 MHz, Chloroform-*d*) δ 7.81 (s, 2H), 7.77 (s, 1H), 3.80 (q, *J* = 6.6 Hz, 1H), 2.32 (s, 3H), 1.43 (d, *J* = 3.5 Hz, 1H), 1.37 (d, *J* = 6.8 Hz, 3H). $[\alpha]_D^{20}$ =-33.9 (c=1.0, EtOH). Enantiomeric excess was determined by chiral HPLC after the product was converted to the corresponding acetamide: IB-3, Hex/IPA = 90:10, 1 mL/min, 220 nm, 5.2 min, 5.9 min

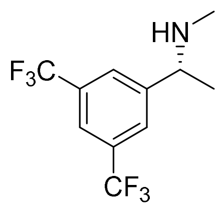

66

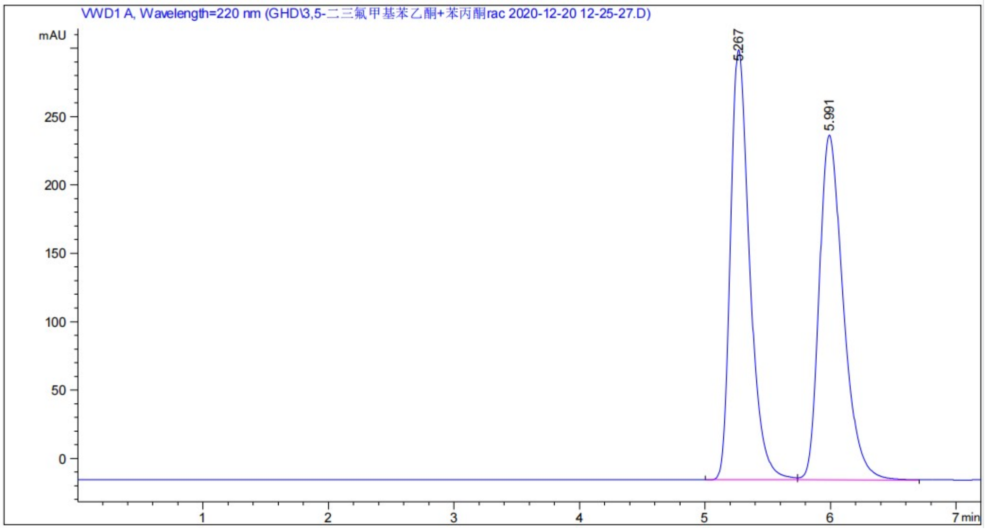

峰 #	保留时间 [min]	类型	峰宽 [min]	峰面积 [mAU*s]	峰高 [mAU]	峰面积 %
1	5.267	BV	0.1620	3344.10181	314.50958	49.8979
2	5.991	VV	0.2034	3357.79224	252.11346	50.1021

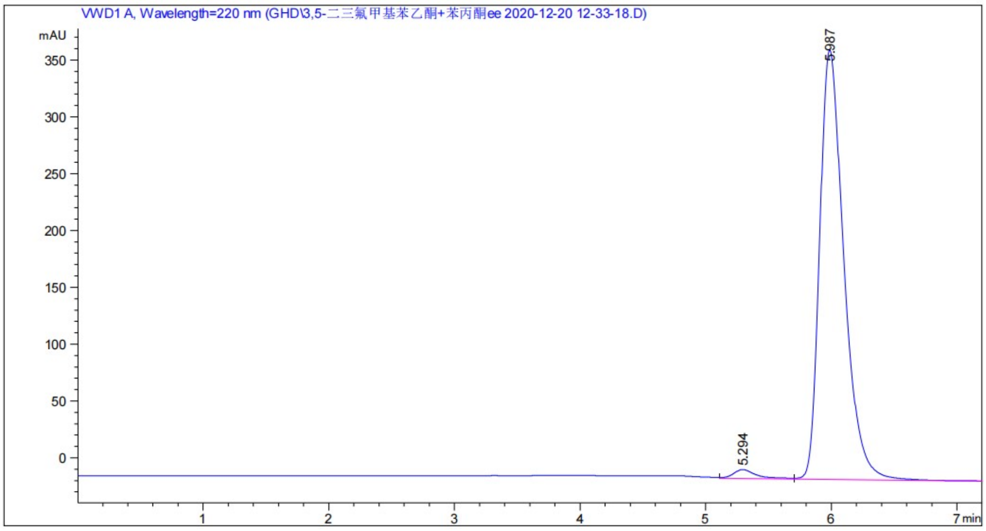

峰 #	保留时间 [min]	类型	峰宽 [min]	峰面积 [mAU*s]	峰高 [mAU]	峰面积 %
1	5.294	VV	0.1871	100.03541	7.82744	1.9366
2	5.987	VB	0.2044	5065.38770	377.79309	98.0634

Supplementary Figure 65. HPLC spectra for racemic and chiral orvepitant intermediate.

3. Supplementary Result and Discussion

DFT calculations

All calculations were performed using Gaussian 09 software[14] with the density functional theory (DFT). Optimizations of intermediates and transition states were carried out at the B3LYP-D3 level of theory with the 6-311G(d,p) basis set for C, P, N, O, F, Cl, H and LANL2DZ for Ir. The vibrational frequencies were also computed at the same level so as to confirm the stationary points as transition states (one and only one imaginary frequency) or minima (zero imaginary frequency). Single point energy calculations were performed on optimized geometries in EthylEthanoate solvent using the IEFPCM model at the B3LYP-D3 level of theory. For all nonmetallic atoms, the 6-311++G(d,p) basis set was adopted. For iridium, the LANL2DZ was employed, which was augmented with one f-polarization function (0.938). The Gibbs Free Energies of the optimized structures were obtained from the thermal correction to Gibbs Free Energy in frequency analysis and the single point energy.

(1) Calculated Gibbs Free Energy profiles in EtOAc and CF_3CH_2OH:

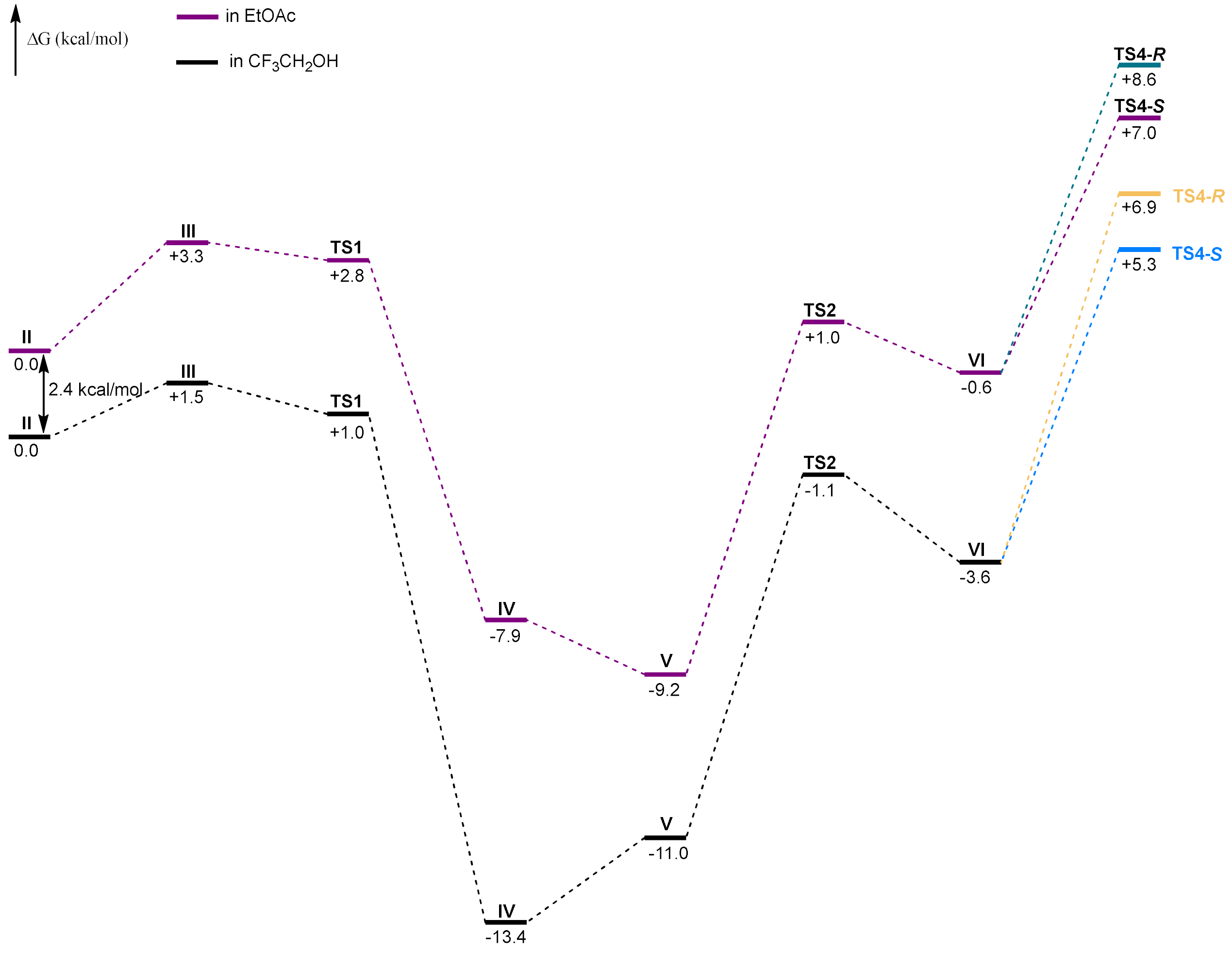

Supplementary Figure 66. Calculated Gibbs Free Energy profiles in EtOAc and CF_3CH_2OH.

(2) Competing reaction pathways: “outer-sphere” H-addition and “inner-sphere” H-addition

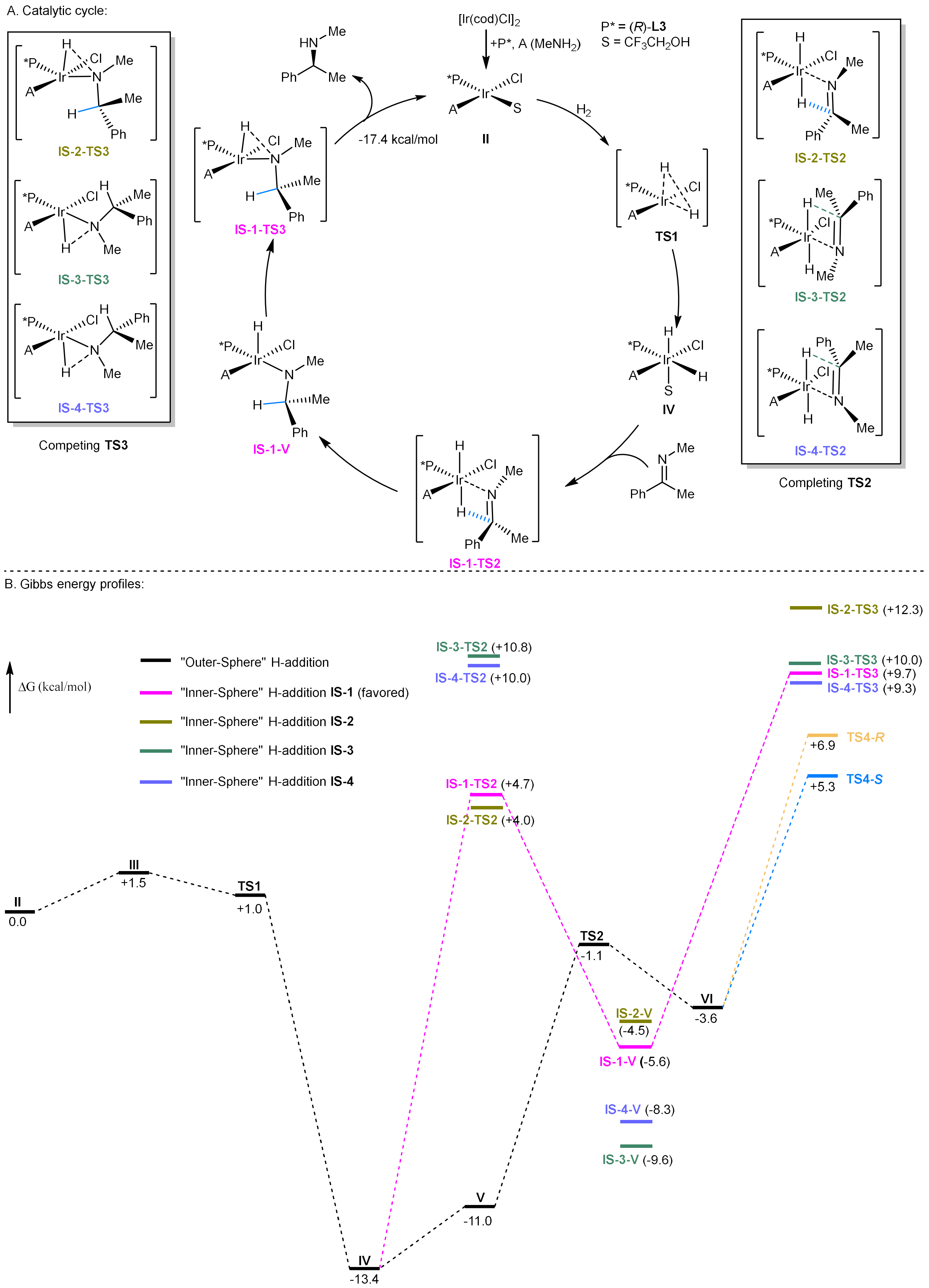

Supplementary Figure 67. Competing reaction pathways. a “Inner-sphere” H-addition pathways. b The Gibbs energy comparison for “inner-sphere” H-addition and “outer-sphere” H-addition.

4. Supplementary Figures

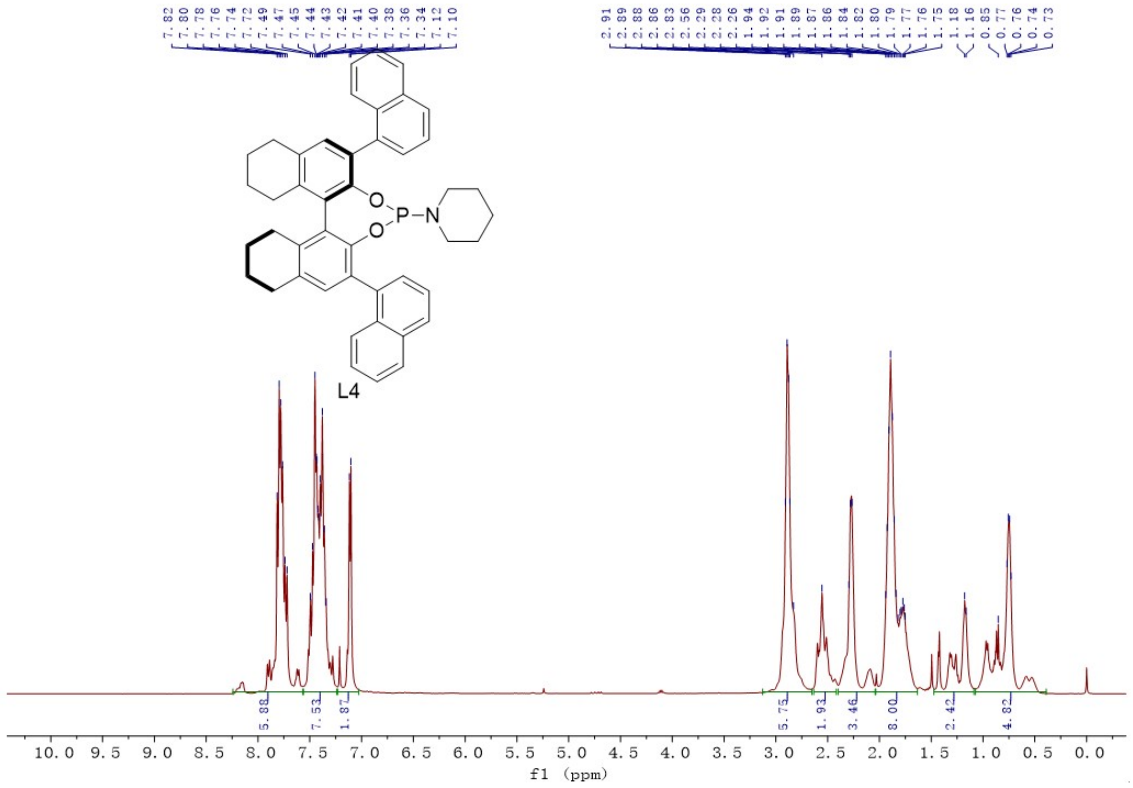

Supplementary Figure 68. ^{1}H NMR spectrum of **L4** in $CDCl_3$.

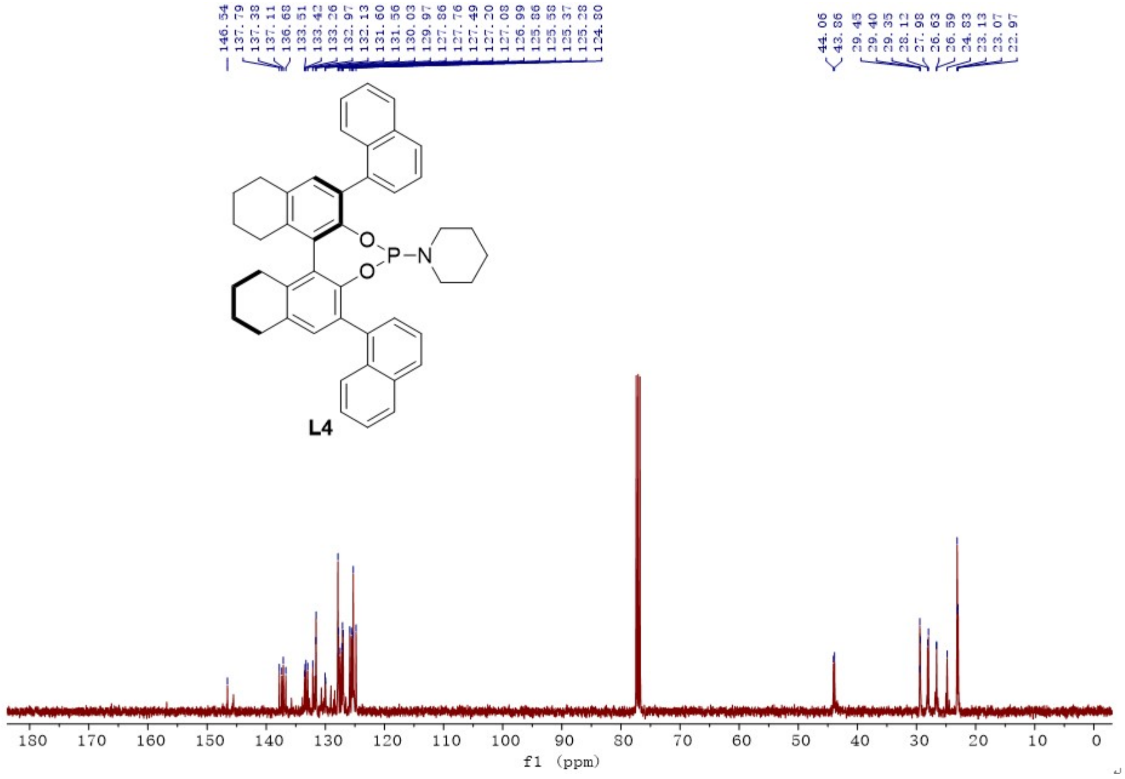

Supplementary Figure 69. ^{13}C NMR spectrum of **L4** in $CDCl_3$.

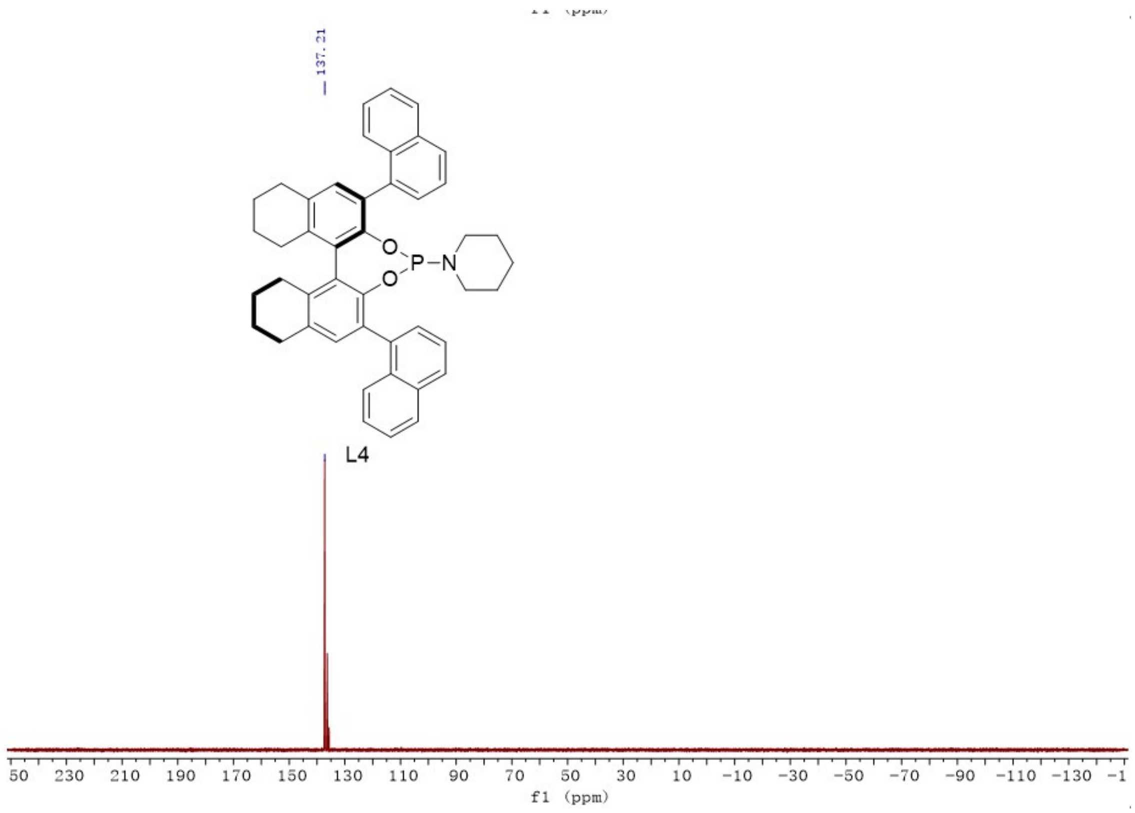

Supplementary Figure 70. ^{31}P NMR spectrum of **L4** in $CDCl_3$.

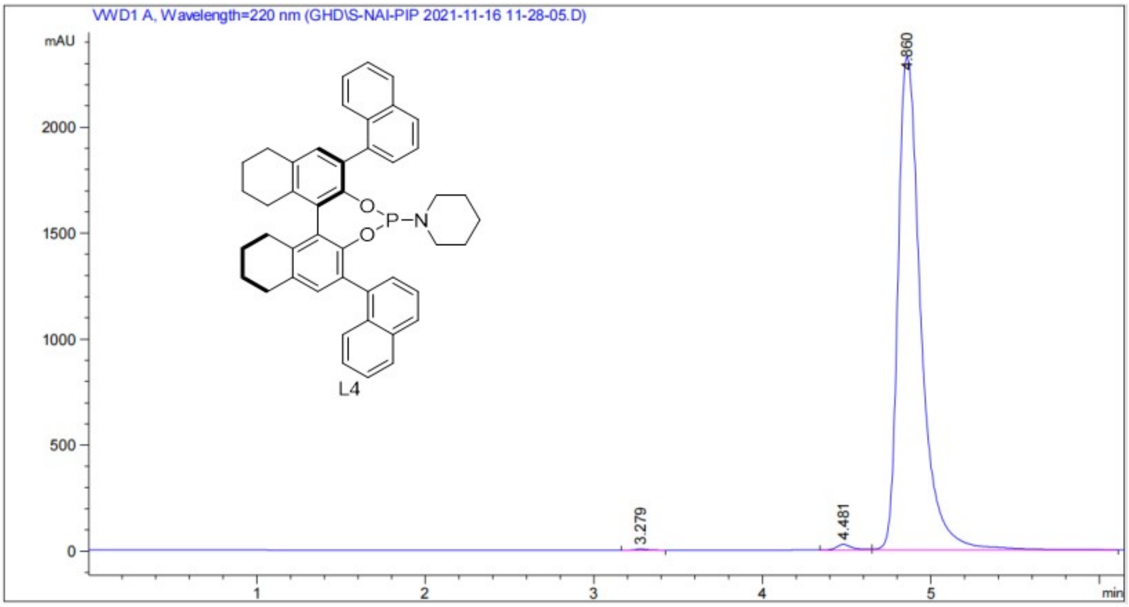

峰 #	保留时间 [min]	类型	峰宽 [min]	峰面积 [mAU*s]	峰高 [mAU]	峰面积 %
1	3.279	VV	0.0760	31.56384	6.14001	0.1422
2	4.481	VV	0.1023	188.47870	27.04276	0.8489
3	4.860	VB	0.1432	2.19838e4	2326.83081	99.0090

Supplementary Figure 71. HPLC spectra for chiral **L4**.

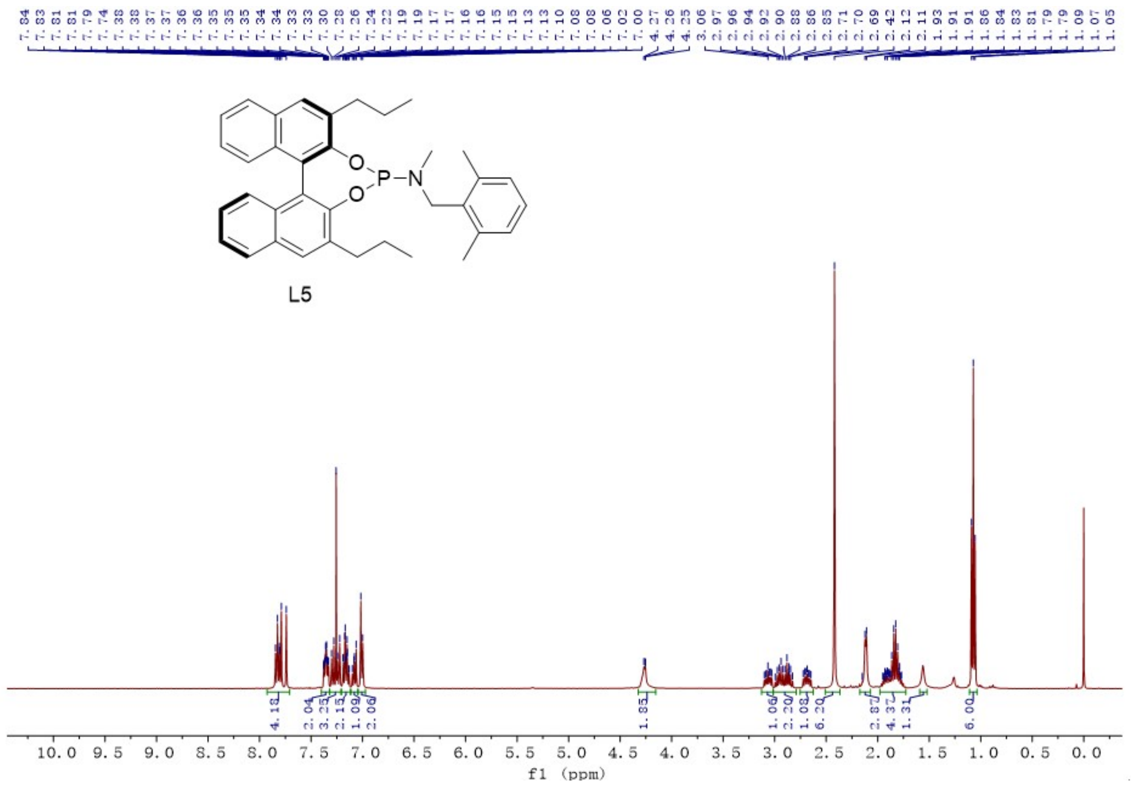

Supplementary Figure 72. ^{1}H NMR spectrum of **L5** in $CDCl_3$.

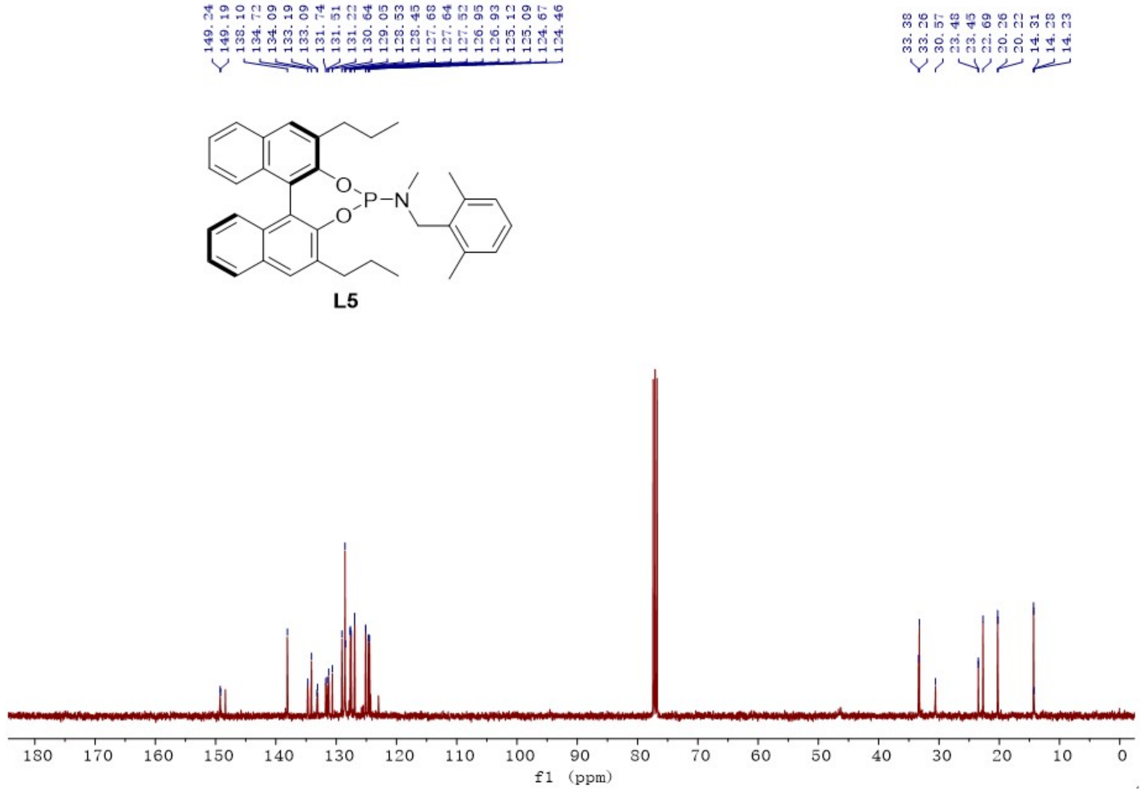

Supplementary Figure 73. ^{13}C NMR spectrum of **L5** in $CDCl_3$.

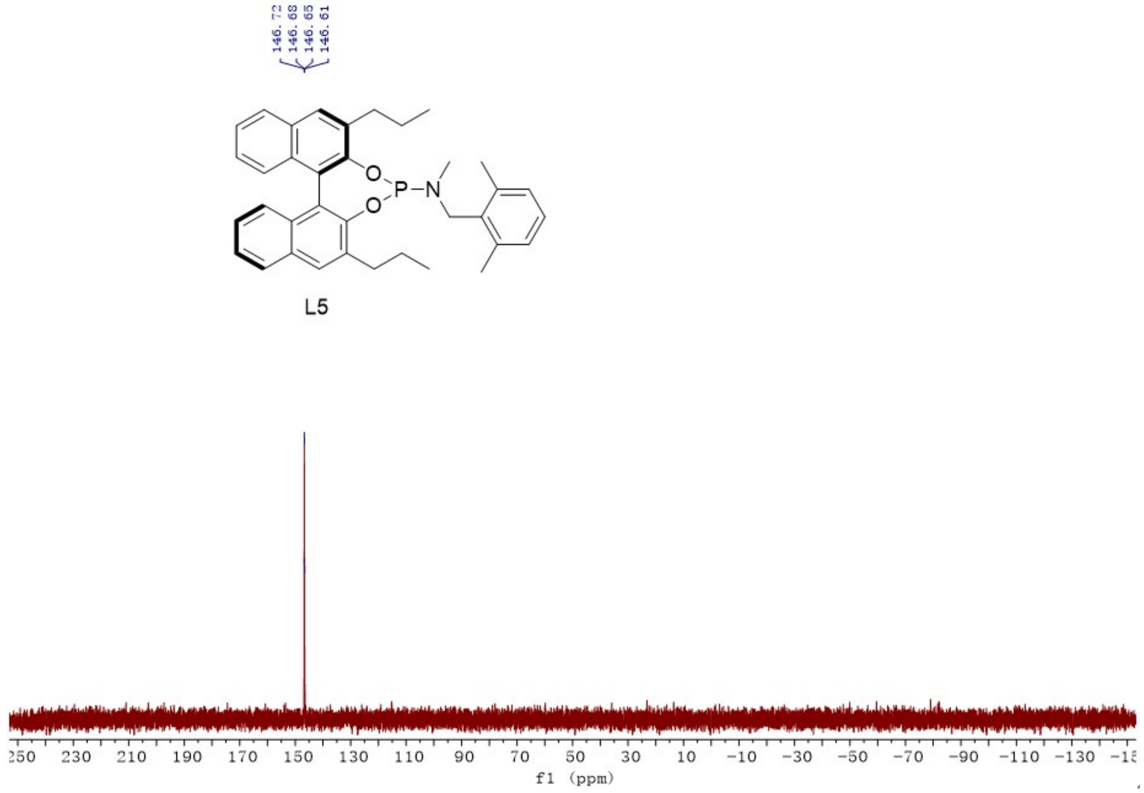

Supplementary Figure 74. ^{31}P NMR spectrum of **L5** in $CDCl_3$.

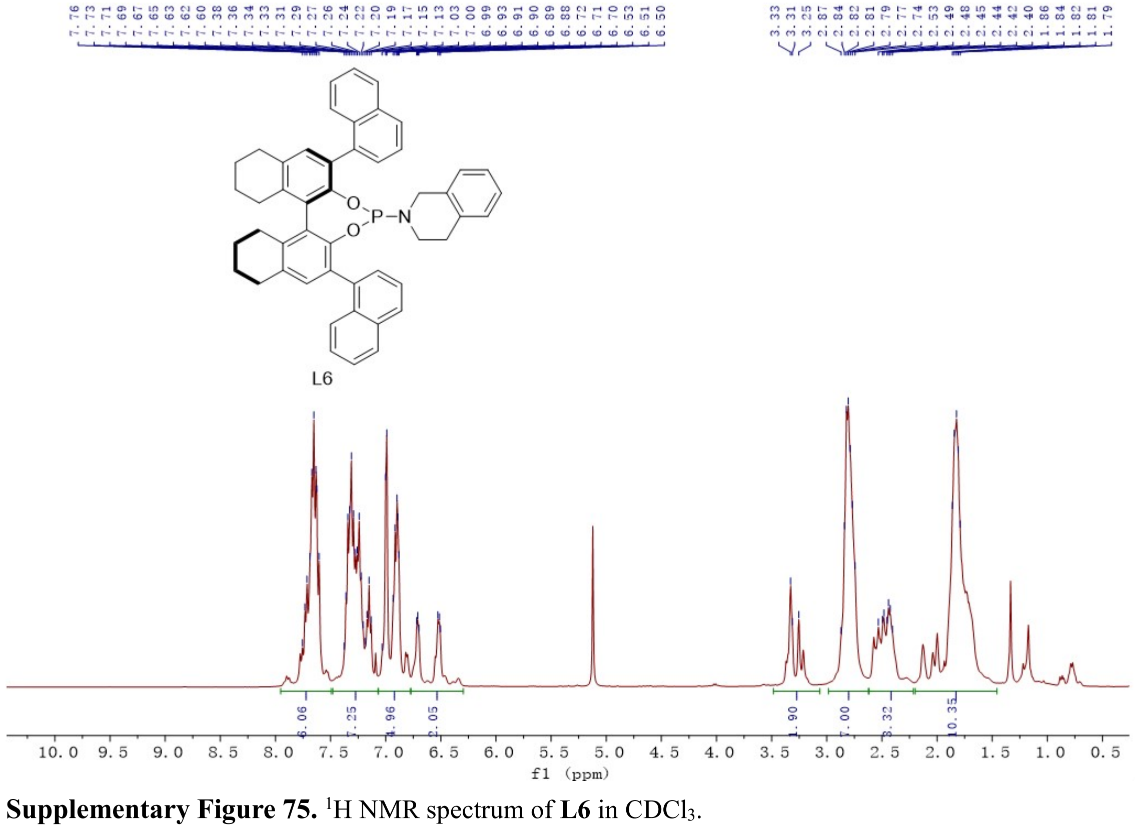

Supplementary Figure 75. ^{1}H NMR spectrum of **L6** in $CDCl_3$.

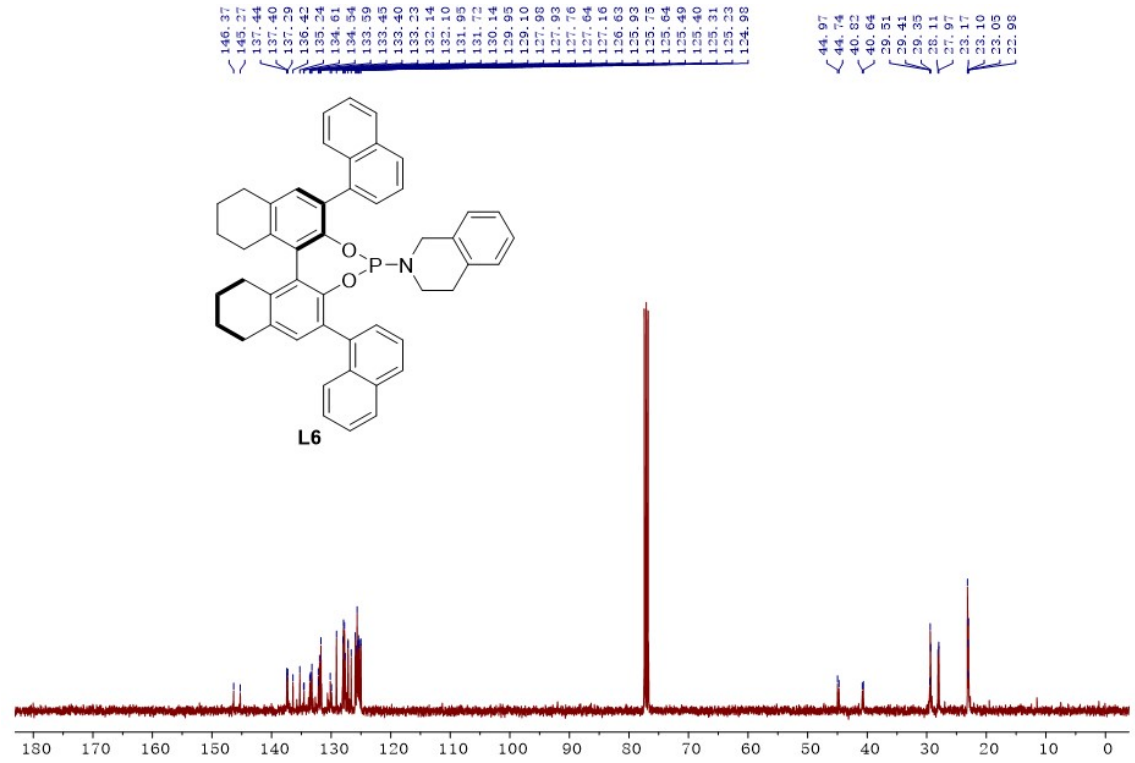

Supplementary Figure 76. ^{13}C NMR spectrum of **L4** in $CDCl_3$.

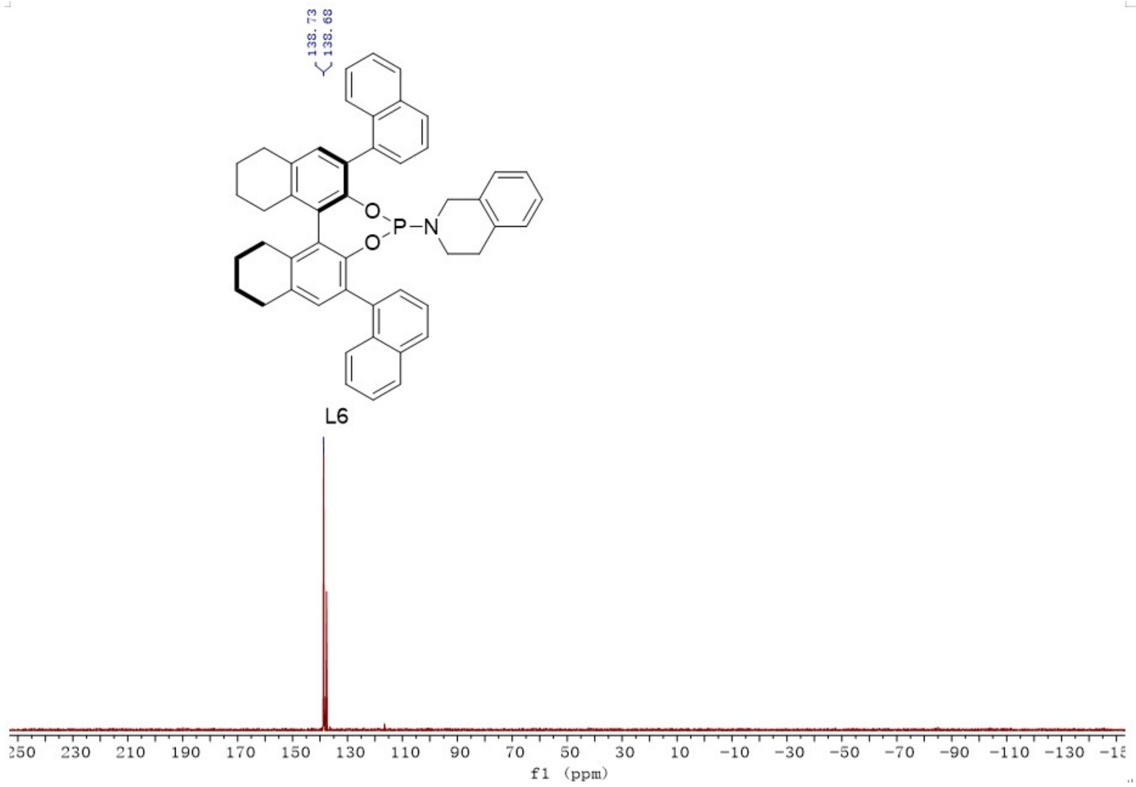

Supplementary Figure 77. ^{31}P NMR spectrum of **L6** in $CDCl_3$.

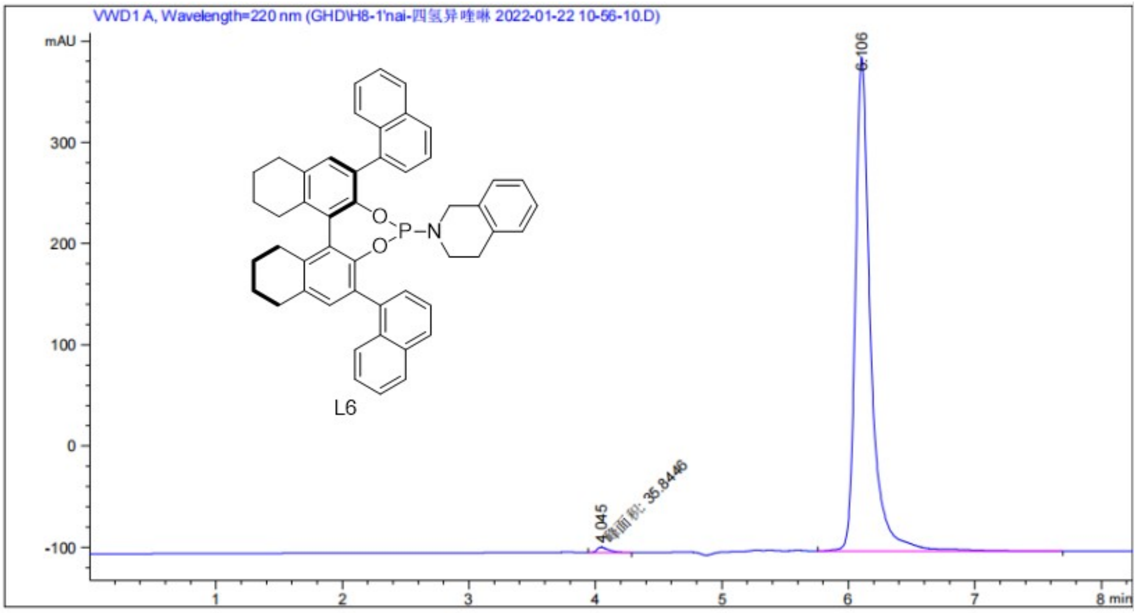

峰 #	保留时间 [min]	类型	峰宽 [min]	峰面积 [mAU*s]	峰高 [mAU]	峰面积 %
1	4.045	MM	0.1078	35.84462	5.54306	0.8635
2	6.106	BB	0.1244	4115.22363	487.80725	99.1365

Supplementary Figure 78. HPLC spectra for chiral **L6**.

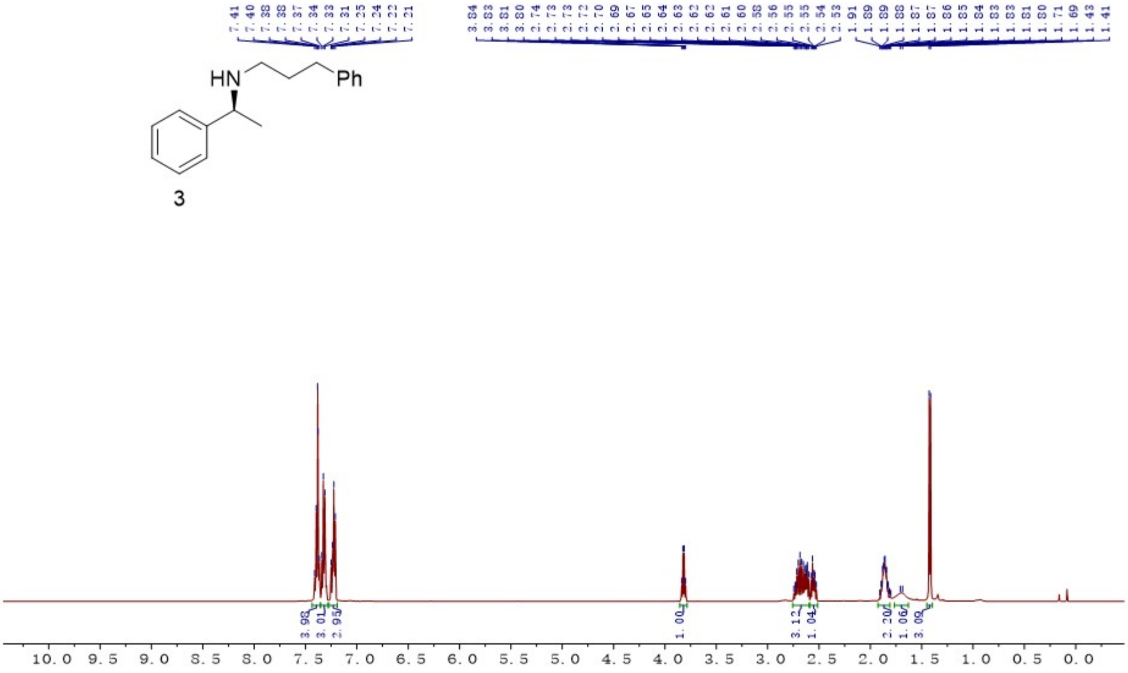

Supplementary Figure 79. ^{1}H NMR spectrum of **3** in $CDCl_3$.

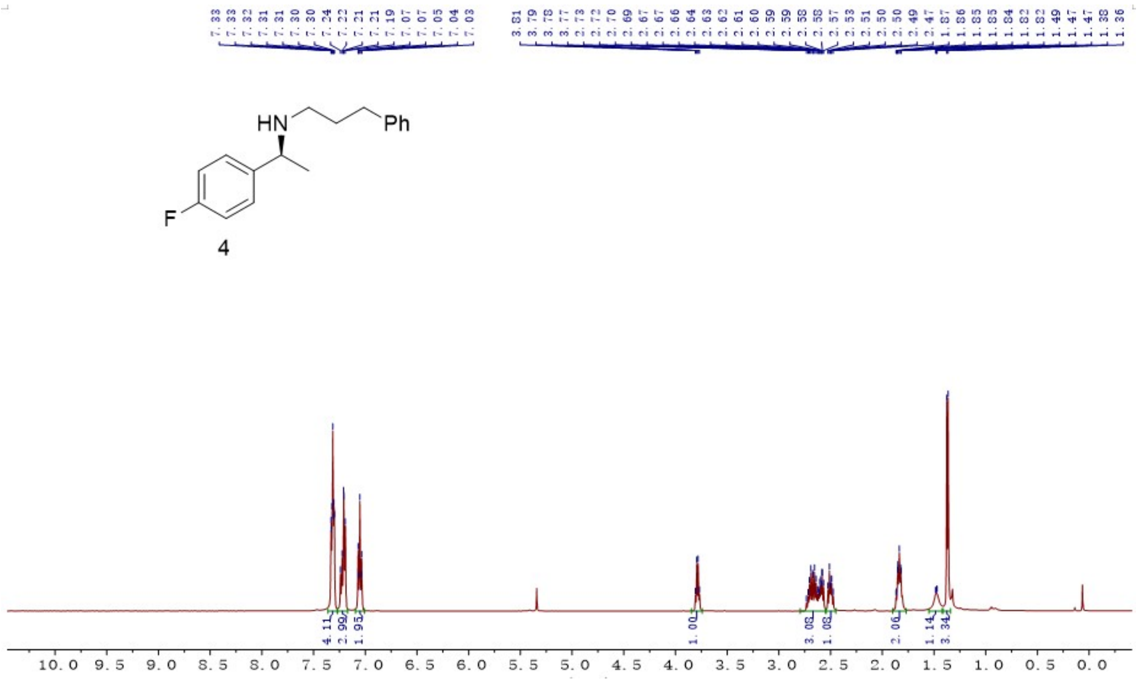

Supplementary Figure 80. ^{1}H NMR spectrum of **3** in $CDCl_3$.

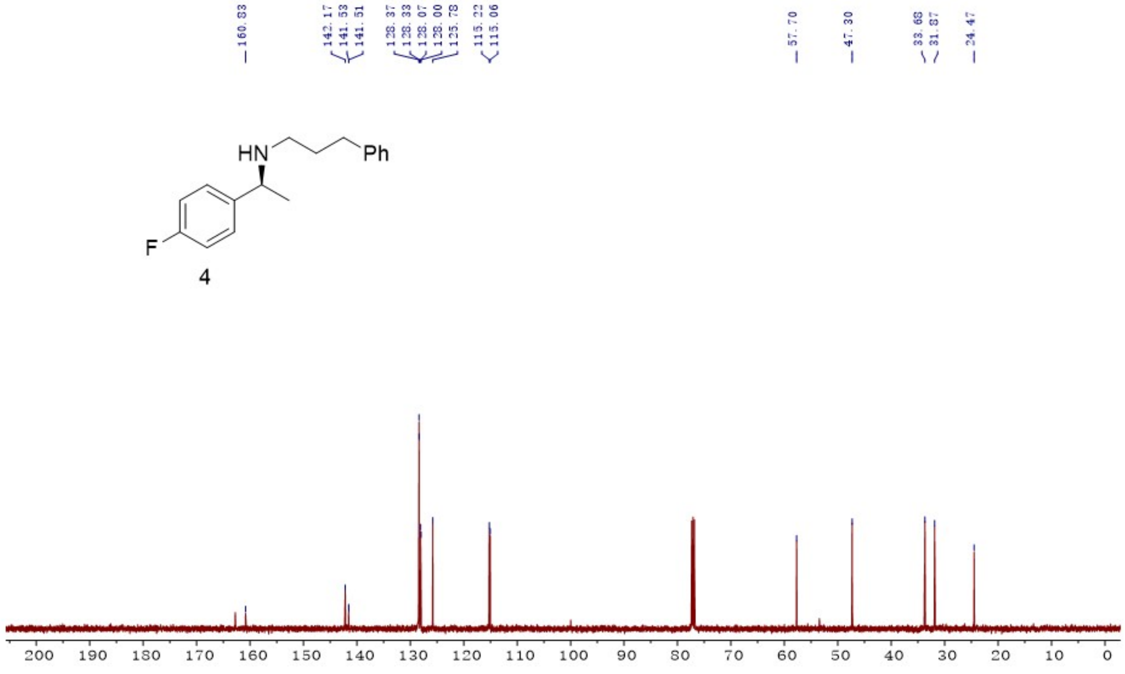

Supplementary Figure 81. ^{13}C NMR spectrum of **3** in $CDCl_3$.

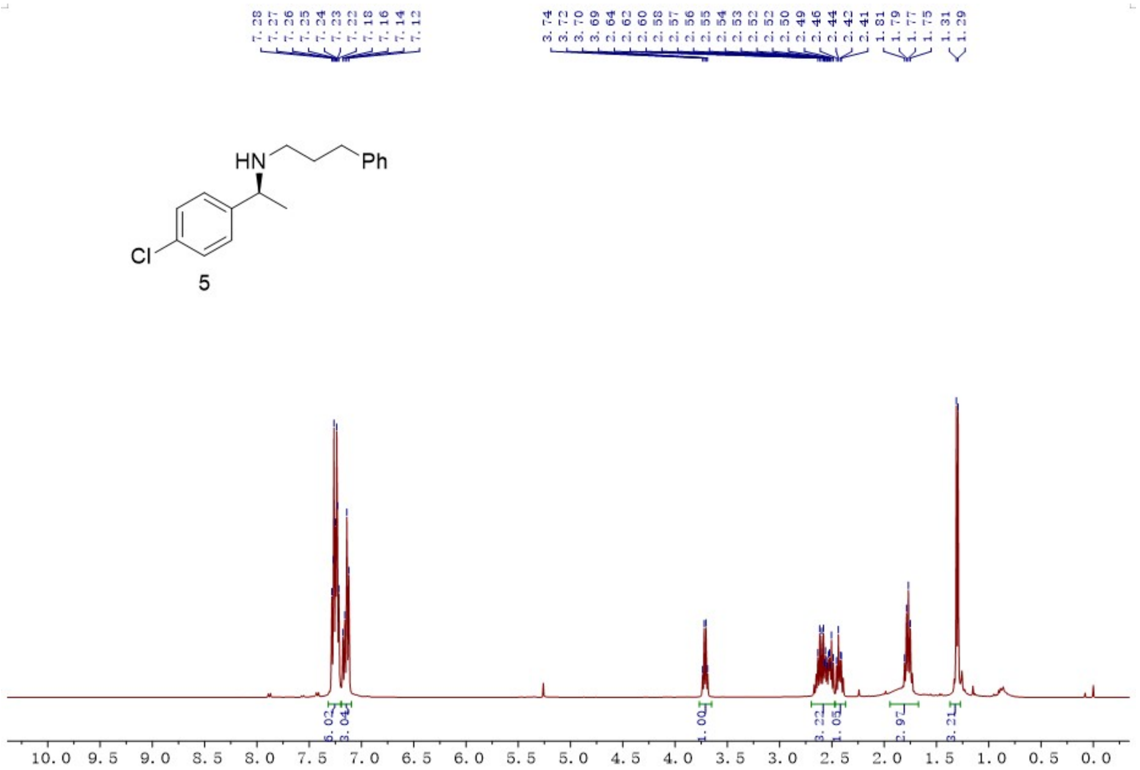

Supplementary Figure 82. ^{1}H NMR spectrum of **5** in $CDCl_3$.

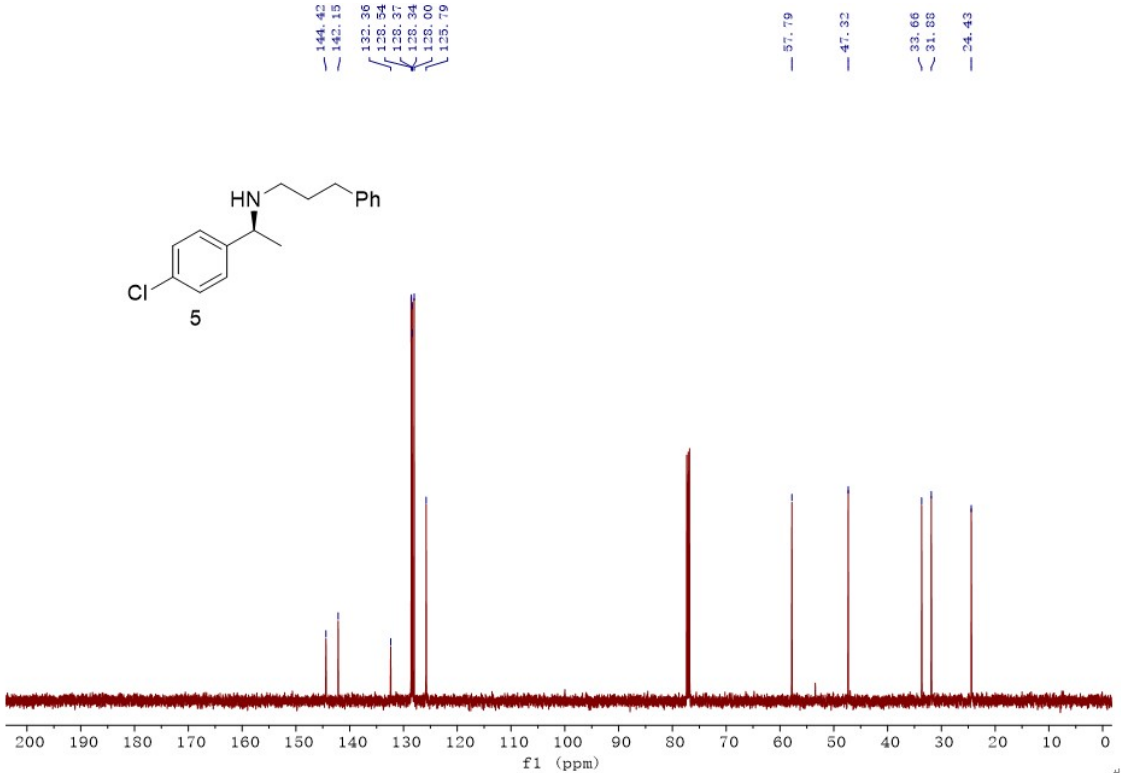

Supplementary Figure 83. ^{13}C NMR spectrum of **5** in $CDCl_3$.

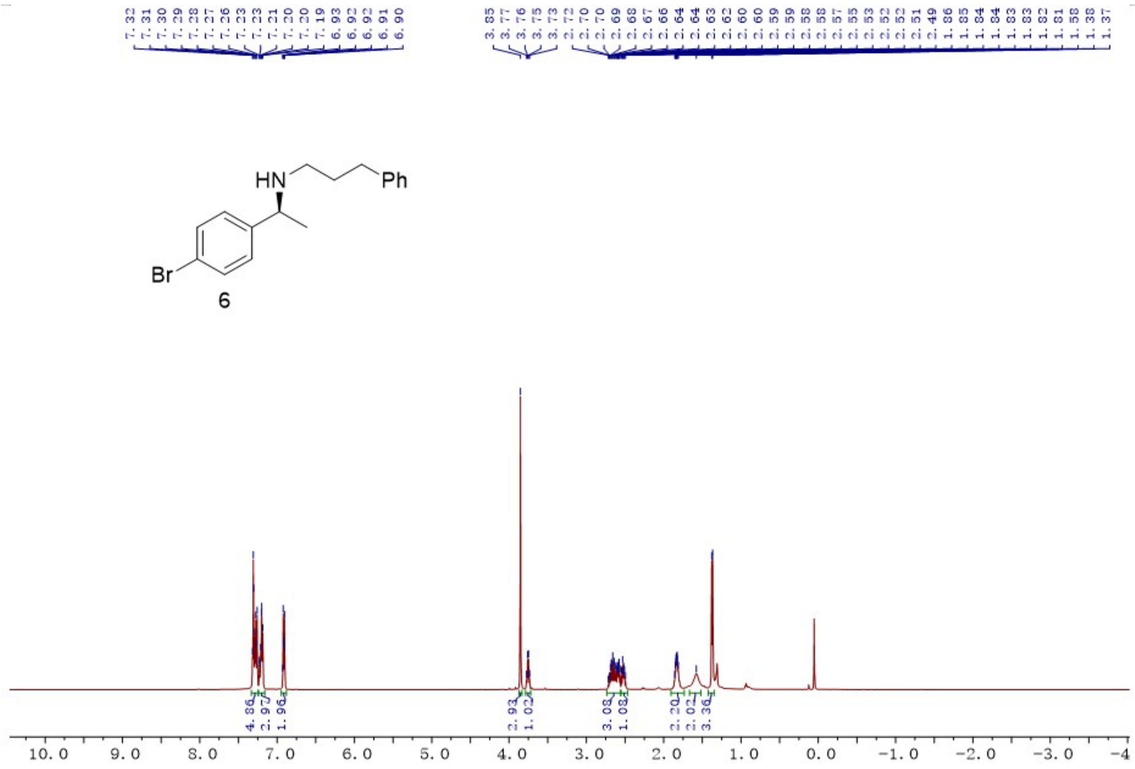

Supplementary Figure 84. ^{1}H NMR spectrum of **6** in $CDCl_3$.

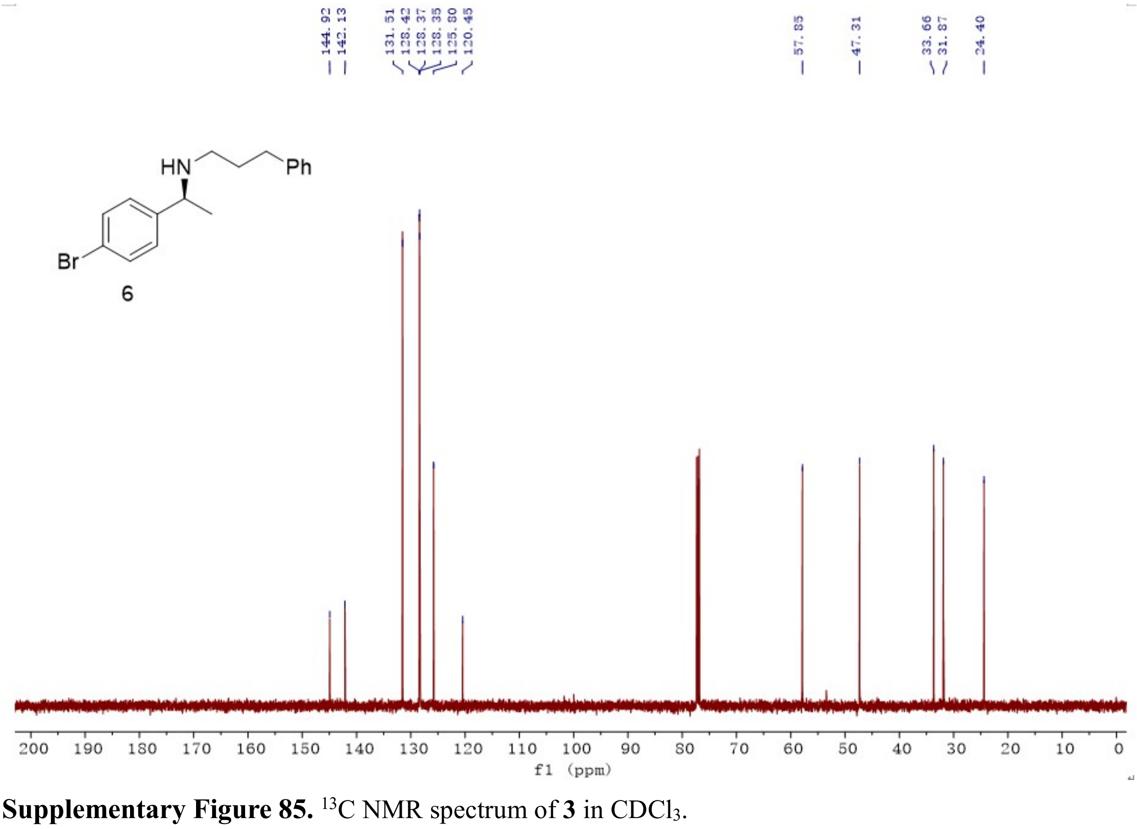

Supplementary Figure 85. ^{13}C NMR spectrum of **3** in $CDCl_3$.

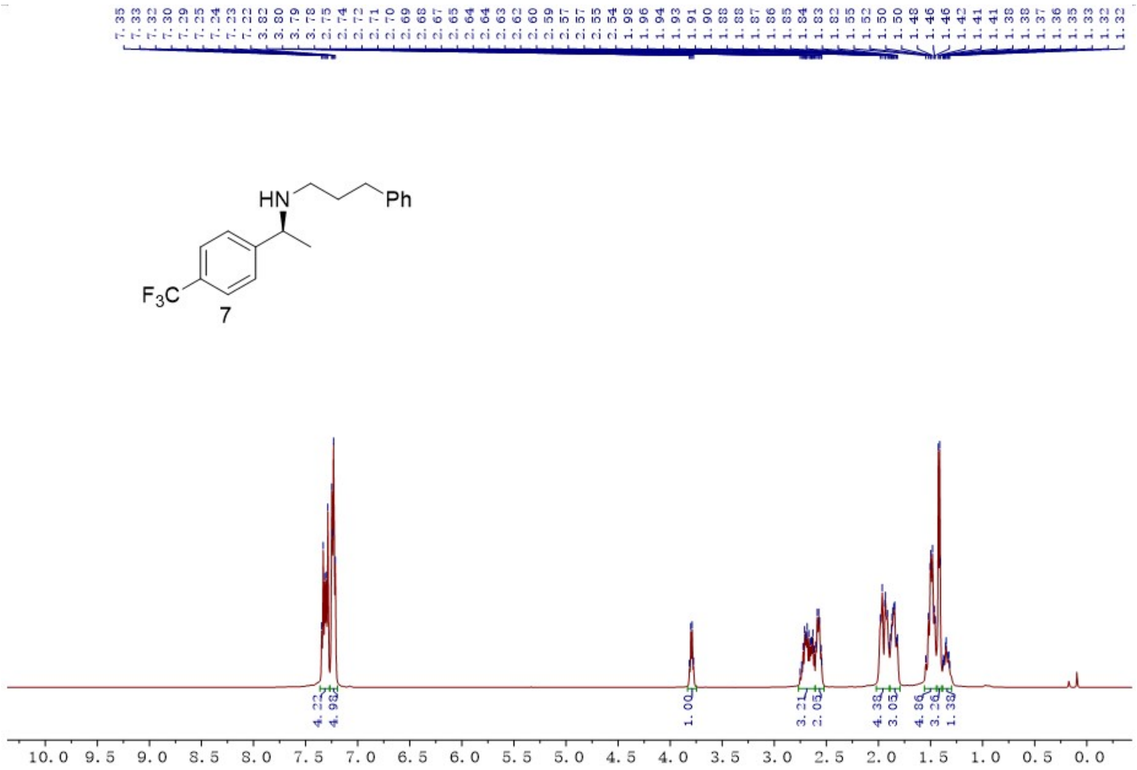

Supplementary Figure 86. ^{1}H NMR spectrum of **7** in $CDCl_3$.

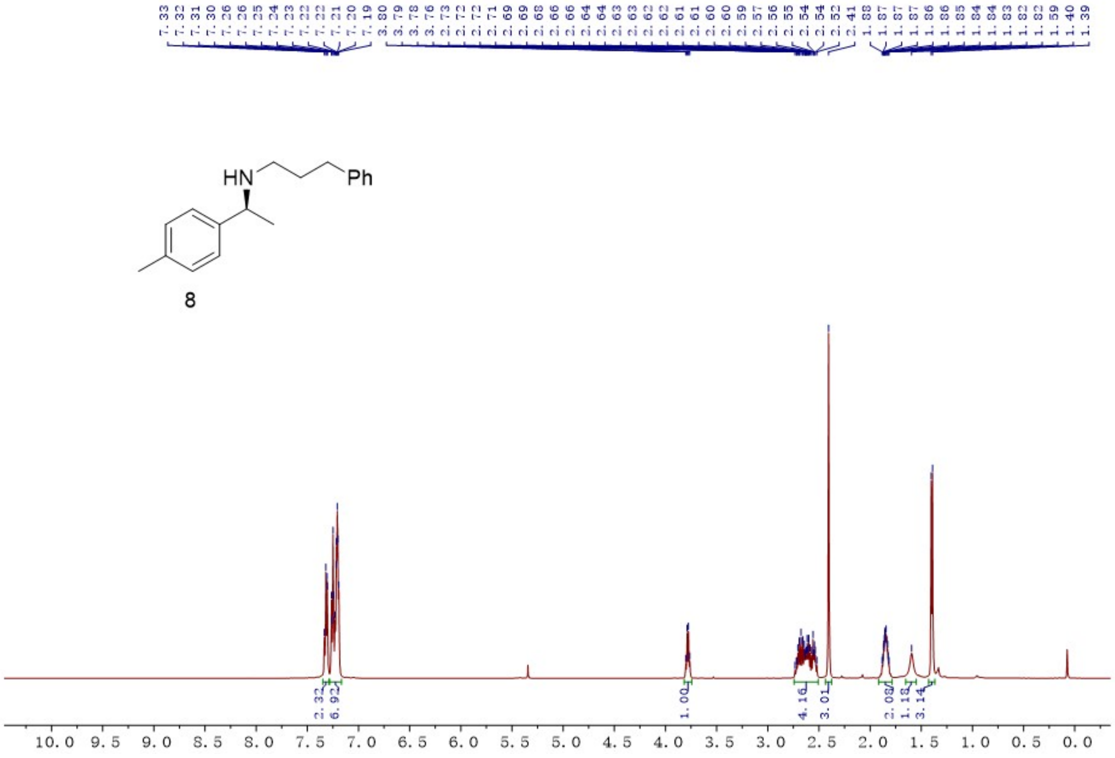

Supplementary Figure 87. ^{1}H NMR spectrum of **8** in $CDCl_3$.

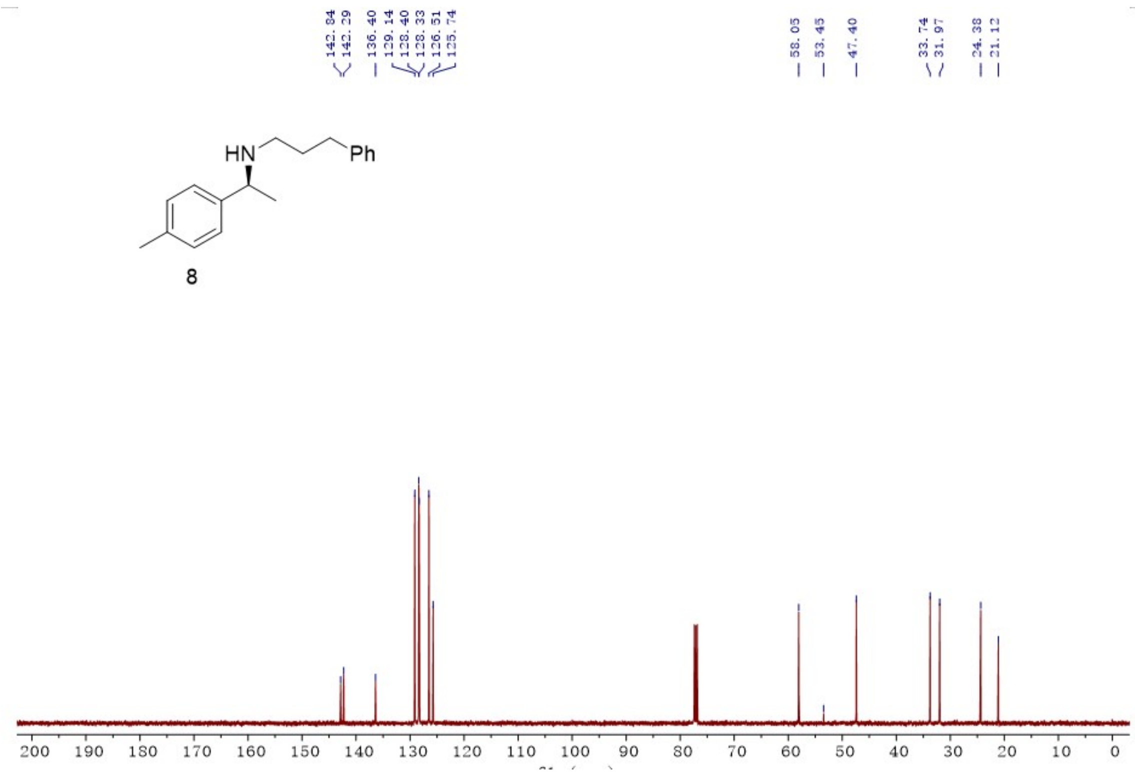

Supplementary Figure 88. ^{13}C NMR spectrum of **8** in $CDCl_3$.

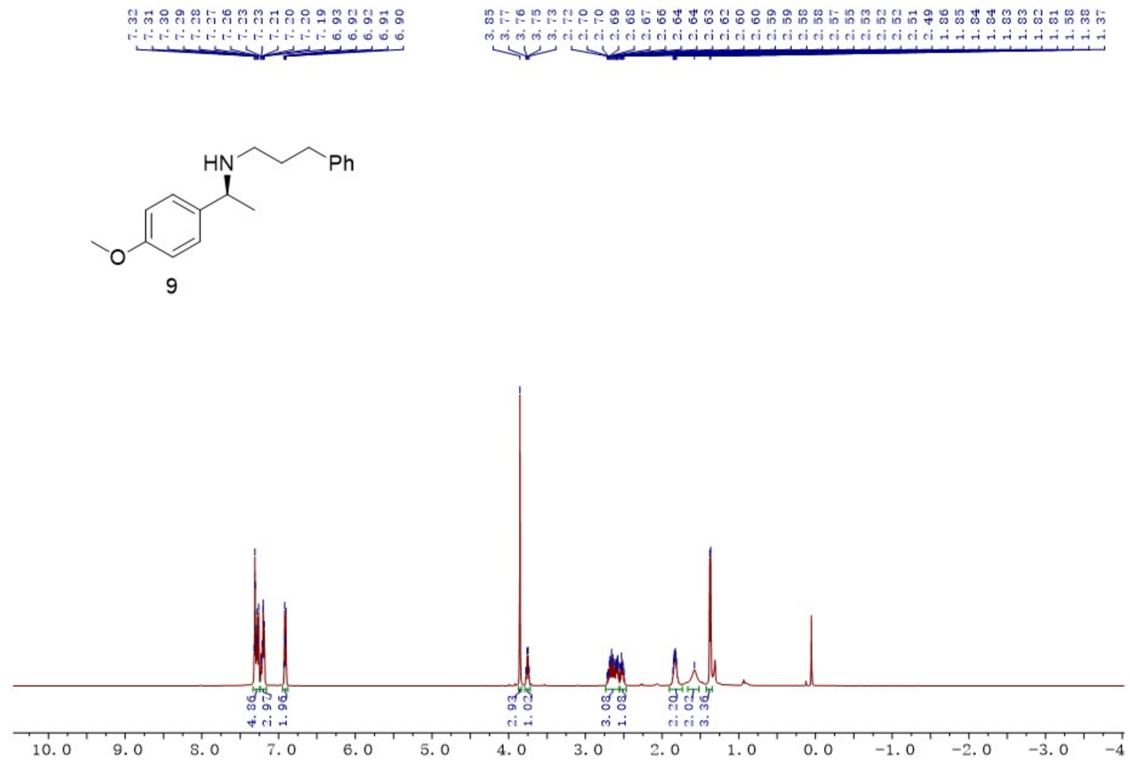

Supplementary Figure 89. ^{1}H NMR spectrum of **9** in $CDCl_3$.

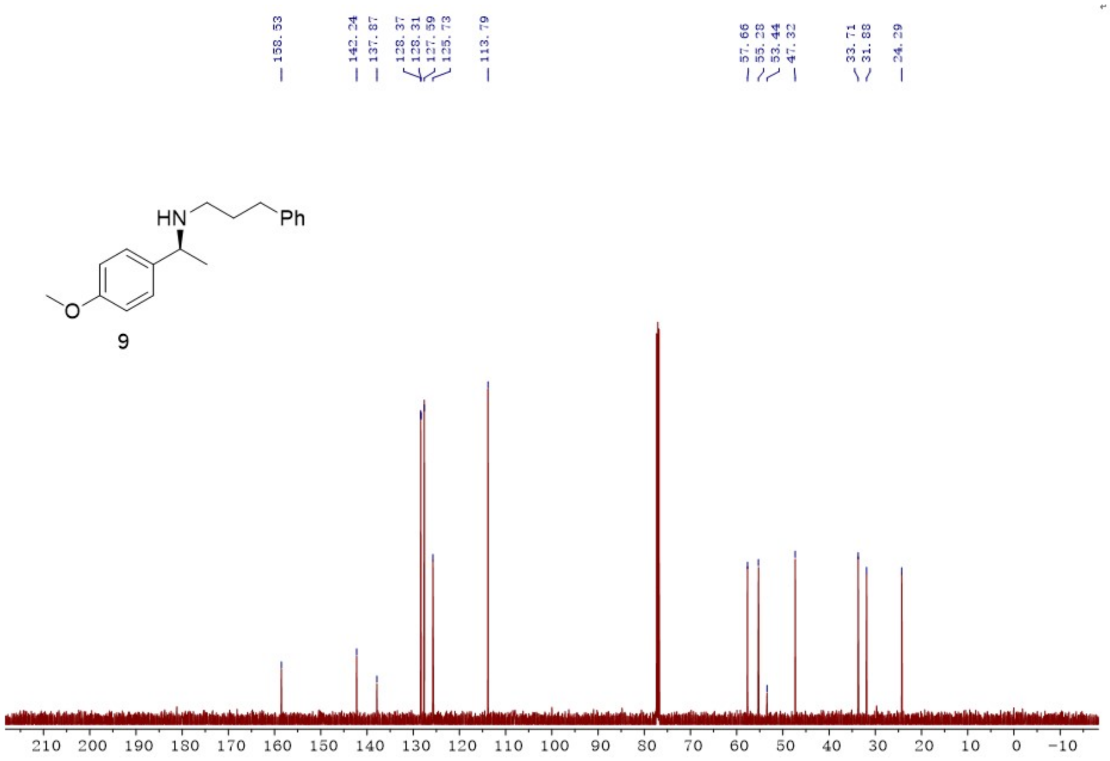

Supplementary Figure 90. ^{13}C NMR spectrum of **9** in $CDCl_3$.

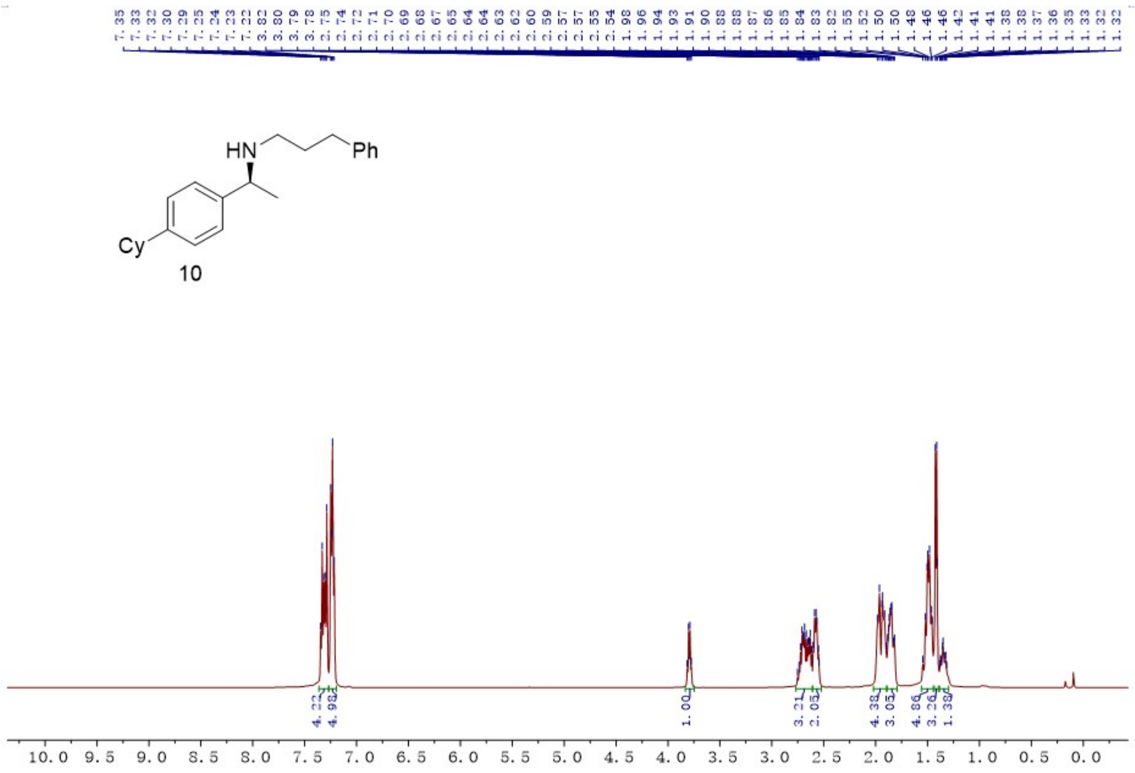

Supplementary Figure 91. ^{13}C NMR spectrum of **10** in $CDCl_3$.

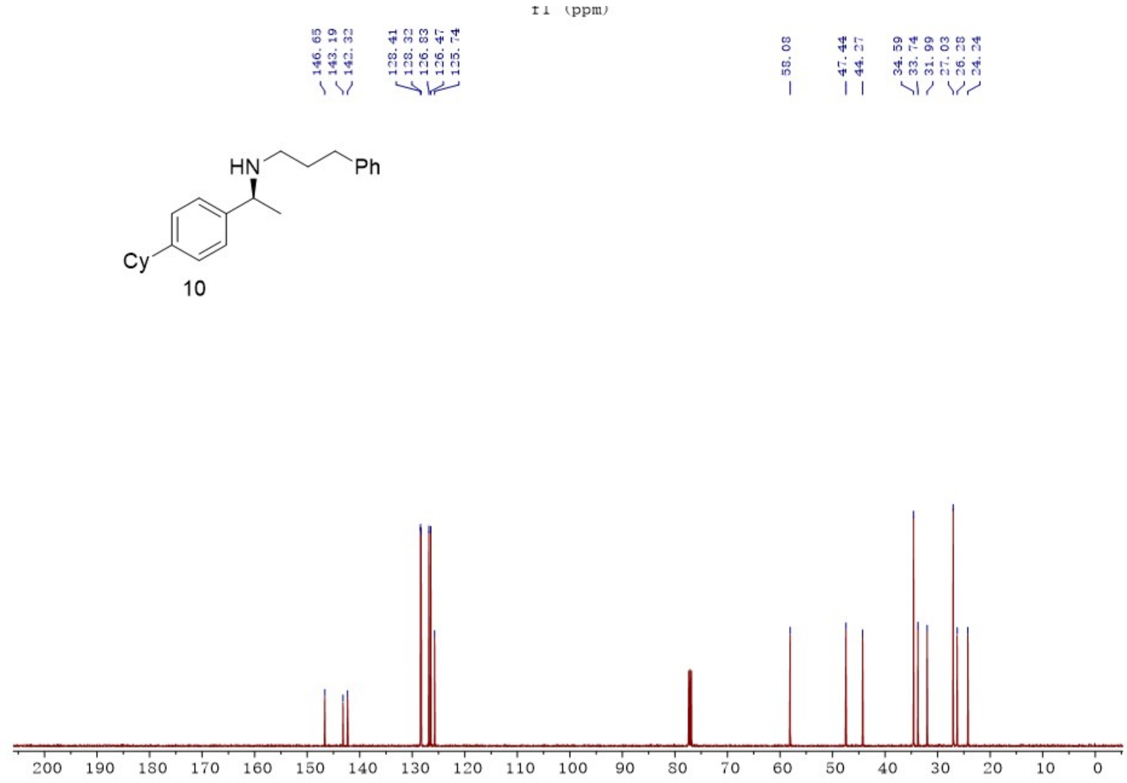

Supplementary Figure 92. ^{13}C NMR spectrum of **10** in $CDCl_3$.

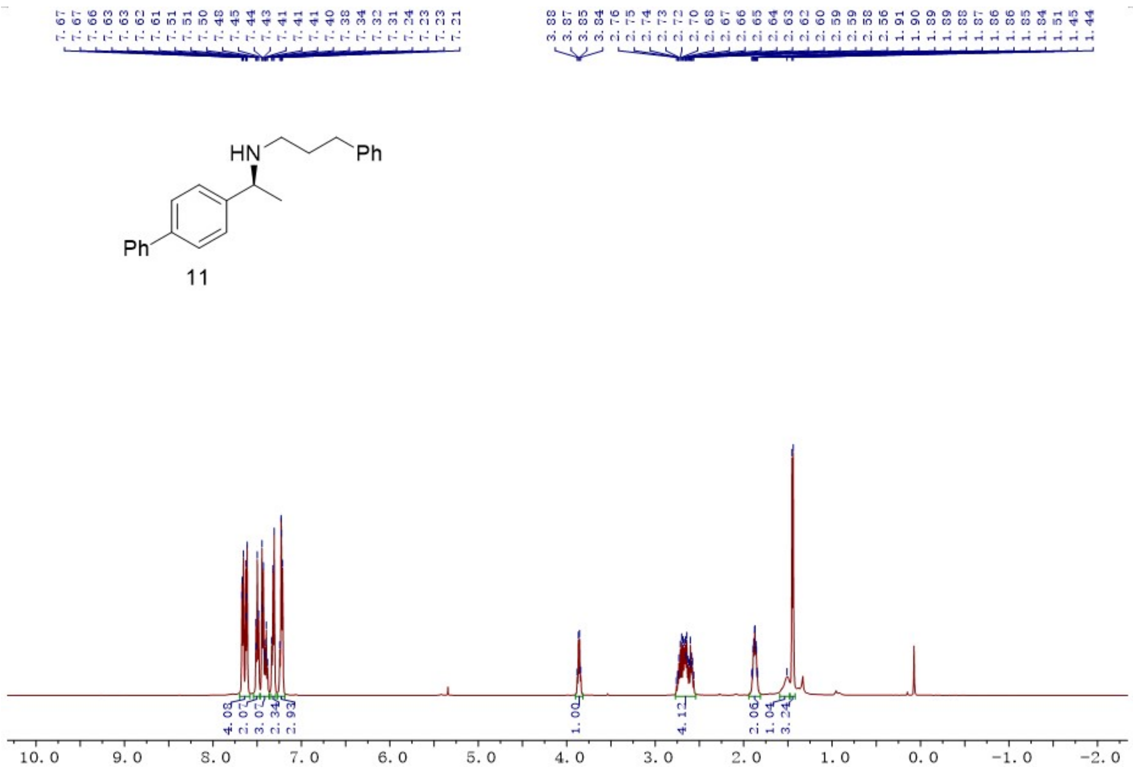

Supplementary Figure 93. ^{1}H NMR spectrum of **11** in $CDCl_3$.

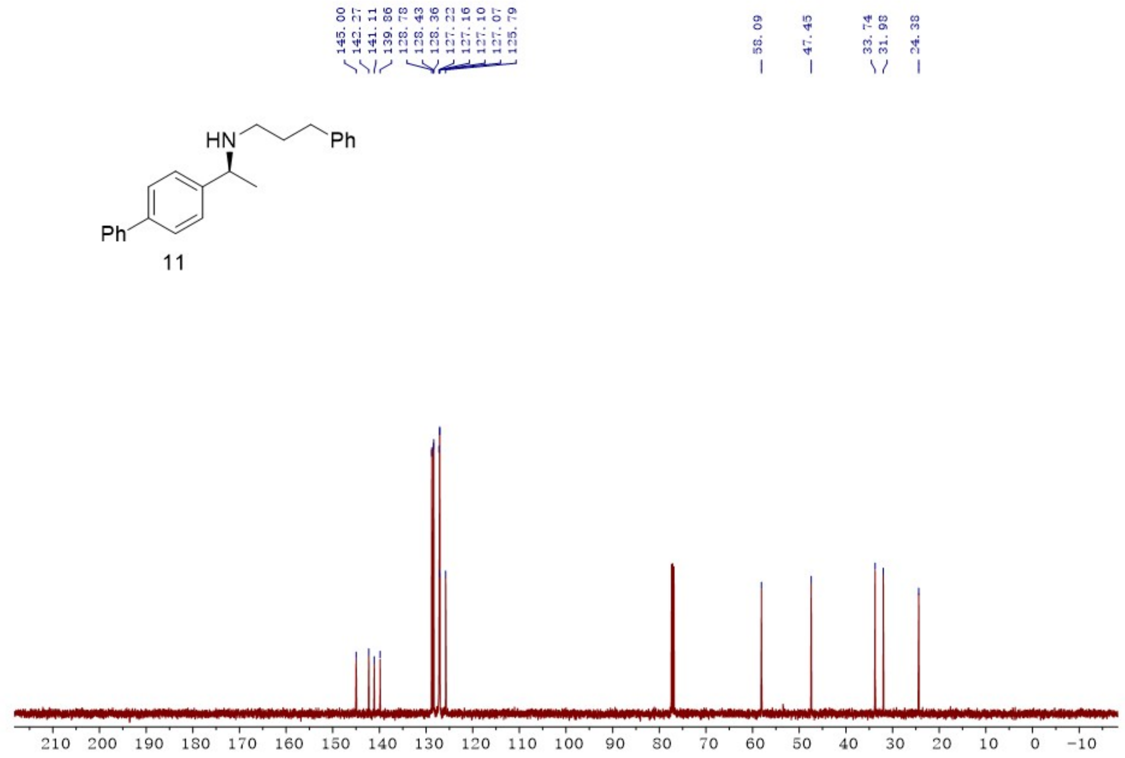

Supplementary Figure 94. ^{1}H NMR spectrum of **11** in $CDCl_3$.

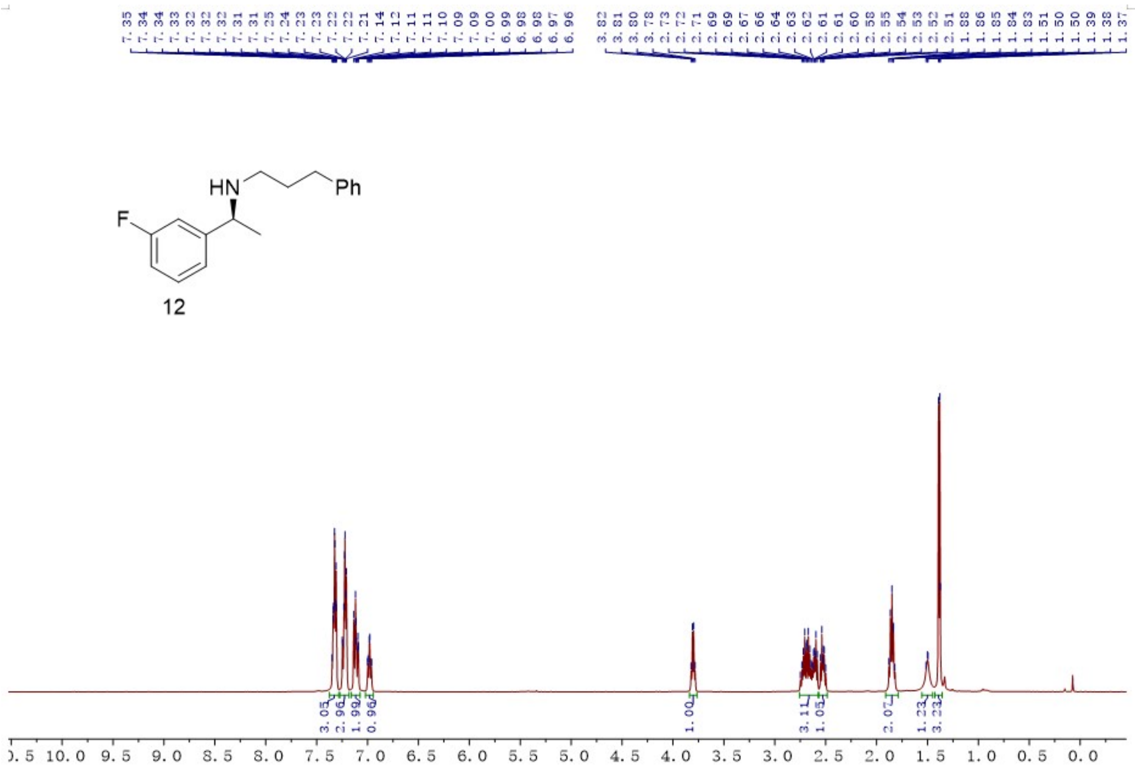

Supplementary Figure 95. ^{1}H NMR spectrum of **12** in $CDCl_3$.

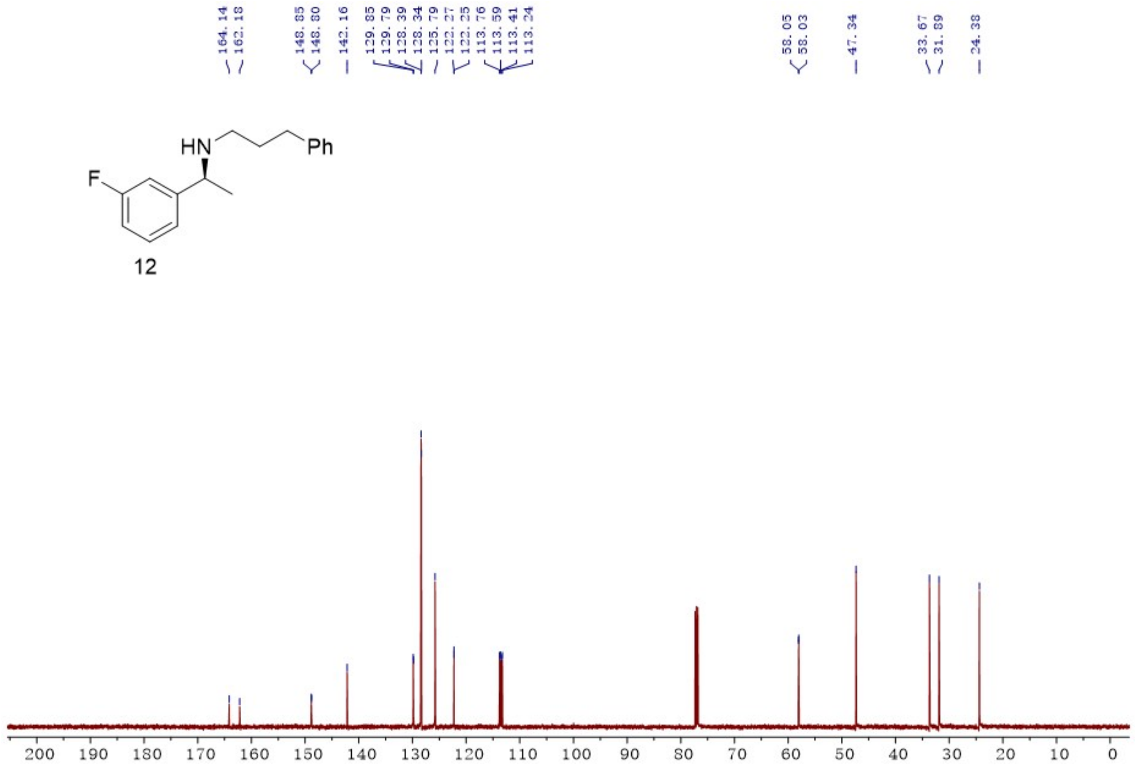

Supplementary Figure 96. ^{13}C NMR spectrum of **12** in $CDCl_3$.

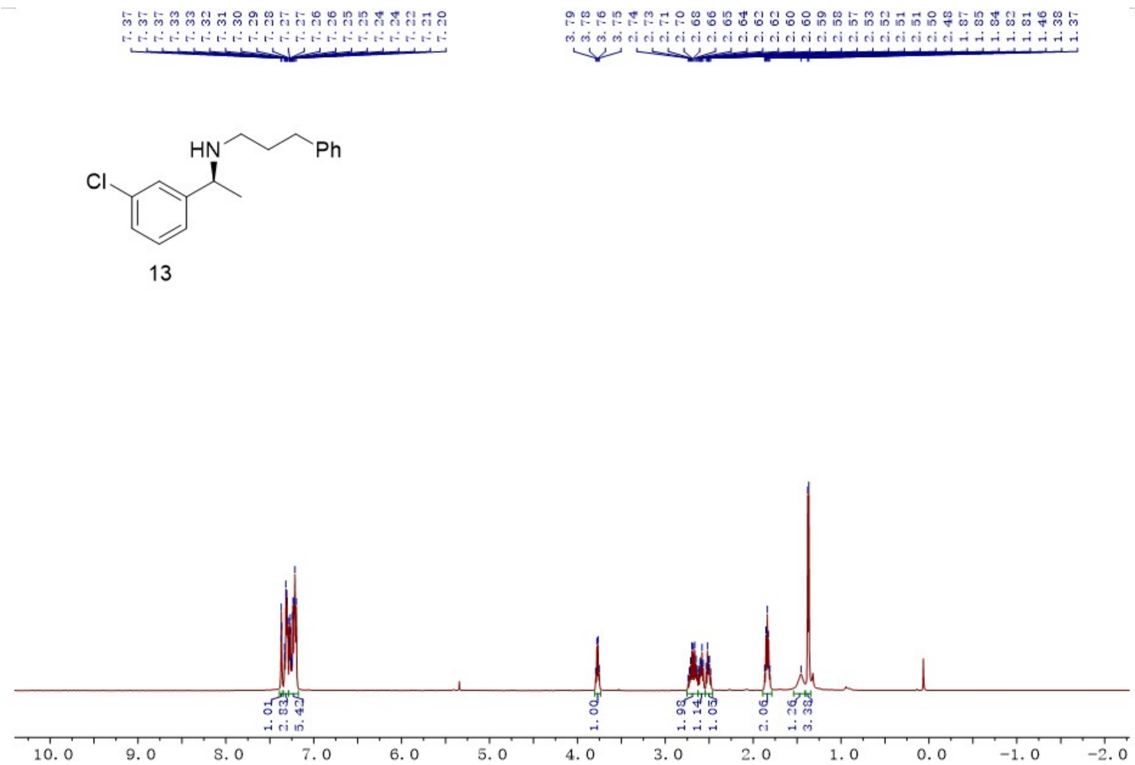

Supplementary Figure 97. ^{1}H NMR spectrum of **13** in $CDCl_3$.

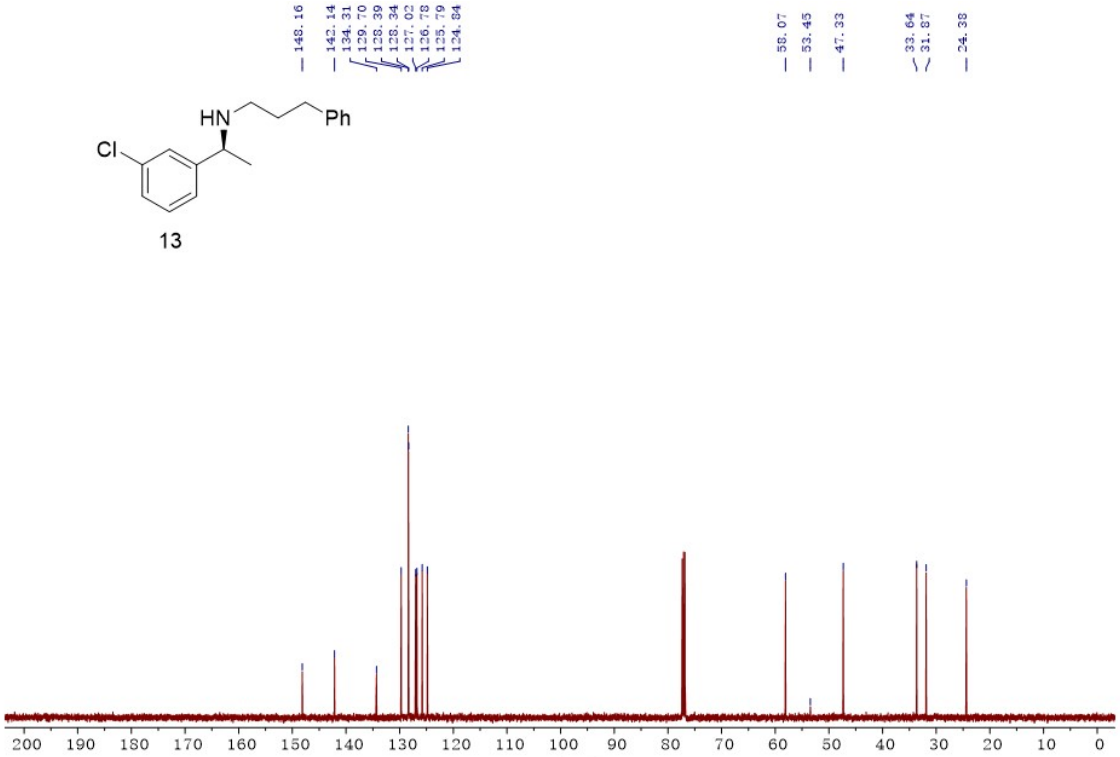

Supplementary Figure 98. ^{13}C NMR spectrum of **13** in $CDCl_3$.

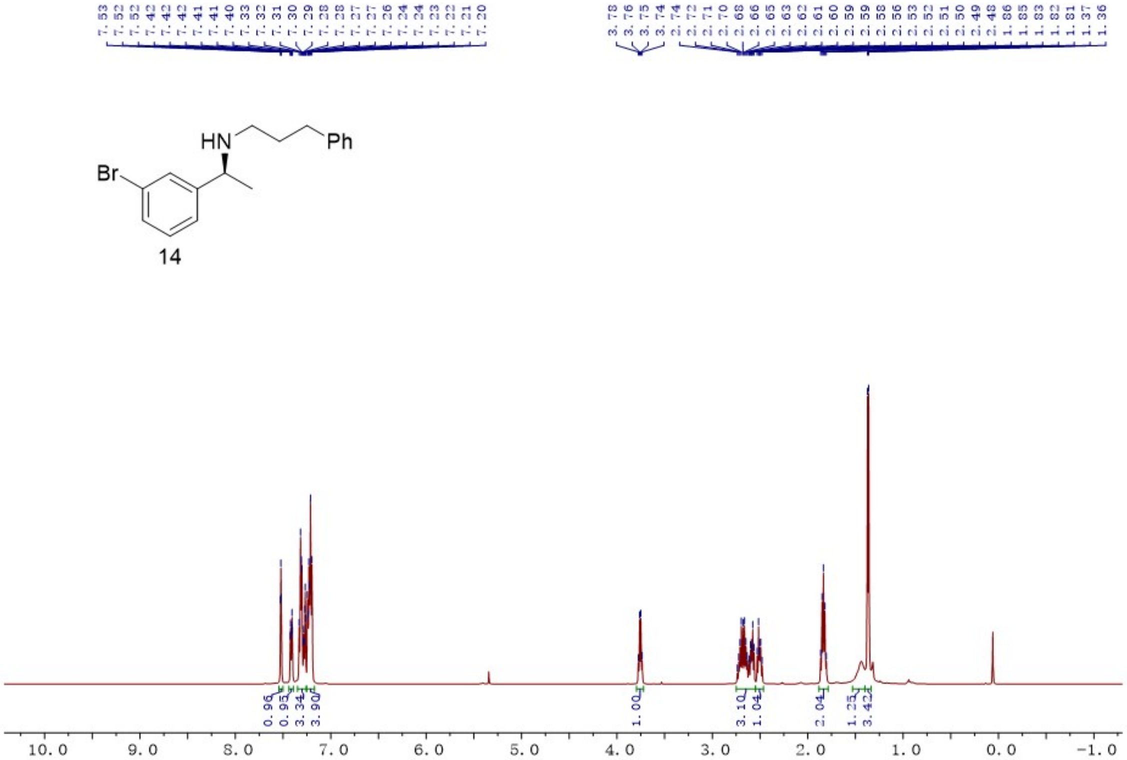

Supplementary Figure 99. ^{1}H NMR spectrum of **14** in $CDCl_3$.

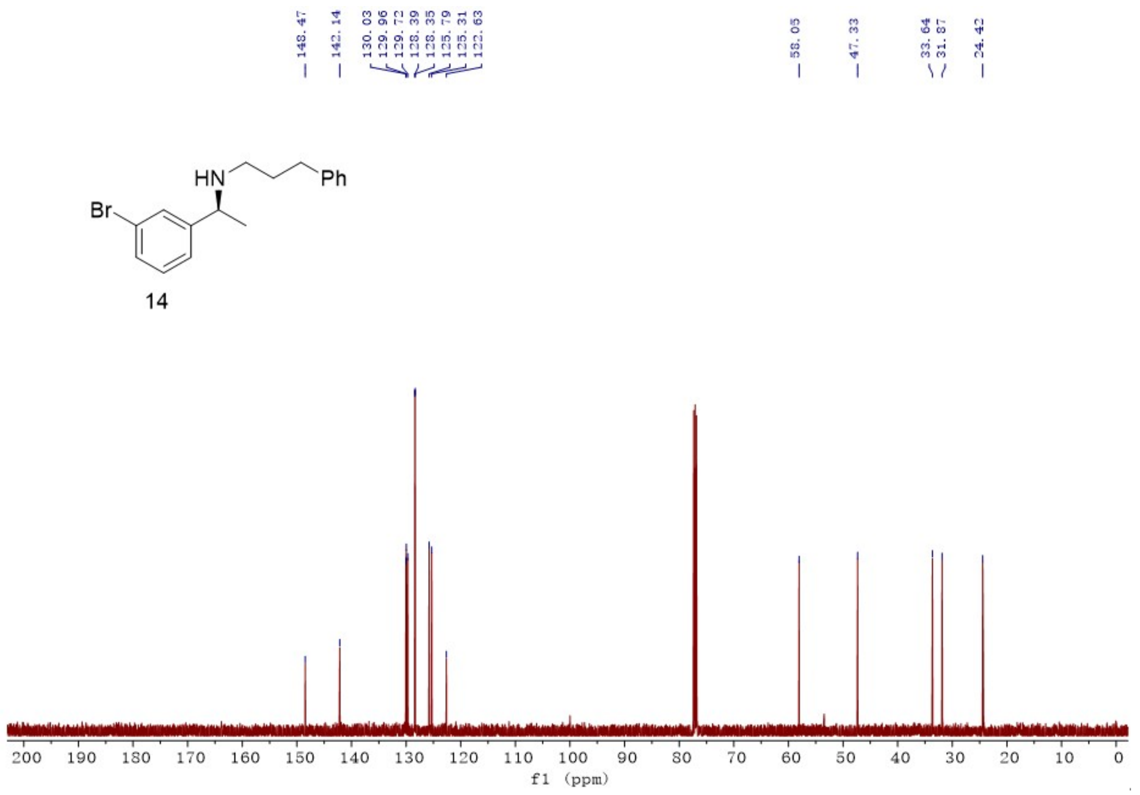

Supplementary Figure 100. ^{13}C NMR spectrum of **14** in $CDCl_3$.

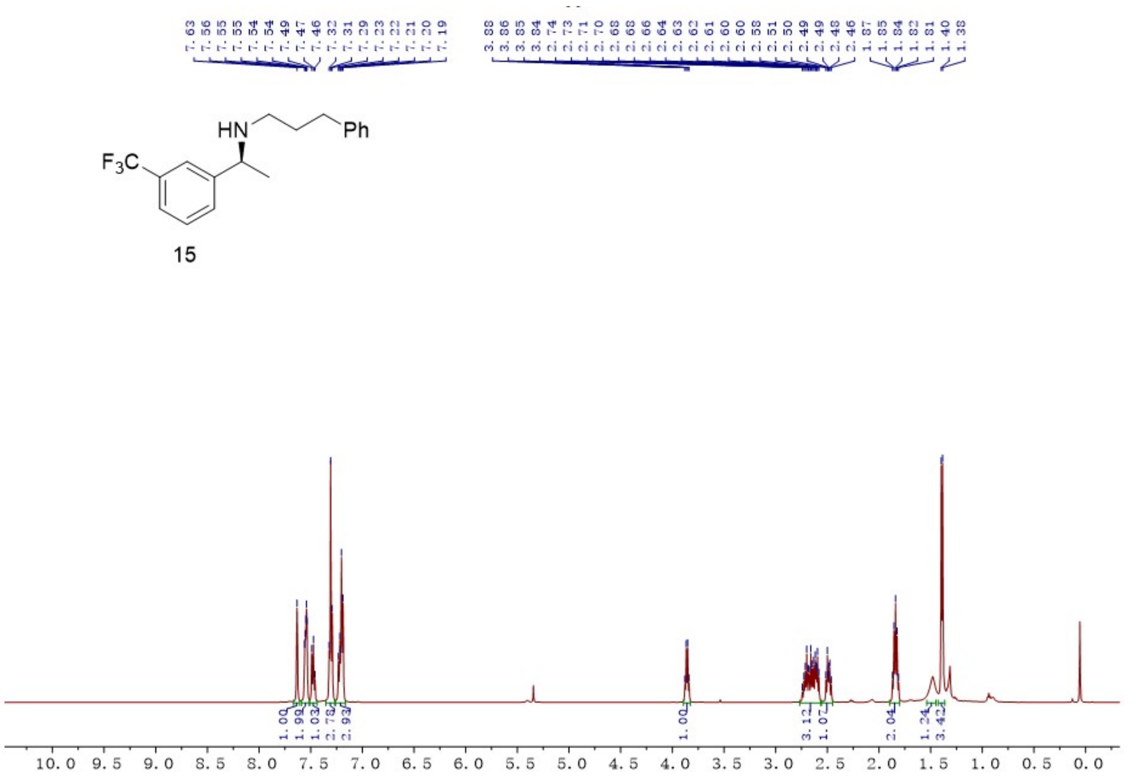

Supplementary Figure 101. ^{1}H NMR spectrum of **15** in $CDCl_3$.

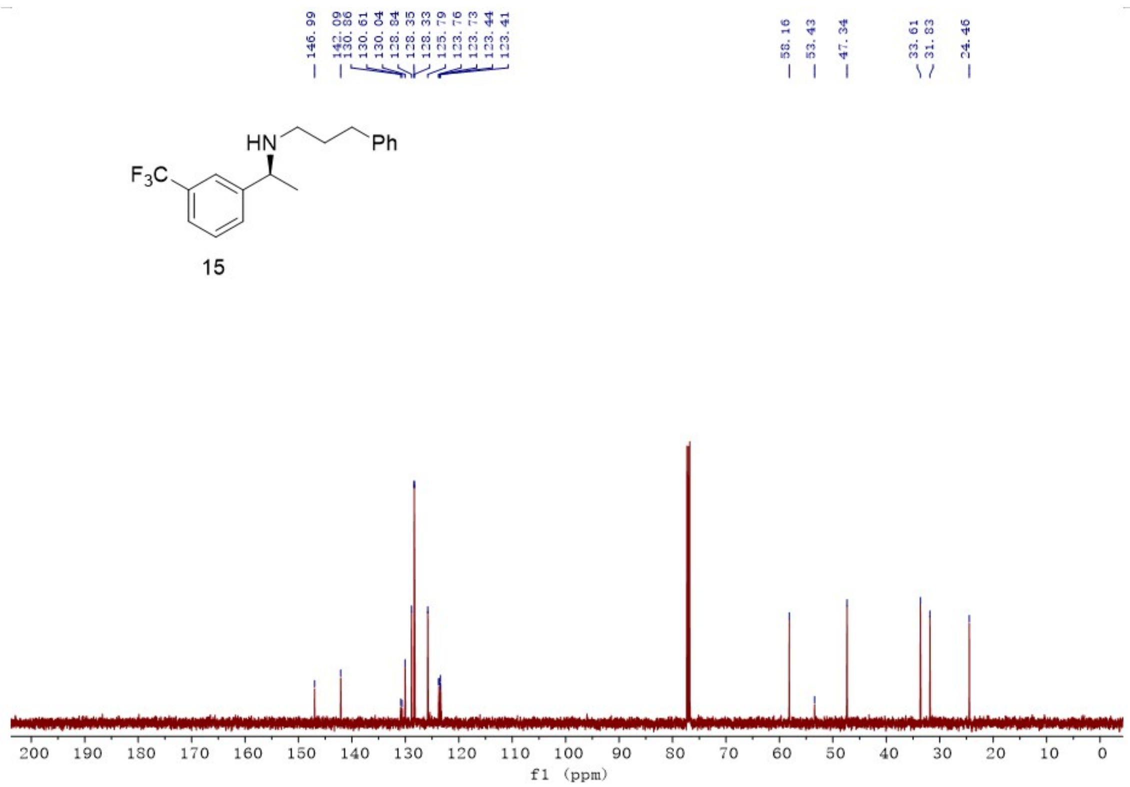

Supplementary Figure 102. ^{13}C NMR spectrum of **15** in $CDCl_3$.

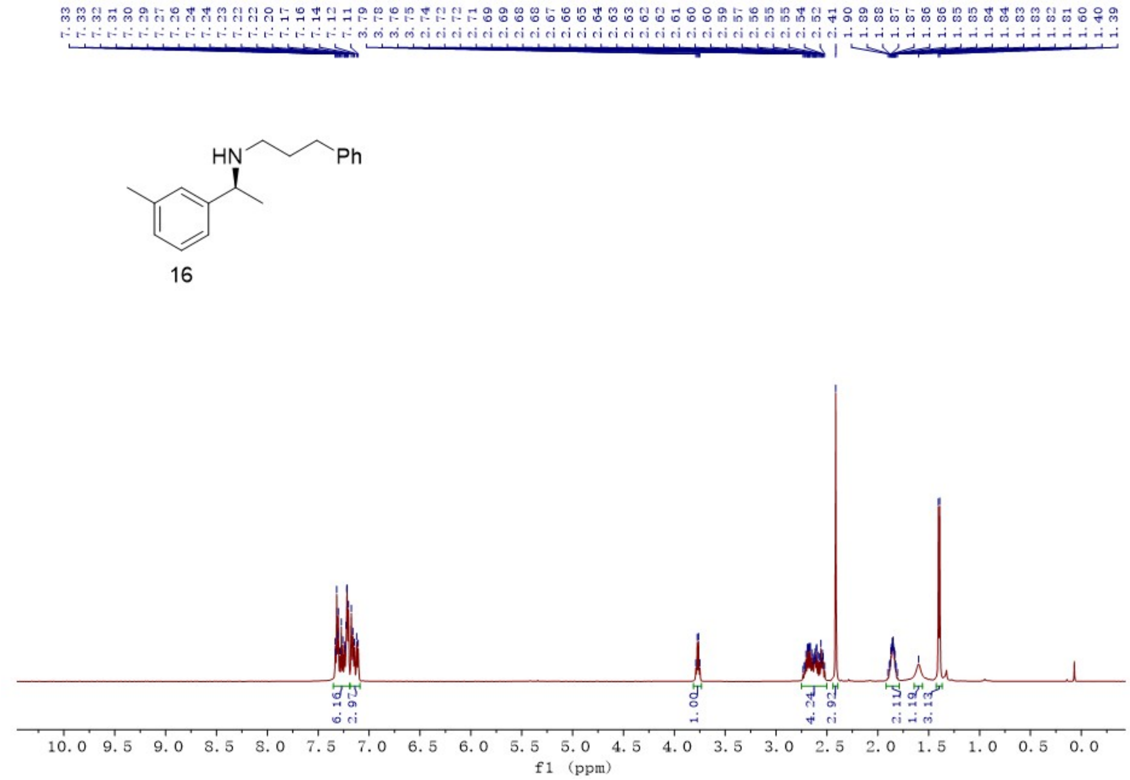

Supplementary Figure 103. ^{1}H NMR spectrum of **16** in $CDCl_3$.

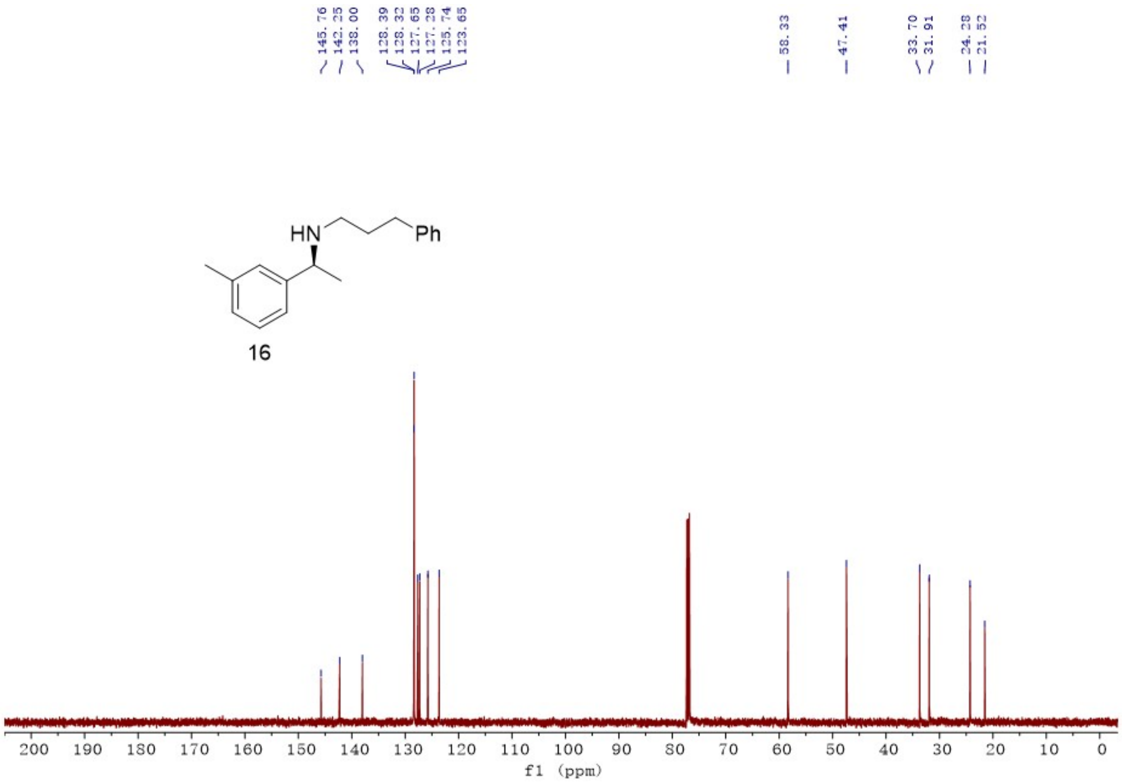

Supplementary Figure 104. ^{13}C NMR spectrum of **16** in $CDCl_3$.

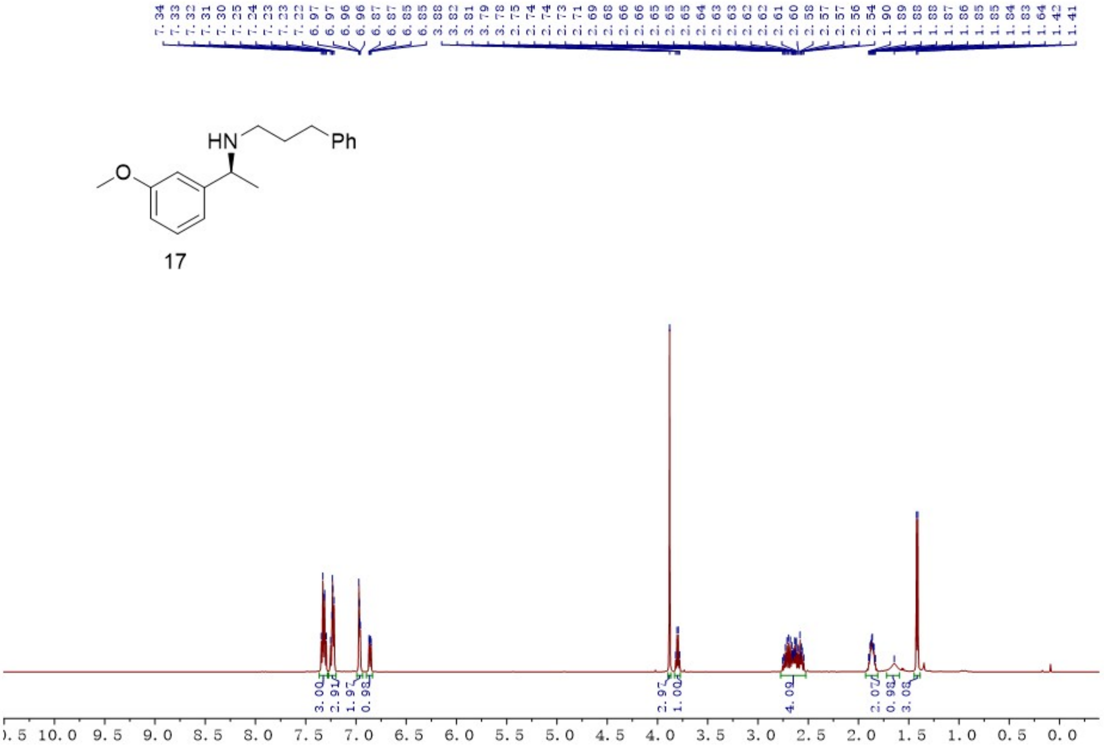

Supplementary Figure 105. ^{1}H NMR spectrum of **17** in $CDCl_3$.

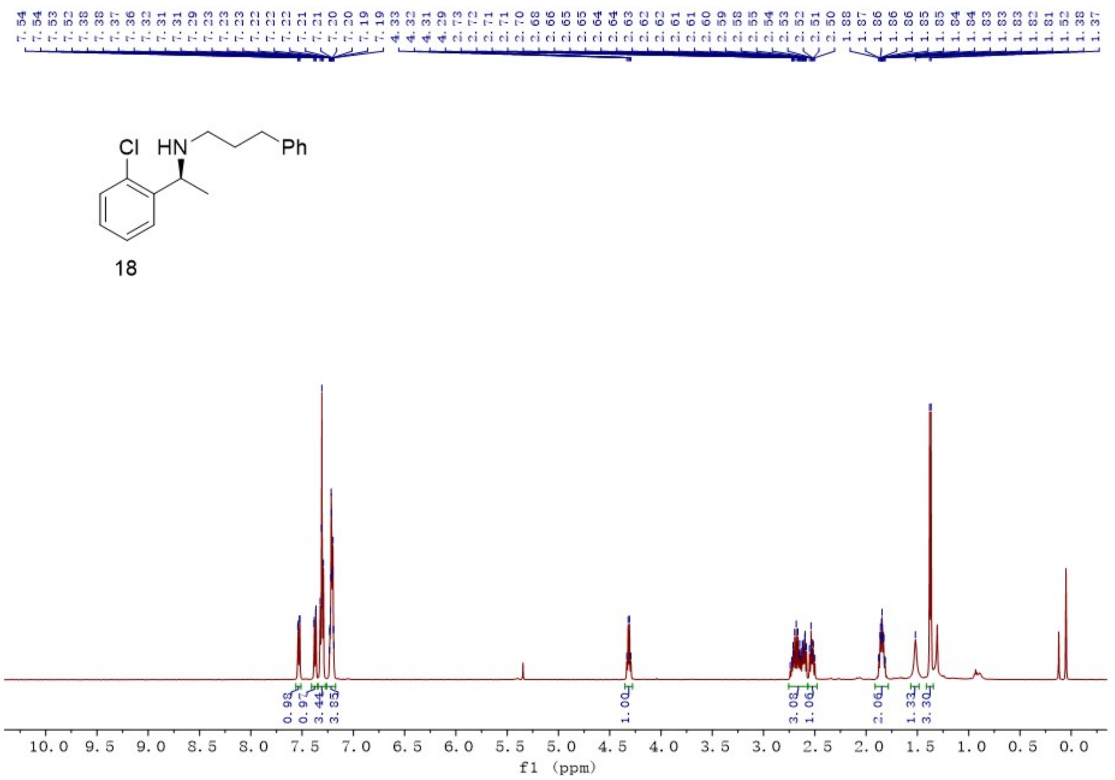

Supplementary Figure 106. ^{1}H NMR spectrum of **18** in $CDCl_3$.

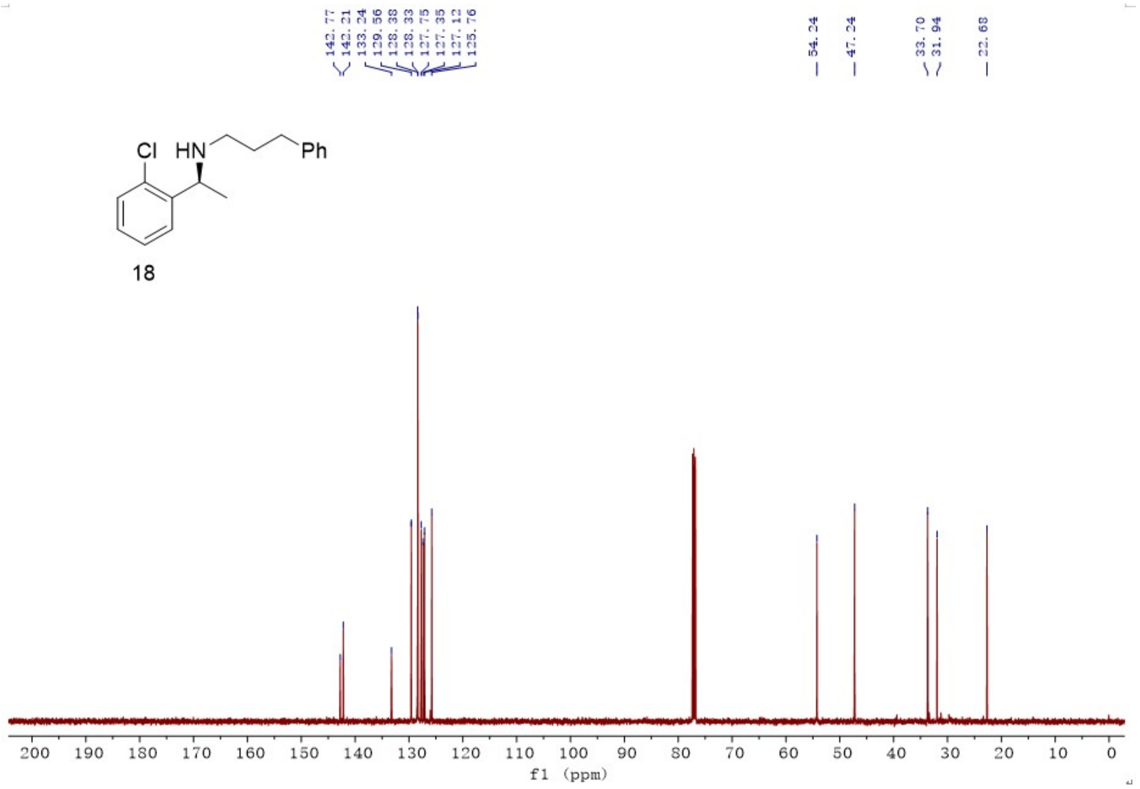

Supplementary Figure 107. ^{13}C NMR spectrum of **18** in $CDCl_3$.

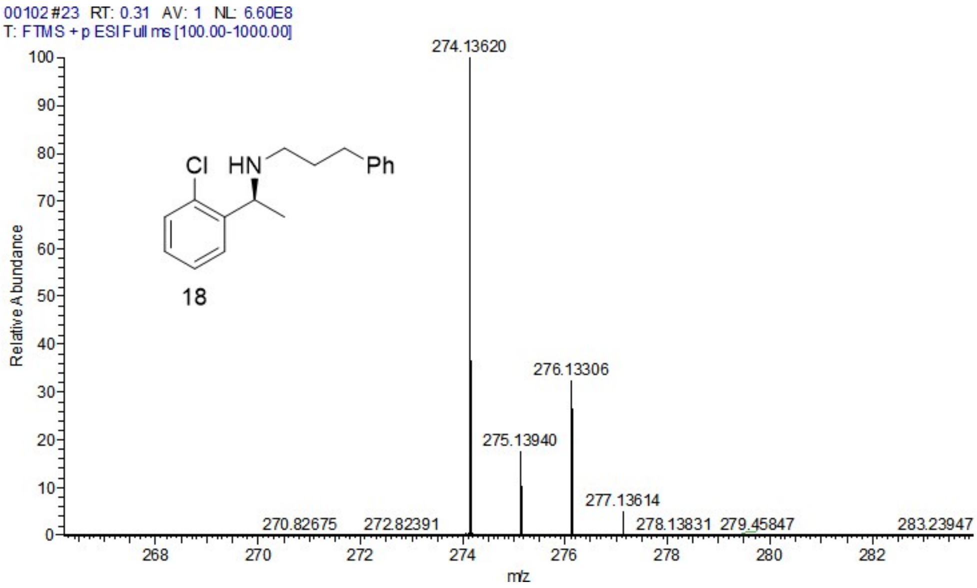

Supplementary Figure 108. HRMS of **18**.

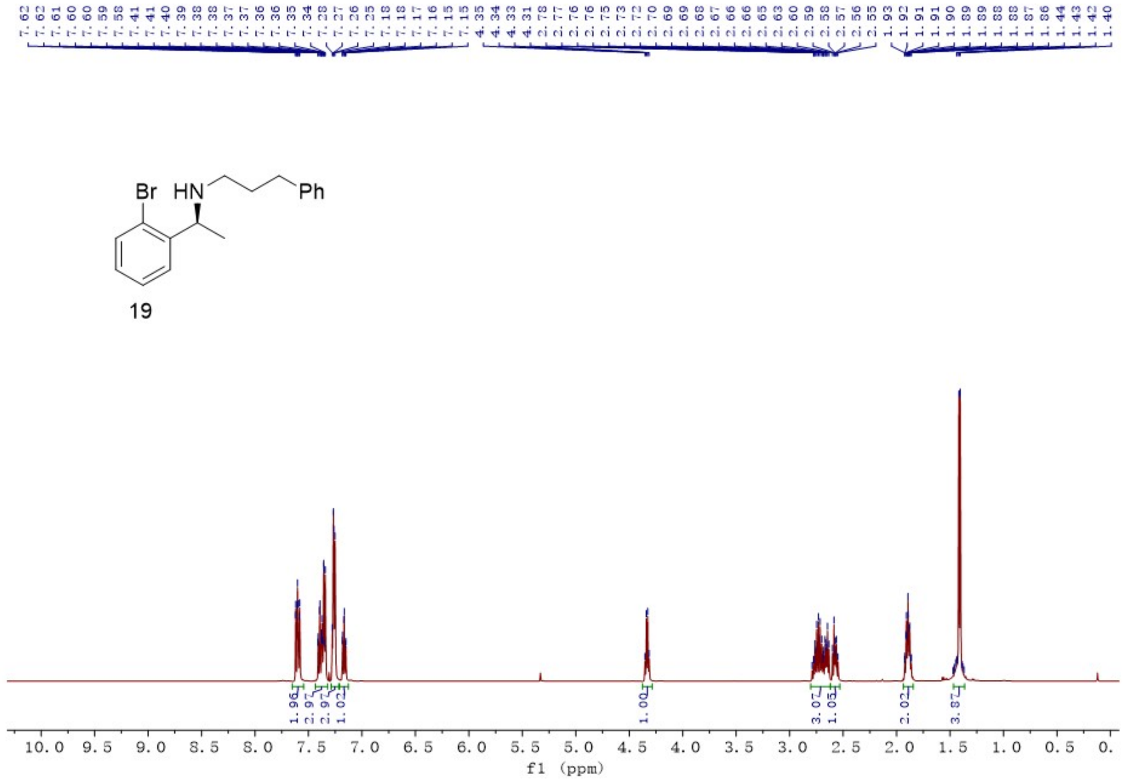

Supplementary Figure 109. ^{1}H NMR spectrum of **19** in $CDCl_3$.

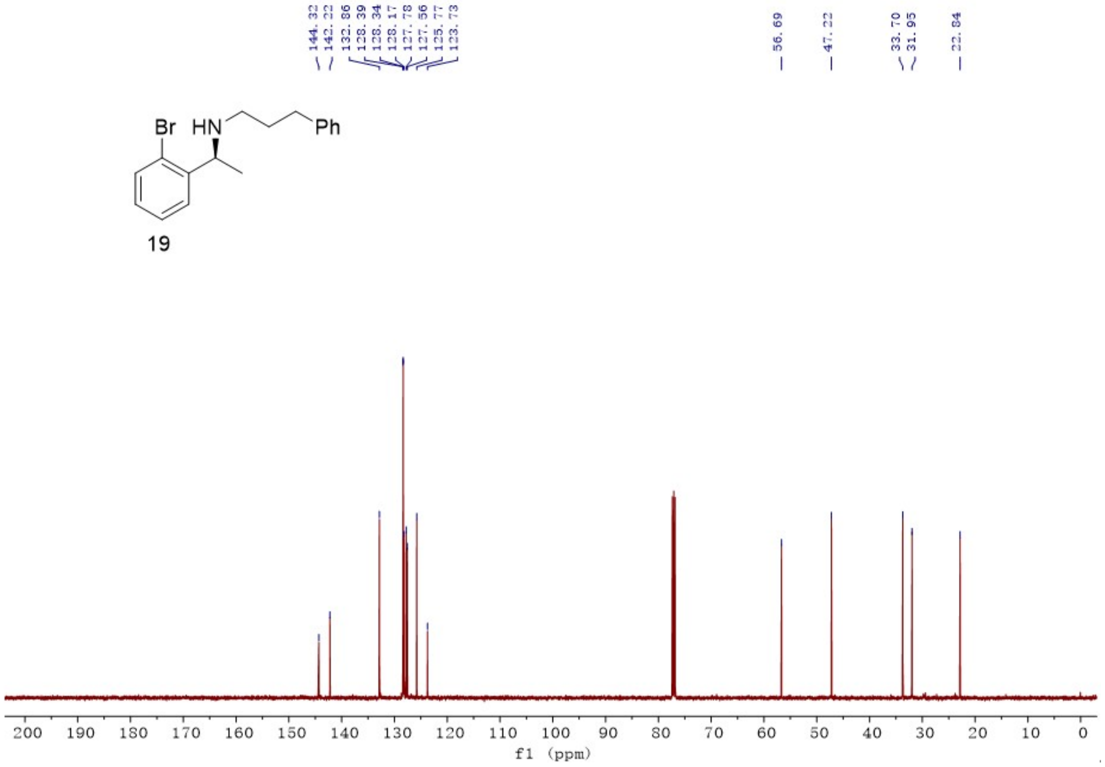

Supplementary Figure 110. ^{13}C NMR spectrum of **19** in $CDCl_3$.

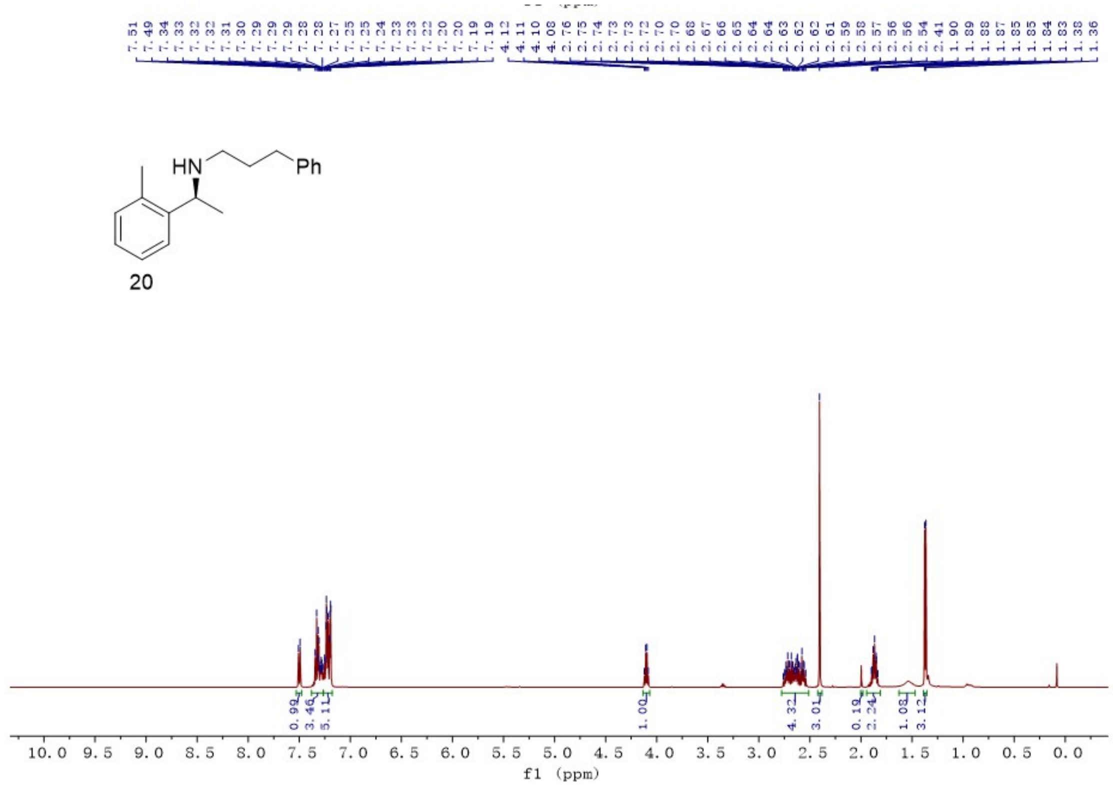

Supplementary Figure 111. ^{1}H NMR spectrum of **20** in $CDCl_3$.

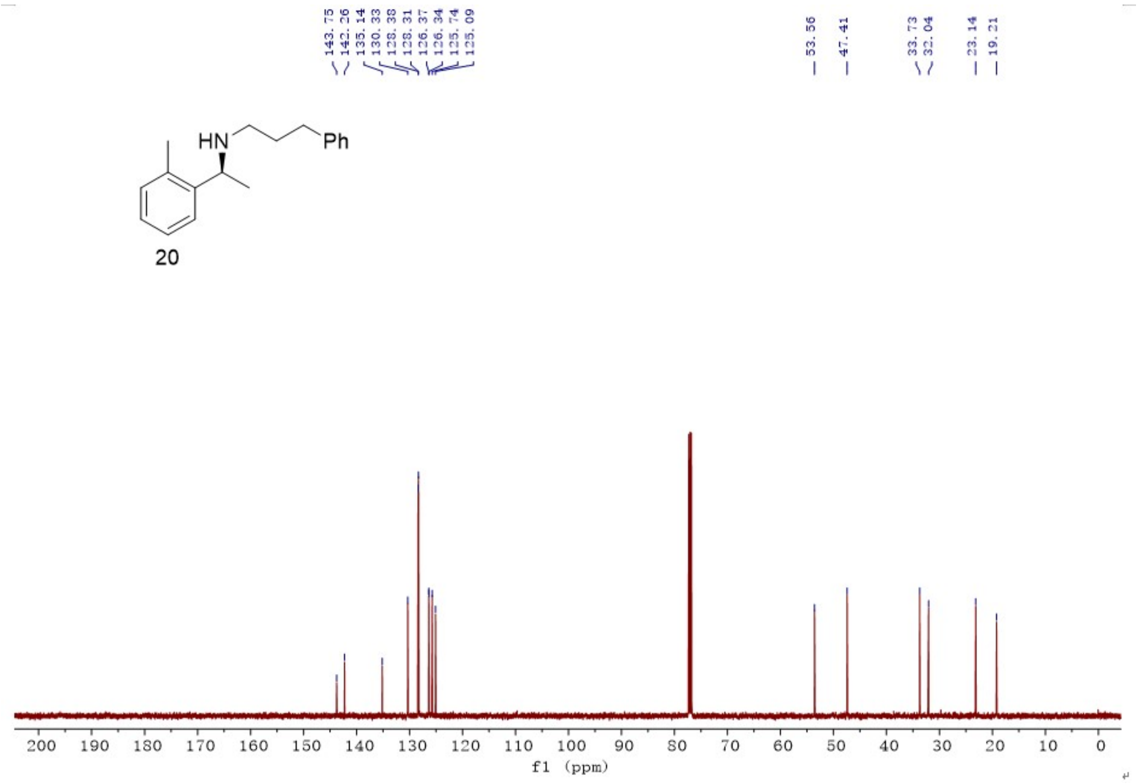

Supplementary Figure 112. ^{13}C NMR spectrum of **20** in $CDCl_3$.

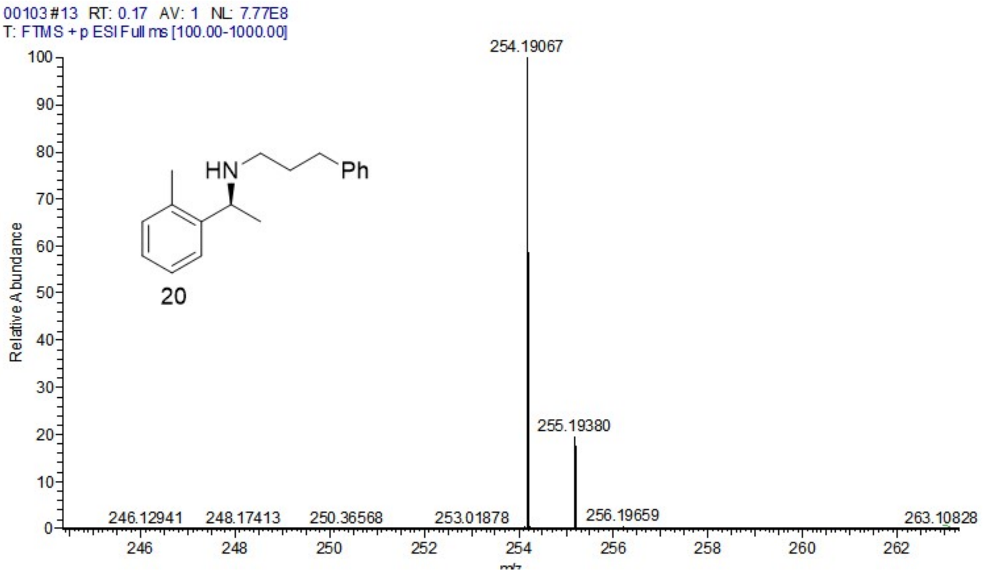

Supplementary Figure 113. HRMS of **20**.

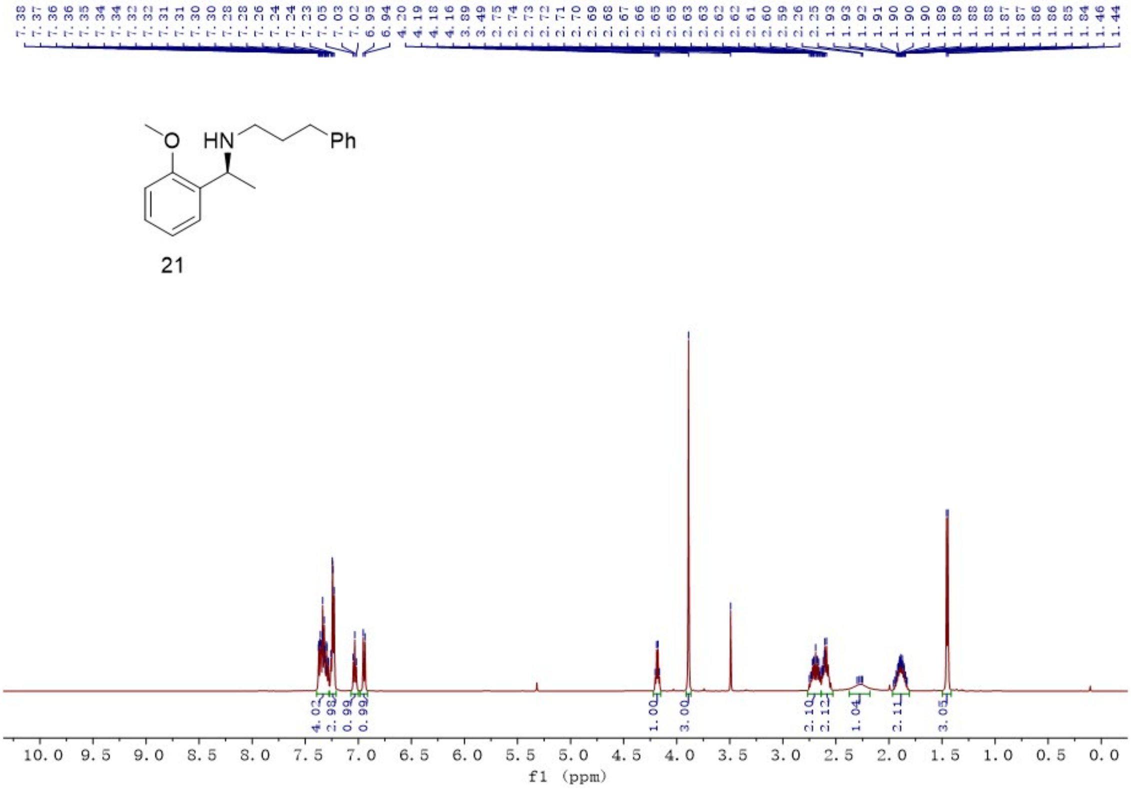

Supplementary Figure 114. ^{1}H NMR spectrum of **21** in $CDCl_3$.

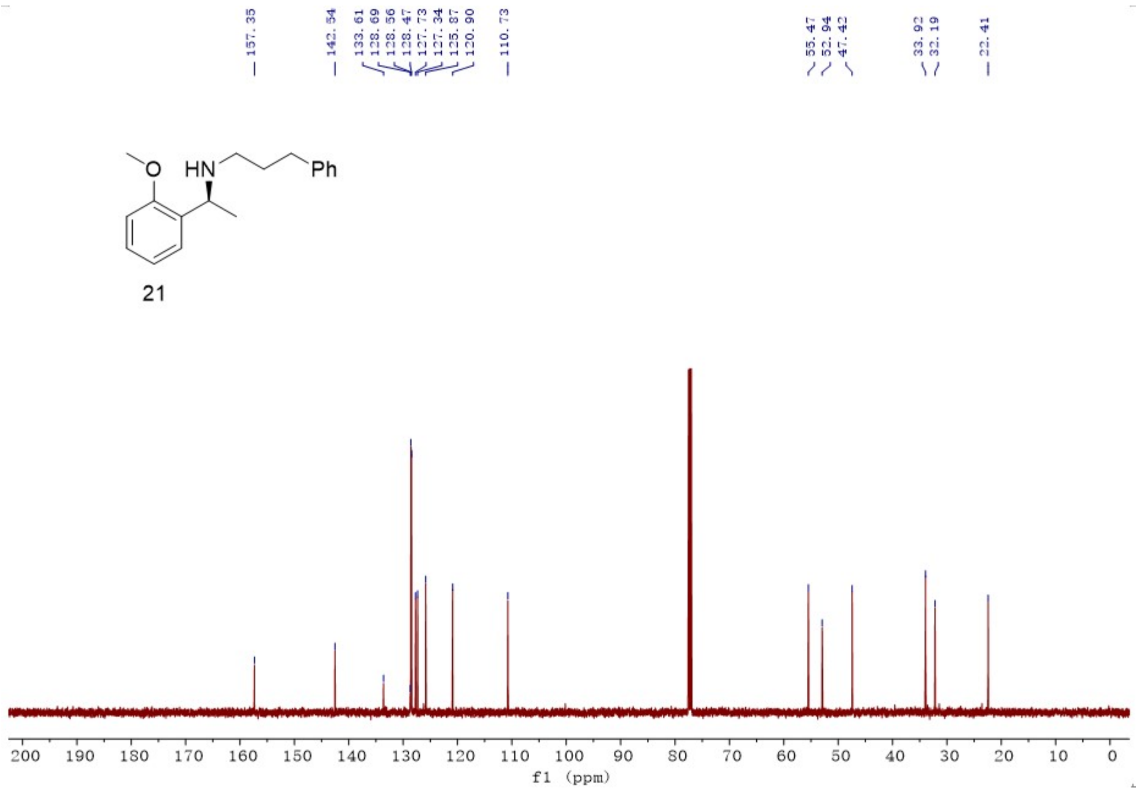

Supplementary Figure 115. ^{13}C NMR spectrum of **21** in $CDCl_3$.

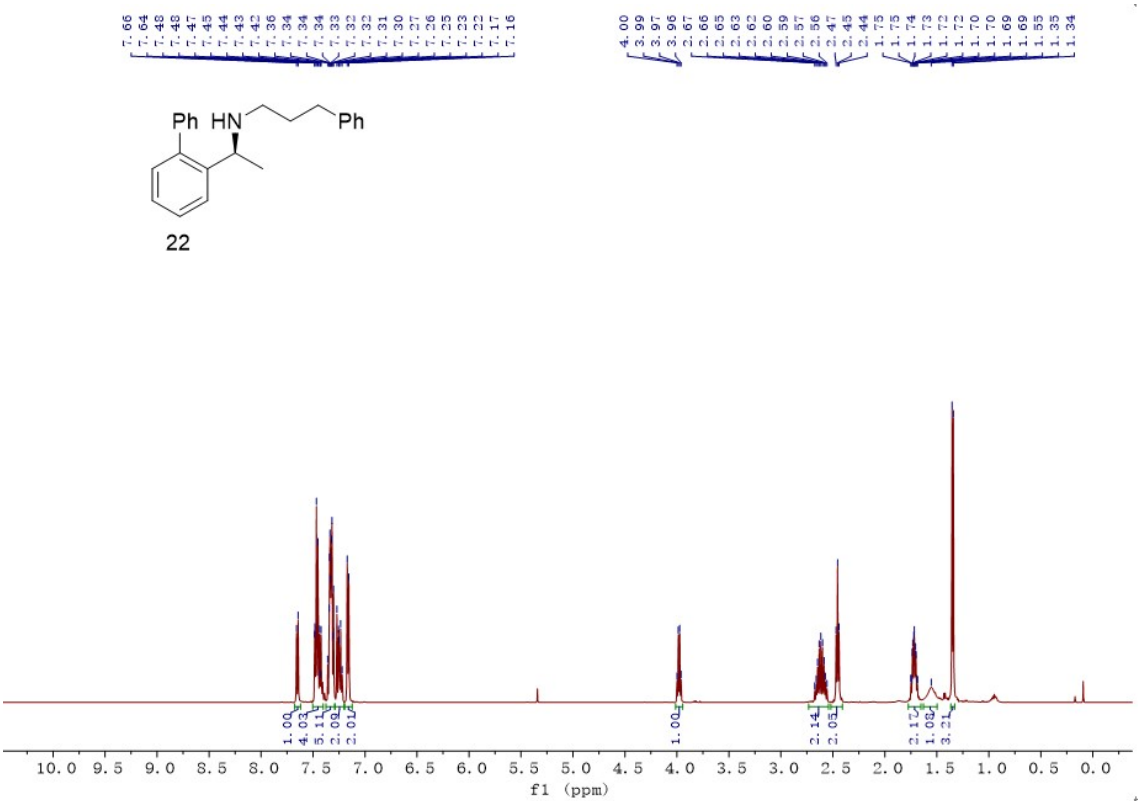

Supplementary Figure 116. ^{1}H NMR spectrum of **22** in $CDCl_3$.

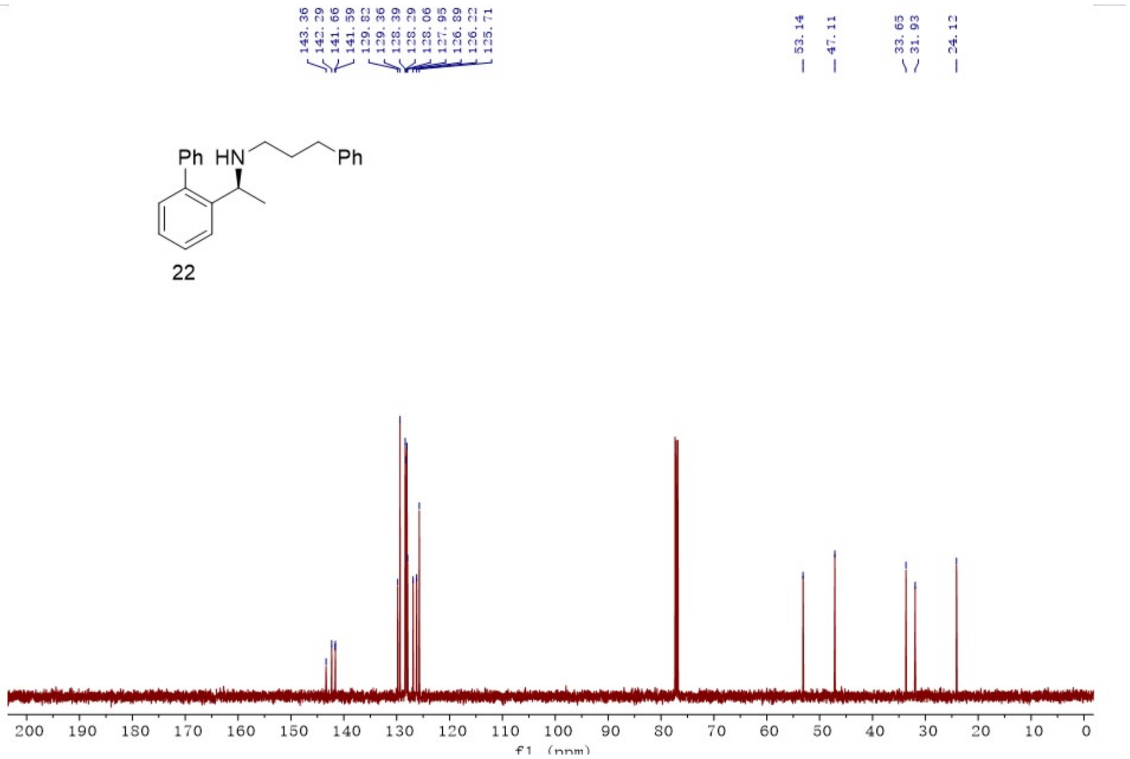

Supplementary Figure 117. ^{13}C NMR spectrum of **22** in $CDCl_3$.

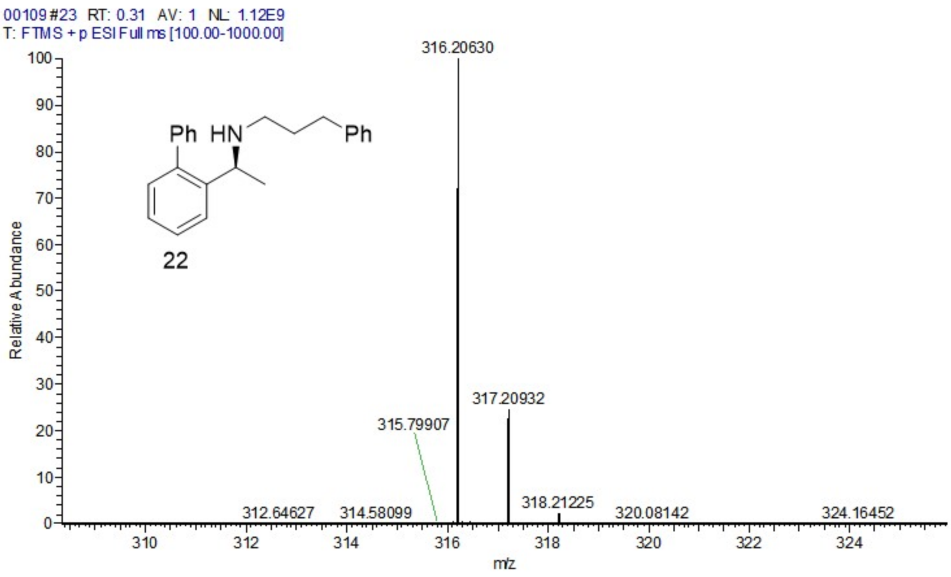

Supplementary Figure 118. ^{13}C NMR spectrum of **22** in $CDCl_3$.

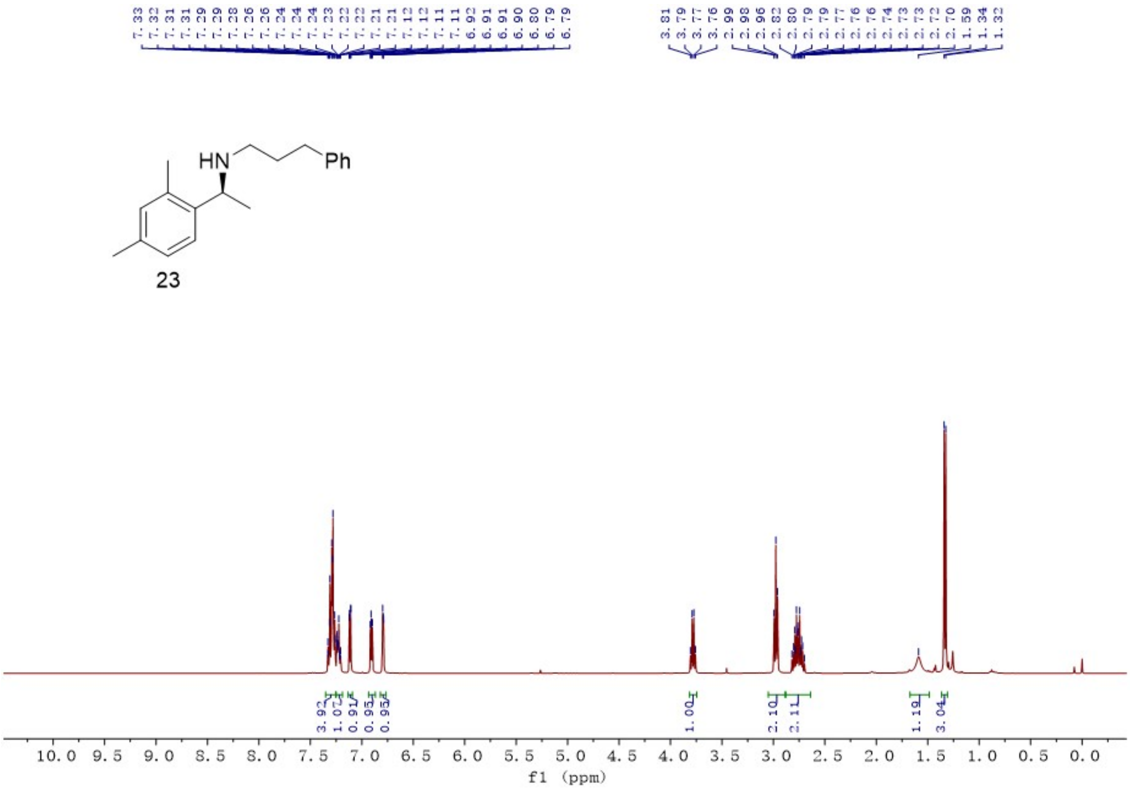

Supplementary Figure 119. ^{1}H NMR spectrum of **23** in $CDCl_3$.

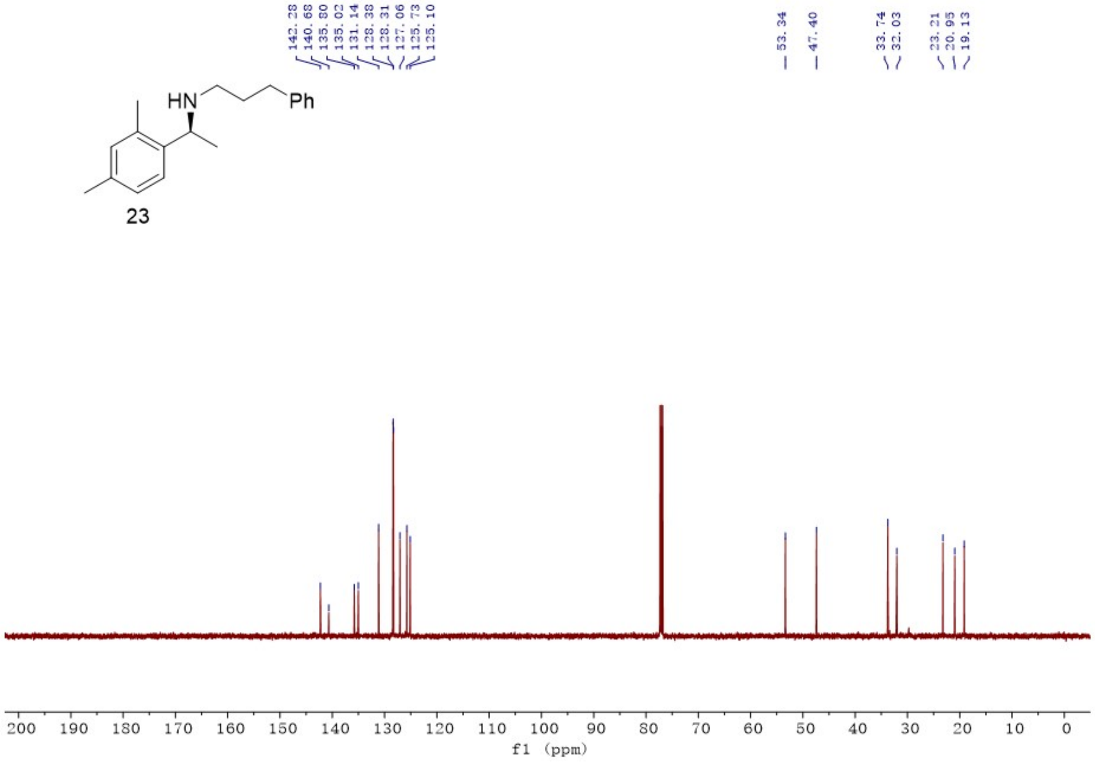

Supplementary Figure 120. ^{13}C NMR spectrum of **23** in $CDCl_3$.

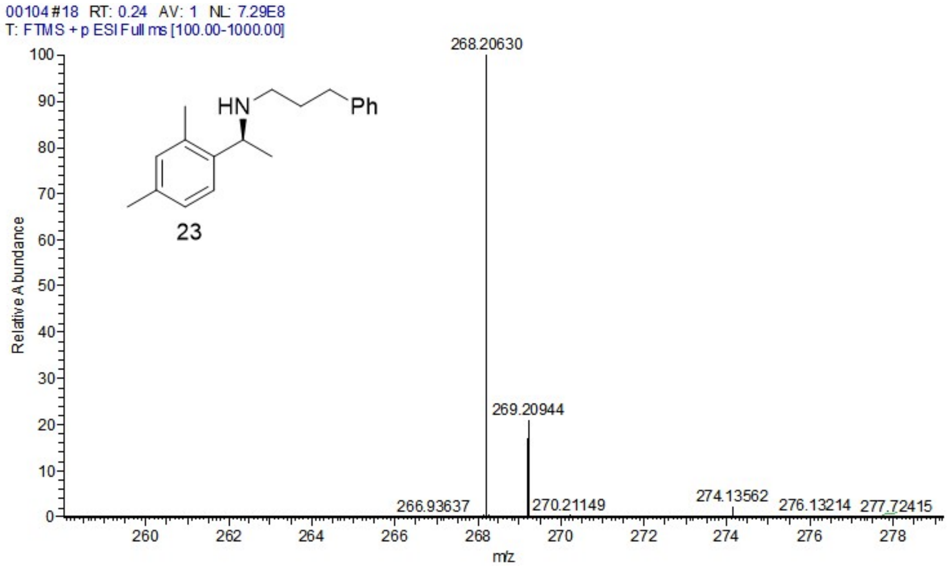

Supplementary Figure 121. HRMS of **23**.

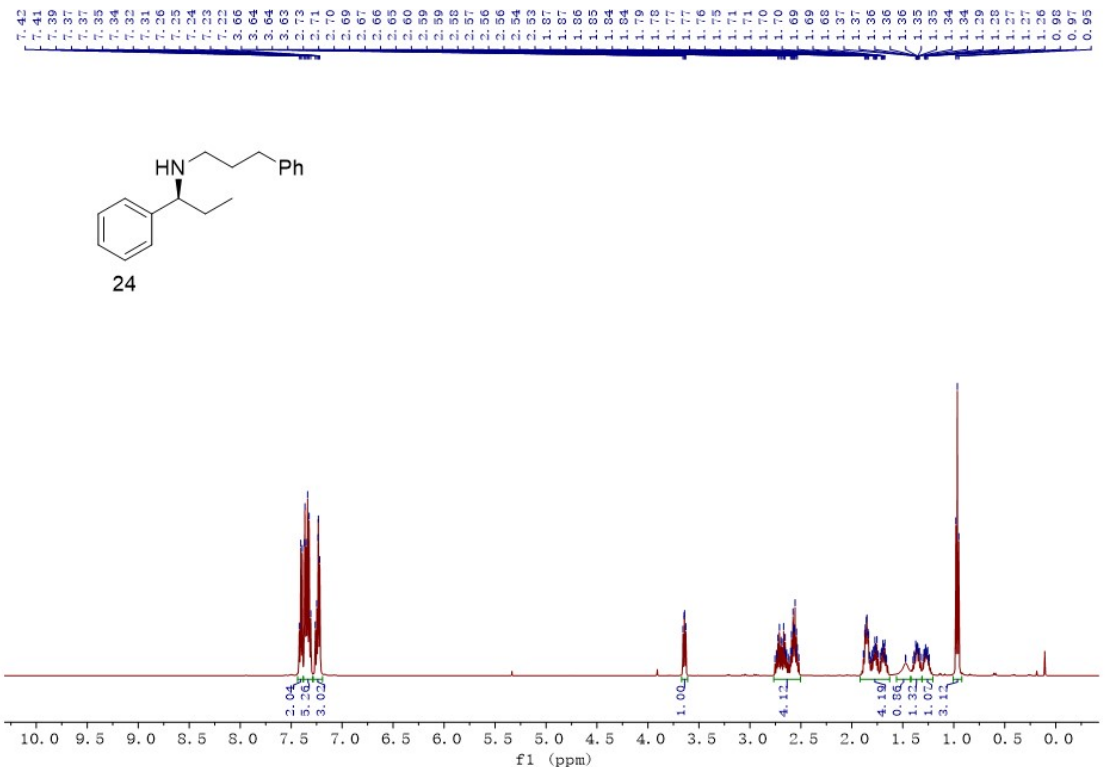

Supplementary Figure 122. ^{1}H NMR spectrum of **24** in $CDCl_3$.

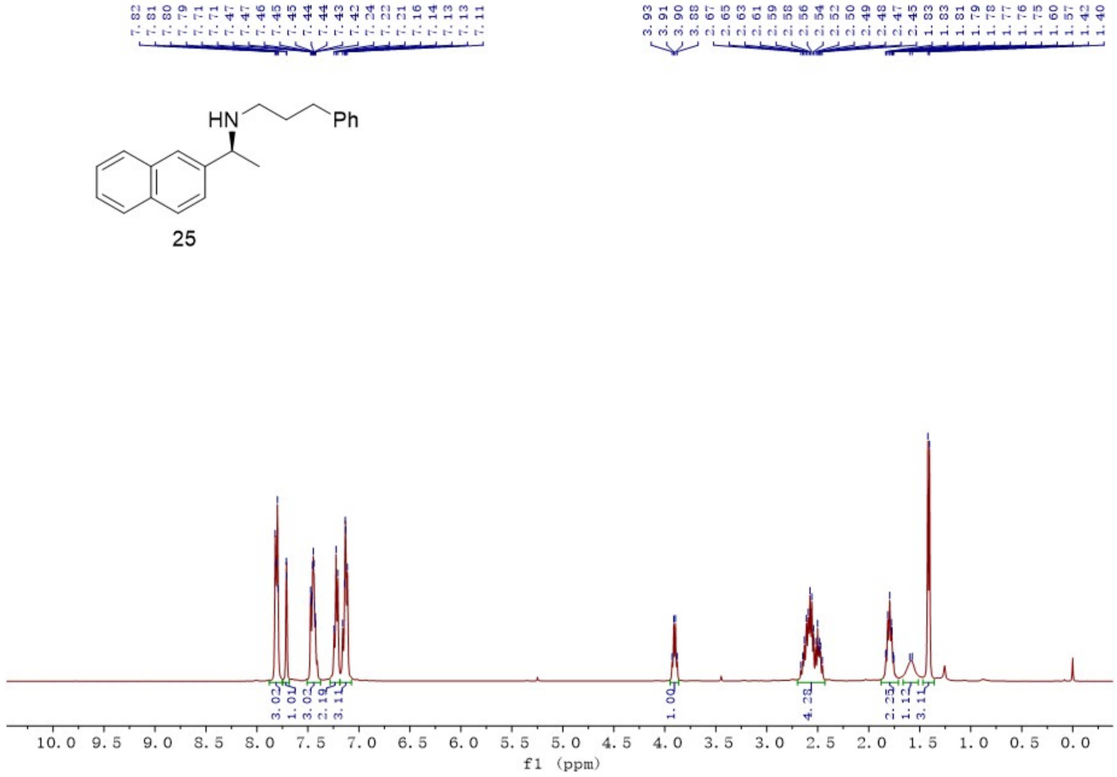

Supplementary Figure 123. ^{1}H NMR spectrum of **25** in $CDCl_3$.

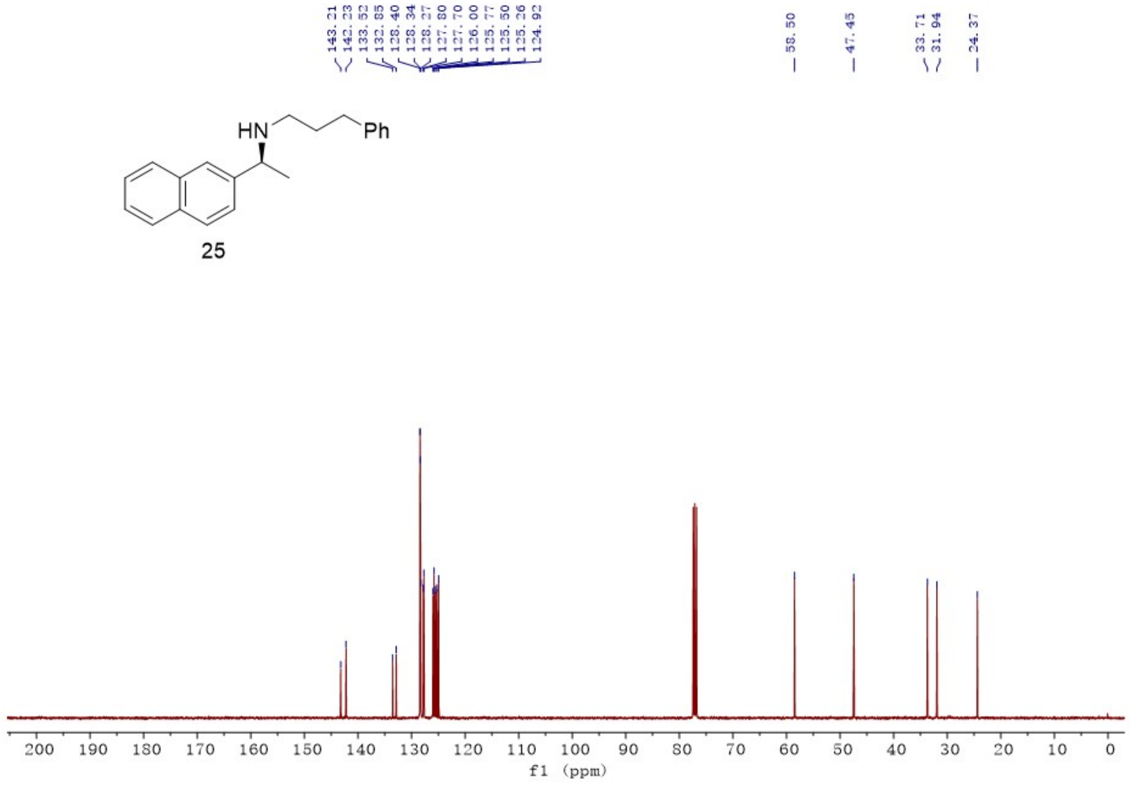

Supplementary Figure 124. ^{13}C NMR spectrum of **25** in $CDCl_3$.

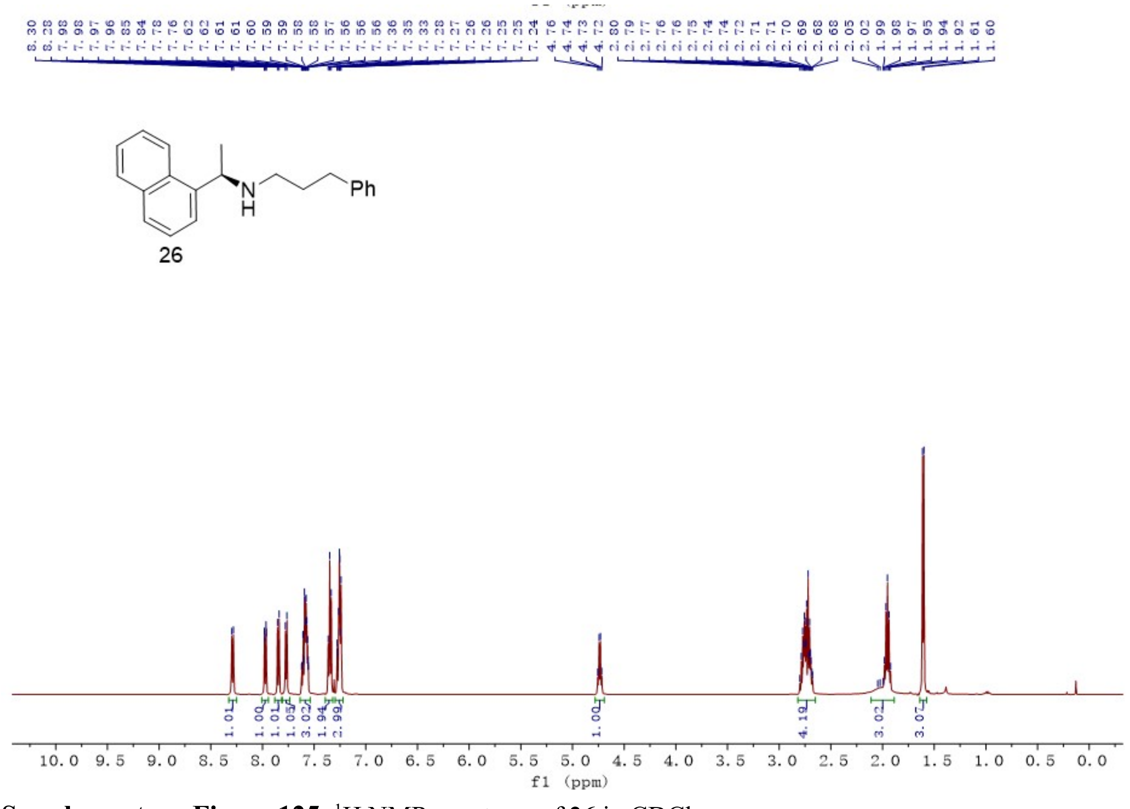

Supplementary Figure 125. ^{1}H NMR spectrum of **26** in $CDCl_3$.

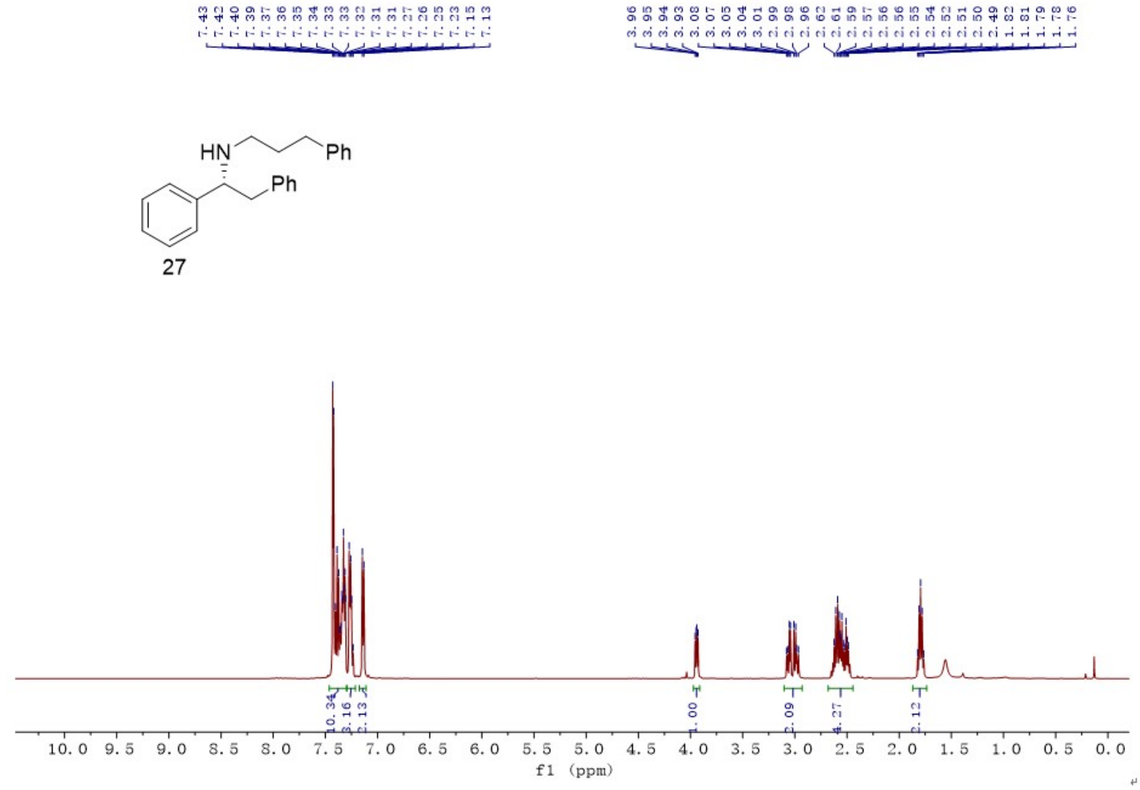

Supplementary Figure 126. ^{1}H NMR spectrum of **27** in $CDCl_3$.

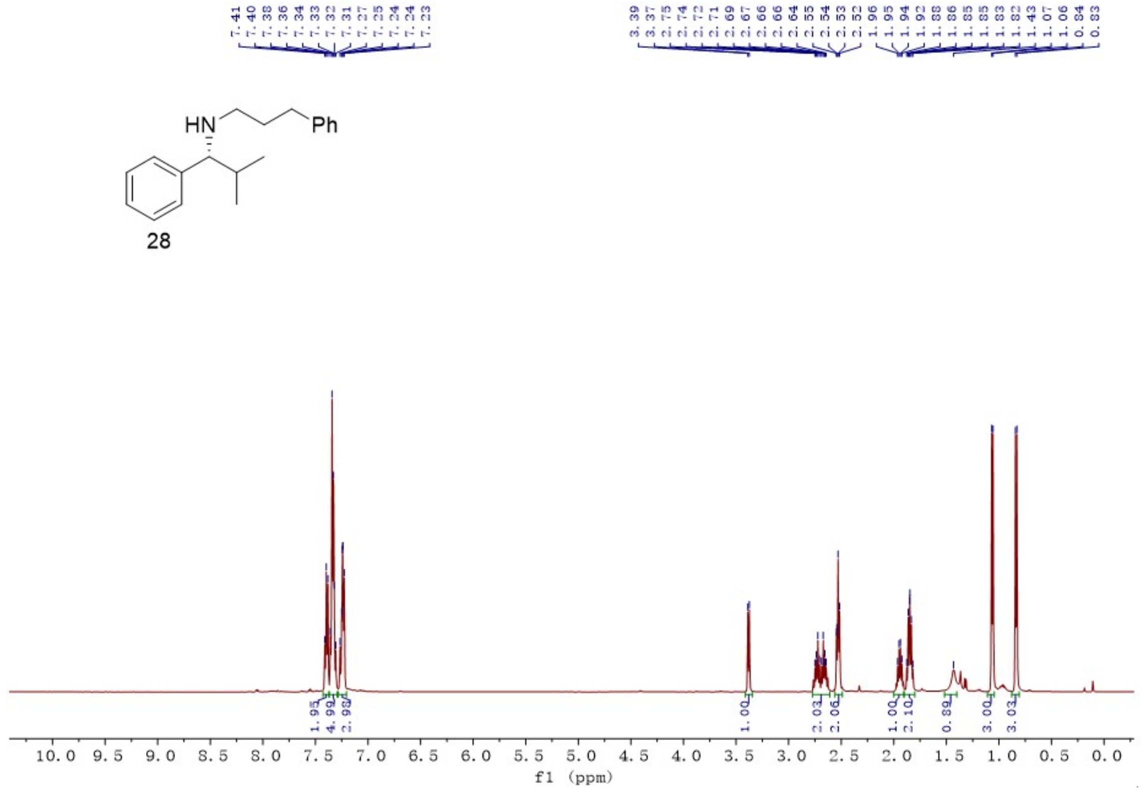

Supplementary Figure 127. ^{1}H NMR spectrum of **28** in $CDCl_3$.

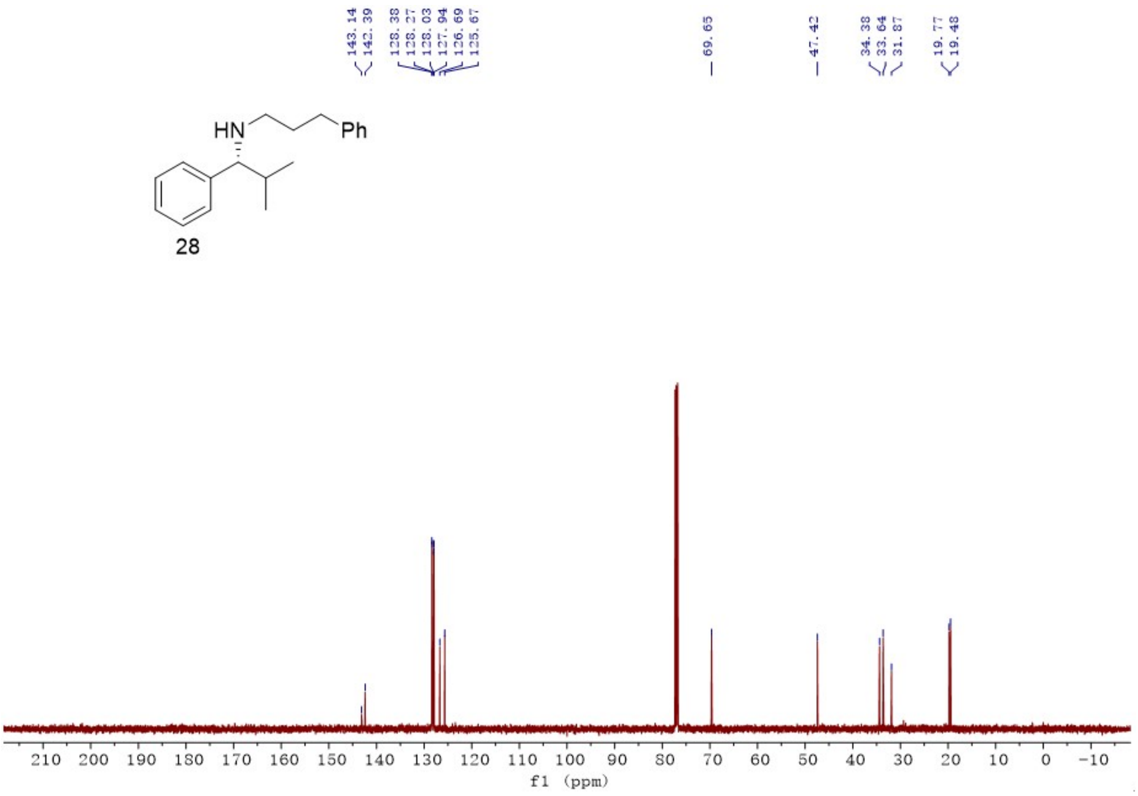

Supplementary Figure 128. ^{1}H NMR spectrum of **28** in $CDCl_3$.

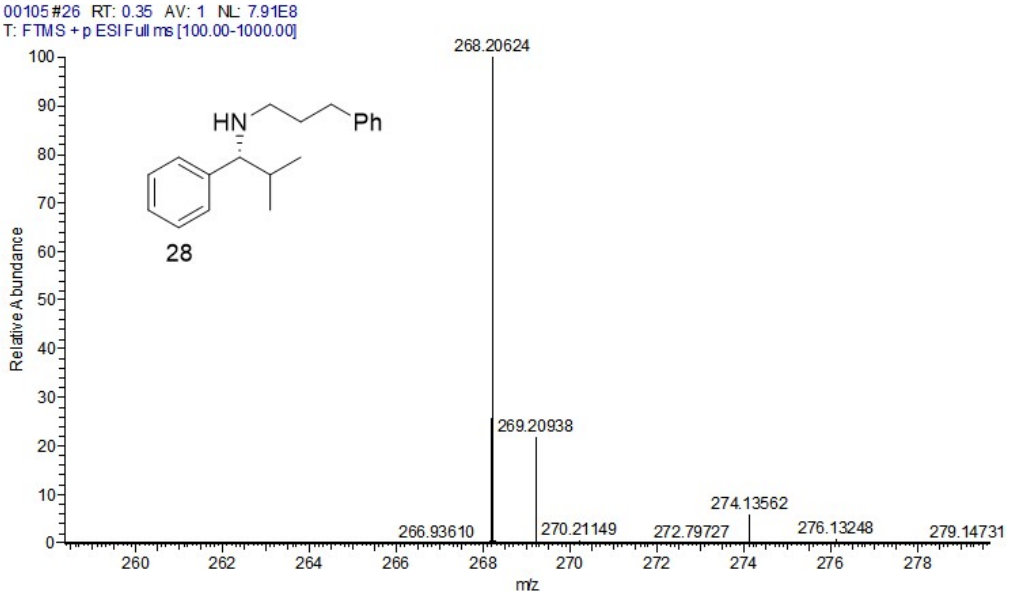

Supplementary Figure 129. HRMS of **28**.

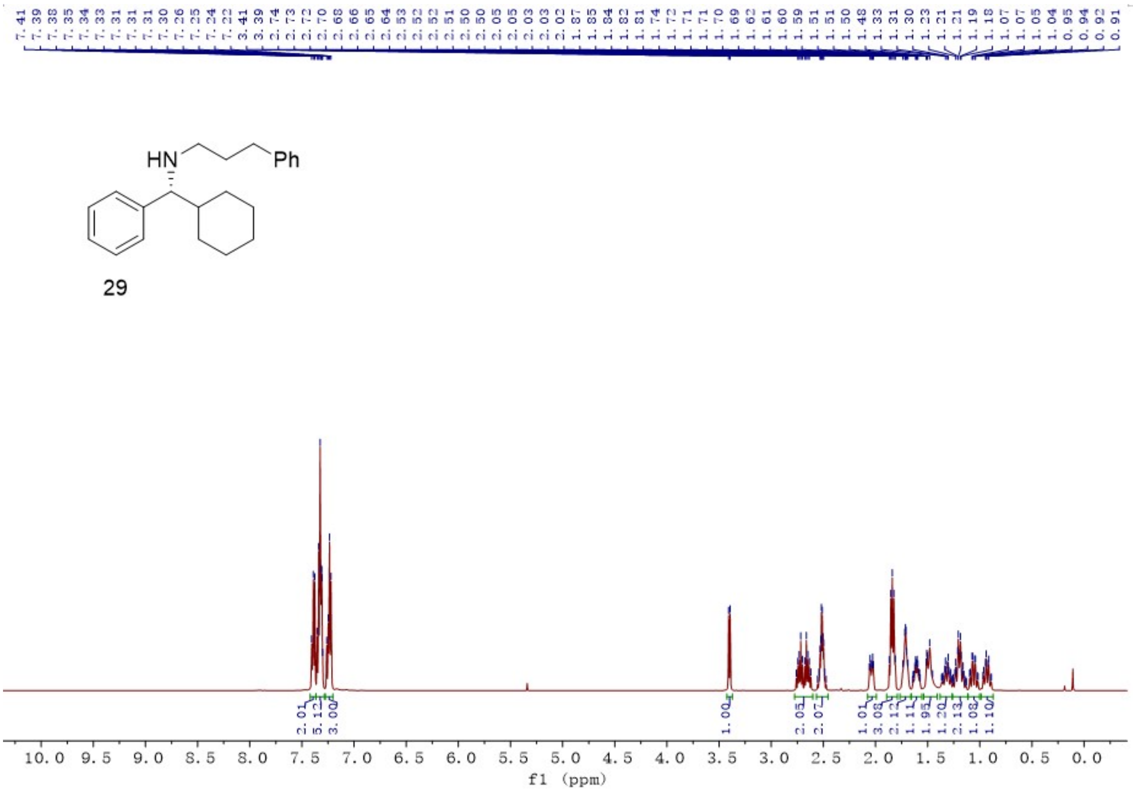

Supplementary Figure 130. ^{1}H NMR spectrum of **29** in $CDCl_3$.

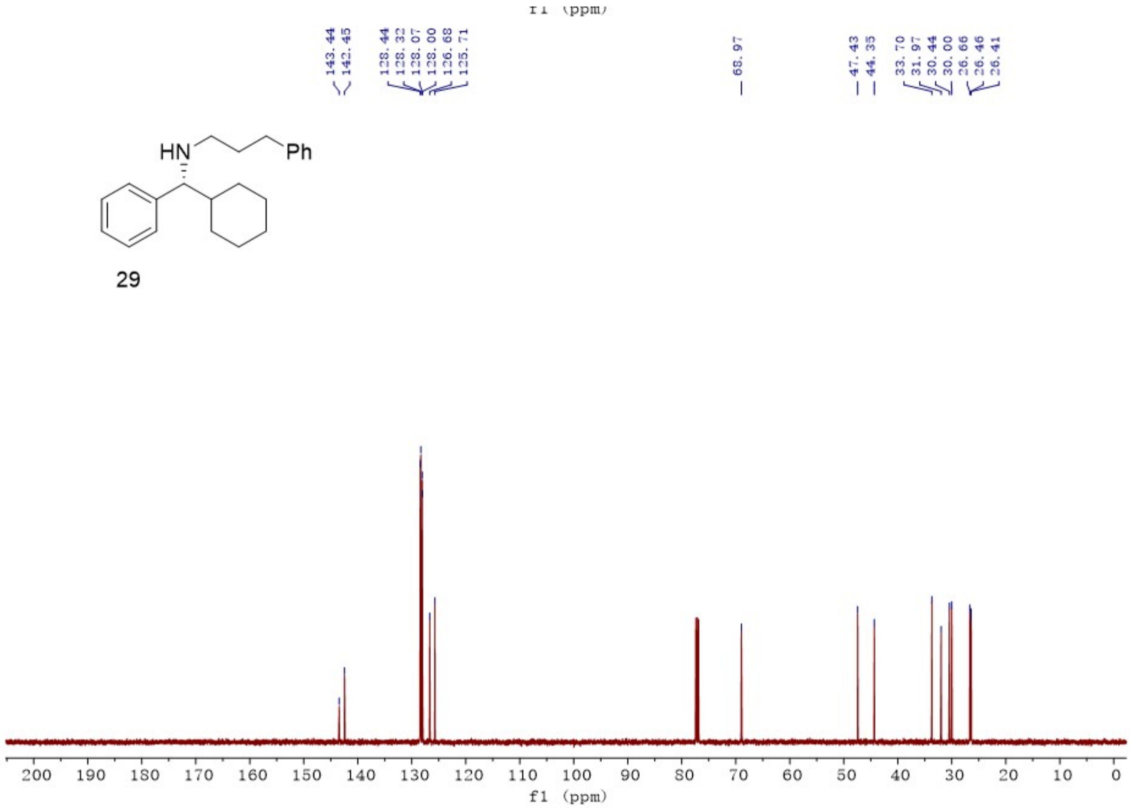

Supplementary Figure 131. ^{1}H NMR spectrum of **29** in $CDCl_3$.

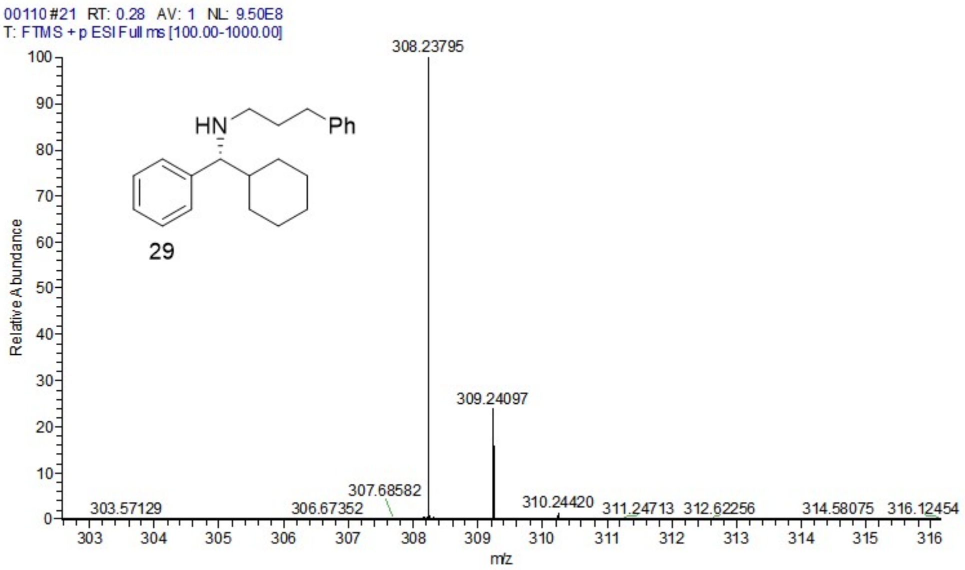

Supplementary Figure 132. HRMS of **29**.

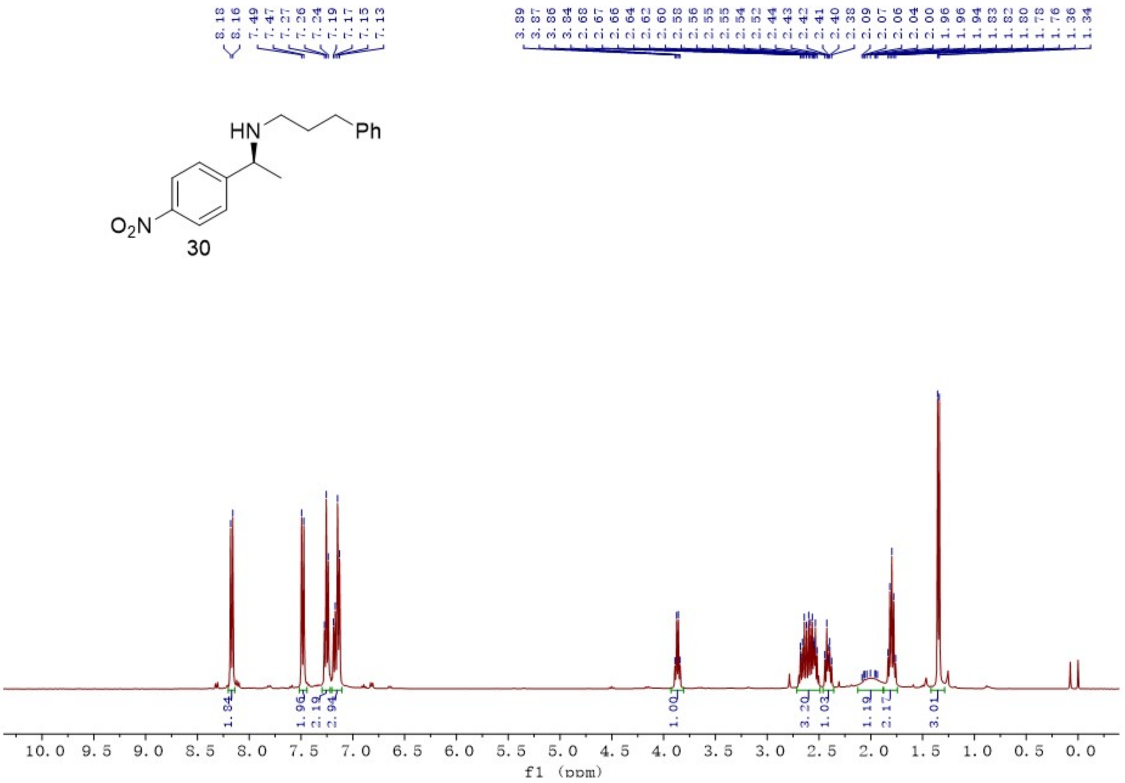

Supplementary Figure 133. ^{1}H NMR spectrum of **30** in $CDCl_3$.

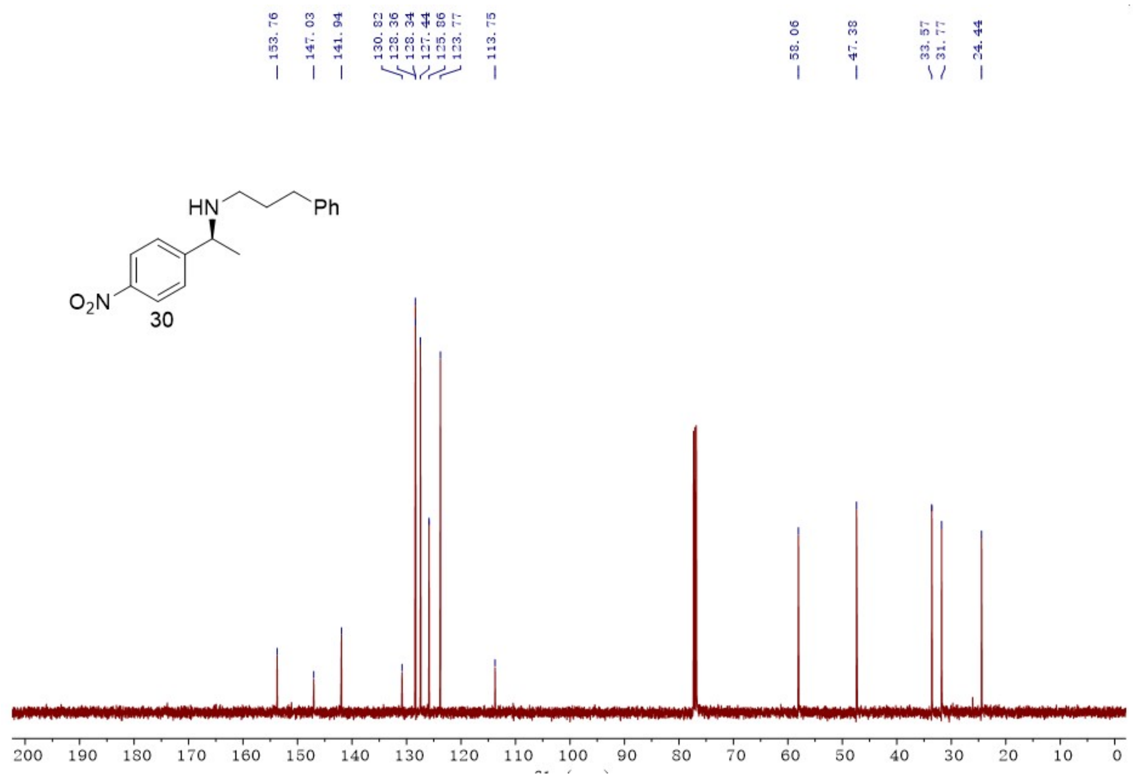

Supplementary Figure 134. ^{13}C NMR spectrum of **30** in $CDCl_3$.

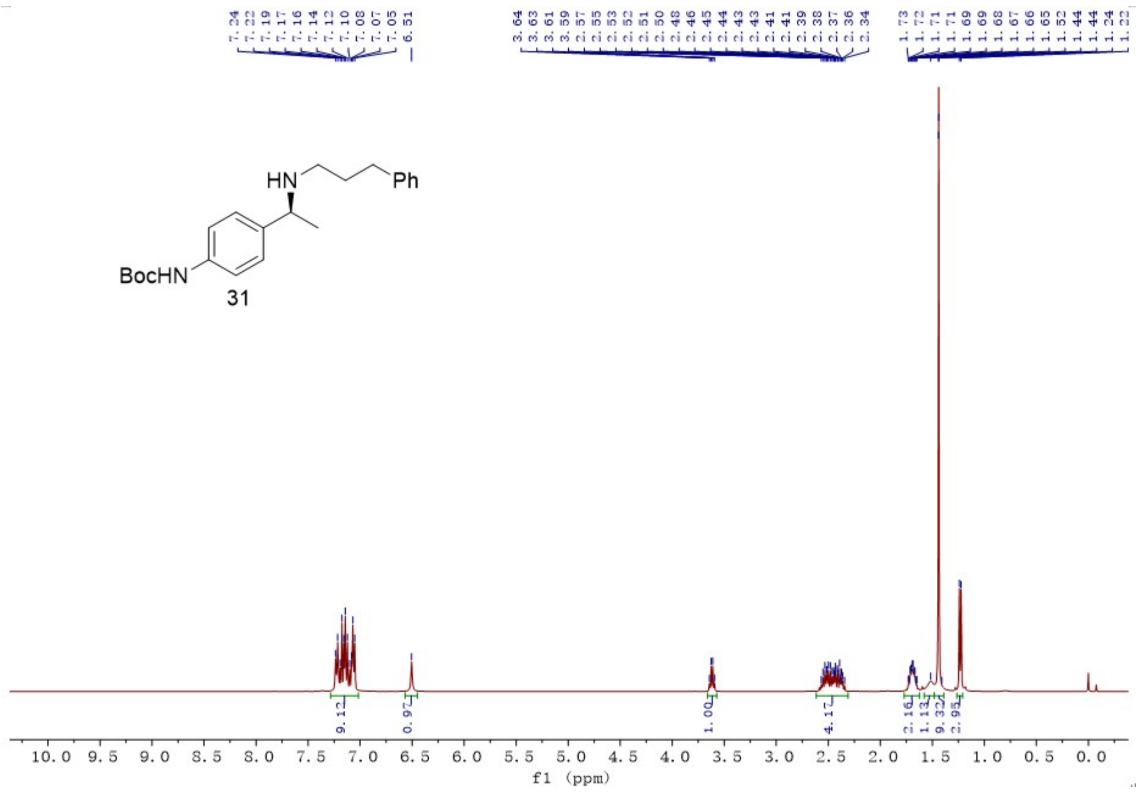

Supplementary Figure 135. ^{1}H NMR spectrum of **31** in $CDCl_3$.

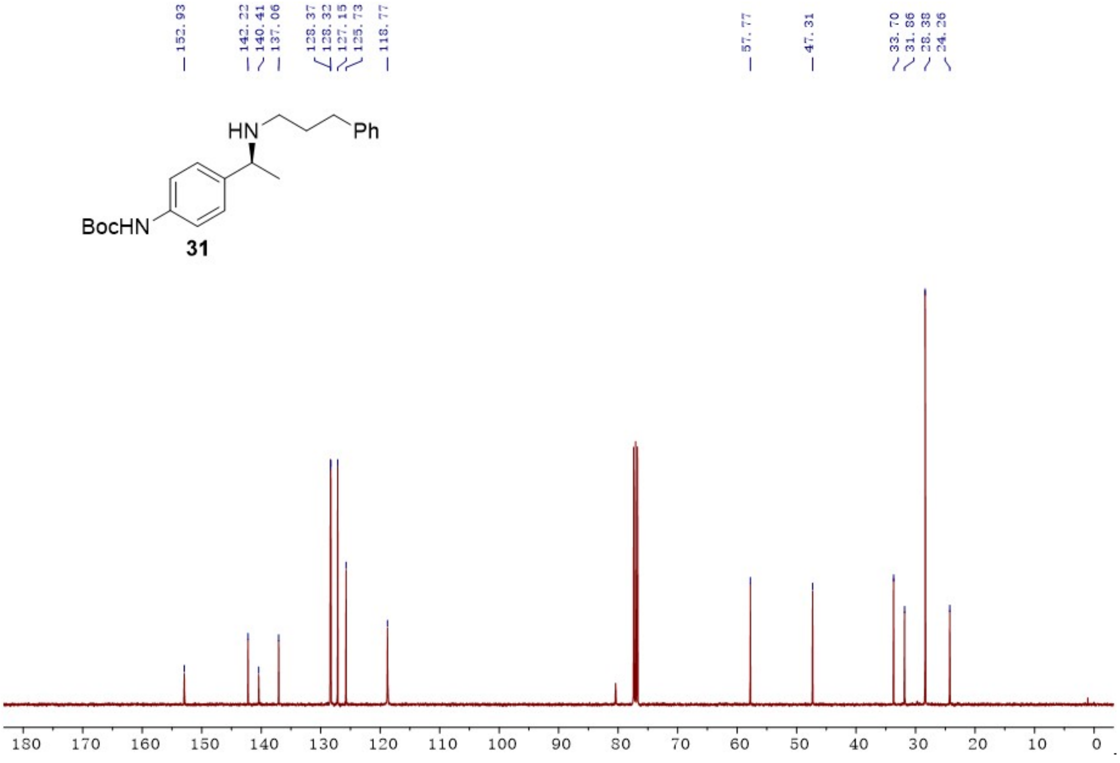

Supplementary Figure 136. ^{13}C NMR spectrum of **30** in $CDCl_3$.

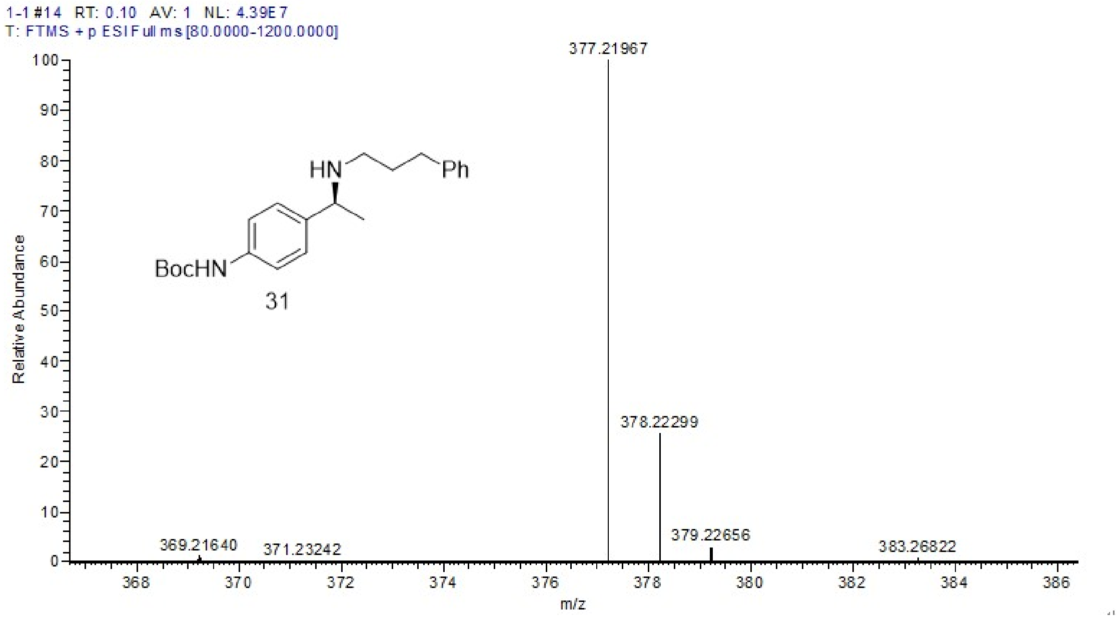

Supplementary Figure 137. HRMS of **31**.

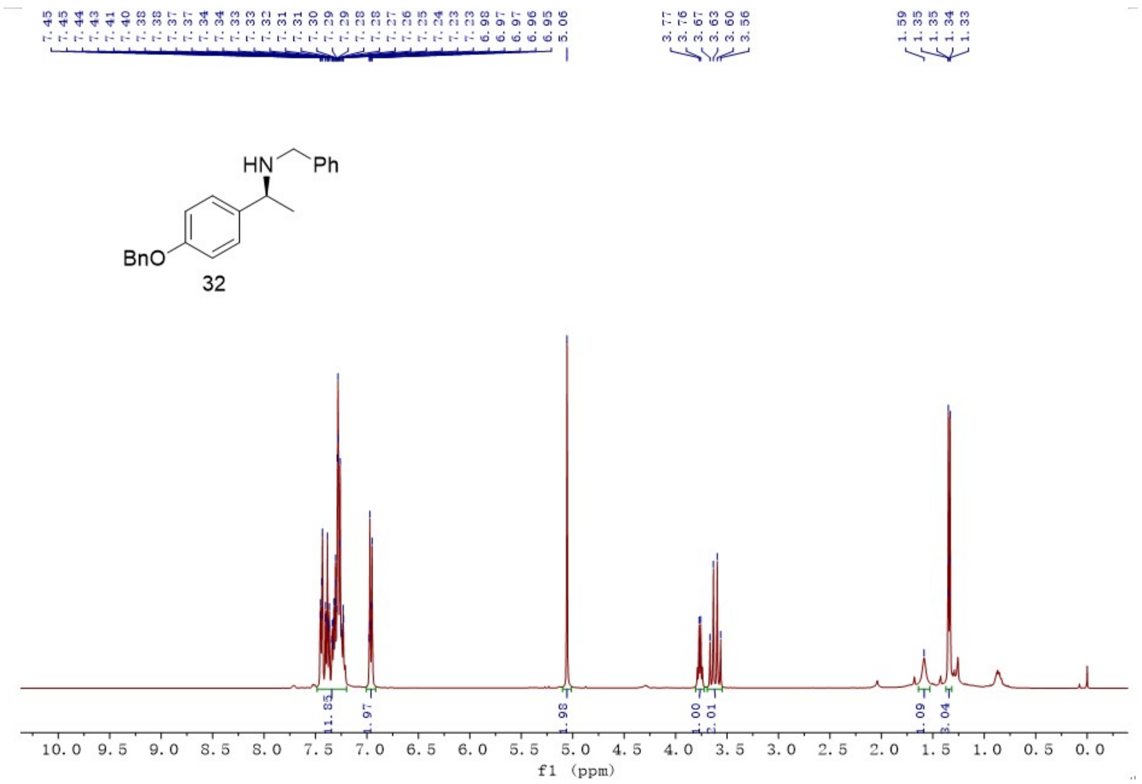

Supplementary Figure 138. ^{1}H NMR spectrum of **32** in $CDCl_3$.

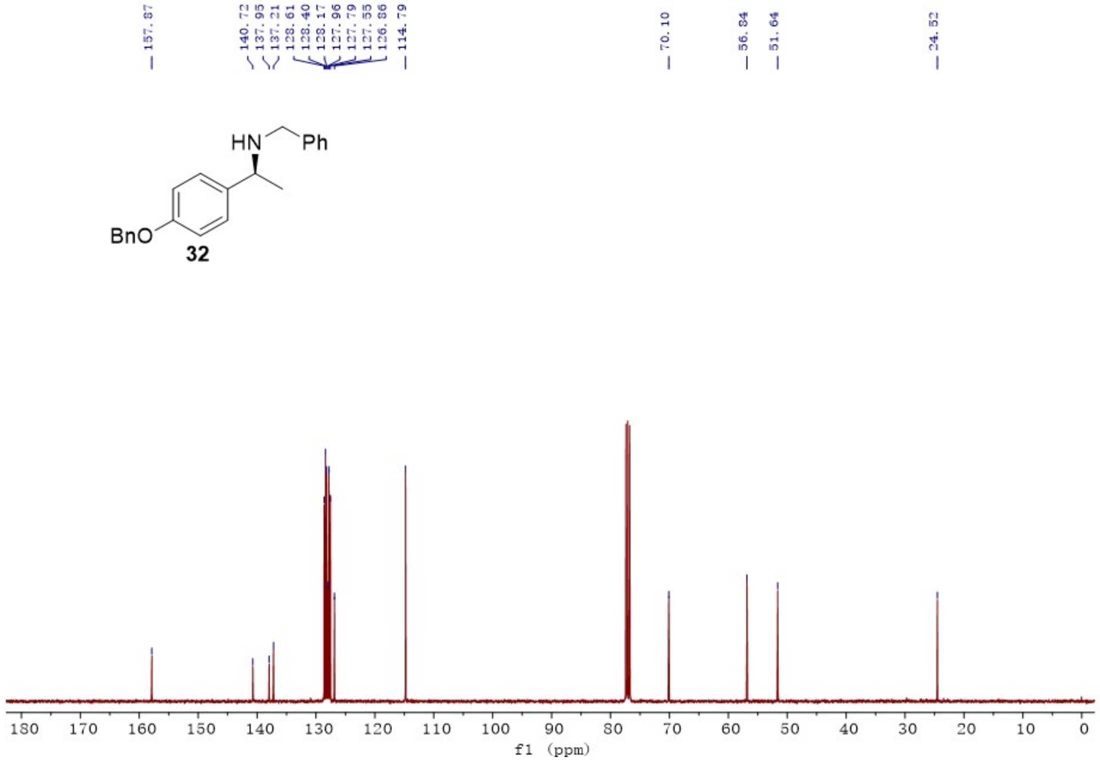

Supplementary Figure 139. ^{13}C NMR spectrum of **32** in $CDCl_3$.

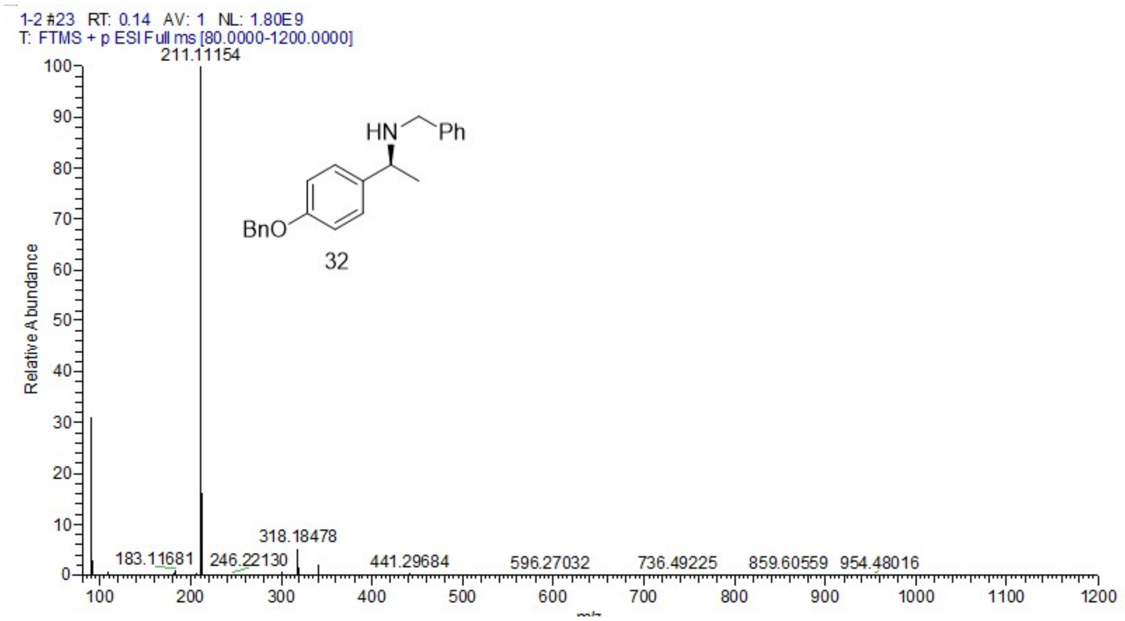

Supplementary Figure 140. HRMS of **32**.

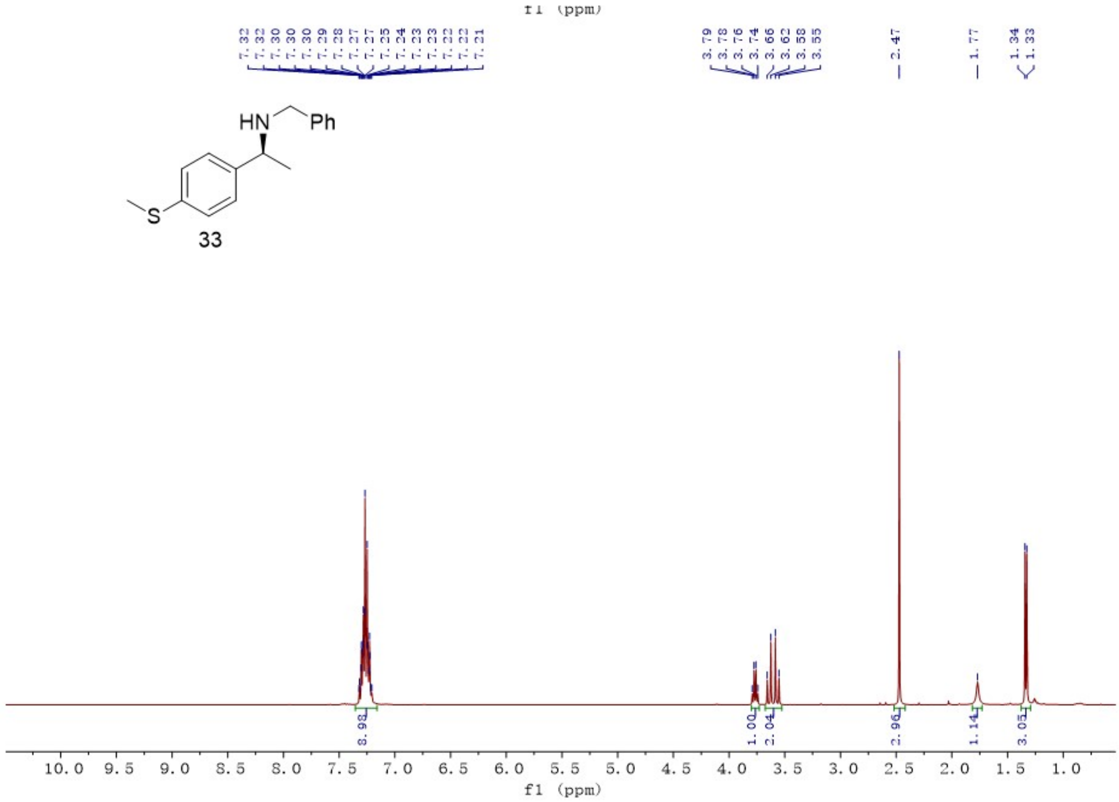

Supplementary Figure 141. ^{1}H NMR spectrum of **33** in $CDCl_3$.

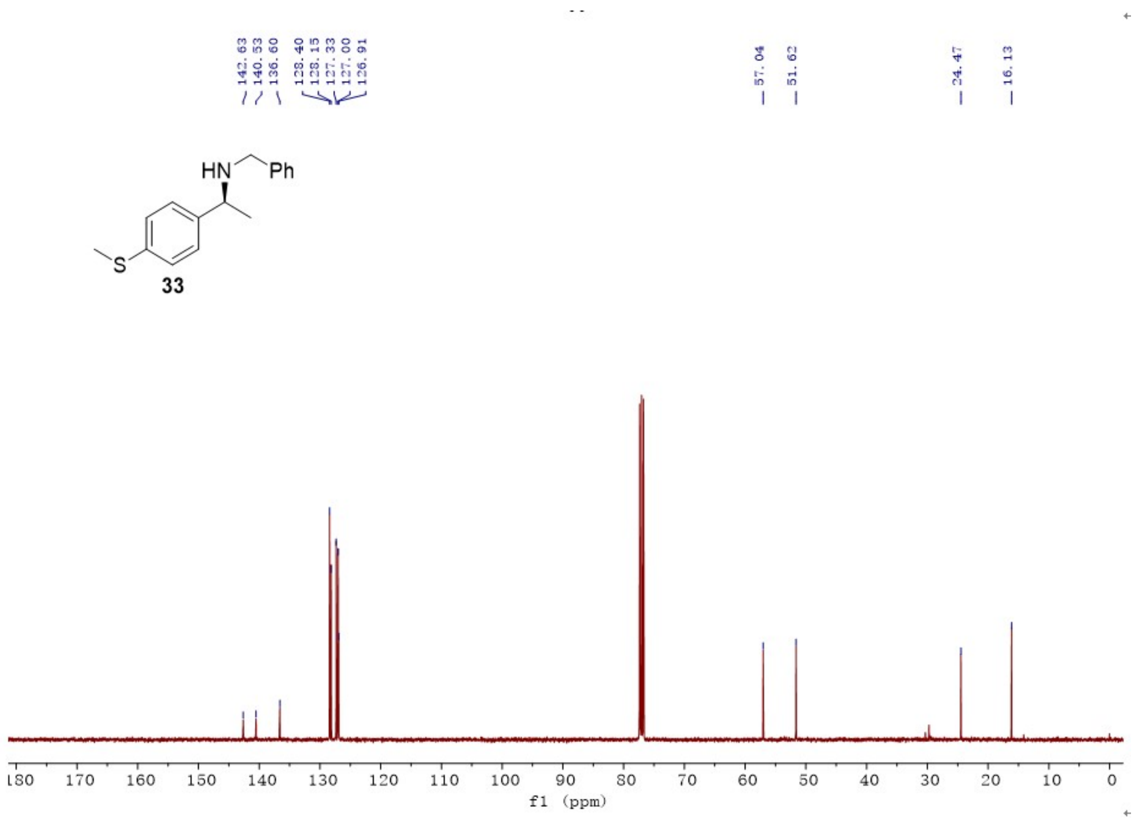

Supplementary Figure 142. ^{13}C NMR spectrum of **33** in $CDCl_3$.

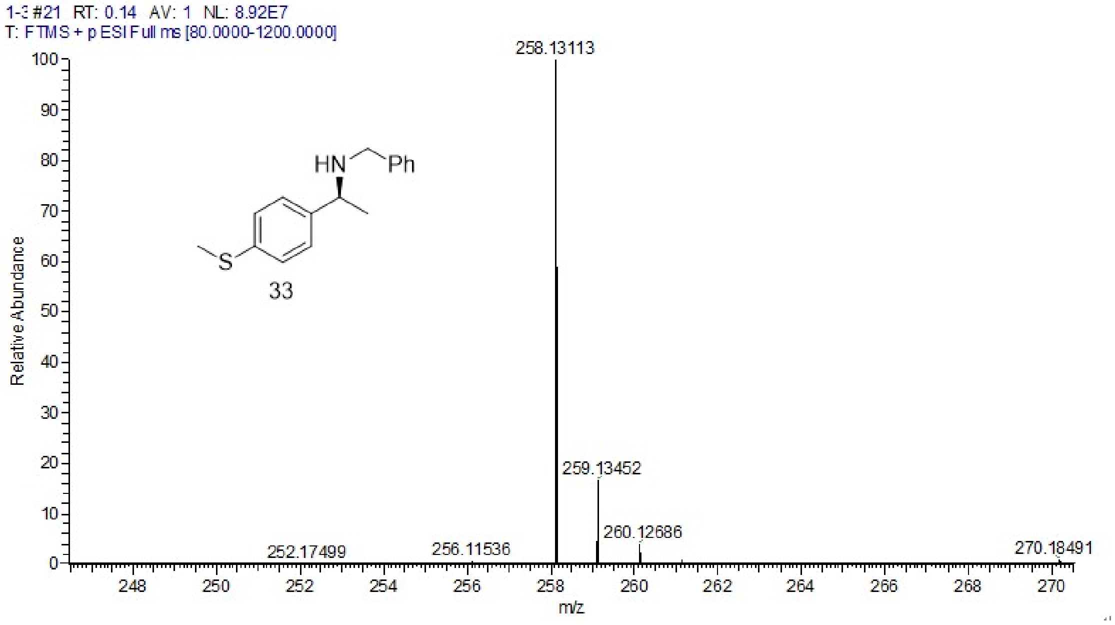

Supplementary Figure 143. HRMS of **33**.

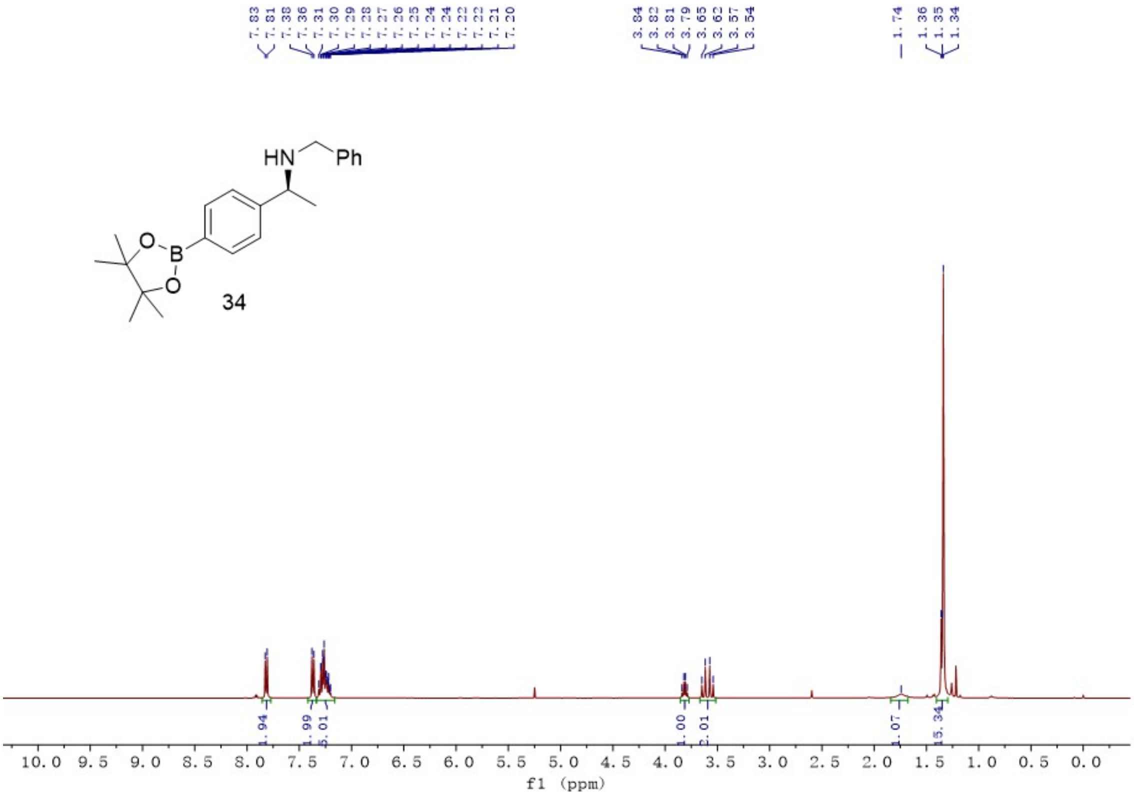

Supplementary Figure 144. ^{1}H NMR spectrum of **34** in $CDCl_3$.

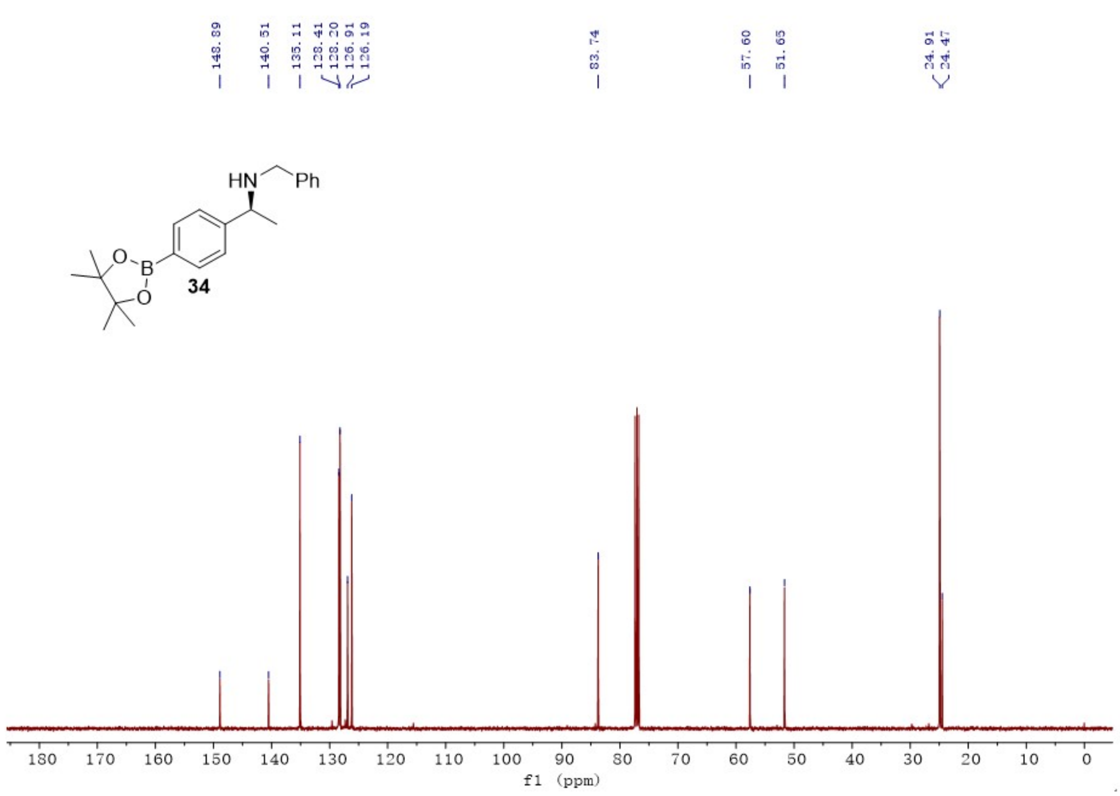

Supplementary Figure 145. ^{13}C NMR spectrum of **34** in $CDCl_3$.

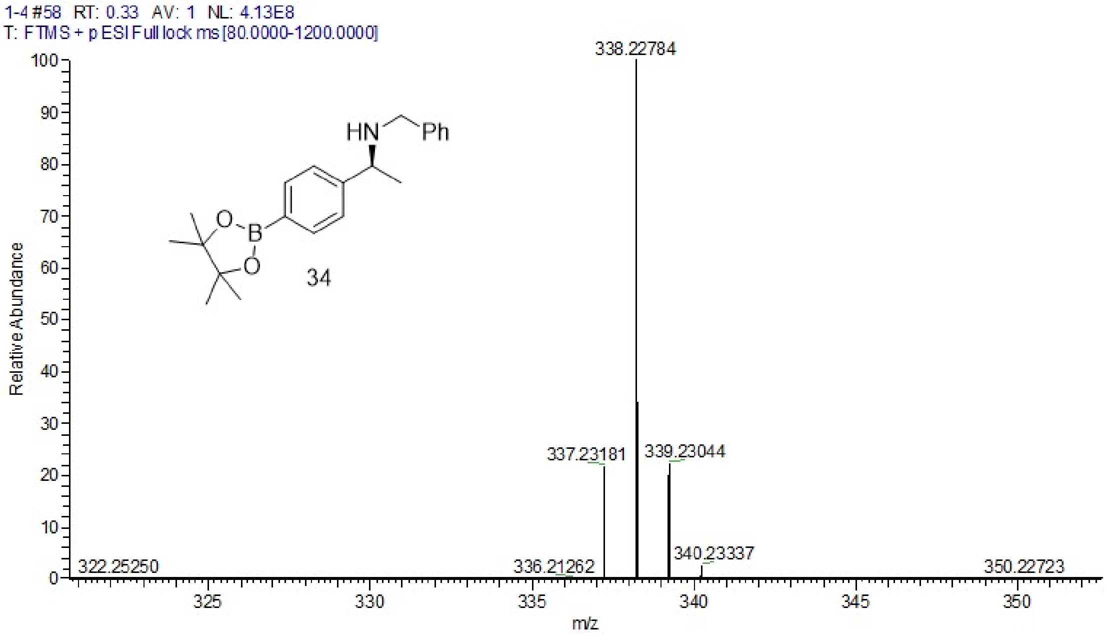

Supplementary Figure 146. HRMS of **34**.

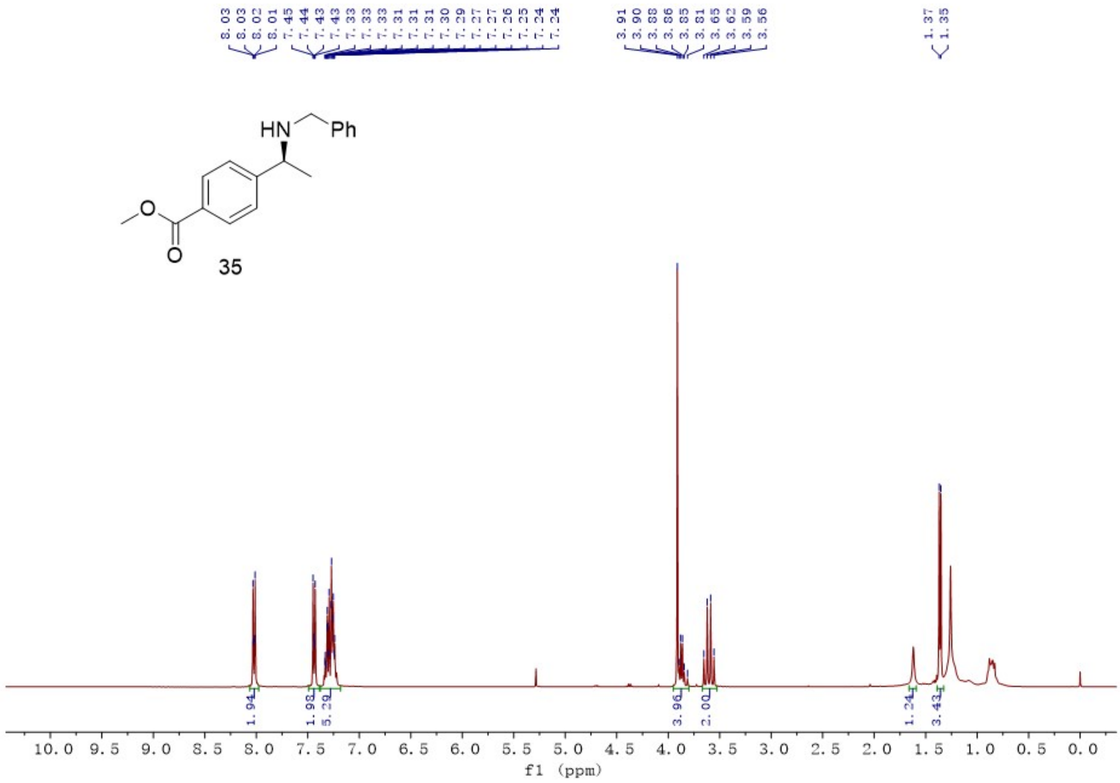

Supplementary Figure 147. ^{1}H NMR spectrum of **35** in $CDCl_3$.

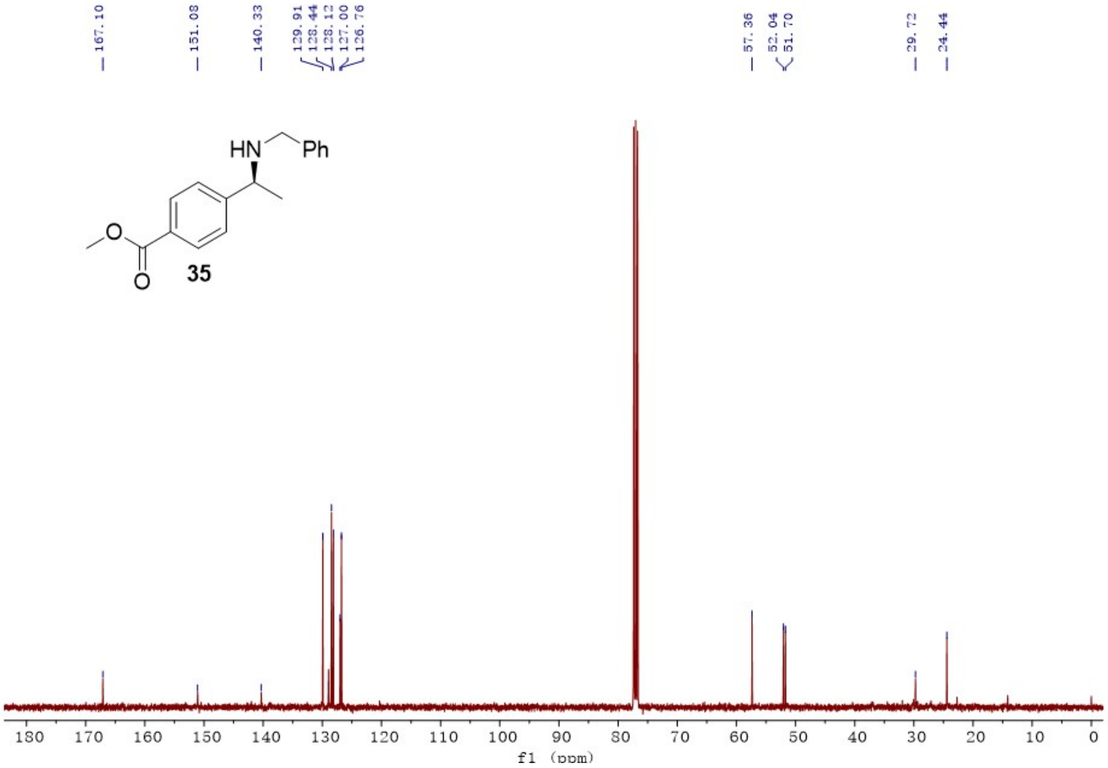

Supplementary Figure 148. ^{13}C NMR spectrum of **35** in $CDCl_3$.

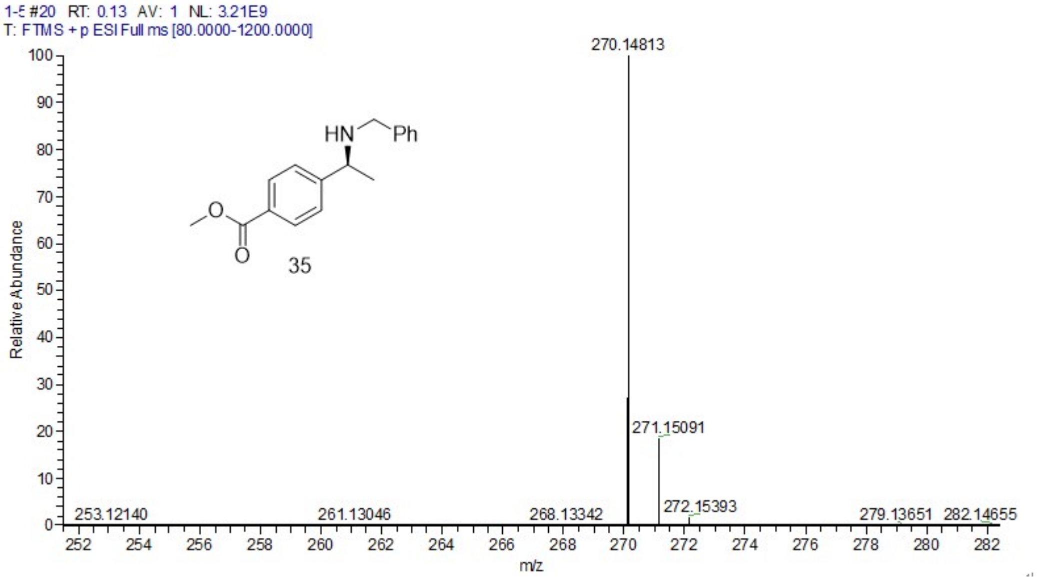

Supplementary Figure 149. HRMS of **35**

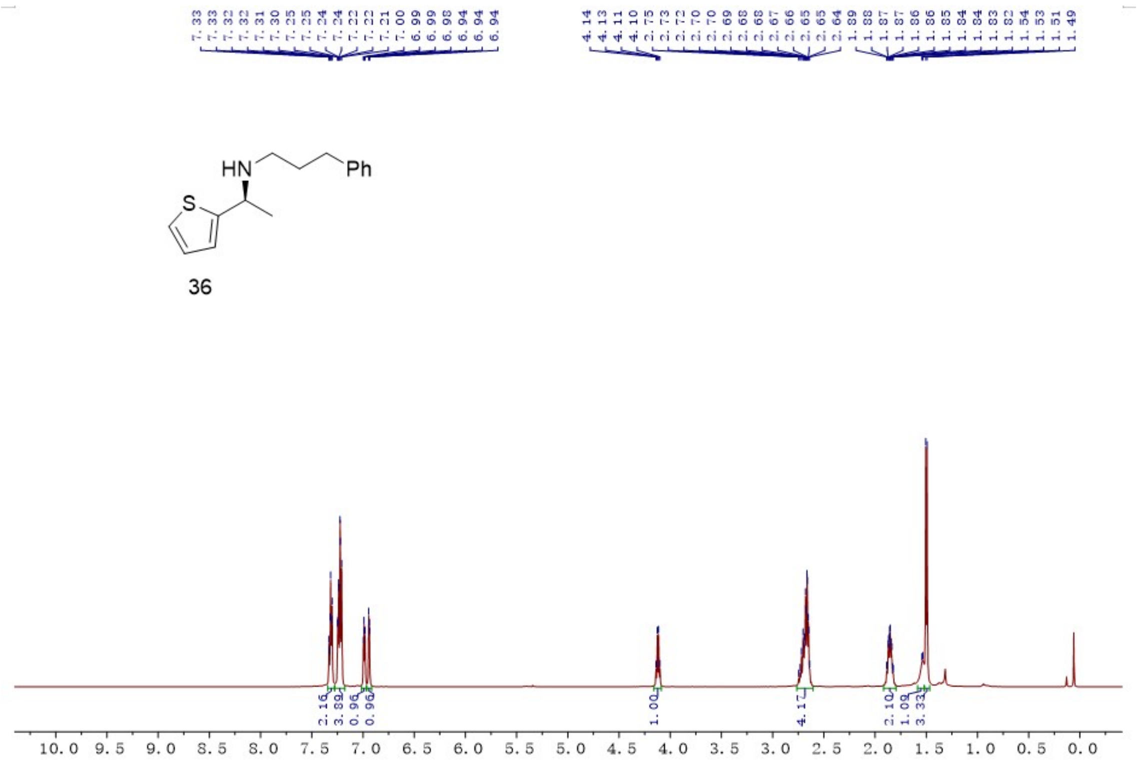

Supplementary Figure 150. ^{1}H NMR spectrum of **36** in $CDCl_3$.

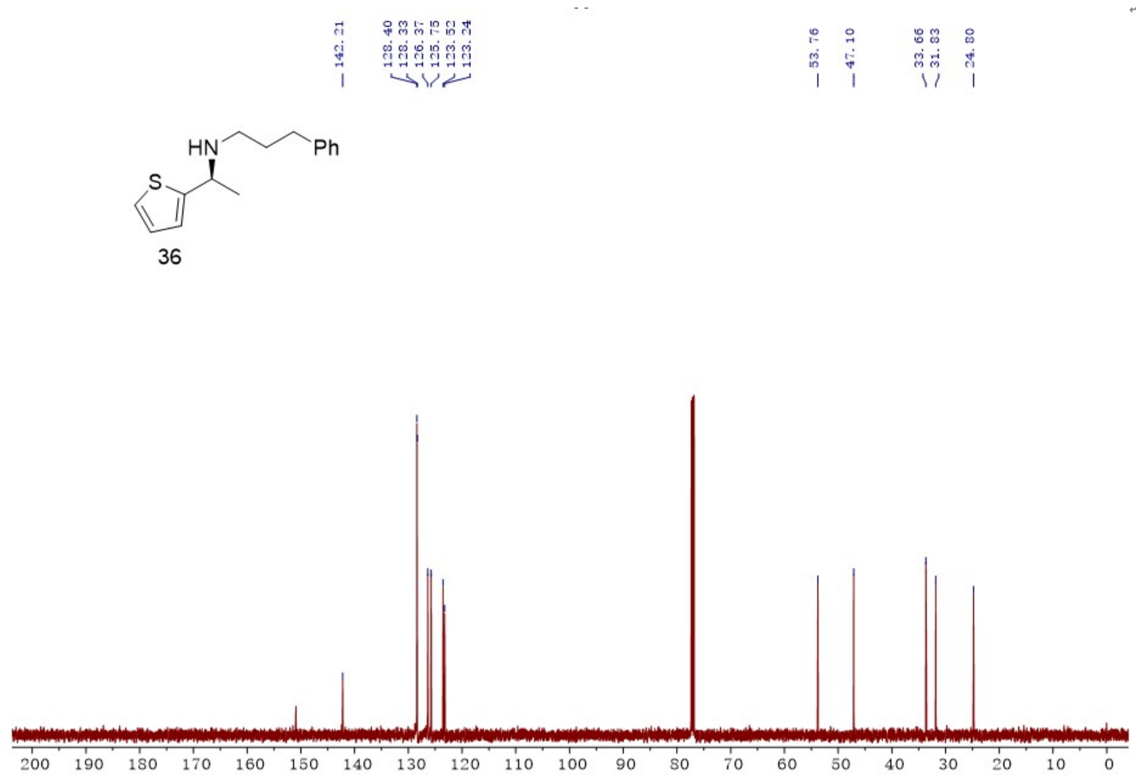

Supplementary Figure 151. ^{13}C NMR spectrum of **36** in $CDCl_3$.

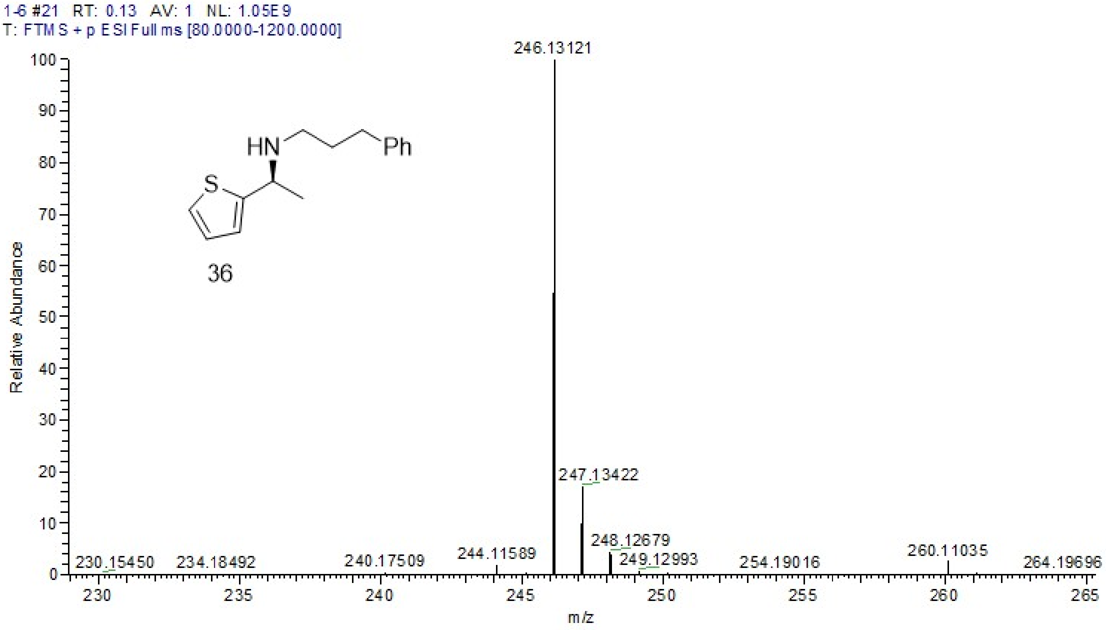

Supplementary Figure 152. HRMS of **36**.

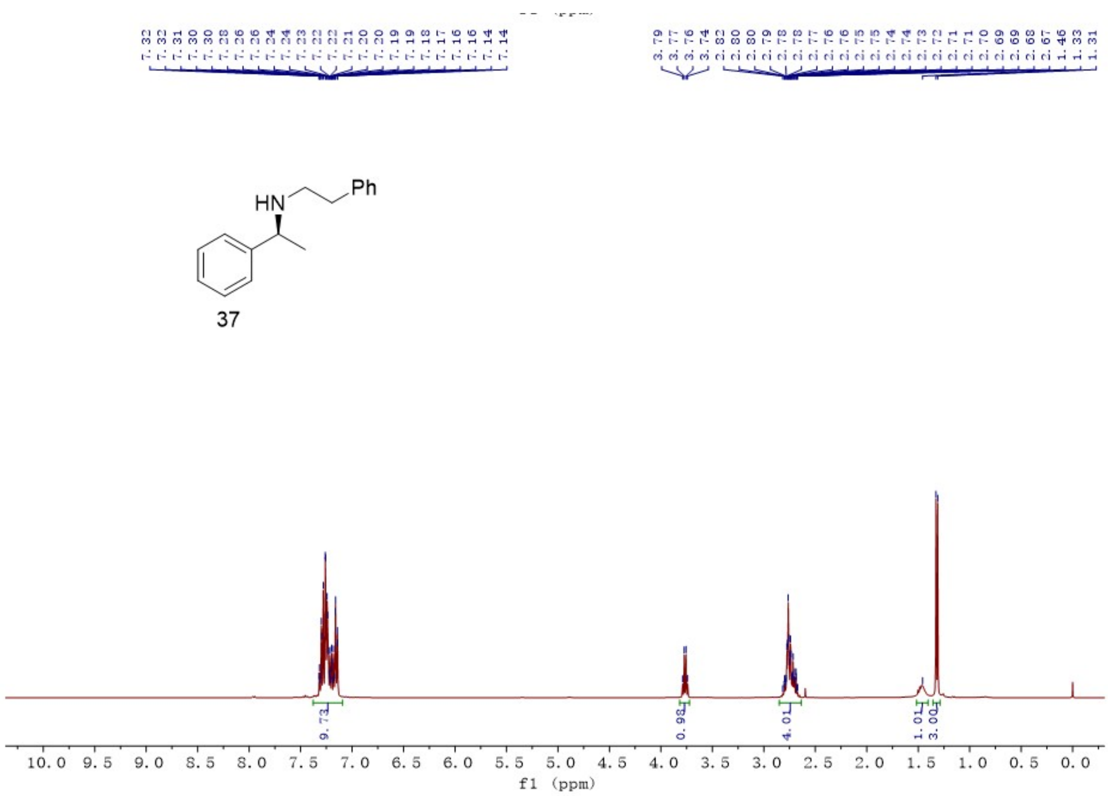

Supplementary Figure 153. ^{1}H NMR spectrum of **37** in $CDCl_3$.

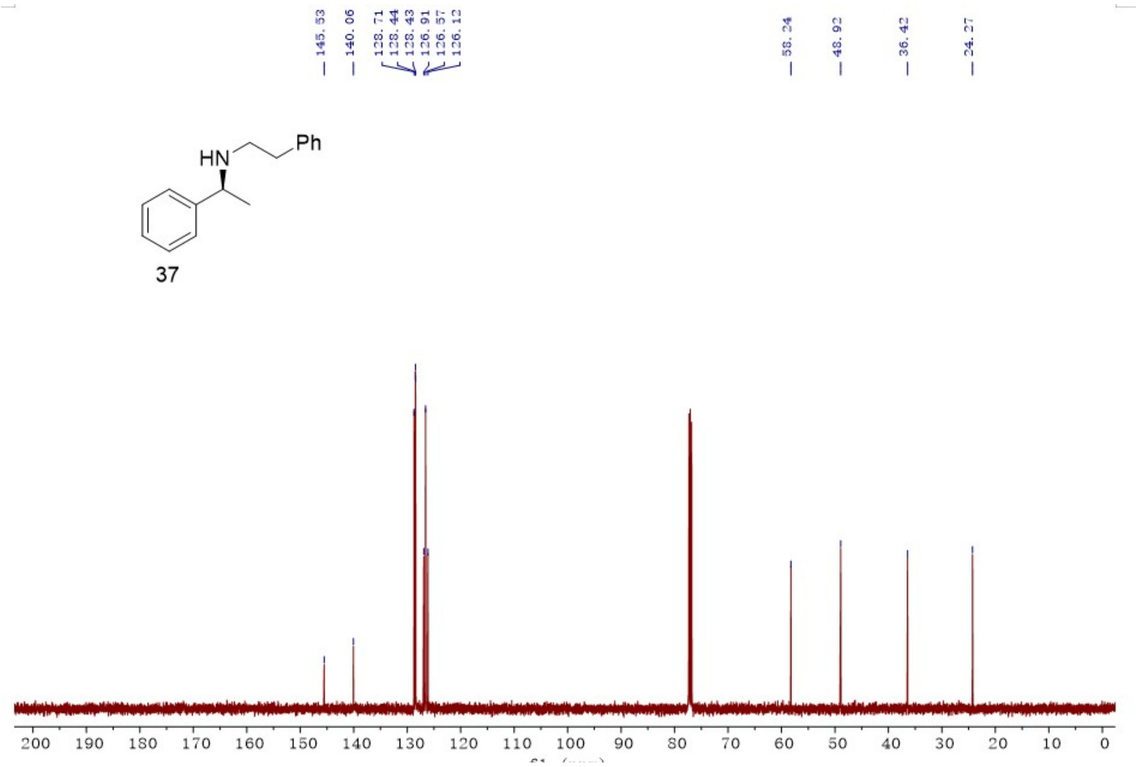

Supplementary Figure 154. ^{13}C NMR spectrum of **37** in $CDCl_3$.

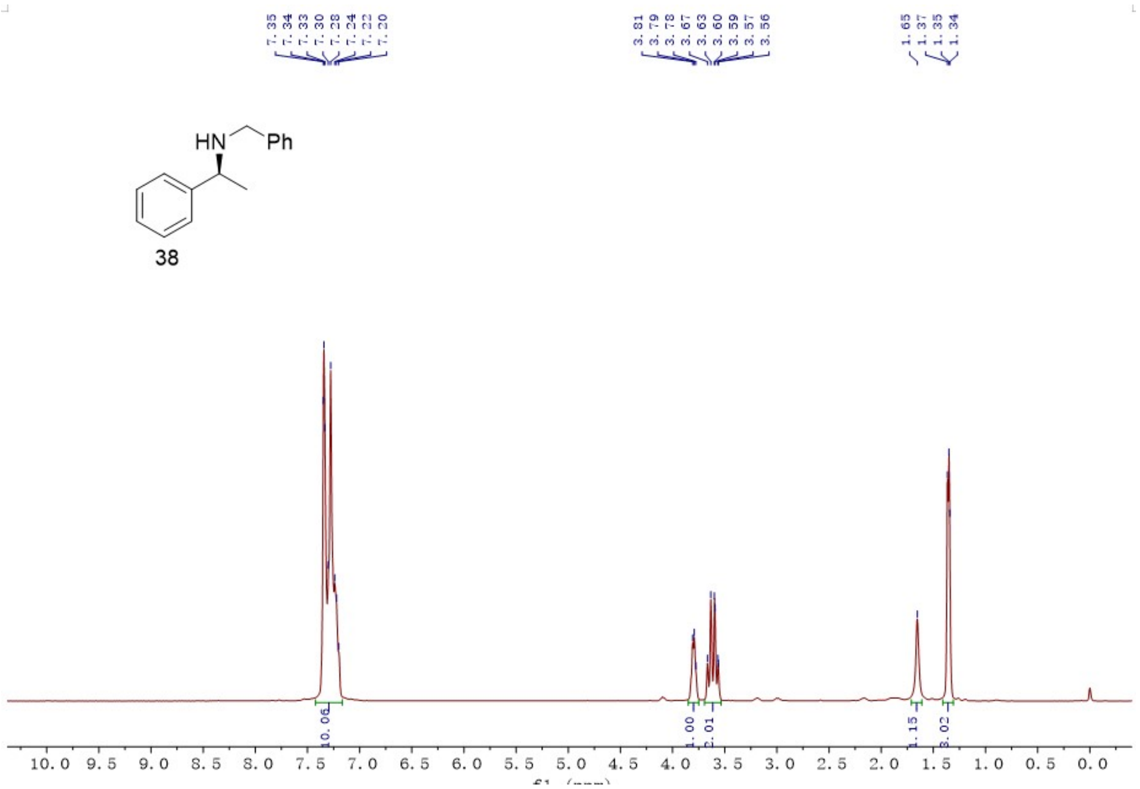

Supplementary Figure 155. ^{1}H NMR spectrum of **38** in $CDCl_3$.

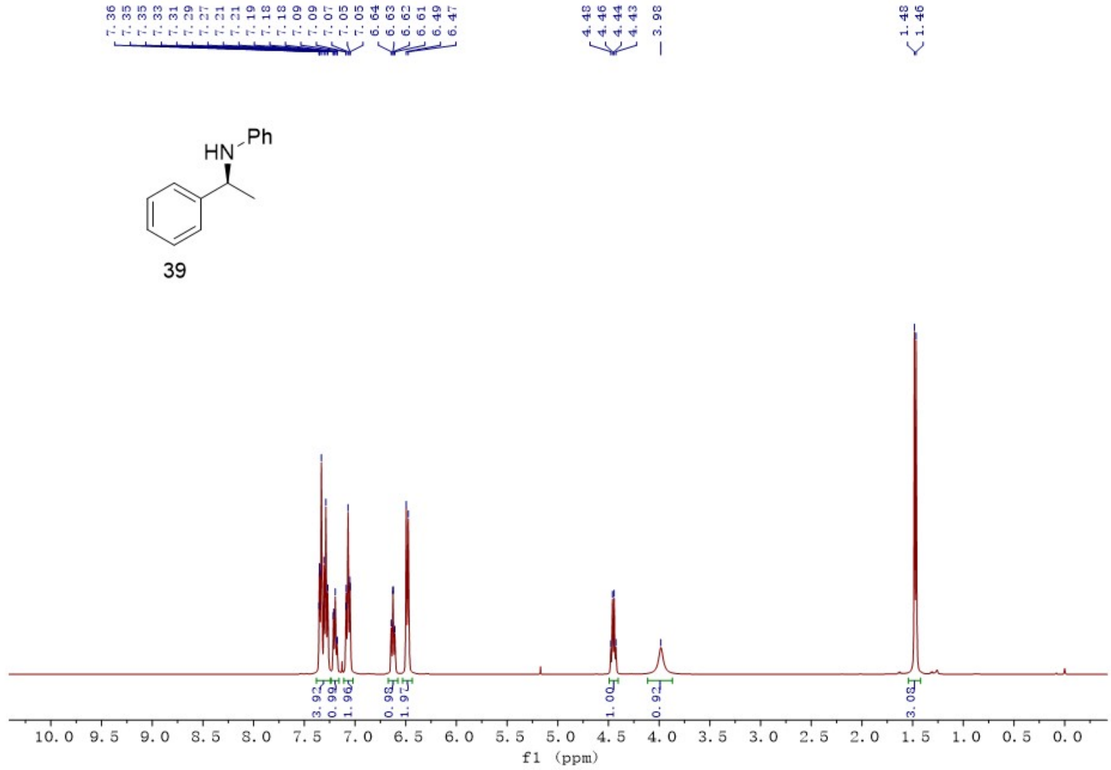

Supplementary Figure 156. ^{1}H NMR spectrum of **39** in $CDCl_3$.

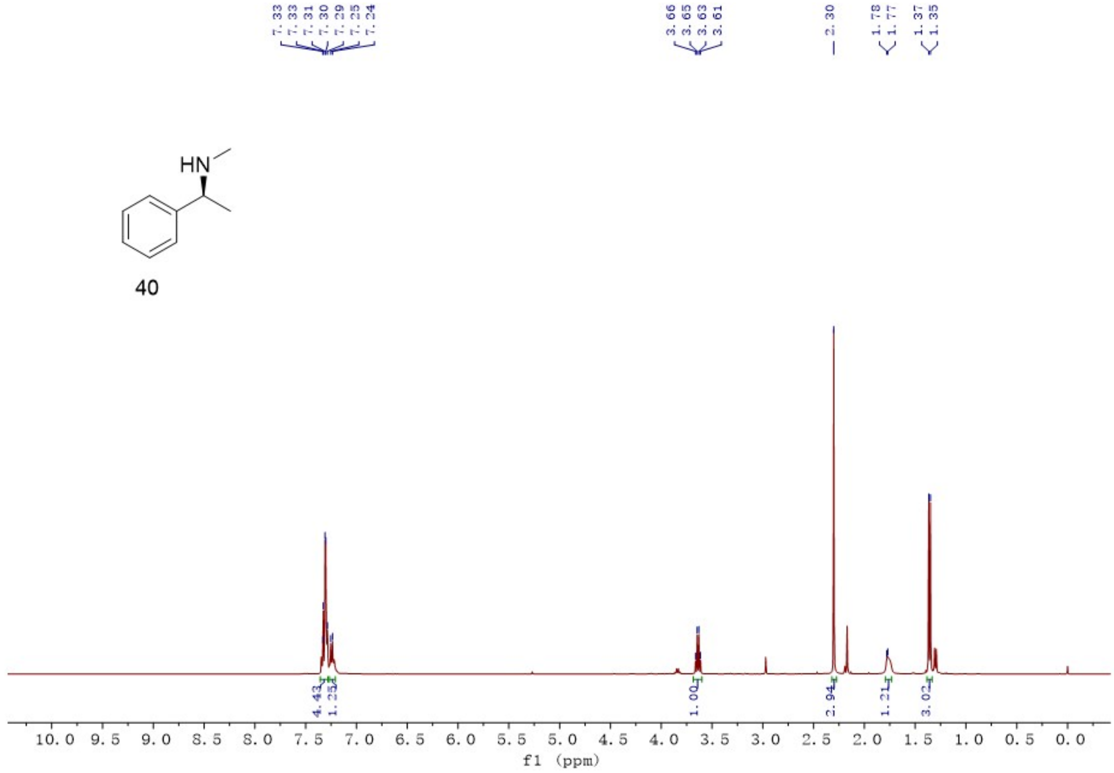

Supplementary Figure 157. ^{1}H NMR spectrum of **40** in $CDCl_3$.

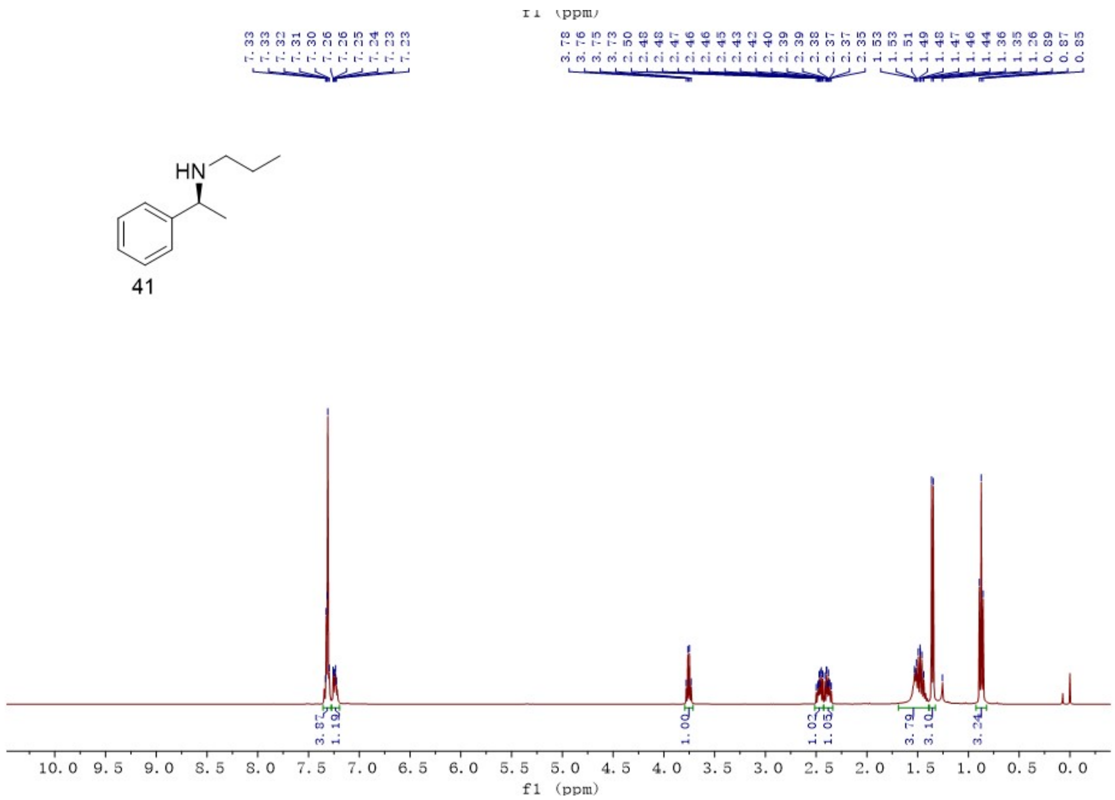

Supplementary Figure 158. ^{1}H NMR spectrum of **41** in $CDCl_3$.

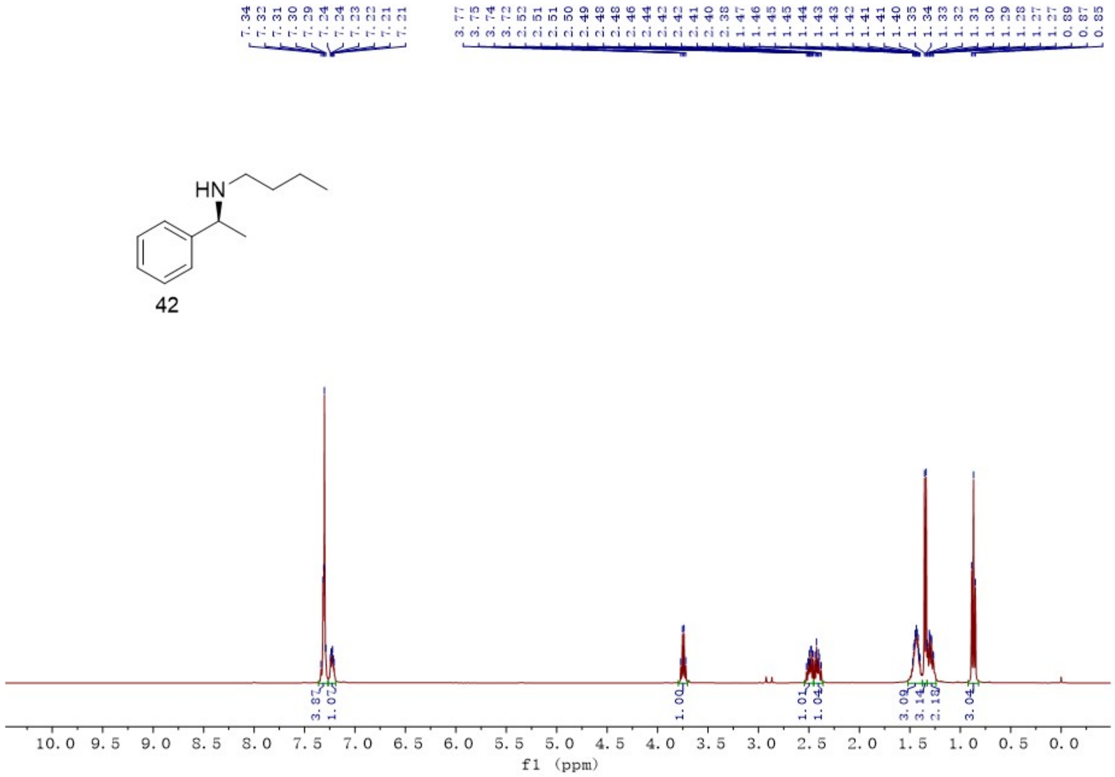

Supplementary Figure 159. ^{1}H NMR spectrum of **42** in $CDCl_3$.

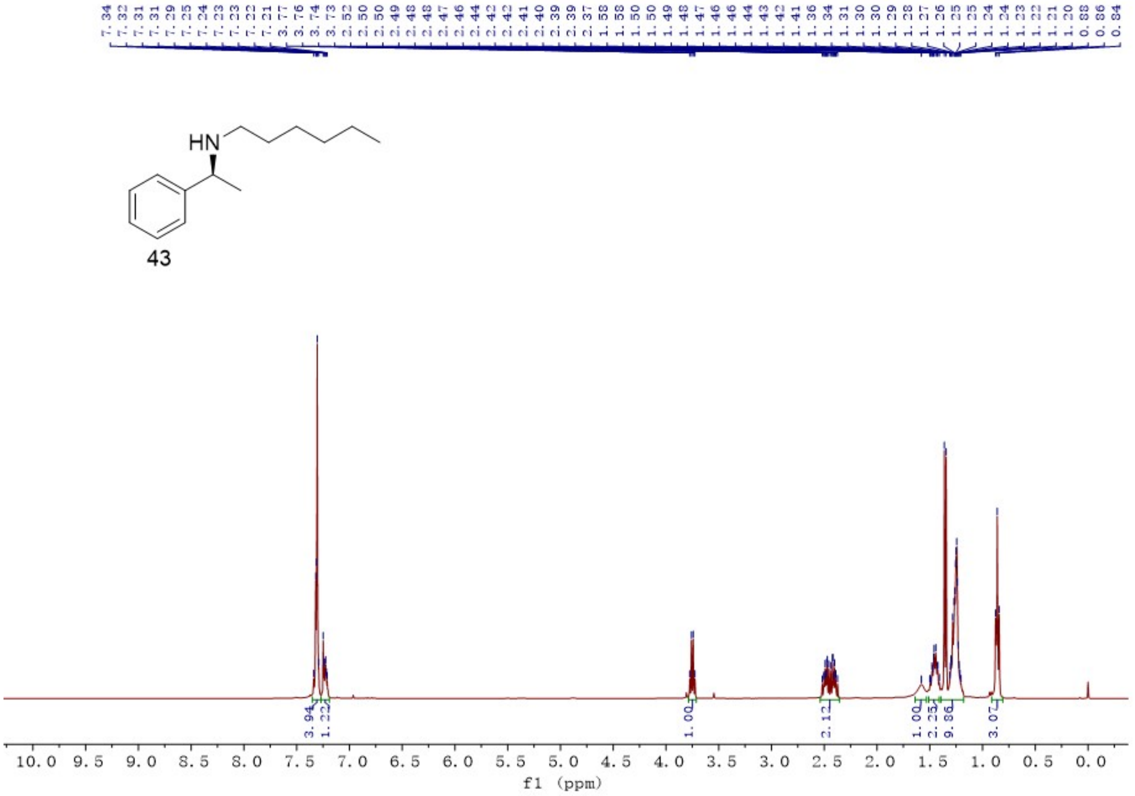

Supplementary Figure 160. ^{1}H NMR spectrum of **43** in $CDCl_3$.

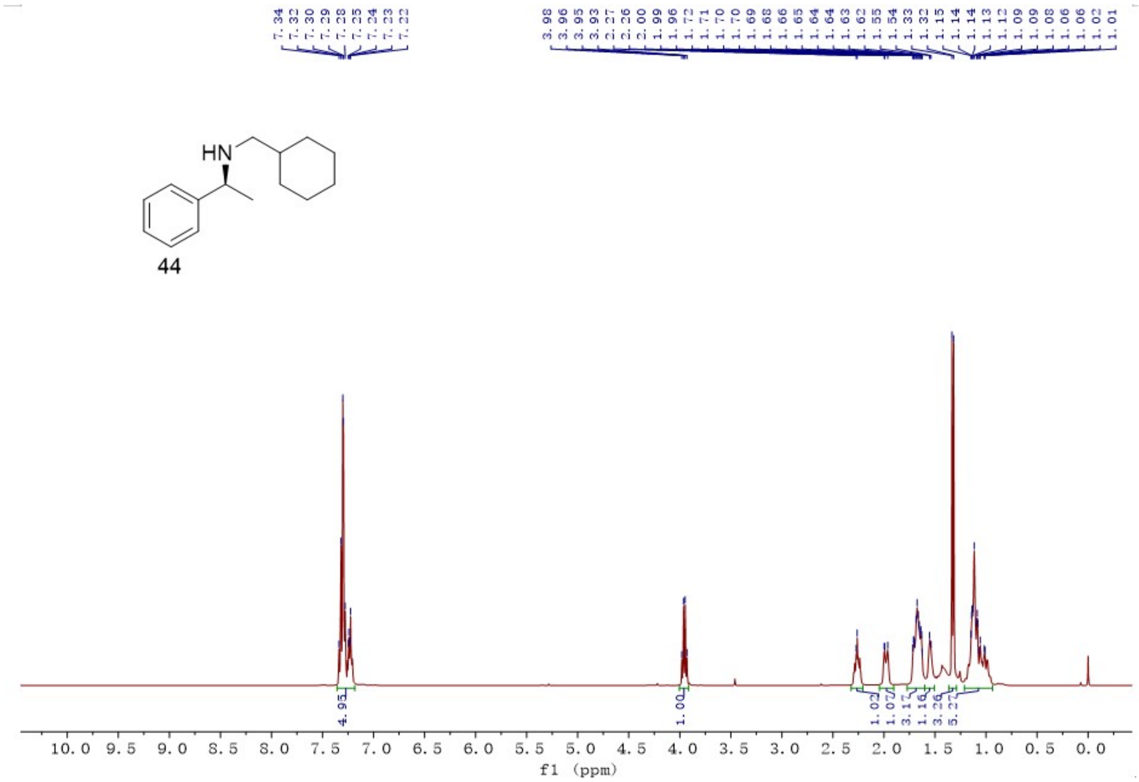

Supplementary Figure 161. ^{1}H NMR spectrum of **44** in $CDCl_3$.

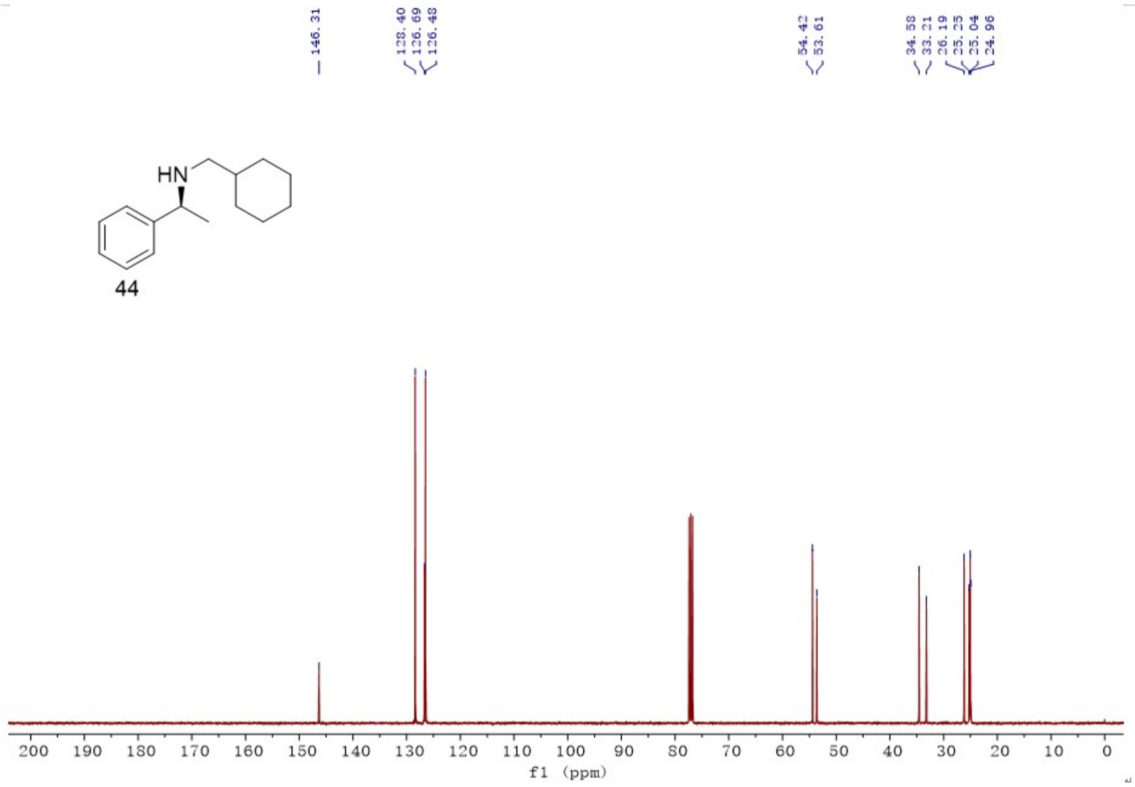

Supplementary Figure 162. ^{13}C NMR spectrum of **44** in $CDCl_3$.

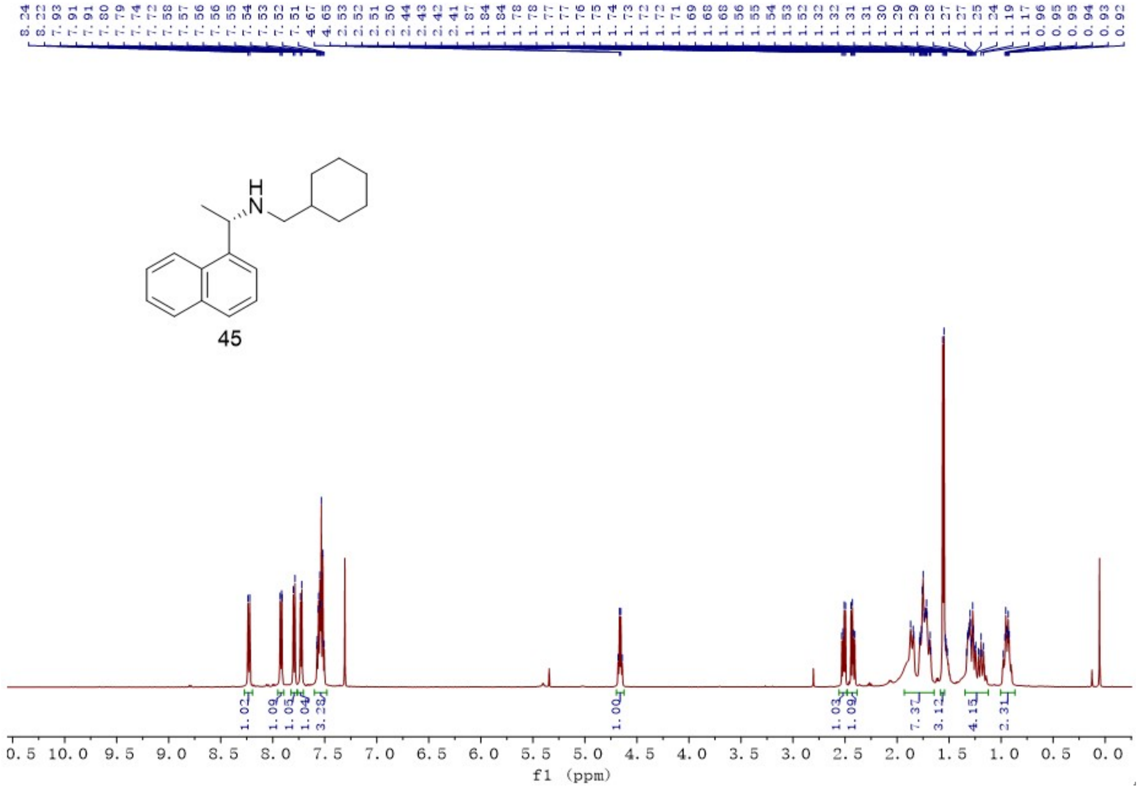

Supplementary Figure 163. ^{1}H NMR spectrum of **45** in $CDCl_3$.

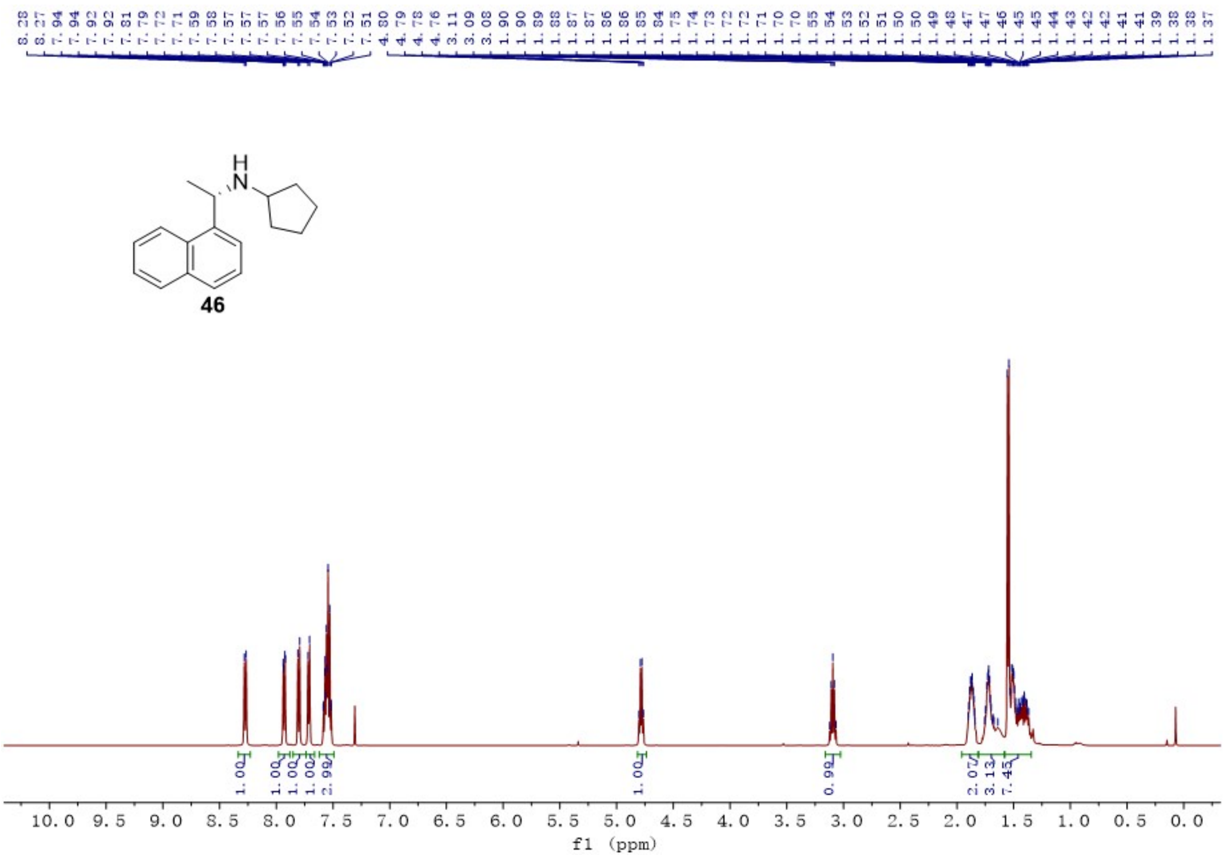

Supplementary Figure 164. ^{1}H NMR spectrum of **46** in $CDCl_3$.

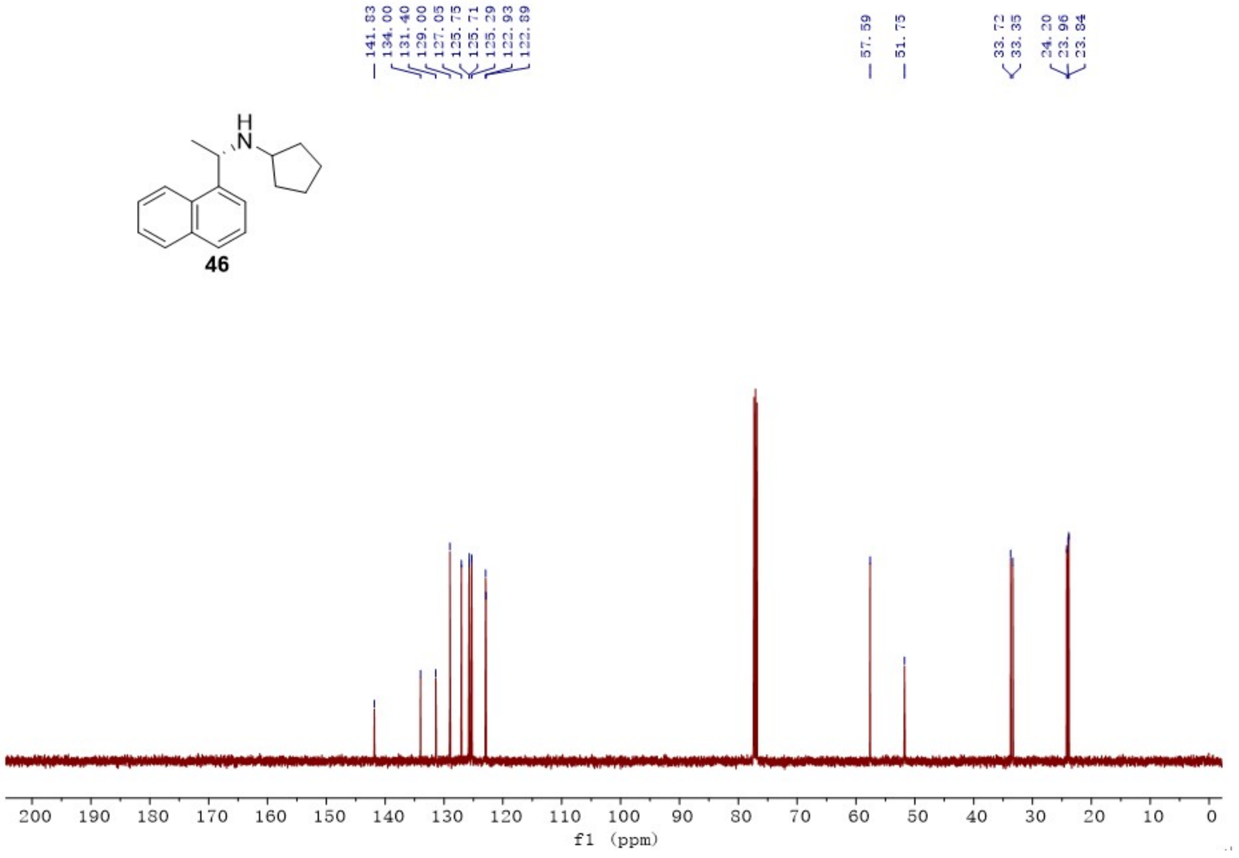

Supplementary Figure 165. ^{13}C NMR spectrum of **46** in $CDCl_3$.

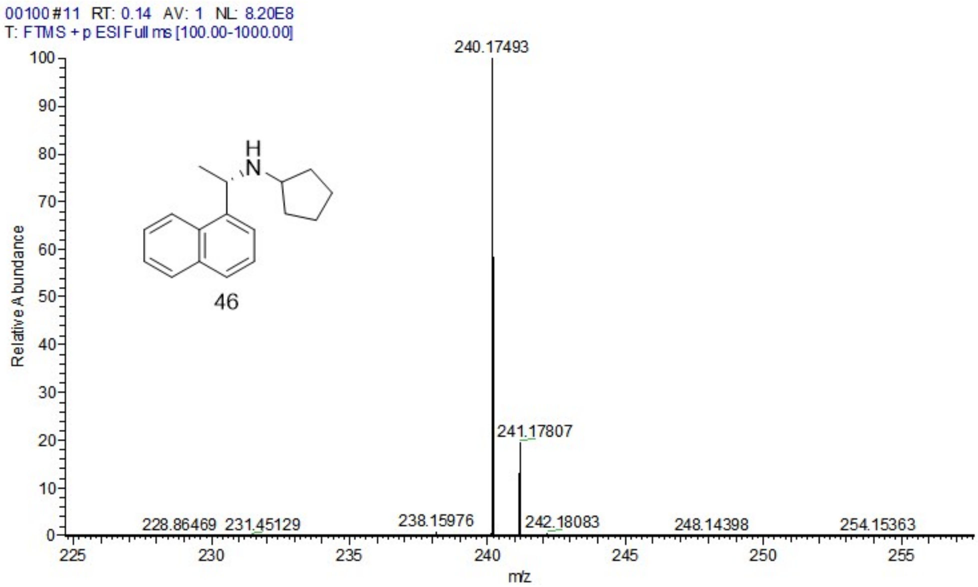

Supplementary Figure 166. HRMS of **46**.

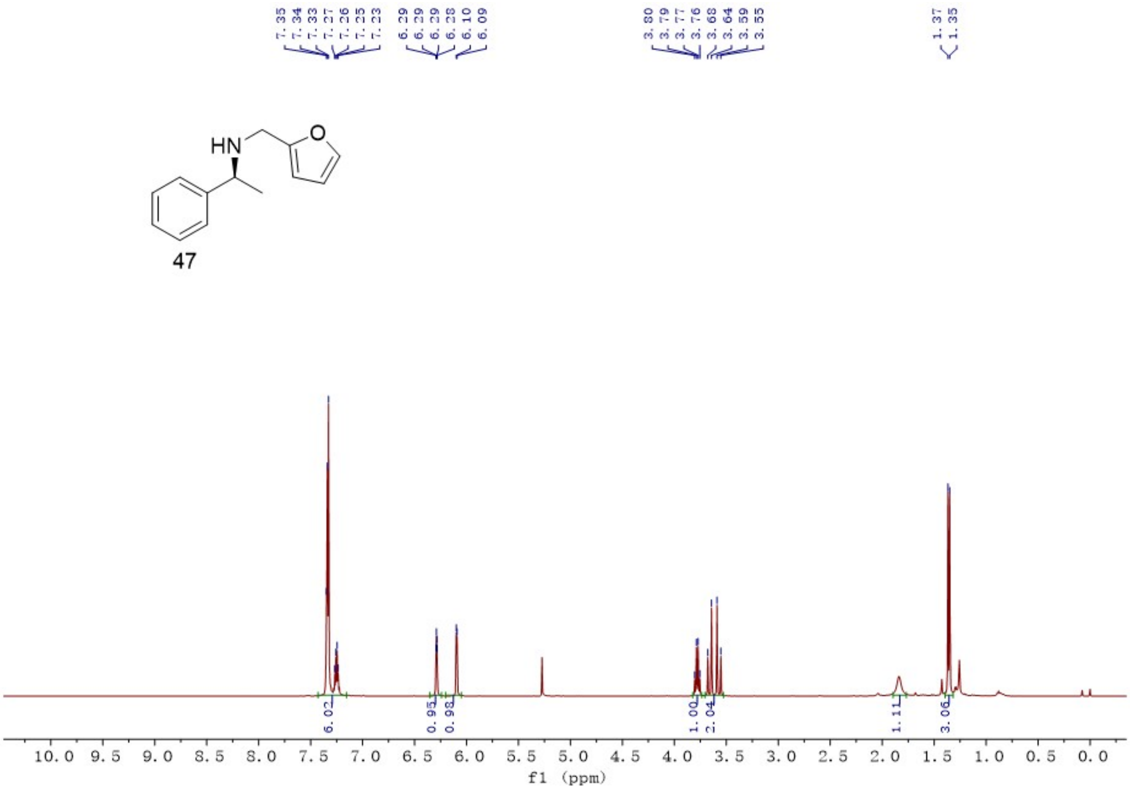

Supplementary Figure 167. ^{1}H NMR spectrum of **47** in $CDCl_3$.

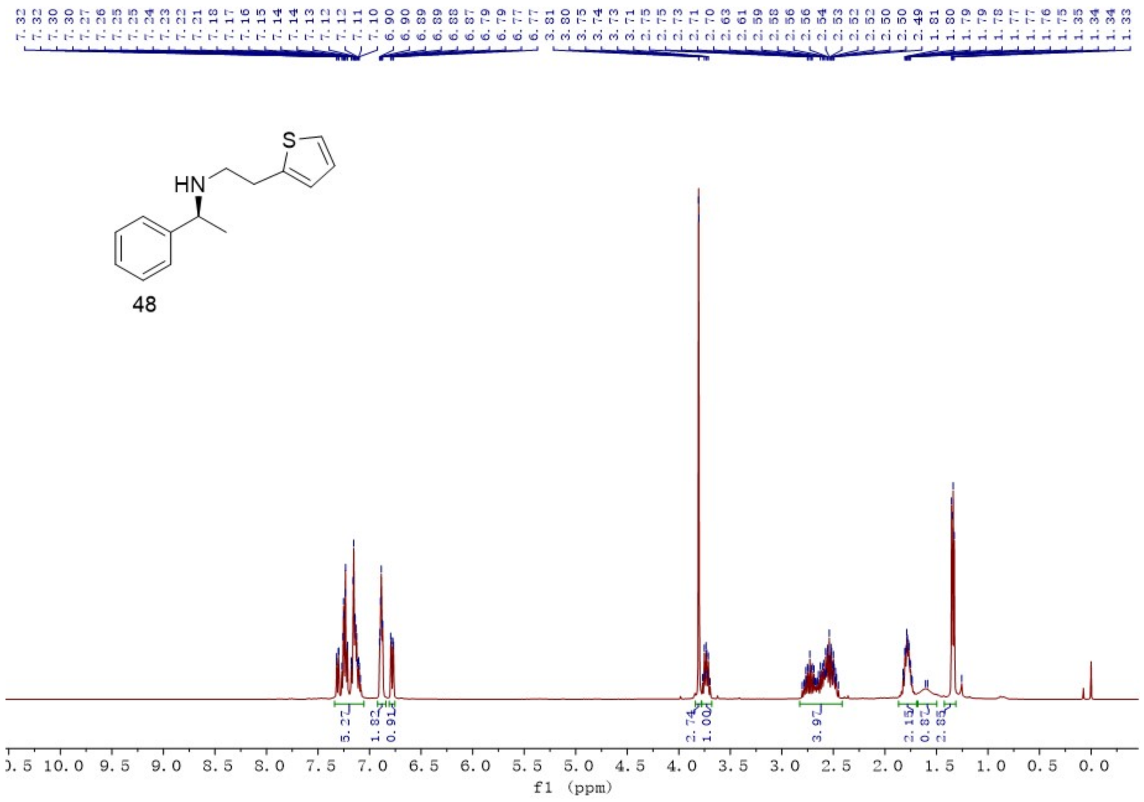

Supplementary Figure 168. ^{1}H NMR spectrum of **48** in $CDCl_3$.

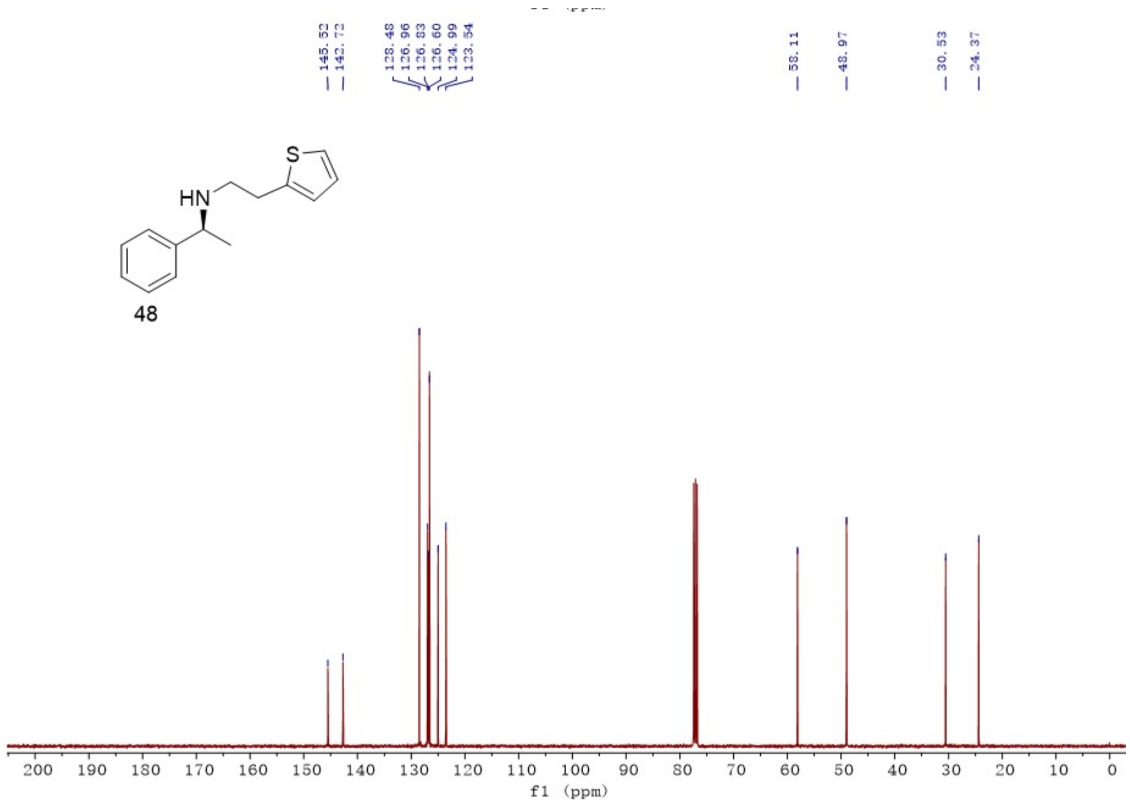

Supplementary Figure 169. ^{13}C NMR spectrum of **48** in $CDCl_3$.

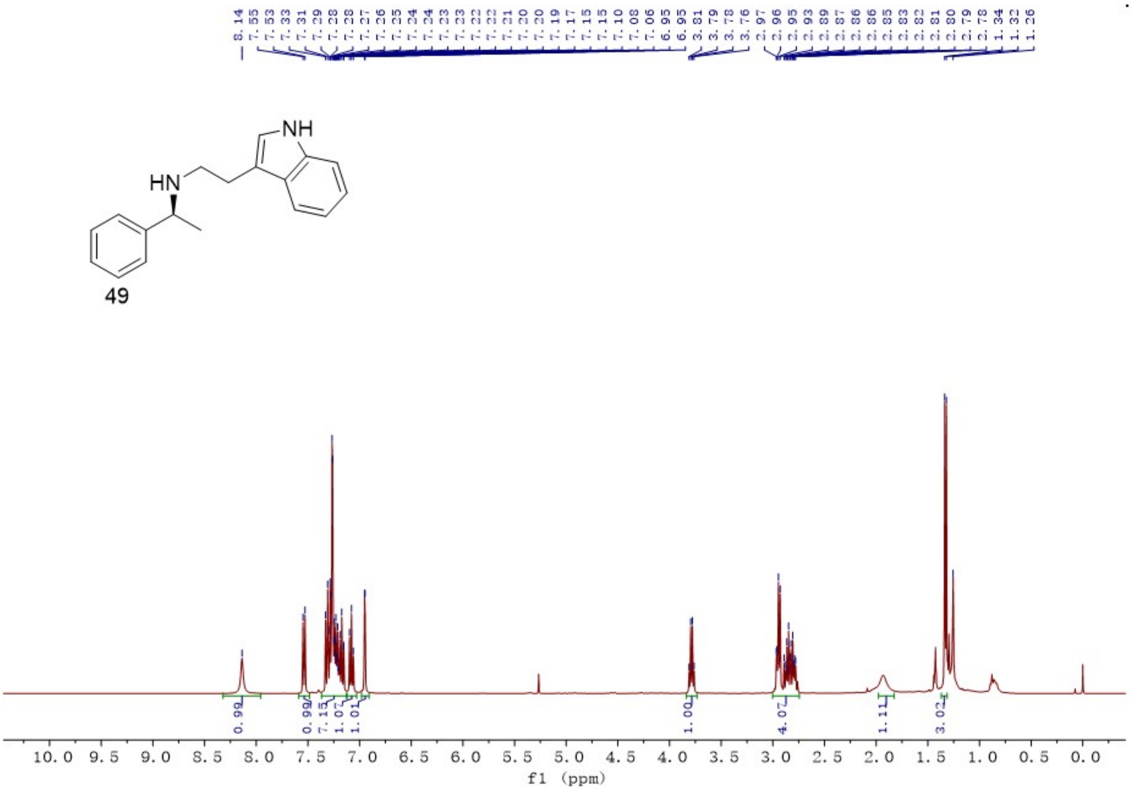

Supplementary Figure 170. ^{1}H NMR spectrum of **49** in $CDCl_3$.

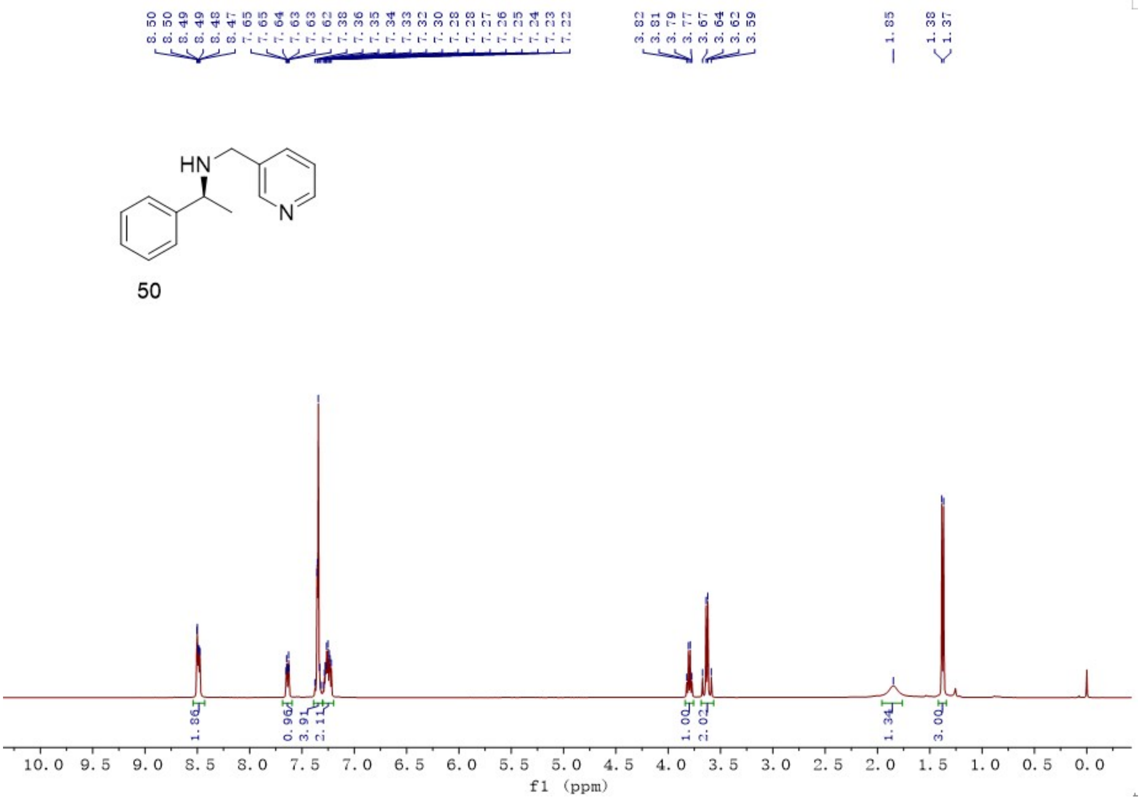

Supplementary Figure 171. ^{1}H NMR spectrum of **50** in $CDCl_3$.

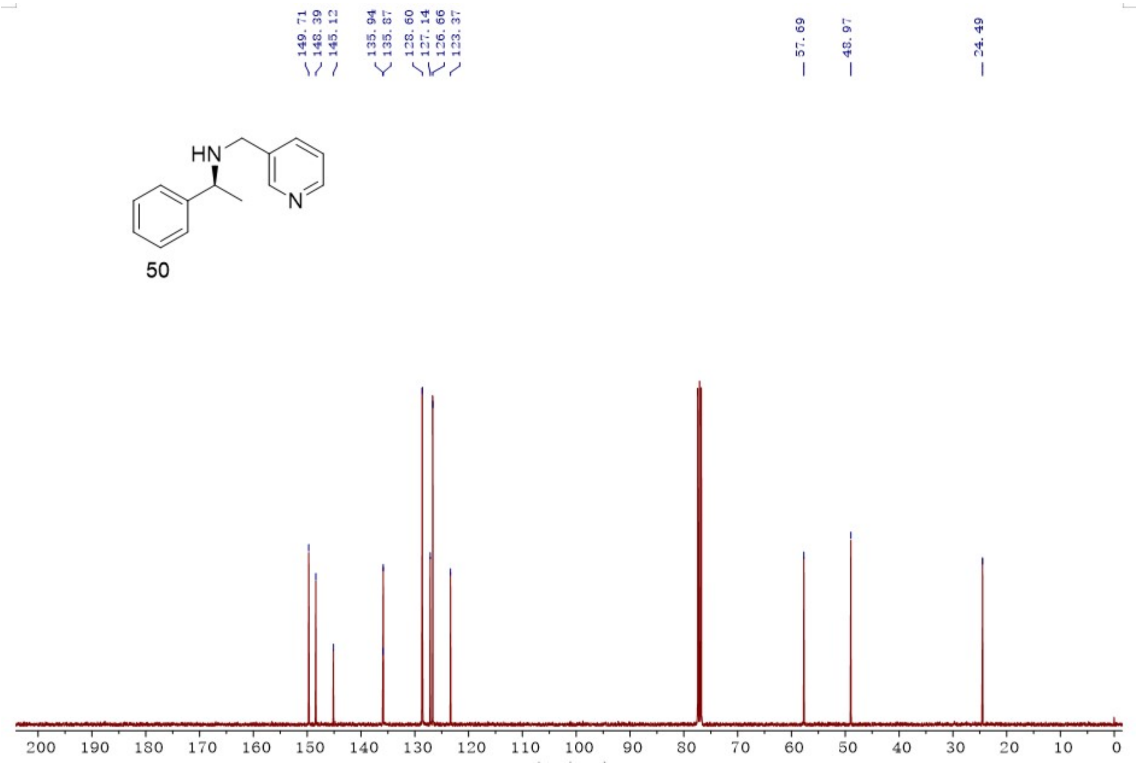

Supplementary Figure 172. ^{13}C NMR spectrum of **50** in $CDCl_3$.

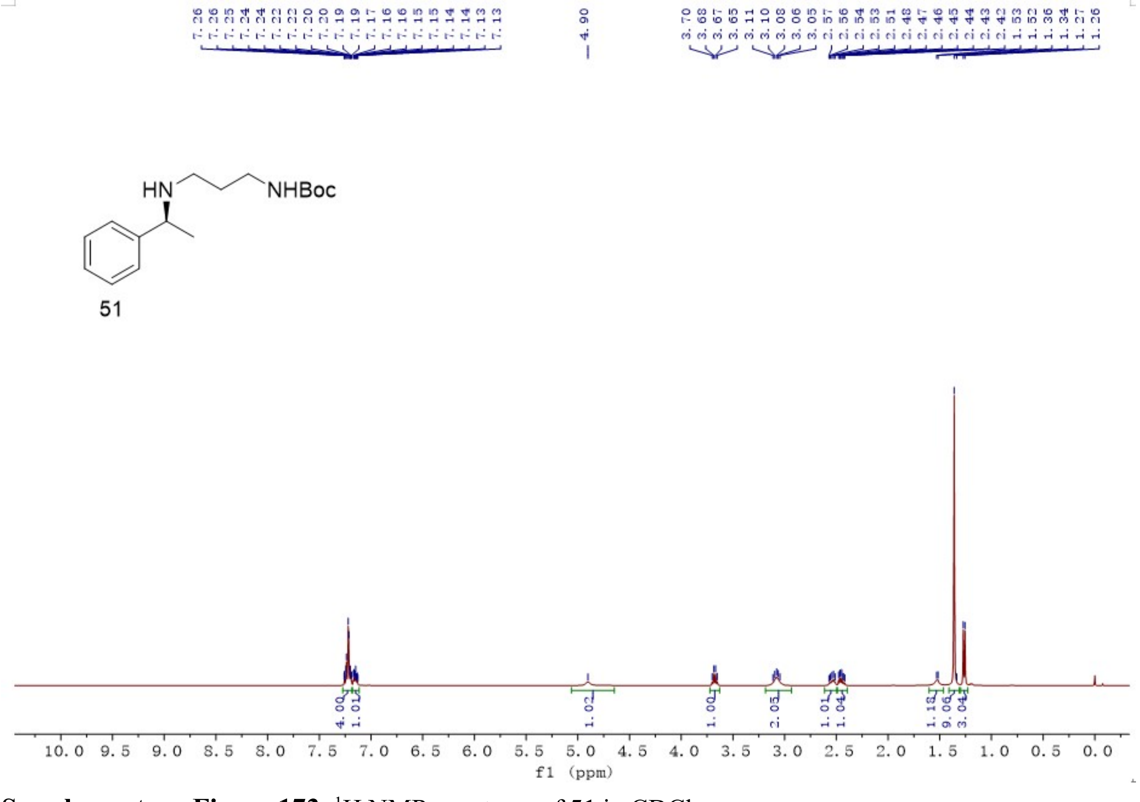

Supplementary Figure 173. ^{1}H NMR spectrum of **51** in $CDCl_3$.

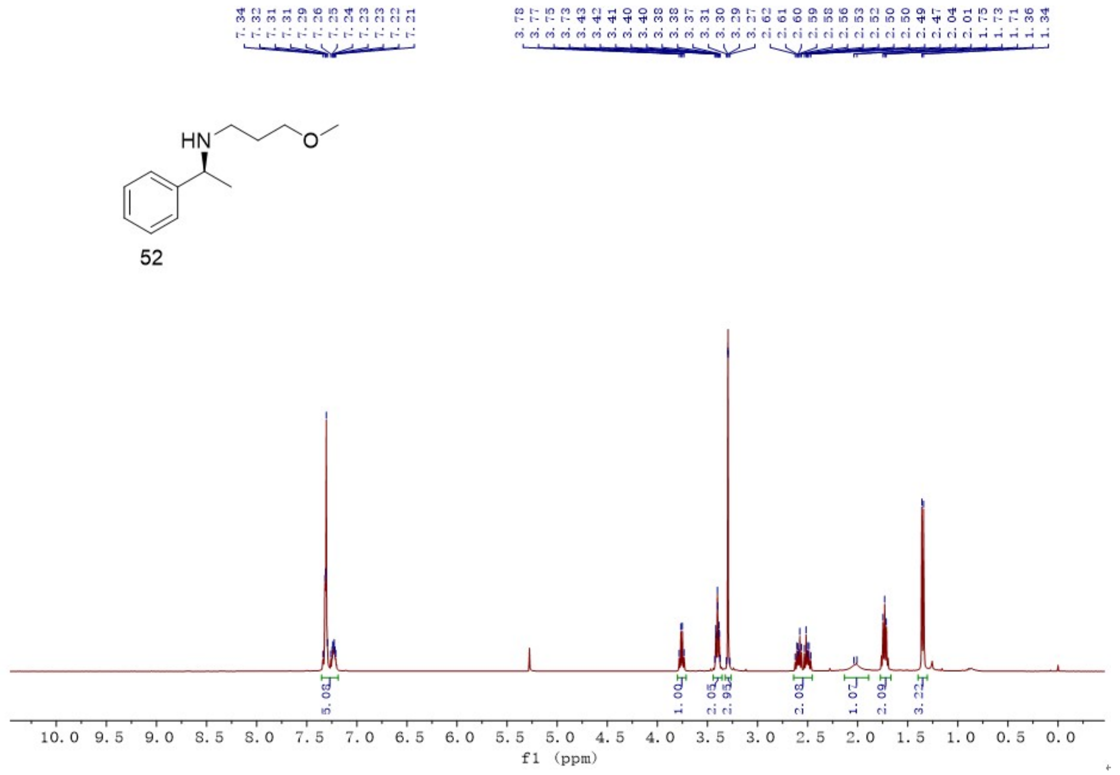

Supplementary Figure 174. ^{1}H NMR spectrum of **52** in $CDCl_3$.

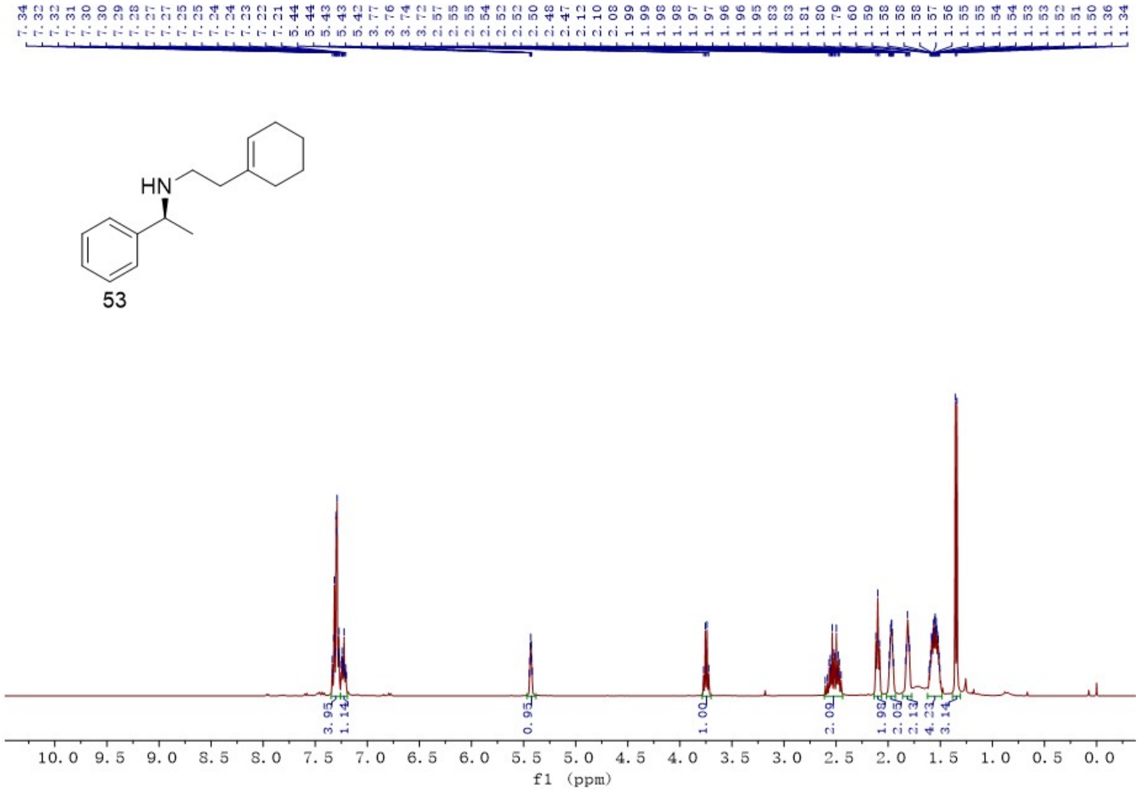

Supplementary Figure 175. ^{1}H NMR spectrum of **53** in $CDCl_3$.

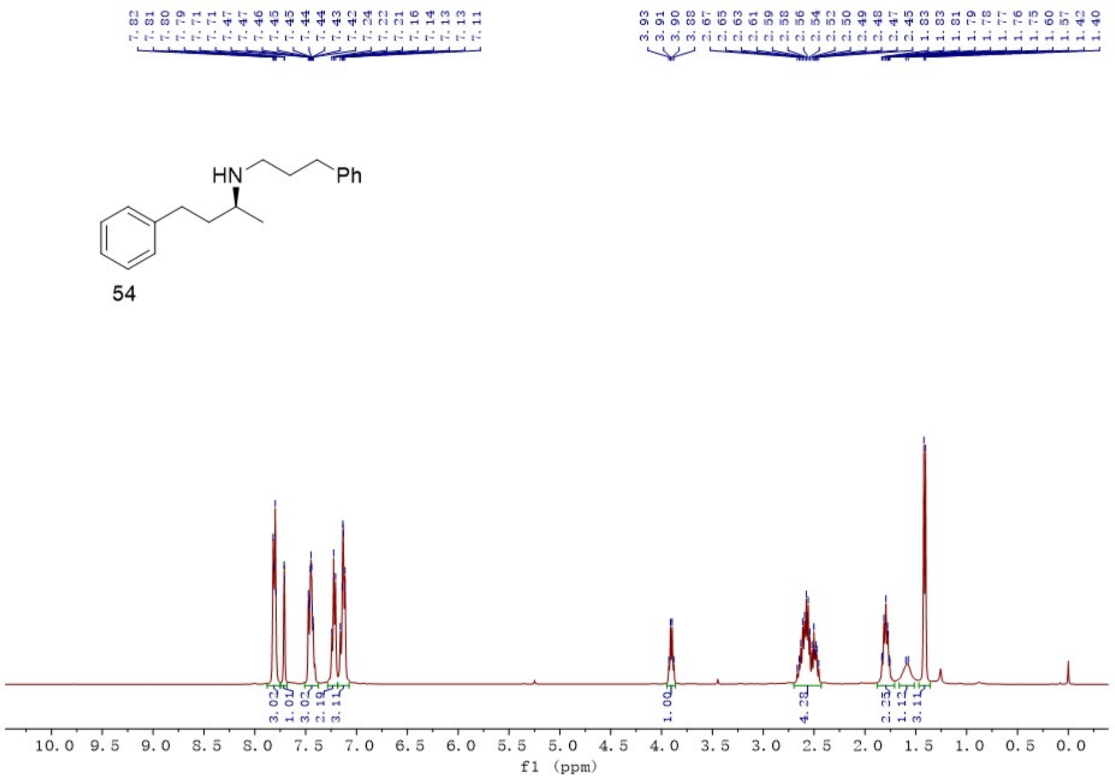

Supplementary Figure 176. ^{1}H NMR spectrum of **54** in $CDCl_3$.

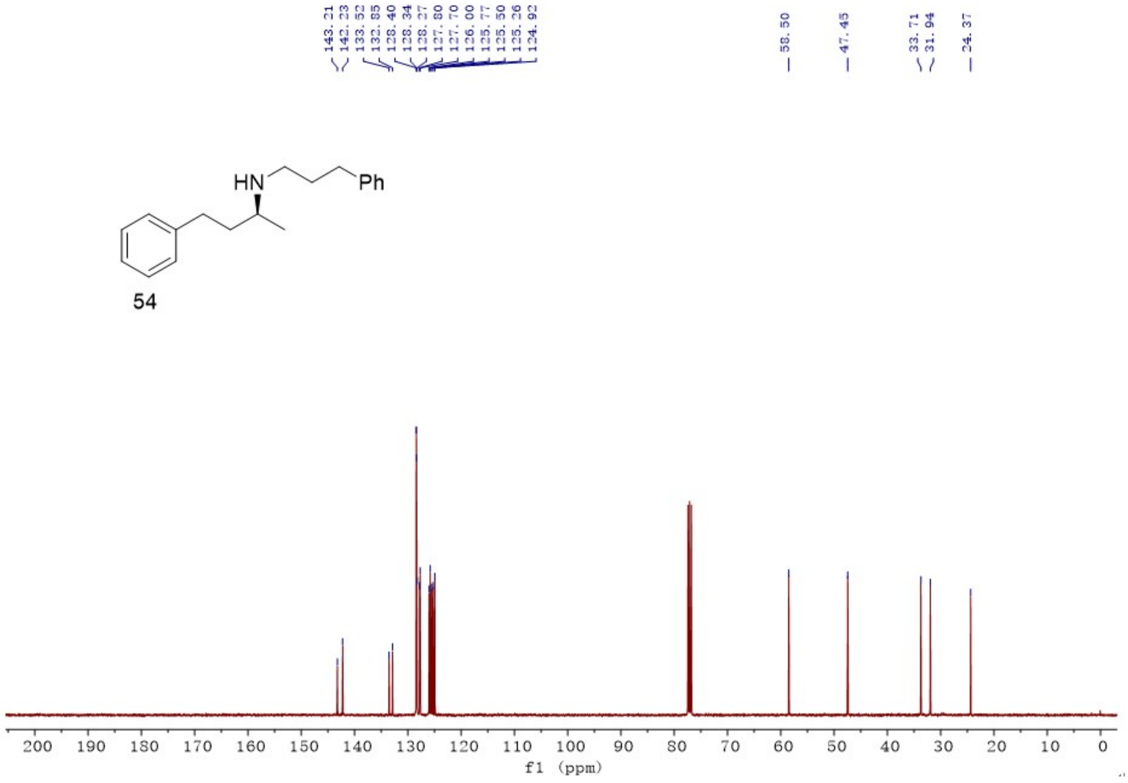

Supplementary Figure 177. ^{13}C NMR spectrum of **54** in $CDCl_3$.

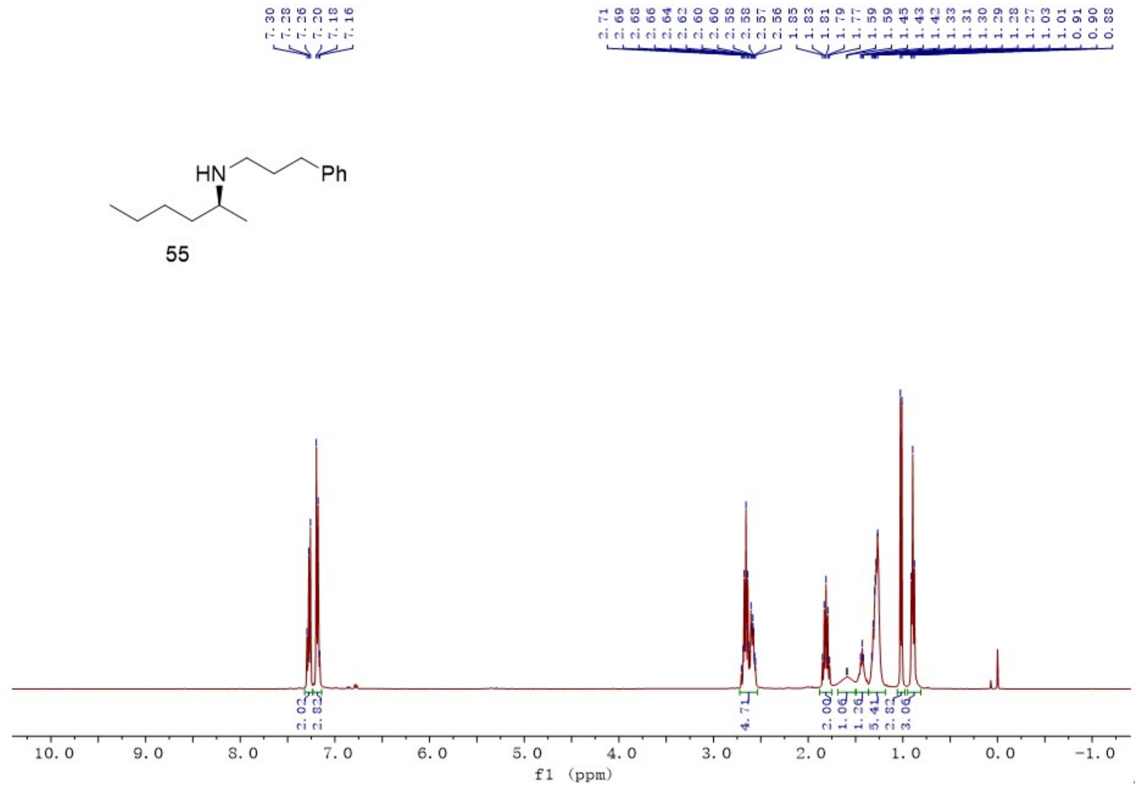

Supplementary Figure 178. ^{1}H NMR spectrum of **55** in $CDCl_3$.

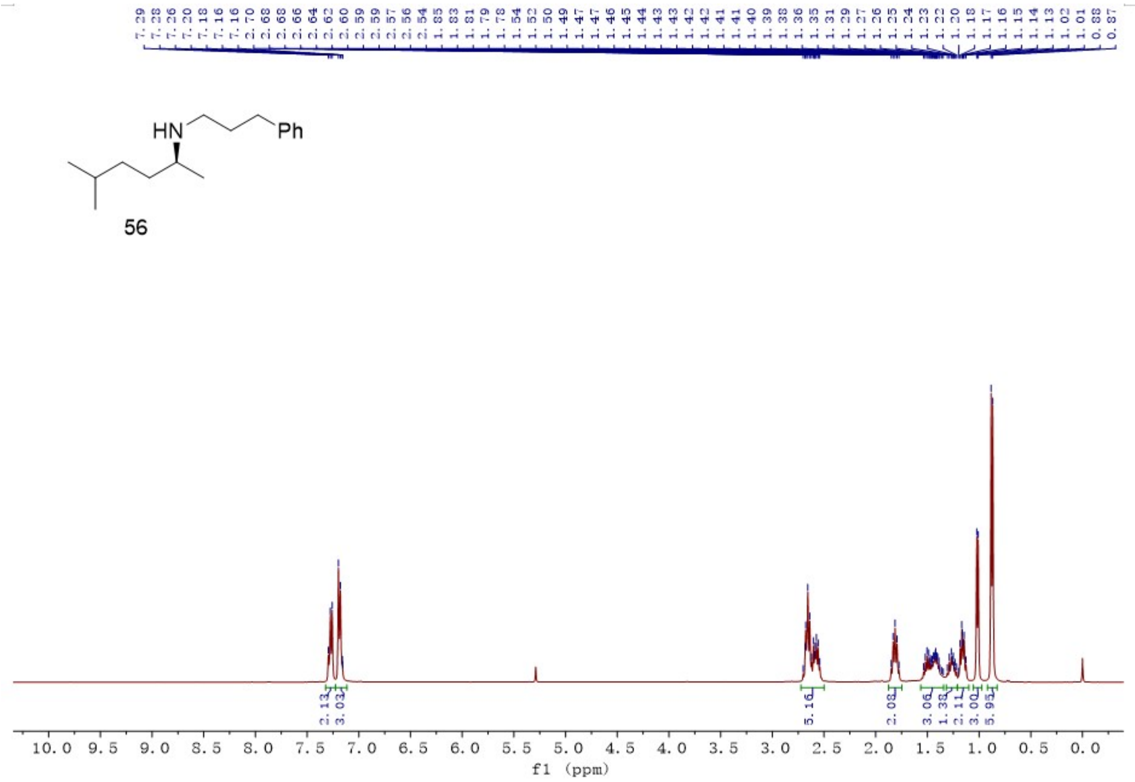

Supplementary Figure 179. ^{1}H NMR spectrum of **56** in $CDCl_3$.

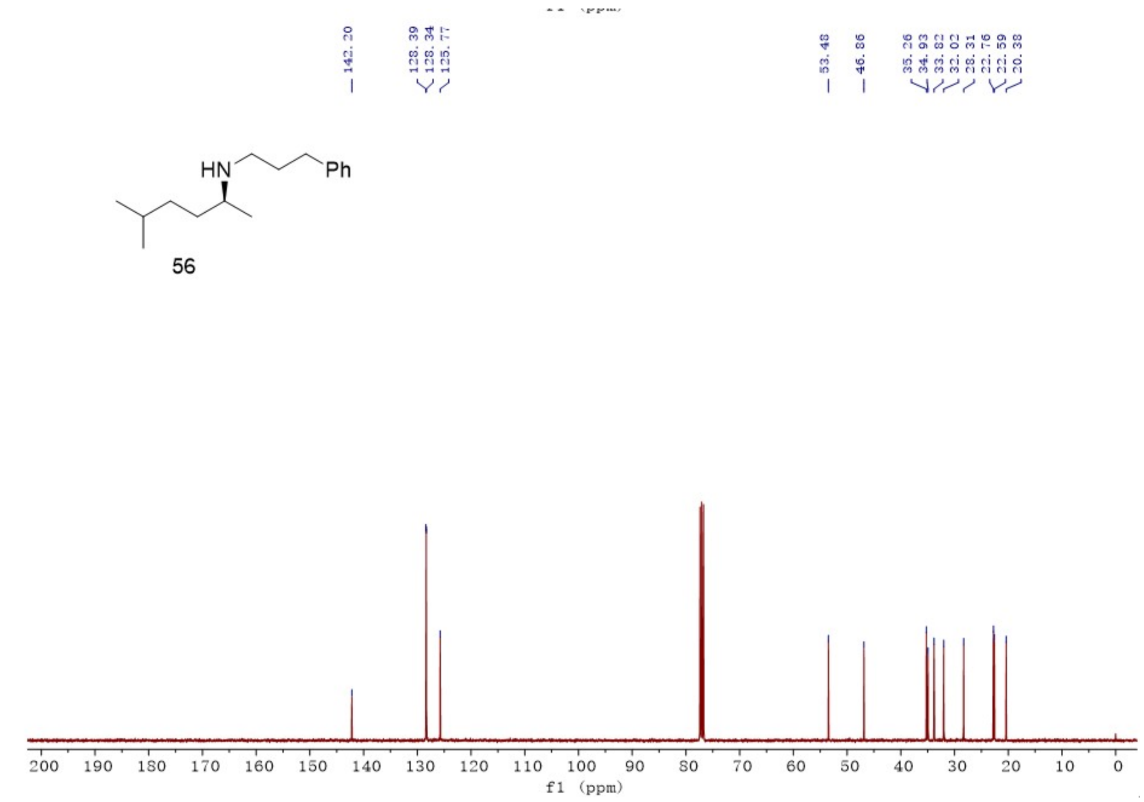

Supplementary Figure 180. ^{13}C NMR spectrum of **56** in $CDCl_3$.

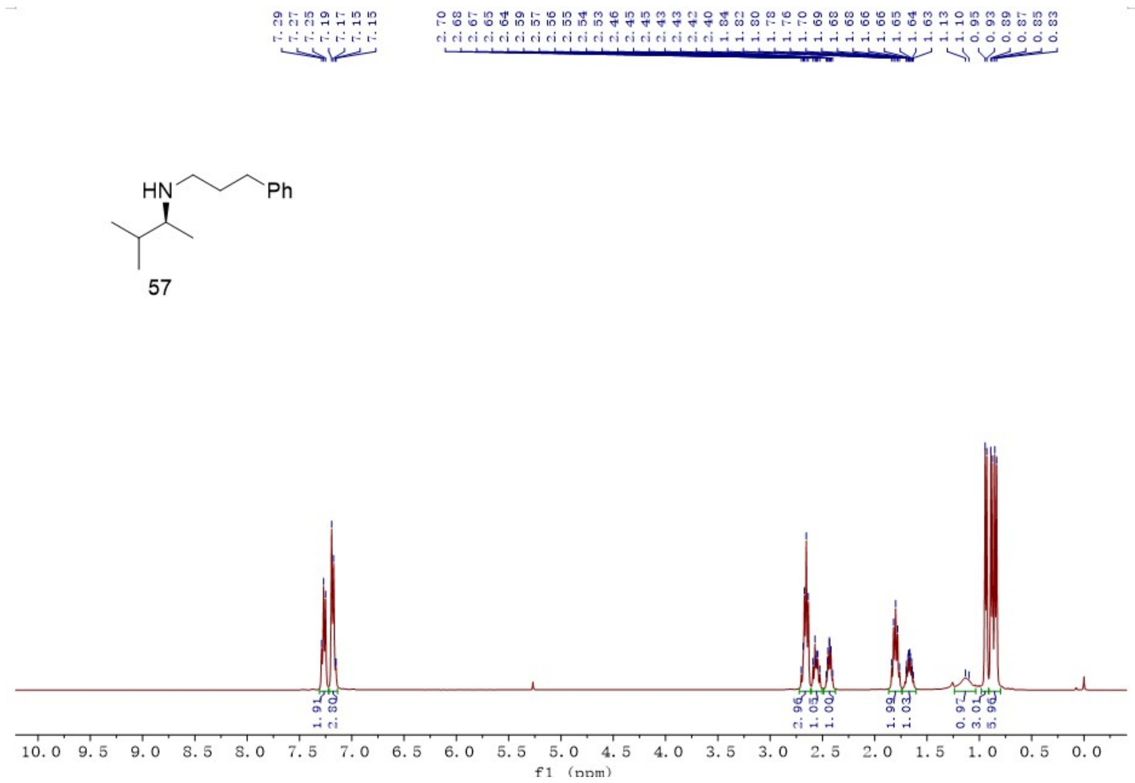

Supplementary Figure 181. ^{1}H NMR spectrum of **57** in $CDCl_3$.

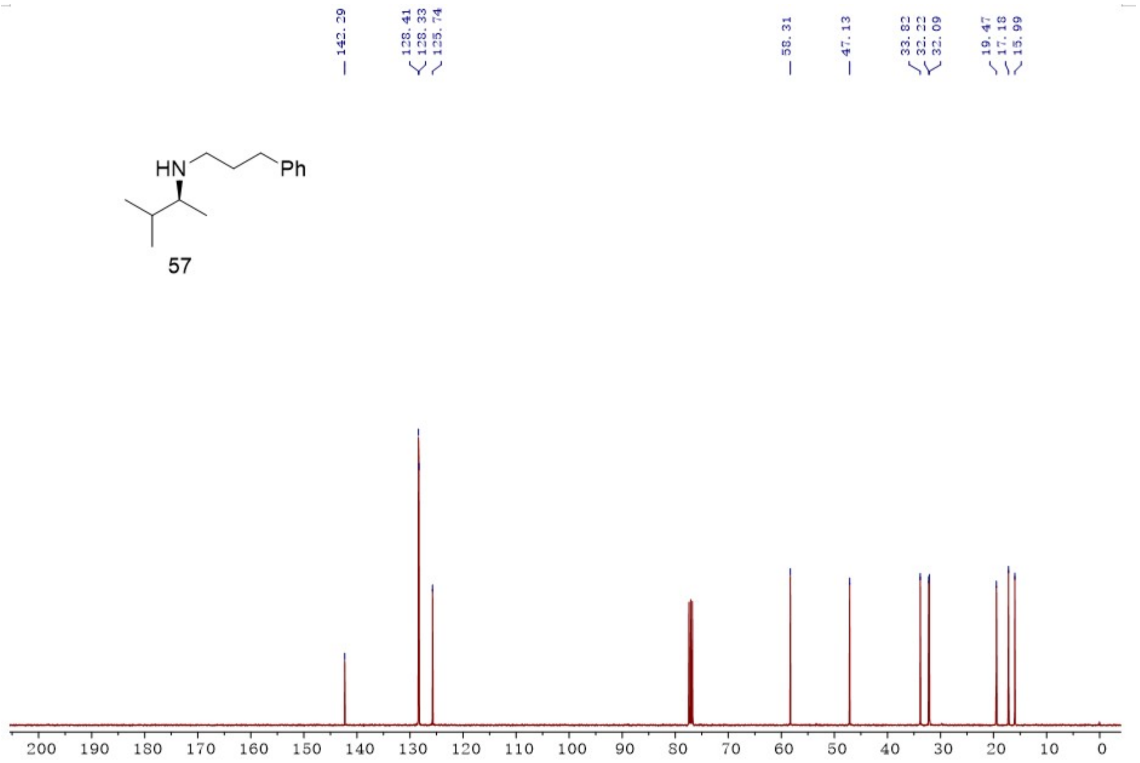

Supplementary Figure 182. ^{13}C NMR spectrum of **57** in $CDCl_3$.

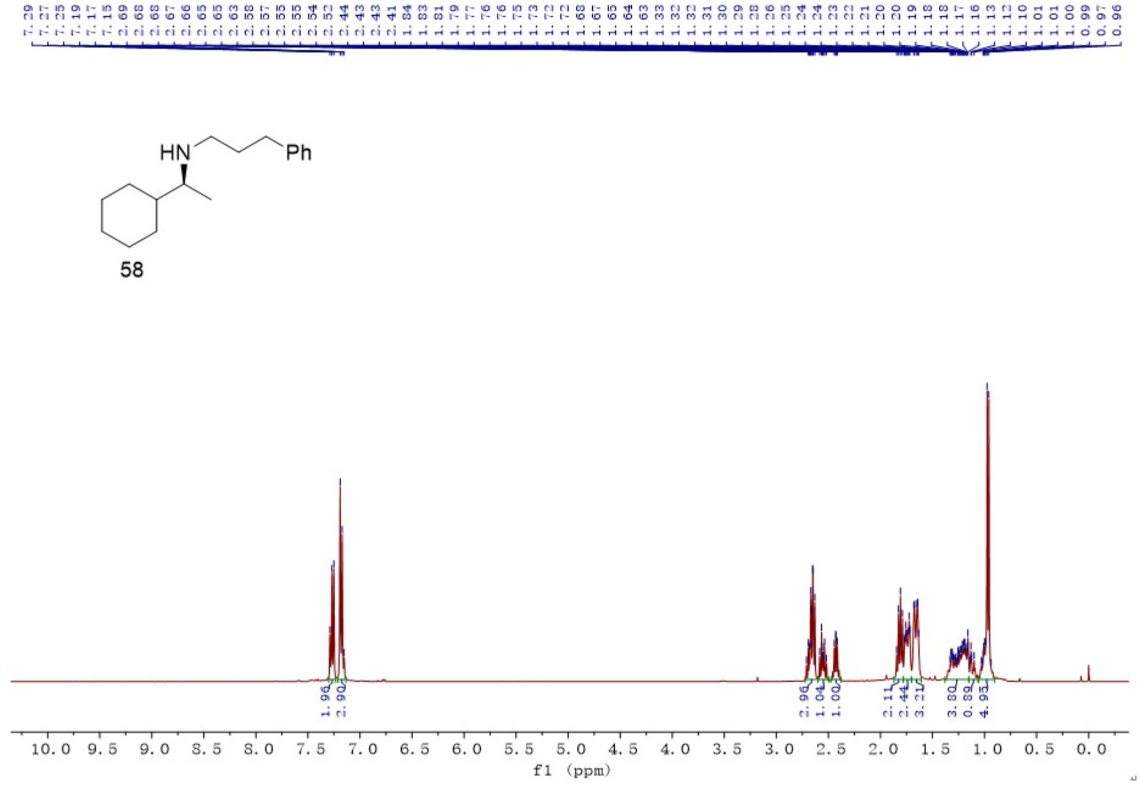

Supplementary Figure 183. ^{1}H NMR spectrum of **58** in $CDCl_3$.

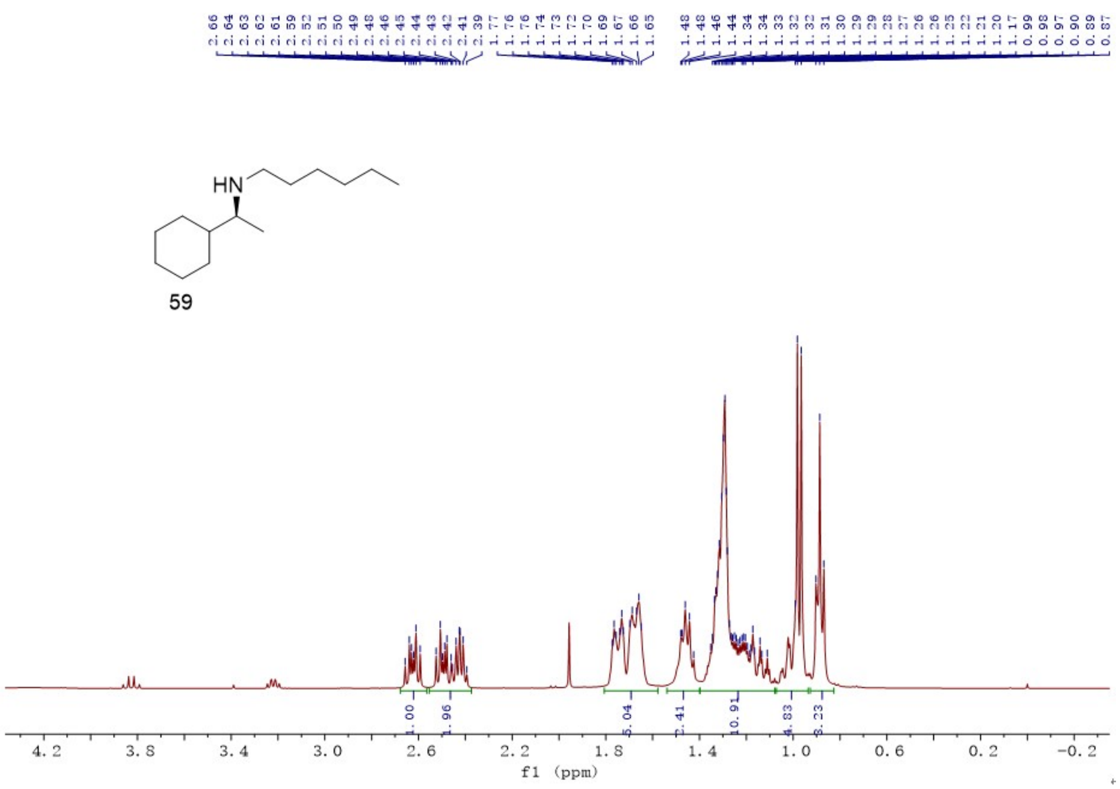

Supplementary Figure 184. ^{1}H NMR spectrum of **45** in $CDCl_3$.

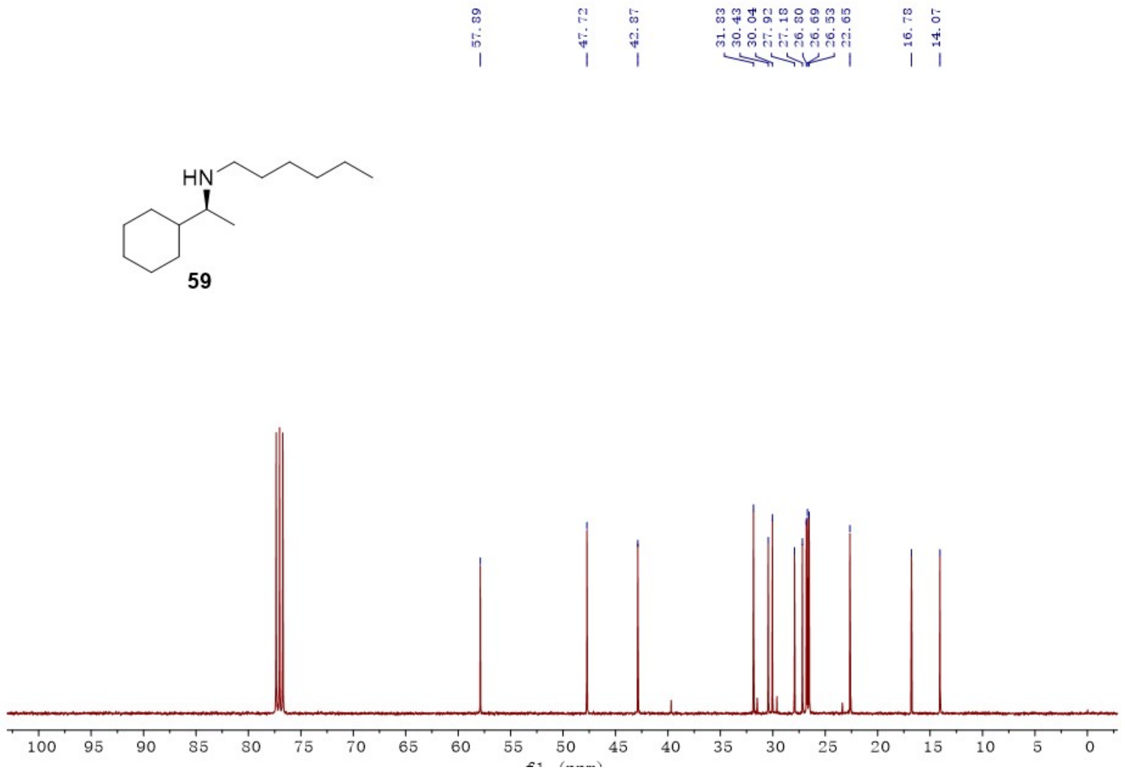

Supplementary Figure 185. ^{13}C NMR spectrum of **59** in $CDCl_3$.

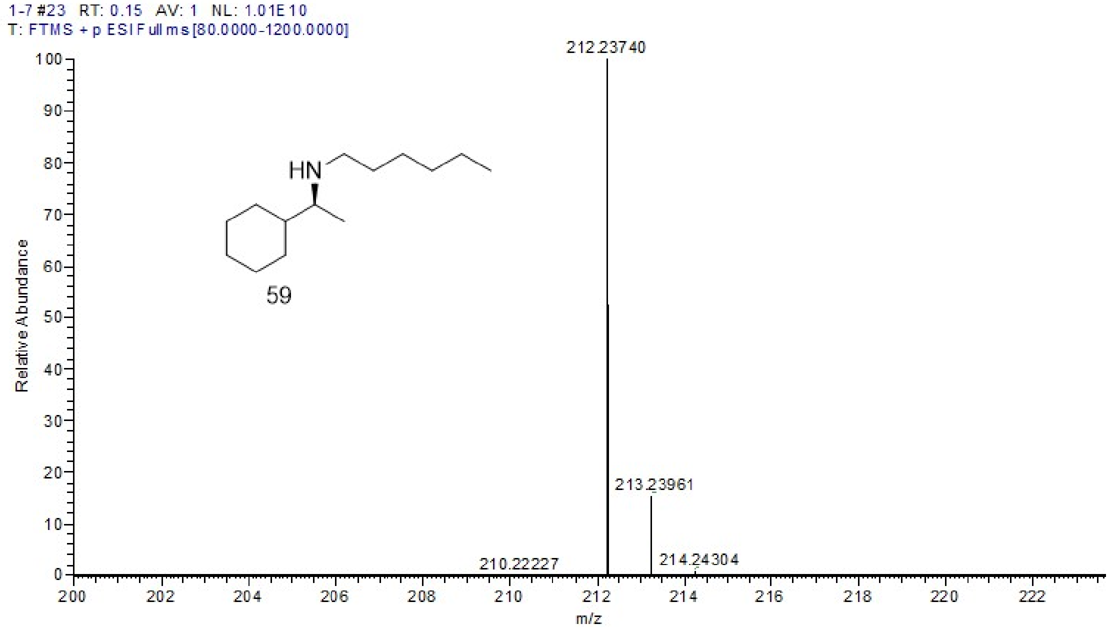

Supplementary Figure 186. HRMS of **59**.

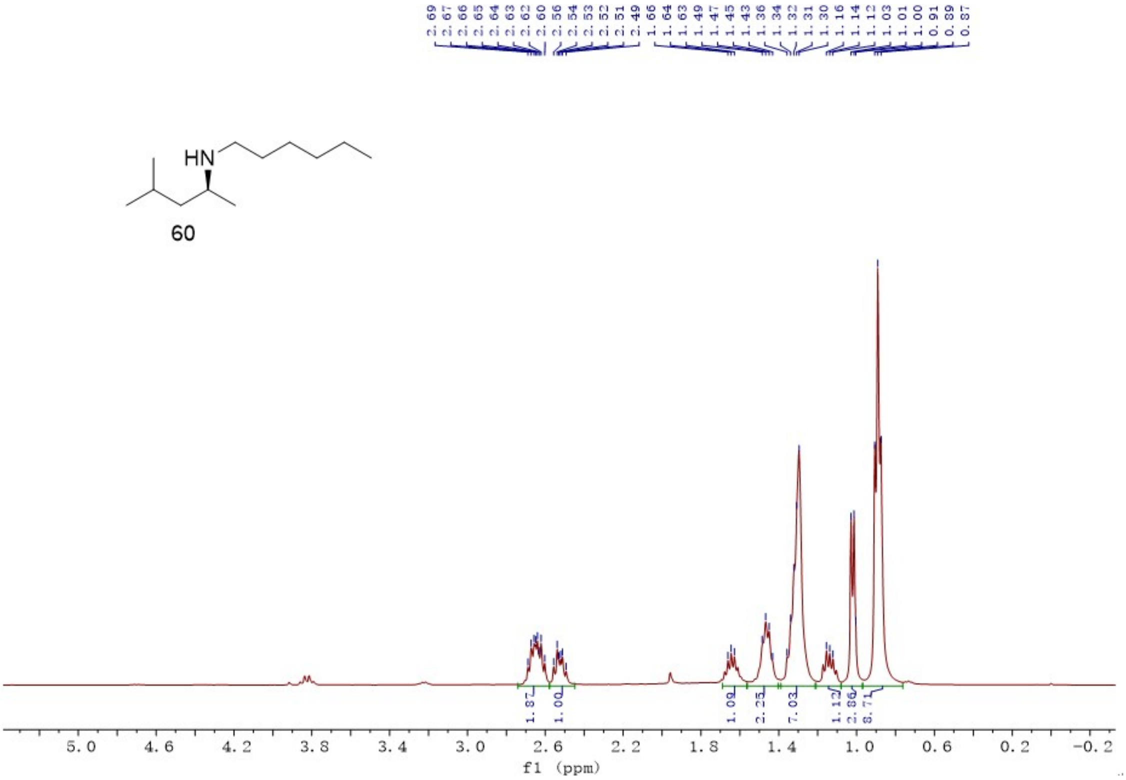

Supplementary Figure 187. ^{1}H NMR spectrum of **60** in $CDCl_3$.

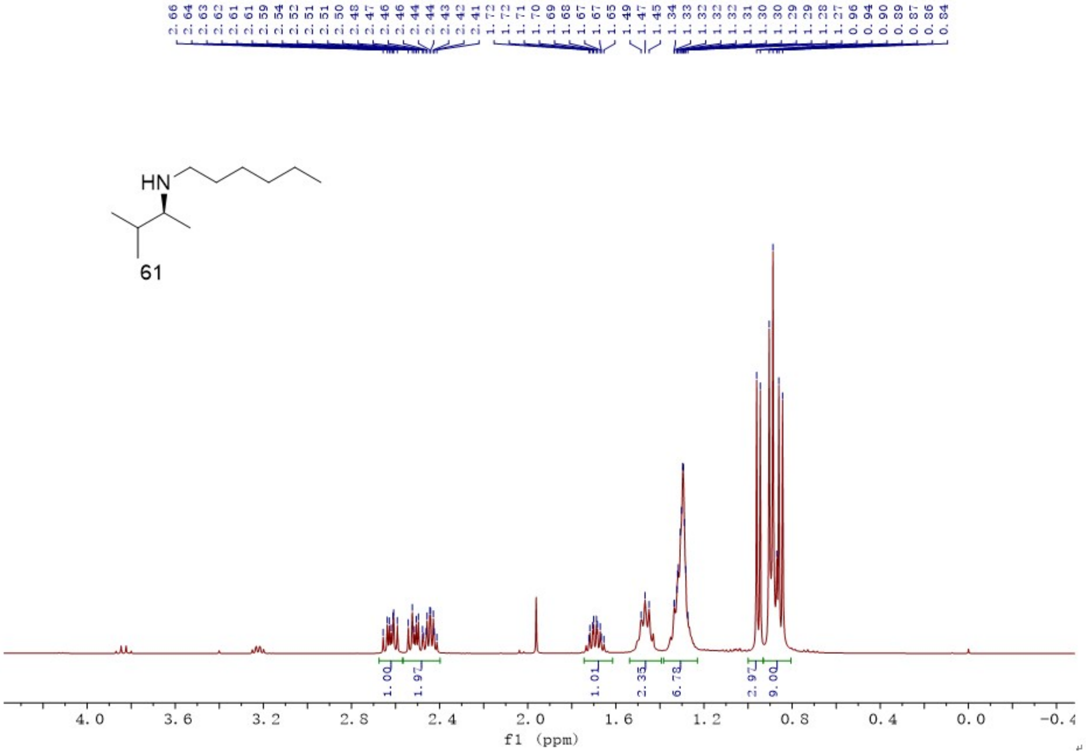

Supplementary Figure 188. ^{1}H NMR spectrum of **61** in $CDCl_3$.

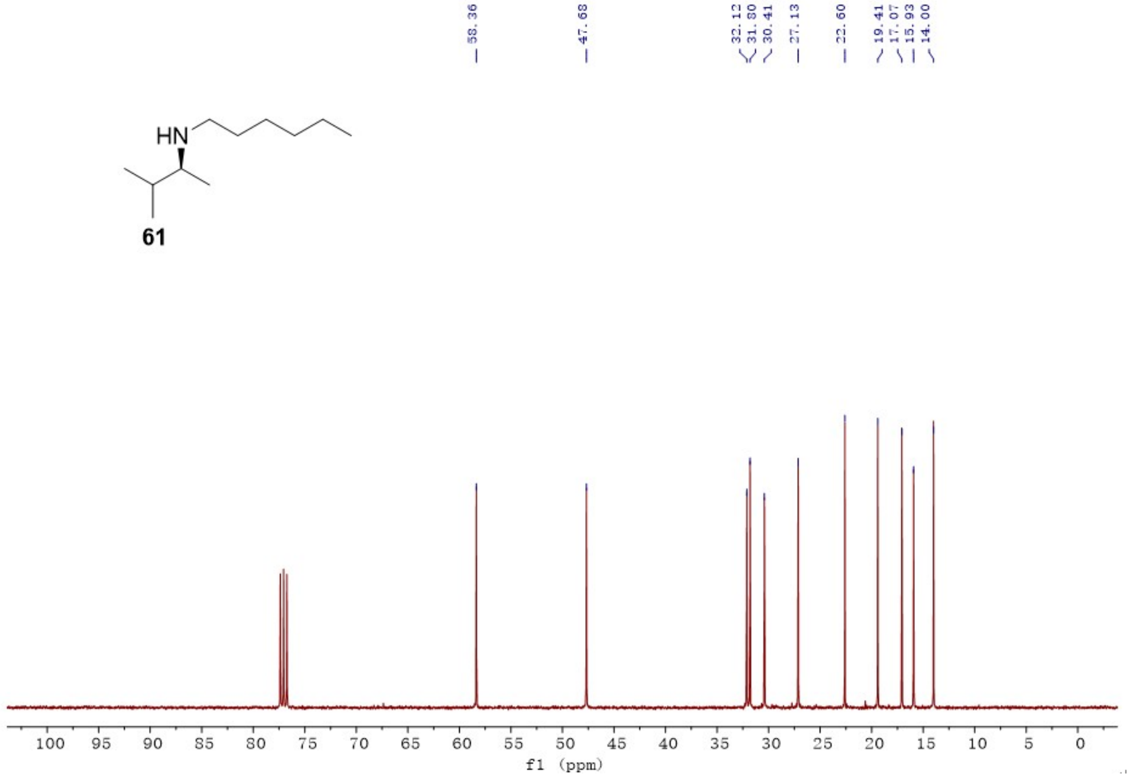

Supplementary Figure 189. ^{13}C NMR spectrum of **61** in $CDCl_3$.

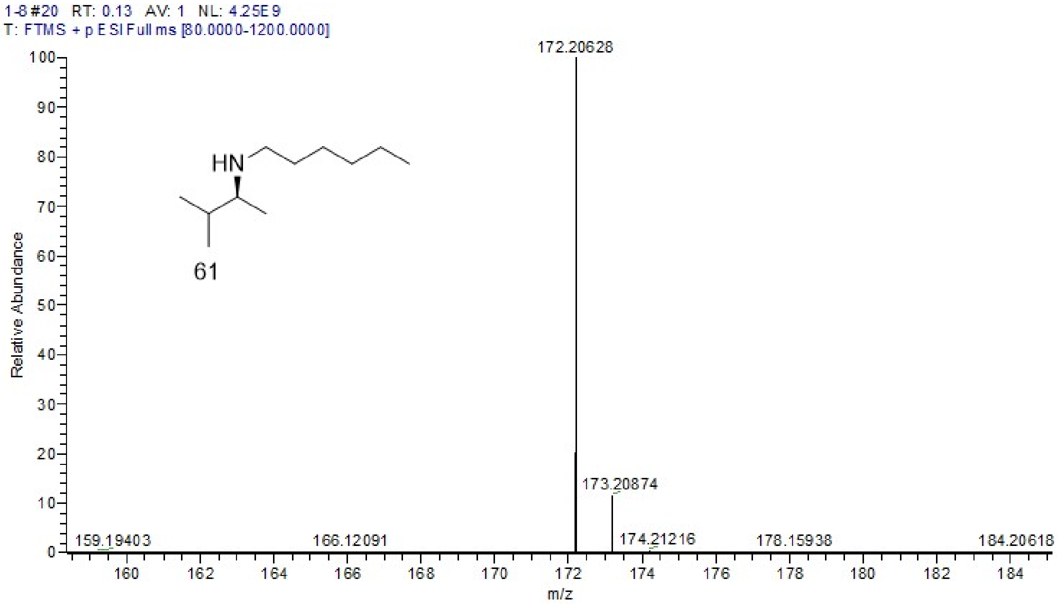

Supplementary Figure 190. ^{1}H NMR spectrum of **61** in $CDCl_3$.

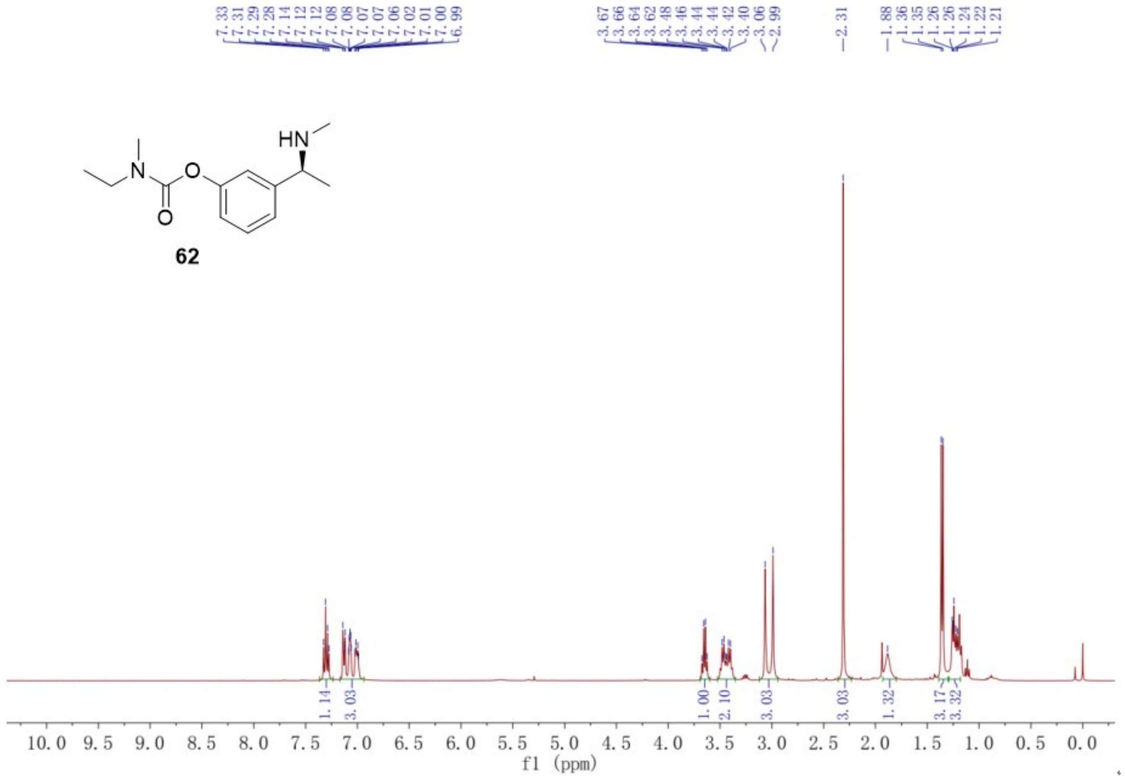

Supplementary Figure 191. ^{1}H NMR spectrum of **62** in $CDCl_3$.

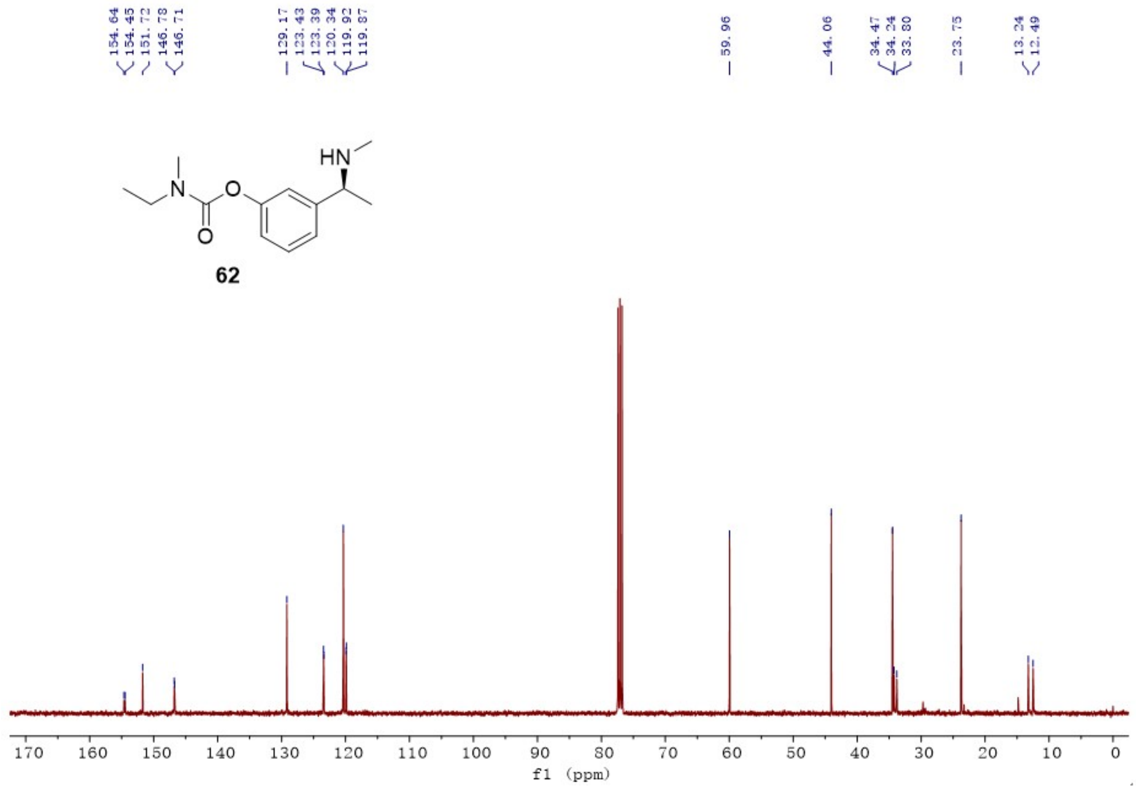

Supplementary Figure 192. ^{13}C NMR spectrum of **62** in $CDCl_3$.

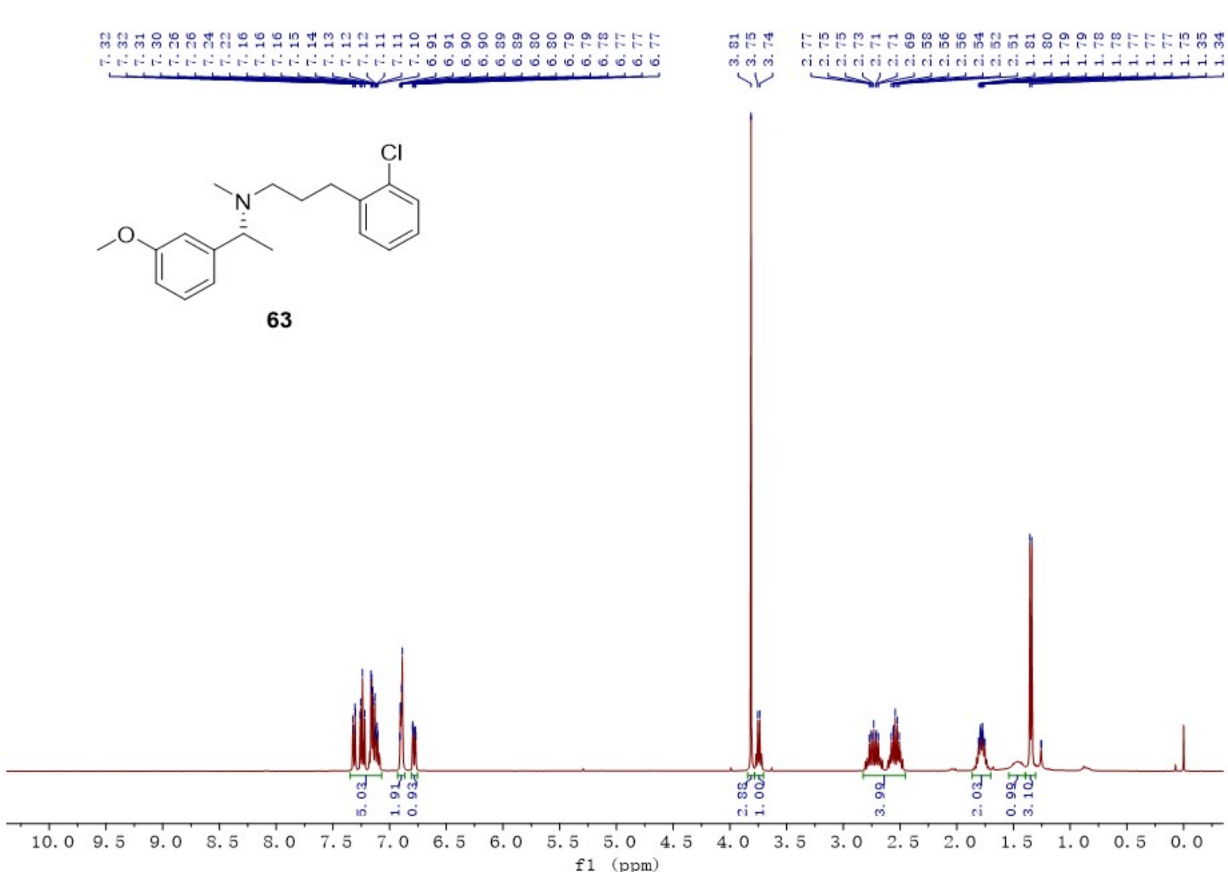

Supplementary Figure 193. ^{1}H NMR spectrum of **63** in $CDCl_3$.

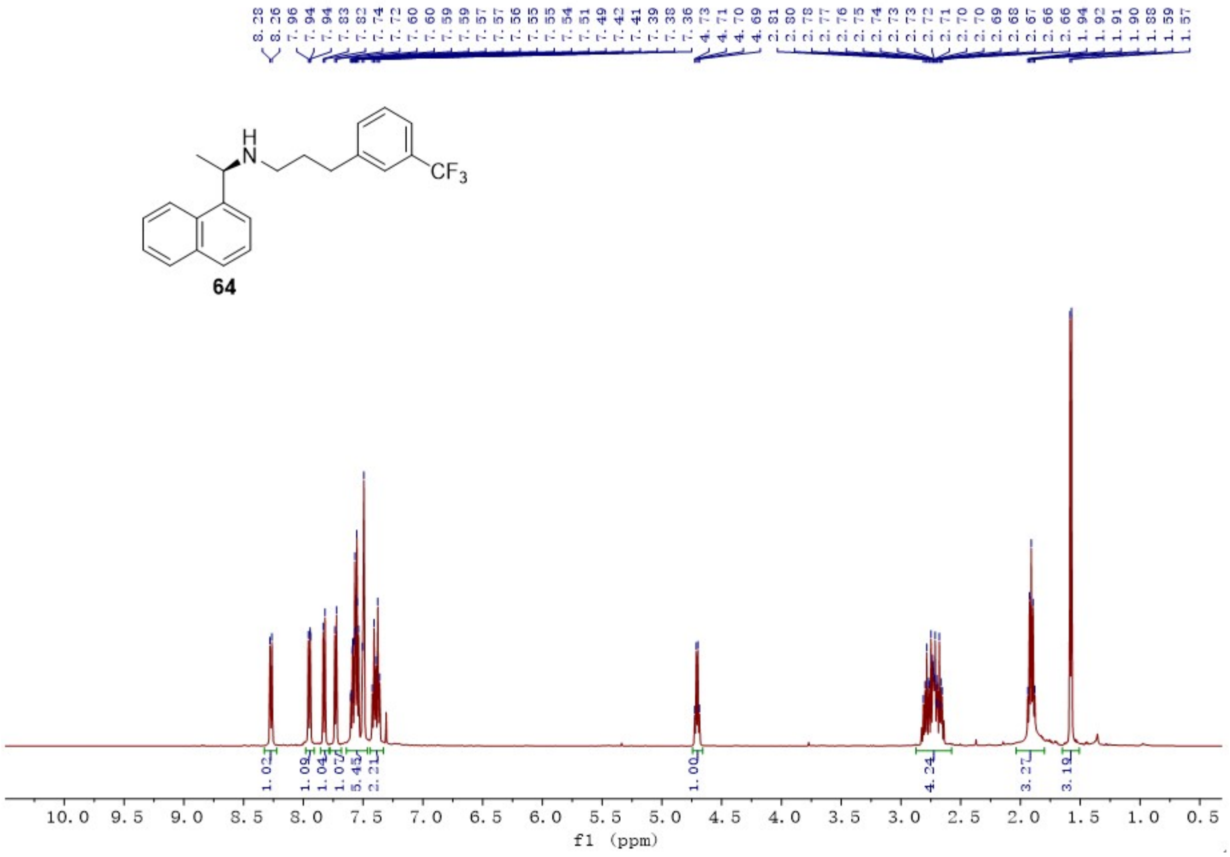

Supplementary Figure 194. ^{1}H NMR spectrum of **64** in $CDCl_3$.

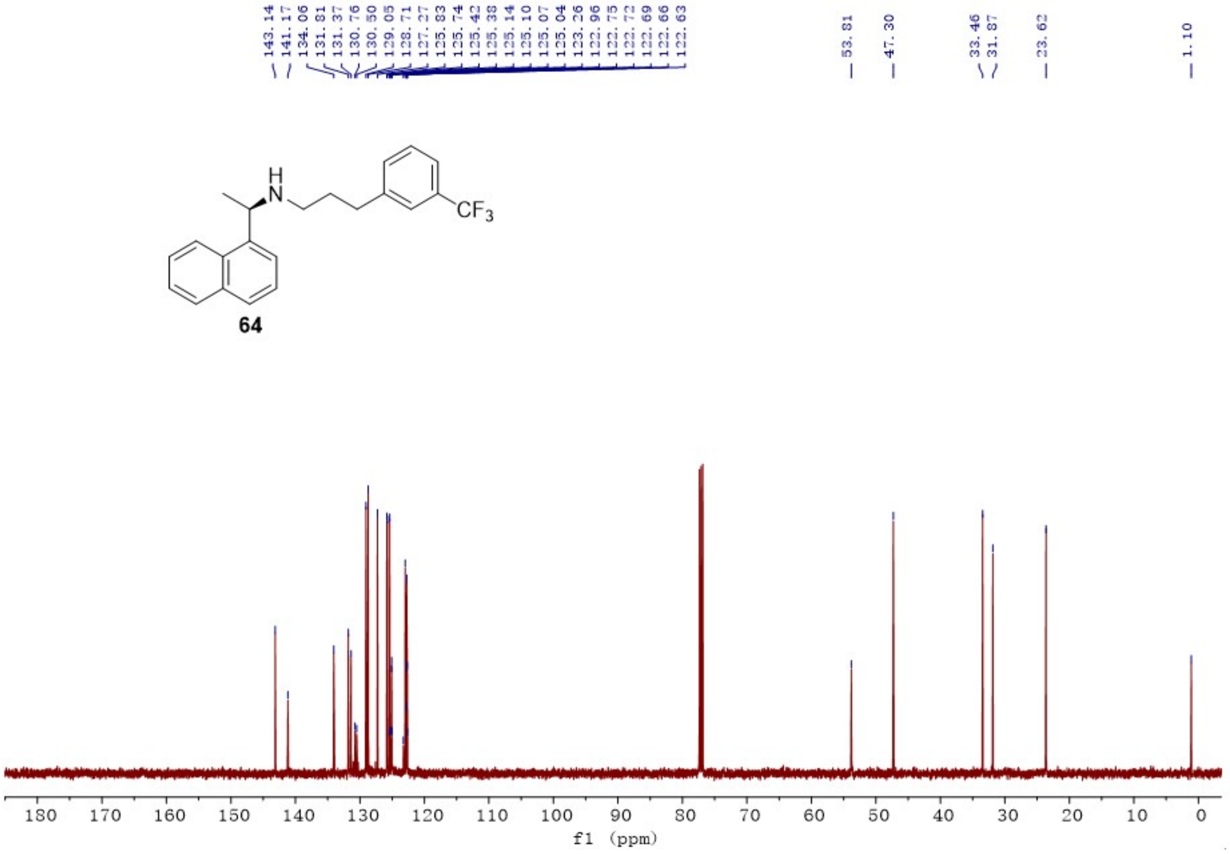

Supplementary Figure 195. ^{13}C NMR spectrum of **64** in $CDCl_3$.

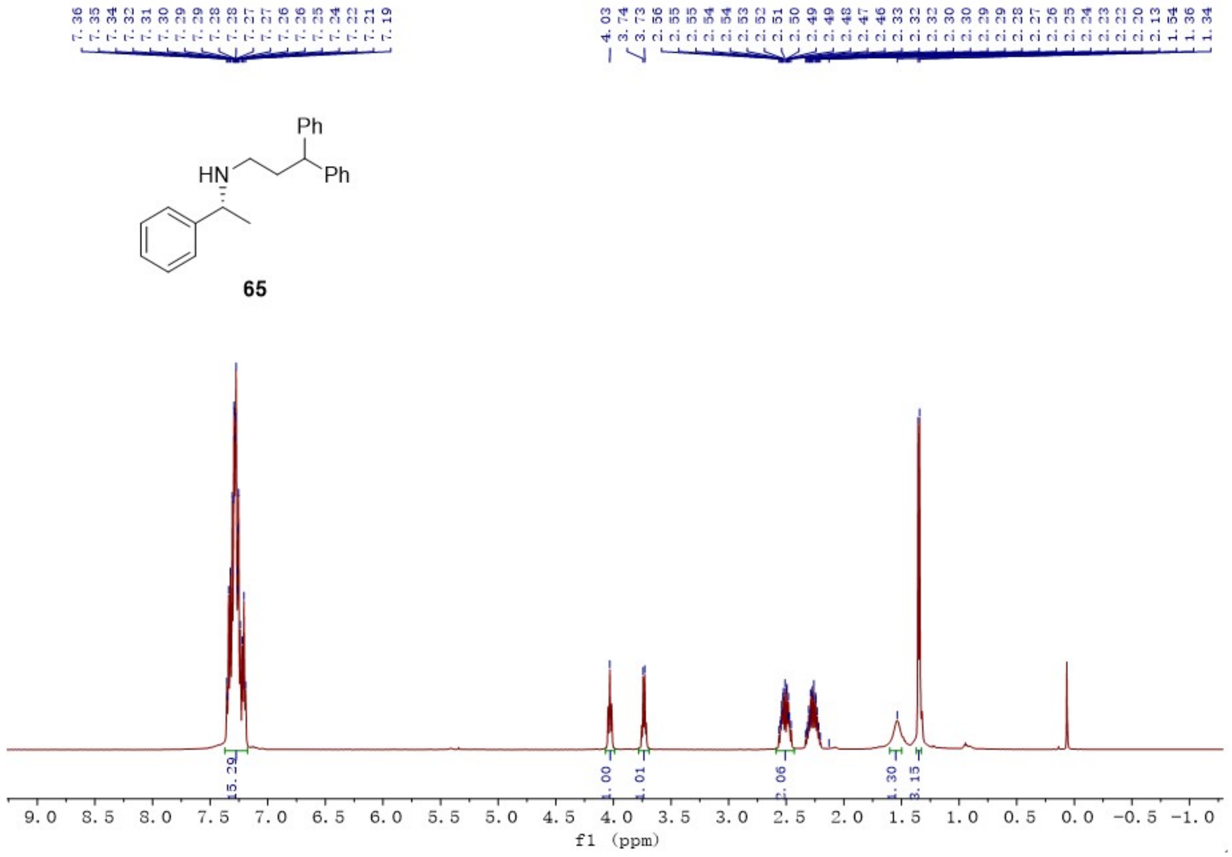

Supplementary Figure 196. ^{1}H NMR spectrum of **65** in $CDCl_3$.

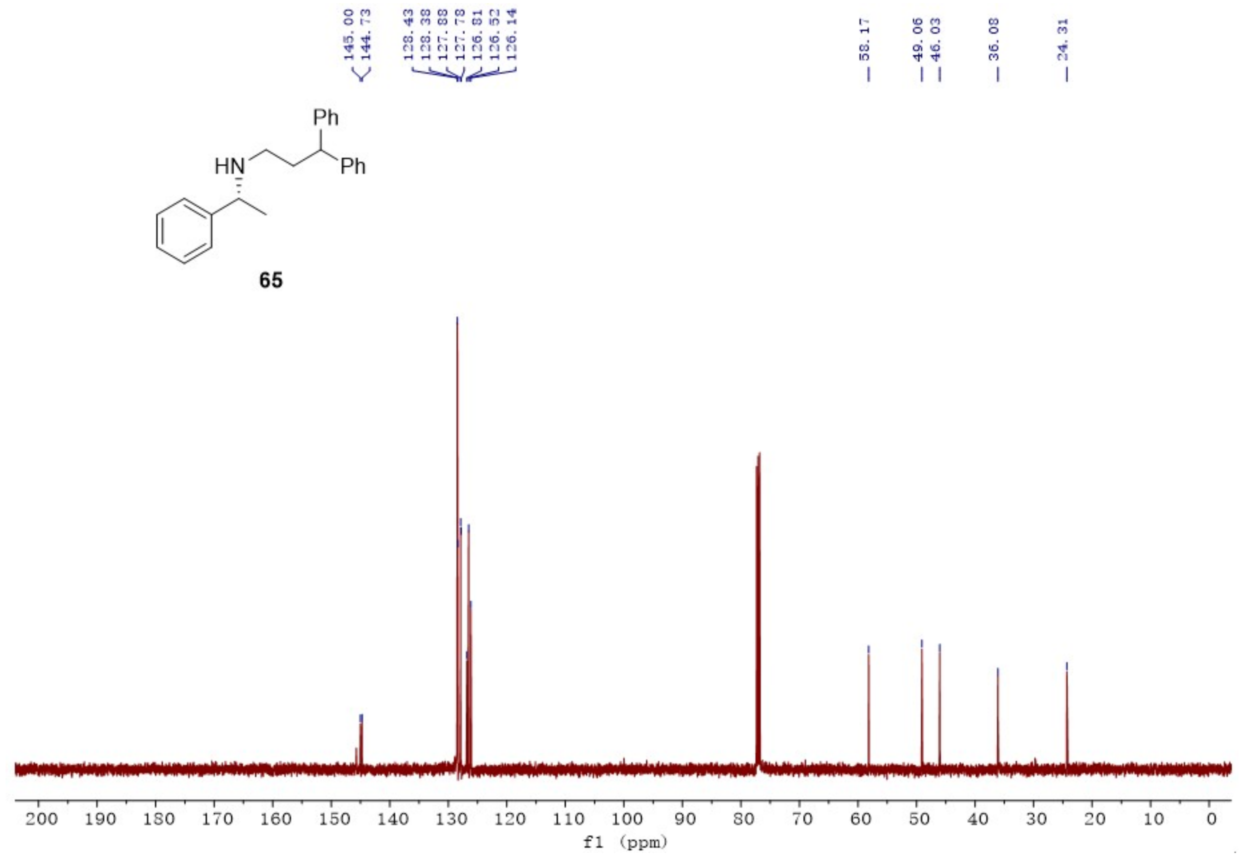

Supplementary Figure 197. ^{13}C NMR spectrum of **65** in $CDCl_3$.

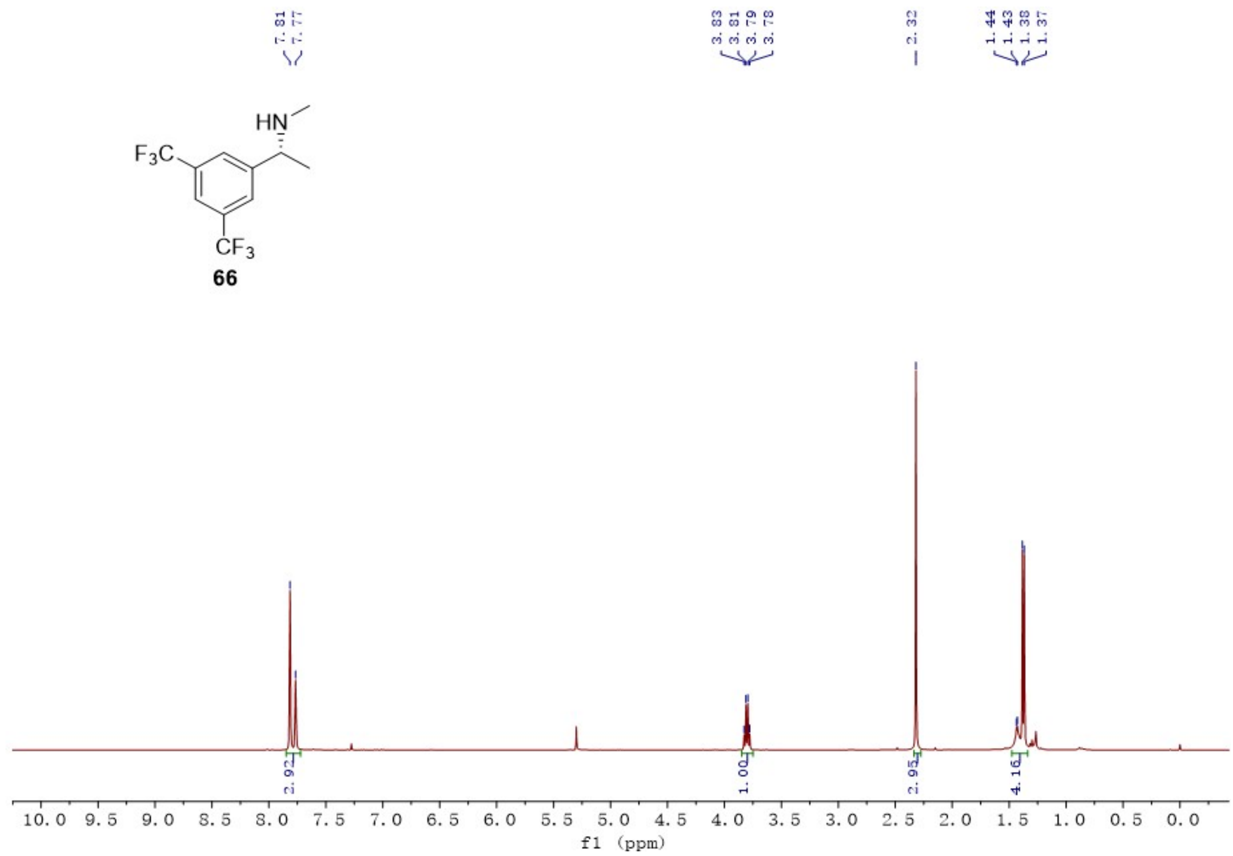

Supplementary Figure 198. ^{1}H NMR spectrum of **66** in $CDCl_3$.

5. Supplementary References

1. W. Hu, J. Zhou, X. Xu, W. Liu, L. Gong, *Org. Synth*. **2011**, *88*, 406-417.
2. W.-B. Liu, C. Zheng, C.-X. Zhuo, L.-X. Dai, S.-L. You, *J. Am. Chem. Soc.* **2012**, *134*, 4812–4821.
3. B. Zhang, T. Guo, Y. Liu, F. E. Kühn, C. Wang, Z. K. Zhao, J. Xiao, C. Li, T. Zhang, *Angew. Chem. Int. Ed.* **2021**,*60*, 20666.
4. B. C. Van Wagenen, S. T. Moe, M. F. Balandrin, E. G. Delmar, E. F. Nemeth, US6211244B1, 2001.
5. D. I. B. Kerr, J. Ong, M. V. Perkins, R. H.Prager, *Aust. J. Chem*. 2006, 59, 445–456.
6. L. Dwoskin, D. Watt, J. Thorson, M. Leggas, K. Guy, J. Hammill, S. Kwiatkowski, D. Ding, G. Zheng, P. A. Crooks, N.-R.Lee, US20200290948A1, 2020.
7. C. D. Matier, J. Schwaben, J. C. Peters, G. C. Fu, *J. Am. Chem. Soc*. **2017**, *139*,17707.
8. H. Eggert, C. Djerassi, *J. Am. Chem. Soc*. **1973**, *95*, 3710.
9. S. Liang, P. Monsen, G. B. Hammond, B. Xu, *Org. Chem. Front.* **2016**, *3*, 505.
10. L. A. Walter, W. K. Chang, J. McGlotten, R. Foester, *J. Heterocycl. Chem*. **1977**, *14*, 47.
11. P. Kang, K. M. Lee, W. K. Lee, K. H. Lee, B. Lee, J. Cho, N. H. Hur, *RSC Adv.* **2014**, *4*, 46203.
12. E. Salomo, A. Gallen, G. Sciortino, G. Ujaque, A. Grabulosa, A. Lledos, A. Riera, X. Verdaguer, *J. Am. Chem. Soc*. **2018**, *140*, 16967.
13. J. Genovino, D. Sames, B. B. Toure, *Tetrahedron Lett*. **2015**, *56*, 3066.
14. C. M. Brandel, J. W. B. Cooke, R. A. J. Horan, F. P. Mallet, D. R. Stevens, *Org. Process. Res. Dev*. **2015**, *19*, 1954.
15. Gaussian 09, Revision D.01, M. J. Frisch, G. W. Trucks, H. B. Schlegel, G. E. Scuseria, M. A. Robb, J. R. Cheeseman, G. Scalmani, V. Barone, B. Mennucci, G. A. Petersson, H. Nakatsuji, M. Caricato, X. Li, H. P. Hratchian, A. F. Izmaylov, J. Bloino, G. Zheng, J. L. Sonnenberg, M. Hada, M. Ehara, K. Toyota, R. Fukuda, J. Hasegawa, M. Ishida, T. Nakajima, Y. Honda, O. Kitao, H. Nakai, T. Vreven, J. A. Montgomery, Jr., J. E. Peralta, F. Ogliaro, M. Bearpark, J. J. Heyd, E. Brothers, K. N. Kudin, V. N. Staroverov, T. Keith, R. Kobayashi, J. Normand, K. Raghavachari, A. Rendell, J. C. Burant, S. S. Iyengar, J. Tomasi, M. Cossi, N. Rega, J. M. Millam, M. Klene, J. E. Knox, J. B. Cross, V. Bakken, C. Adamo, J. Jaramillo, R. Gomperts, R. E. Stratmann, O. Yazyev, A. J. Austin, R. Cammi, C. Pomelli, J. W. Ochterski, R. L. Martin, K. Morokuma, V. G. Zakrzewski, G. A. Voth, P. Salvador, J. J. Dannenberg, S. Dapprich, A. D. Daniels, O. Farkas, J. B. Foresman, J. V. Ortiz, J. Cioslowski, and D. J. Fox, Gaussian, Inc., Wallingford CT, 2013.
