# Supplementary material for: Iridium-catalyzed direct asymmetric reductive amination utilizing primary alkyl amines as the N-sources
Source: Nat Commun. 2022 Jun 10;13:3344. doi: 10.1038/s41467-022-31045-5 (PMC9187633; doi:10.1038/s41467-022-31045-5)
Supplement: Supplementary file 3 — Description of Additional Supplementary Files [file 41467_2022_31045_MOESM3_ESM.docx]

1. File Name: Supplementary Data 1
   Description: Cartesian coordinates of optimized structures for the “Out-sphere” pathways in CF_3_CH_2_OH.
2. File Name: Supplementary Data 2
   Description: Cartesian coordinates of optimized structures for the “Out-sphere” pathways in EtOAc.
3. File Name: Supplementary Data 3
   Description: Cartesian coordinates of optimized structures for the “inner-sphere” pathways in CF_3_CH_2_OH.
